# Application of seed micromorphology in taxonomy of the genus *Polystachya* Hook. (Vandeae, Orchidaceae)

**DOI:** 10.3389/fpls.2026.1761768

**Published:** 2026-05-18

**Authors:** Katarzyna Sanek, Sławomir Nowak, Przemysław Baranow, Marta Kolanowska, Konrad Kaczmarek, Ann Bogaerts, Sofie De Smedt, Agnieszka Rewicz

**Affiliations:** 1 Department of Geobotany and Plant Ecology, Faculty of Biology and Environmental Protection,University of Łódź, Łódź, Poland; 2 Department of Plant Taxonomy and Nature Conservation, University of Gdańsk, Gdańsk, Poland; 3 Meise Botanic Garden, Meise, Belgium

**Keywords:** anticlinal wall, herbarium specimens, Polystachyinae, seed coat cells, SEM

## Abstract

**Introduction:**

Seed morphology is widely used as an auxiliary source of evidence in orchid systematics, but its taxonomic value varies among lineages. For several genera within Vandeae, the extent to which seed traits reflect phylogenetic relationships or provide diagnostic features remains insufficiently evaluated.

**Methods:**

To assess the systematic relevance of seed characters, we examined a broad set of qualitative and quantitative traits across representatives of the genus *Polystachya*, including seed length, shape, and surface architecture. Measurements were obtained from scanning electron microscopy, and patterns of variation were compared within and among taxa to identify traits with potential diagnostic utility.

**Results:**

The analyses revealed that some seed characters display consistent patterns within *Polystachya*, while others show overlapping ranges that limit their diagnostic value. In several cases, seed length and anticlinal wall features proved informative for distinguishing certain species, whereas surface ornamentation was more variable. No single character was universally reliable, but combined trait assessment increased discriminatory power.

**Discussion:**

The results demonstrate that seed morphology provides a useful, though not standalone, source of taxonomic information within the studied group. Patterns observed across taxa indicate that seed traits can reinforce existing hypotheses of relationships and highlight potential discrepancies where boundaries may require reassessment. The findings emphasize the value of integrating seed morphology with molecular and ecological evidence to improve the resolution of taxonomic questions in *Polystachya*.

## 1 Introduction

The orchid family (Orchidaceae) is among the most diverse and taxonomically challenging groups of angiosperms. Since the first classification attempts by Linnaeus, orchid systematics have been repeatedly revised and refined (Kozłowski et al., 2009). Comprehensive classifications (e.g., Dressler, 1993; Szlachetko, 1995) established important frameworks, yet higher-level relationships and generic delimitations remain unresolved (Fay and Chase, 2009). This persistent uncertainty results largely from the family’s extraordinary morphological diversity and immense species richness, currently estimated at nearly 750 genera and more than 30,000 species (Fay et al., 2025). While molecular phylogenetics has significantly changed our understanding of evolutionary relationships within Orchidaceae (Cameron, 2010), it has not fully resolved all taxonomic challenges, particularly in groups marked by morphological complexity or homoplasy. Moreover, the continual discovery of approximately 500 new species each year further complicates generic and sectional boundaries (Chase et al., 2015) These dynamics highlight the need for an integrative taxonomic framework that synthesizes molecular, morphological, anatomical, and ecological data.

Traditionally, orchid taxonomy has relied heavily on macromorphological traits such as floral architecture, leaf form, and vegetative habit (Chase et al., 2003; Kolanowska, 2021). However, these characters are frequently shaped by environmental factors, developmental plasticity, or convergent evolution, complicating both species delimitation and higher-level classification. Consequently, researchers have increasingly turned toward alternative and less explored sources of data, including seeds. Numerous studies have demonstrated that both qualitative and quantitative seed characters offer diagnostic and phylogenetic insights across many plant families (Barthlott and Ziegler, 1981; Gamarra et al., 2007; Latowski et al., 2015; Uğurlu Aydın and Dönmez, 2019). In Orchidaceae, scanning electron microscopy (SEM) has revealed a far greater diversity of seed ultrastructure than previously recognised (Senghas et al., 1974; Kurzweil, 1993), and these traits have been effectively applied to phylogenetic and taxonomic problems in several lineages (Akçin et al., 2009; Gamarra et al., 2012; Collier et al., 2023). The extensive survey by Barthlott et al. (2014), encompassing more than 1000 species from 350 genera, showed that micromorphological features often carry substantial phylogenetic signal and can serve as a valuable complement to molecular evidence.

Seed micromorphology is therefore gaining prominence in orchid systematics. A recent study on the subtribe Angraecinae by Gamarra et al. (2025) illustrates this trend particularly well. By examining seeds of 121 species from 38 genera, the authors identified a suite of stable ultrastructural characters — including elongated testa cells, thickened and arched anticlinal walls, and deeply sunken periclinal walls — whose distribution among taxa corresponded closely to relationships inferred from established phylogenetic analyses. More subtle traits, such as seed outline and testa cell arrangement, further helped resolve long-standing taxonomic ambiguities and supported the segregation of broad, heterogeneous groups like *Angraecum* Bory sensu lato. This work indicates that, although seed traits have long been documented, their potential contribution to refining molecular hypotheses and clarifying evolutionary relationships remains insufficiently explored.

The study by Gamarra et al. (2025) demonstrates that seed micromorphological characters can provide taxonomically informative signals within the tribe Vandeae, as exemplified by Angraecinae, one of its major subtribes. These results challenge earlier assumptions that seed traits are of limited systematic value in Vandeae. For instance, Dressler (1993) stated that all members of the subtribe Polystachyinae produce Vanda-type seeds, and therefore considered seed characters taxonomically uninformative within this group. However, recent evidence suggests that, even within Vanda-type seeds, additional micromorphological features may contribute useful characteristics for taxonomic delimitation and for reassessing relationships within Polystachyinae (Gamarra et al., 2025).

The genus *Polystachya* Hook. represents one of the most taxonomically intricate groups of orchids. Comprising roughly 240 species distributed across Africa, Madagascar, the Neotropics, and parts of Asia (Jiang et al., 2022; Mytnik et al., 2021), *Polystachya* displays pronounced morphological variability in both vegetative and floral characters and a complex nomenclatural history, with nearly 500 names associated with currently recognized taxa. Kraenzlin’s (1926) early division of the genus into 12 sections has been repeatedly revised, with subsequent authors variously expanding or reducing sectional limits. The most recent treatment recognizes 13 sections (Mytnik-Ejsmont, 2011), yet even this framework remains provisional, as molecular studies continue to reveal discordances between morphology-based groupings and phylogenetic relationships (Russell et al., 2010). Moreover, Mytnik-Ejsmont (2011) proposed a broader concept of the subtribe Polystachyinae, separating *Polystachya* into eight new, smaller genera.

Although the number of carpological studies on various orchid groups is steadily increasing, seeds of the genus *Polystachya* remain poorly known, and the available literature on this topic is limited. On the other hand, there are many publications describing the anatomy and morphology of the genus. Carpological literature is limited to a general characterization of *Polystachya* seeds, classifying them as representatives of the Vandeae type and attributing to them typical traits of this group (Barthlott et al., 2014; Dressler, 1993; Mytnik-Ejsmont, 2011), often highlighting the high seed similarity within the tribe and the limited taxonomic usefulness. There are also works that provide general traits narrowed down for the genus *Polystachya*. For example, Barthlott et al. (2014) does not present a detailed set of quantitative and qualitative traits for individual species examined, only generalized data for the entire genus were included. Similarly, Gamarra et al. (2018) present only qualitative data on the entire genus *Polystachya*, including seed shape, type of anticlinal and periclinal walls, and ornamentation type. More detailed descriptions are given only for selected features of three species: *P. concreta* (Jacq.) Garay & H.R. Sweet, *P. fusiformis* (Thouars) Lindl., and *P. polychaete* Kraenzl. The features of these species, presented with SEM images, are mostly given as examples illustrating the variation of particular morphological aspects among genera analyzed in the study. So far, only individual species have been analyzed, and these studies do not consider a full set of traits (Lallana et al., 2019; Ochora 2001). Lallana et al. (2019) present morphometric data on length, width, shape, color, and average seed mass for only *P. concreta*. The most comprehensive study by Ochora (2001) contains metric data (average length and width of seeds and testa cells, as well as the number of testa cells with standard deviations) and qualitative data (seed shape and color, traits related to testa cells, type of periclinal and anticlinal walls). However, it should be noted that these data concern only twelve species.

No comprehensive investigation has yet synthesized seed micromorphology across the genus or assessed the taxonomic value of these traits within *Polystachya*. Given the demonstrated utility of seed micromorphology in other orchid groups, this knowledge gap is particularly striking. For these reasons, the present study aims to examine seed coat morphology among selected species of *Polystachya*, with the broader goal of determining the usefulness of these characters in the systematics of the genus. Specifically, we evaluate whether micromorphological traits of the seed coat possess diagnostic value and whether they can serve as reliable criteria for species identification and classification within *Polystachya* and subtribe Polystachyinae.

## 2 Materials and methods

A total of 228 specimens representing the broadly defined genus *Polystachya* Hook. were examined. Twelve of these specimens represent species that, according to the most recent revision of Polystachyinae Schltr (Mytnik-Ejsmont, 2011), were treated as belonging to separate genera – *Chelystachya* Mytnik & Szlach., *Dendrobianthe* (Schltr.) Mytnik, *Epiphorella* Mytnik & Szlach., and *Unguiculabia* Mytnik & Szlach. Nevertheless, for the purposes of the present study, a wider circumscription of *Polystachya* was adopted (see Discussion). The analyzed material represents 69 taxa. A complete list of species together with their corresponding voucher numbers is provided in **Supplementary Table 1**. Material for the study was obtained from mature seed capsules, which were collected and classified as herbarium specimens by the following seven herbaria: UGDA, MA, BR, B, P, WAG, MO (Thiers 2025). The number of seeds used per sample ranged from 3 to 30, depending on the quality and availability of material. The number of samples was determined by the availability of herbarium specimens bearing fruits and, at the same time, allowing for reliable taxonomic identification. Consequently, the number of samples differed among species (**Supplementary Table 1**). An exception was *P. concreta*, which was sampled more extensively (45 samples) because of its high morphological variability and pantropical distribution. This increased sampling effort was intended to adequately represent the observed variation in seed morphology within the species.

In this study, the term *sample* refers to a single analyzed sample obtained from a studied herbarium specimen. In many cases, several samples from different herbarium specimens, representing the same species were analyzed. The term *species* or *taxon*, in turn, is used collectively to refer to all samples analyzed within a given species.

### 2.1 Scanning electron microscope analyses

Seed micromorphology was analyzed using a scanning electron microscope (SEM – Phenom Pro X, Thermo Fisher Scientific, Waltham, USA) at the Department of Invertebrate Zoology and Hydrobiology, University of Łódź, Poland. Seeds were mounted on 12 mm carbon tabs (Agar Scientific, PIK Instruments, Poland) attached to sample stubs (Ø 12.5 mm, PIN 3.2×8 mm) and then sputter-coated with a 4 nm thick layer of gold. For selected samples, especially those with distinctly different surface textures, 3D models of seed surface ultrastructure were generated using the 3D Roughness Reconstruction software for the Phenom SEM. The obtained SEM images of seeds were compiled into plates for each analyzed specimen (**Supplementary Figures S1-S183**).

### 2.2 Quantitative and qualitative analysis

For metric analyses, fully developed and undamaged seeds were randomly selected from each sample. Their condition was verified using a stereomicroscope (Nikon SMZ-800 DS-Fi equipped with a DeltaPix Invenio 12EIII, 12 MegaPixel Microscope Camera). Seeds were characterised using five metric traits: seed length (SL), seed width (SW), seed circumference (SC), seed area (SA), and seed volume (SV). Seed volume (SV) was calculated following Arditti et al. (1979, 1980) using the formula: SV = 2 × [(SW²) × 2 × (½SL) × 1.047], where 1.047 = π/3.13. All measurements were compiled in a table of metric data (**Supplementary Table 2**). Seed length and width were recorded in micrometers (µm). Seed length was assigned to five size categories according to Barthlott et al. (2014) modified: very small: 100–200 µm, small: 201–300 µm, medium: 301–400 µm, large: 401–500 µm, very large: 501–610 µm (**Supplementary Table 3**).

Qualitative seed characters were assessed based on SEM micrographs (**Supplementary Table 3**). Seed shape terminology followed Barthlott et al. (2014), modified and included the following categories: fusiform, fusiform-ellipsoid, ellipsoid, ellipsoid-ovoid, and ovoid. The shape of the seed apex was classified into four types: cuneate, cuneate-rounded, rounded, and rectangular. The number of testa cells observed along the longitudinal axis was categorized as either few (≤5) or many (≥6).

The shape of individual seed coat cells was described following Barthlott et al. (2014); Arditti et al., 1979, 1980 – modified, distinguishing between elongated irregular and elongated regular cells. Additionally, the apex of testa cells was classified as either rounded or rectangular-rounded. The appearance of the anticlinal walls was described according to Barthlott et al. (2014) and was consistently straight. The visibility of the periclinal wall was noted as either visible or not visible; when visible, its surface structure was described as smooth or absent (**Supplementary Table 3**).

### 2.3 Statistical analyses

For the metric seed characters, basic descriptive statistics were calculated, including the arithmetic mean ( $\bar{x}$), minimum and maximum values, standard deviation and the coefficient of variation (**Supplementary Table 4**). Similarities and relationships among taxa were assessed using Gower’s general similarity coefficient (Gower 1971), which enables the combined analysis of both quantitative and qualitative characters. Morphological affinities among taxa were further evaluated using cluster analysis performed with the UPGMA method (ang. *Unweighted Pair-Group Method using arithmetic averages*). For this analysis, qualitative seed characters were encoded numerically, while quantitative data were used directly (**Supplementary Table 2**). Principal Component Analysis (PCA) was also conducted to investigate the spatial distribution of taxa based on all quantitative traits. All numerical analyses were carried out using STATISTICA PL v.13.3 (StatSoft Inc., 2011) and PAST v.4.03. A 3D PCA visualization was generated using Python 3.11.7 (Python Software Foundation, 2024) within the Anaconda 24.1.2 environment (Anaconda Software Distribution, 2024). The analysis employed the following libraries: ‘pandas’ v2.1.4, ‘numpy’ v1.26.4, ‘plotly’ v5.9.0, ‘scipy’ v1.11.4, and ‘scikit-learn’ v1.2.2.

## 3 Results

### 3.1 Variation in quantitative traits

The analysis of quantitative seed traits revealed substantial variation in seed length among the studied taxa. Seed length ranged from 115.29 μm in *Polystachya reflexa* Lindl. (P. ref) to 609.40 μm in *P. doggettii* Rendle & Rolfe (P. dog) (see **Supplementary Table 2**). Most taxa of the genus *Polystachya* produced very small to small seeds.

A large proportion of taxa of the genus *Polystachya* produced very small (41% of the samples) to small (40% of the samples) seeds. The medium-sized seed group (301–400 μm) comprised approximately 13% of the samples and included species such as *P. bennettiana* Rchb. f. (P. ben) and *P. microbambusa* Kraenzl. (P. mic).

Taxa producing large seeds (401–500 μm) were much less numerous, representing about 4% of the measured samples, including *P. dendrobiiflora* Rchb. f. (P. den2) and *P. melanantha* Schltr. (P. mel). Very large seeds (>500 μm) were the rarest, constituting only about 2% of all recorded values. These were observed in only three taxa: *P. dendrobiiflora* (P. den3), *P. doggettii* (P. dog), and *P. piersii* P. J. Cribb (P. pie) (**Supplementary Tables 2, 3**).

The examined samples exhibited substantial variation in seed width, circumference, surface area, and volume (**Supplementary Table 2**). The narrowest seeds were recorded in *Polystachya adansoniae* Rchb. f. (P. ada7), with an average width of 29.23 μm, while the widest seeds were observed in *P. dendrobiiflora* (P. den2), reaching 169.15 μm. Considerable diversity was also noted in seed circumference and surface area. The smallest circumference, 275.25 μm, occurred in *P. reflexa* (P. ref), whereas the largest, 1404.63 μm, was characteristic of *P. doggettii* (P. dog). A similar pattern was found for seed surface area: *P. reflexa* (P. ref) exhibited the lowest value (3097.05 μm²), while the seeds of *P. doggettii* (P. dog) reached the highest recorded area (66617.23 μm²). Seed volume also varied across taxa. The smallest average volume was identified in *P. cultriformis* (Thouars) Spreng. (P. cul10), measuring 0.18 μm³, whereas *P. polychaete* (P. pol2) displayed the largest volume at 0.50 μm³.For seed length, values ranged from 5.78% in *P. polychaete* (P. pol4) to 27.05% in *P. pachychila* Summerh. (P. pach2). Seed width showed the lowest variability in *P. hastata* Summerh. (P. has2) (7.15%) and the highest in *P. caduca* Rchb. f. (P. cad) (27.94%). The smallest variability in circumference was recorded in *P. concreta* (P. con13) (5.18%), while the greatest again occurred in *P. pachychila* (P. pach2) (25.22%). For surface area, the lowest value of the coefficient of variation was found in *P. cultriformis* (P. cul4) (5.36%), and the highest in *P. pachychila* (P. pach2) (44.89%).

### 3.2 Variation in seed shape

**Figure 1:**
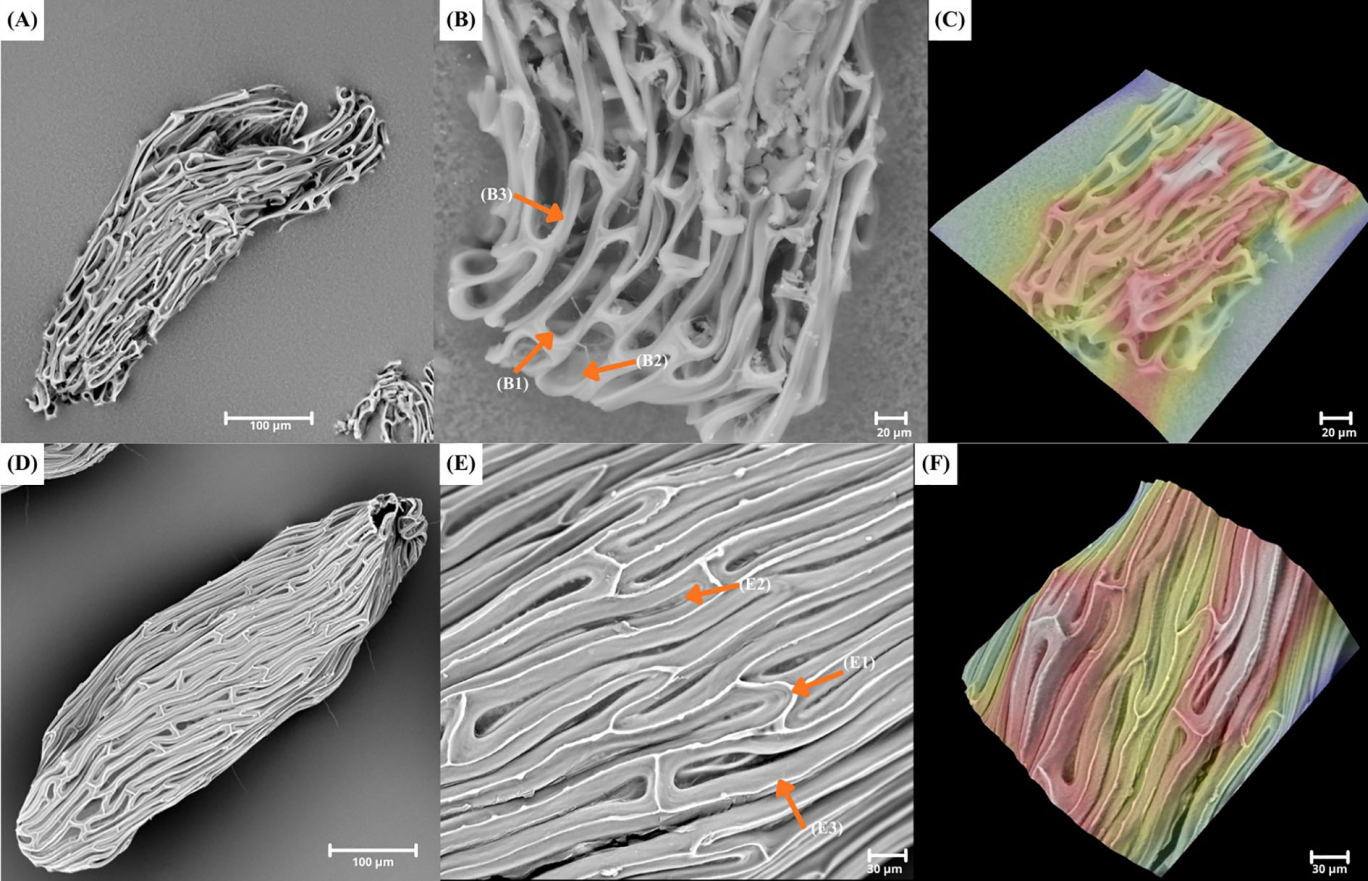
A plate showing SEM and 3D pictures of **(A–C)** *Polystachya affinis* (P.aff2) with ellipsoid seed, rounded apex; rectangular-rounded apex of testa cells (B1), smooth periclinal wall (B2) and elongated irregular shape of individual seed coat cells (B3); **(D–F)** *Polystachya dendrobiiflora* (P.den2) with ellipsoid seed, rounded apex; rounded apex of testa cells (E1), smooth periclinal wall (E2) and elongated irregular shape of individual seed coat cells (E3). Seeds of both species with straight anticlinal walls.

**Figure 2:**
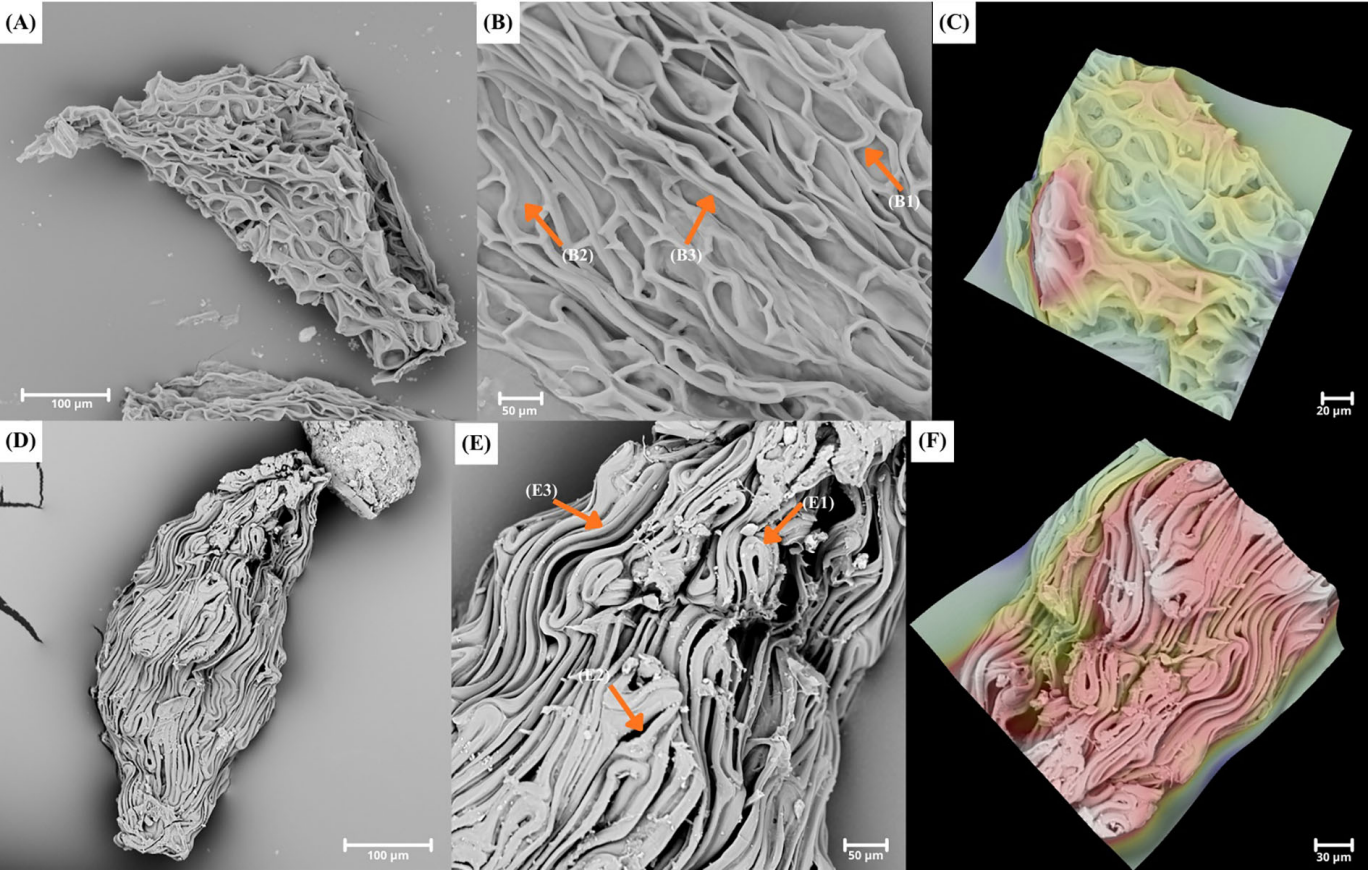
A plate showing SEM and 3D pictures of **(A–C)** *Polystachya fractiflexa* (P.fra) with ellipsoid-ovoid seed, cuneate apex; rounded apex of testa cells (B1), smooth periclinal wall (B2) and elongated irregular shape of individual seed coat cells (B3); **(D–F)** *Polystachya piersii* (P.pie) with ellipsoid-ovoid seed, cuneate apex; rounded apex of testa cells (E1), not visible periclinal wall (E2) and elongated irregular shape of individual seed coat cells (E3).

**Figure 3:**
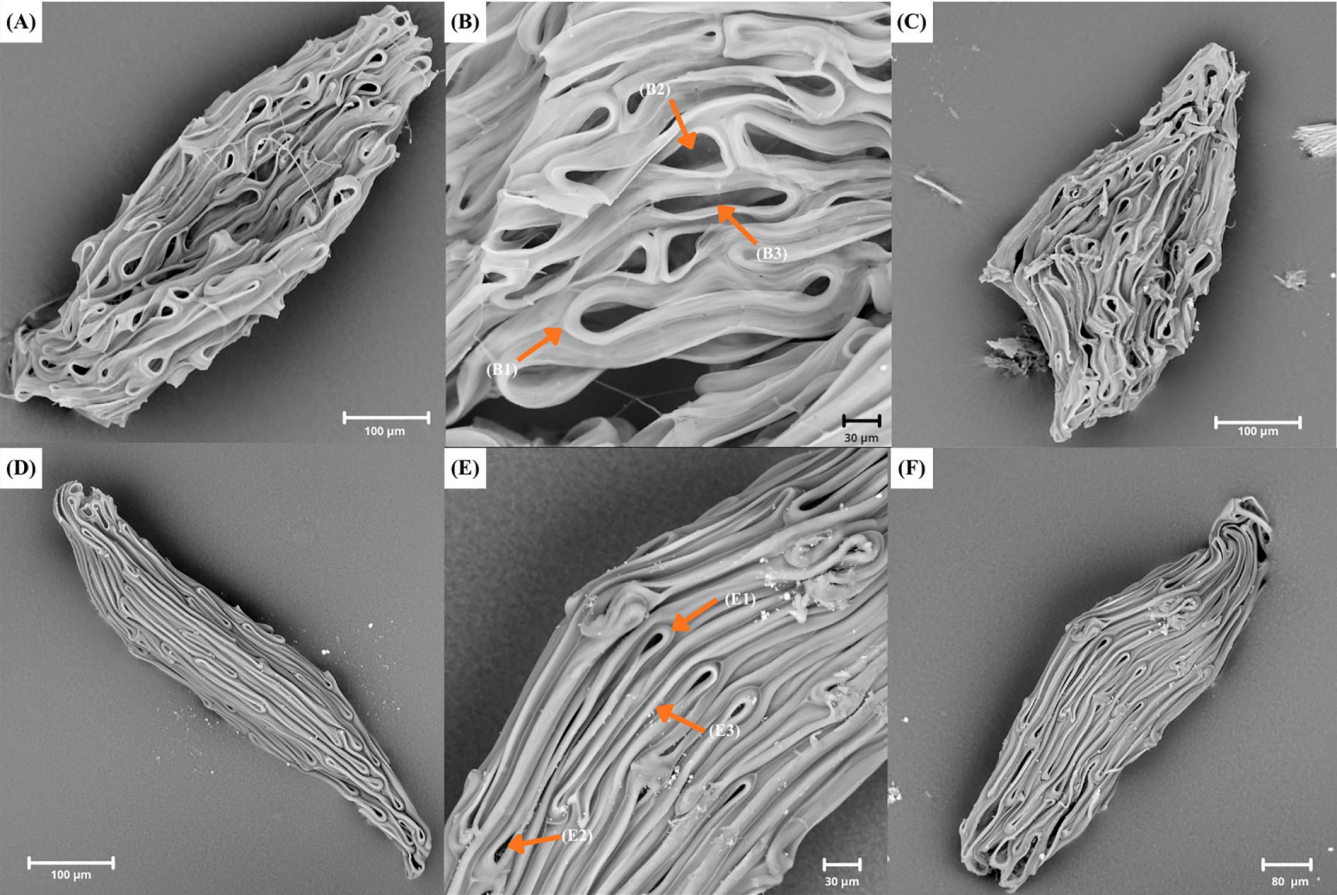
A plate showing SEM pictures of **(A–C)** *Polystachya rhodoptera* (P.rho5) with ellipsoid-ovoid seed, rounded apex; rounded apex of testa cells (B1), smooth periclinal wall (B2) and elongated irregular shape of individual seed coat cells (B3); **(D–F)** *Polystachya transvaalensis* (P. tra3) with fusiform-ellipsoid seed, cuneate apex; rounded apex of testa cells (E1), not visible periclinal wall (E2) and elongated regular shape of individual seed coat cells (E3).

**Figure 4:**
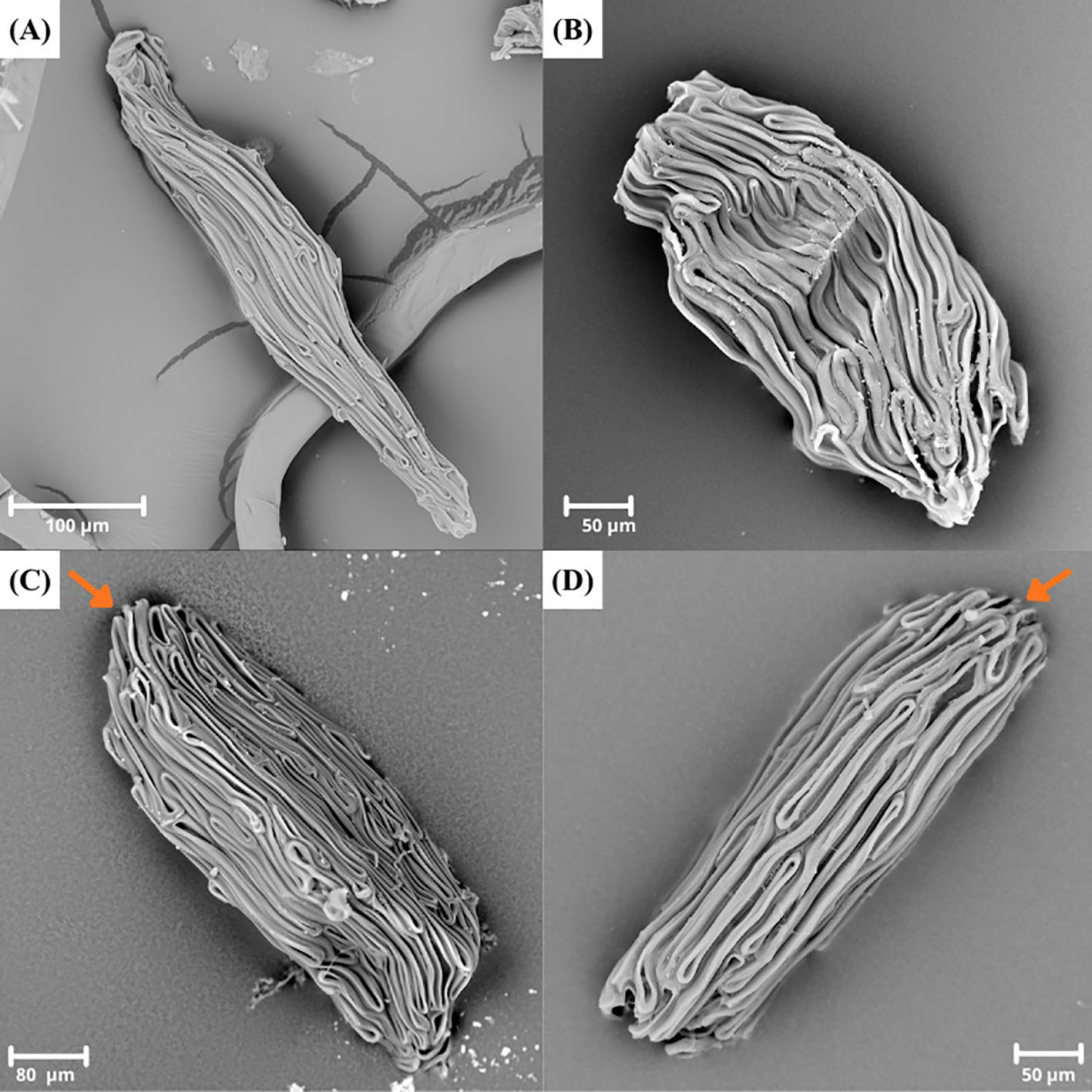
A plate showing SEM pictures of **(A)** *Polystachya albescens* ssp. *imbricata* (P. alb. ssp. imb) with fusiform seed; **(B)** *Polystachya concreta* (P. con45) with ovoid seed; **(C)** *Polystachya aconitiflora* (P. aco2) with a cuneate-rounded apex (orange arrow) and **(D)** *Polystachya calluniflora* (P. cal) with a rectangular apex (orange arrow).

Most of the analyzed seeds were ellipsoid (**Figures 1A, D**), this category included as many as 94 specimens (e.g., *Polystachya aconitiflora* Summerh. – P. aco, *P. albescens* Ridl. ssp. *musozensis* (Rendle) Summerh. – P. alb. subsp. mus, *P. doggettii* – P. dog). The second most common seed form was ellipsoid-ovoid (**Figures 2A**, **3A**), recorded in 55 specimens (e.g., *P. paniculata* (Sw.) Rolfe – P. pan, *P. villosa* Rolfe – P. vil, *P. zambesiaca* Rolfe – P. zam2). Other seed shapes occurred far less frequently. Fusiform seeds were found in 14 specimens (e.g., *P. bifida* Lindl. – P. bif, *P. mildbraedii* Kraenzl. var. *angustifolia* (Summerh) Geerinck – P. mil. var. ang, *P. rhodoptera* Rchb. f. – P. rho3) (**Figure 4A**). Ovoid seeds were observed in 12 specimens (e.g., *P. foliosa* (Hook.) Rchb. f. – P. fol2, *P. galeata* (Sw.) Rchb. f. – P. gal2, *P. seticaulis* Rendle – P. set2) (**Figure 4B**). The rarest category comprised fusiform-ellipsoid seeds, recorded in 8 specimens (e.g., *P. laxiflora* Lindl. – P. lax2, *P. odorata* Lindl. ssp. *gabonensis* (Summerh.) Stévart – P. ssp. gab, *P. transvaalensis* Schltr. – P. tra2) (**Figures 3D, F**).

### 3.3 Variation in seed apex shape

Seeds with cuneate apexes were by far the most common among the studied taxa, occurring in 131 specimens (**Figures 2A, D**, **3D, F**). Representative examples include *Polystachya affinis* Lindl. – P. aff, *P. caduca* – P. cad, and *P. doggettii* – P. dog. The second most frequent apex type was rounded, found in 35 specimens (e.g., *P. elegans* Rchb.f. *–* P. ele, *P. odorata* Lindl. – P. odo6, *P. pachychila* – P. pach) (**Figures 1A, D**, **3A, C**). Less common were cuneate-rounded apexes, observed in 10 specimens (e.g., *P. clavata* Lindl. – P. cla, *P. galeata* – P. gal, *P. pachychila* – P. pach2) (**Figure 4C**). The rarest category comprised rectangular apexes, present in 7 specimens (e.g., *P. adansoniae* – P. ada13, *P. poikilantha* Kraenzl. var. *poikilantha* – P. poi. var. poi, *P. stuhlmannii* Kraenzl. – P. stu) (**Figure 4D**).

### 3.4 Variation in seed coat cells

Most of the analyzed seeds (99 samples) were characterized by a small number of testa cells (<5), whereas a larger number of cells (>6) was recorded in 84 specimens (**Supplementary Table 3**). Analysis of the shape of individual testa cells showed that in most specimens (110), the cells were elongated and regular (**Figure 3E**). This type of cell occurs, for example, in *Polystachya golungensis* Rchb. f. (P. gol), *P. melanantha* (P. mel.), and *P. ruwenzoriensis* Rendle (P. ruw). In contrast, elongated and irregular testa cells were considerably less common, occurring in 73 specimens (**Figures 1B, C, E, F**, **2B, C, E, F**), for example, *P. affinis* (P. aff2), *P. dendrobiiflora* (P. den2), *P. mildbraedii* Kraenzl. var. *mildbraedii* (P. mil. var. mil) and *P. polychaete* (P. pol3).

The endings of testa cells showed homogeneity within taxa in most cases. It was found that the dominant shape of seed coat cell endings (181 specimens) is rounded (e.g., *Polystachya kermesina* Kraenzl. –P. ker, *Polystachya fractiflexa* –P. fra and *P. woosnamii* Rendle –P. woo2) (**Figures 1E**, **2B, C**, **3A**). A rectangular-rounded shape was also recorded in two specimens: *P. affinis* (P. aff2) and *P. dendrobiiflora* (P. den3) (**Figure 1B**). This was an exception regarding shape uniformity within a given taxon in *P. affinis*, both rounded and rectangular-rounded endings occurred, while among six specimens of *P. dendrobiiflora*, one had rectangular-rounded testa cell endings (**Figure 1B**). Seed coat cells of *Polystachya* seeds showed no differences in the structure of the anticlinal wall. In all analysed seeds, the anticlinal wall of these cells was straight and did not exhibit any curvature (**Figures 1B, E**). The periclinal wall was invisible in most specimens (102), which results from the tight packing of the cells forming the anticlinal walls (e.g., in *P. elegans*, P. ele). However, it can be observed in 81 specimens (e.g., *P. concreta*, P. con39). In seeds where the periclinal wall was visible, it had a smooth surface (**Figures 1B**, **2B**, **3B**). Examples of such taxa include *P. dalzielii* Summerh. – P. dal, *P. undulata* P.J. Cribb & Podz. – P. und, and *P. villosa* – P. vil.

### 3.5 Variation in qualitative traits

**Figure 5:**
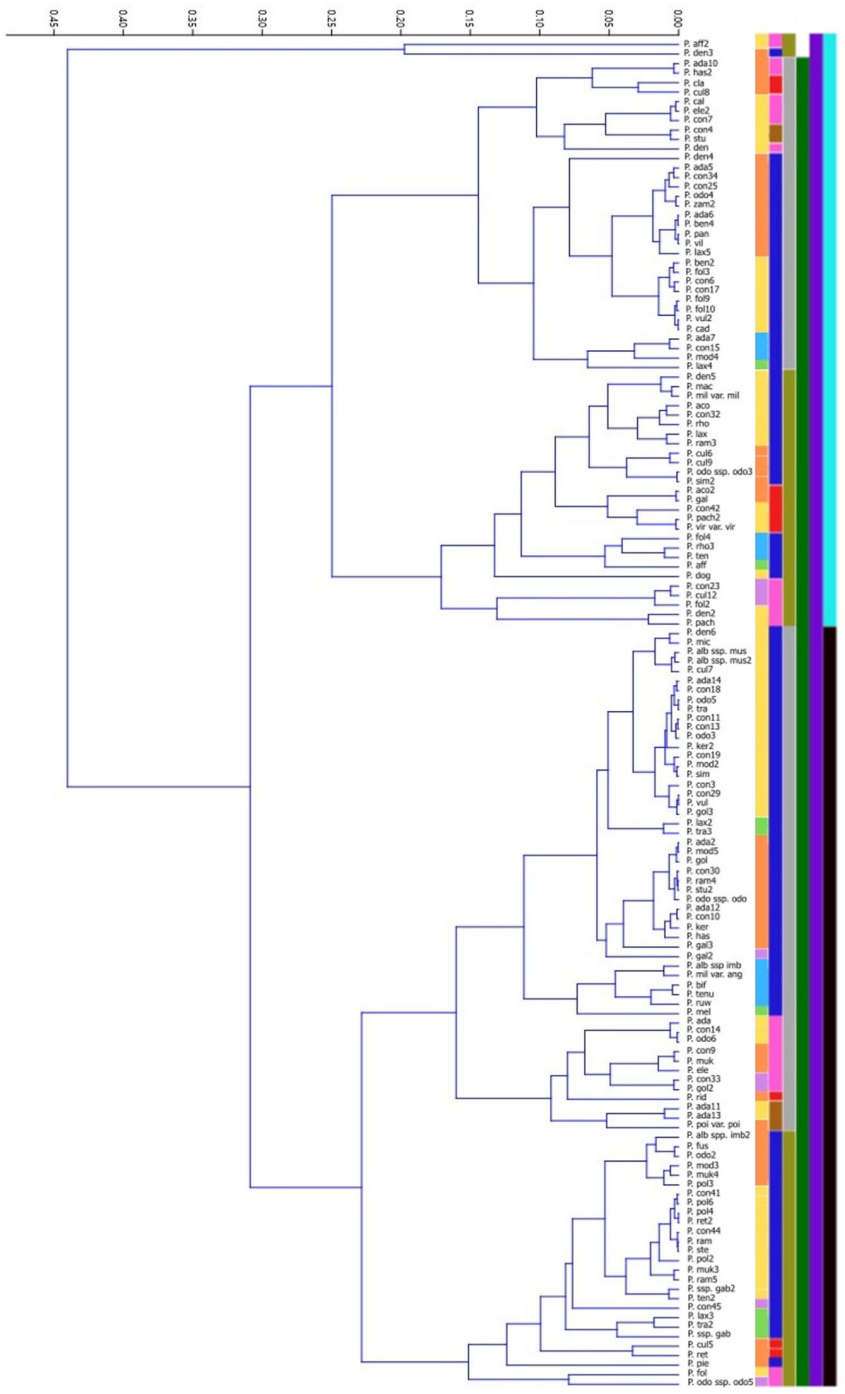
UPGMA cluster analysis for *Polystachya* and related genera based on the overall Gower’s coefficient (1971) for qualitative and quantitative traits. Explanation: seed shape (yellow, ellipsoid; orange, ellipsoid-ovoid; light blue, fusiform; light green, fusiform-ellipsoid; light purple, ovoid); shape of the seed apex (pink, rounded; dark blue, cuneate; red, cuneate-rounded; brown, rectangular); seed coat cells shape (olive green, elongated irregular; grey, elongated regular); apex of testa cells (dark green, rounded; white, rectangular-rounded); appearance of the anticlinal walls (dark purple, straight), visibility of the periclinal wall (turquoise, visible; black, not visible).

The dendrogram of the hierarchical cluster analysis based on Gower’s distance (**Figure 5**) reveals two major clusters. The first cluster consists of only two specimens,*Polystachya affinis* (P. aff2) and *P. dendrobiiflora* (P. den3) which separate from all other specimens at a relatively high distance level. Both share several diagnostic features, including elongated and irregular testa cells, rectangular-rounded cell endings, simple anticlinal walls, and visible periclinal walls. They differ, however, in seed shape (*P. affinis* has elliptical seeds, whereas *P. dendrobiiflora* has elliptical-oval seeds) and in the form of the seed apex (rounded in *P. affinis* and wedge-shaped in *P. dendrobiiflora*). Notably, among all analyzed specimens, only these two possess rectangular-rounded testa cell endings. The second major cluster is divided into two well-defined subgroups. The first subgroup primarily includes seeds with elliptical and elliptical-oval shapes, and specimens within this group also exhibit visible periclinal walls. The second subgroup, which contains, among others, *Polystachya concreta* (P. con19), *P. adansoniae* (P. ada11), and *P. foliosa* (P. fol) is much more diverse in terms of seed shape, although elliptical seeds remain predominant. This subgroup is further characterised by a prevalence of species with wedge-shaped seed apices and elongated, regular testa cells, such as *P. microbambusa* (P. mic), *P. transvaalensis* (P. tra), and *P. bifida* (P. bif). It is also evident that the numerous samples of *P. concreta* do not cluster into a single coherent group but are dispersed across different parts of the dendrogram.

**Figure 6:**
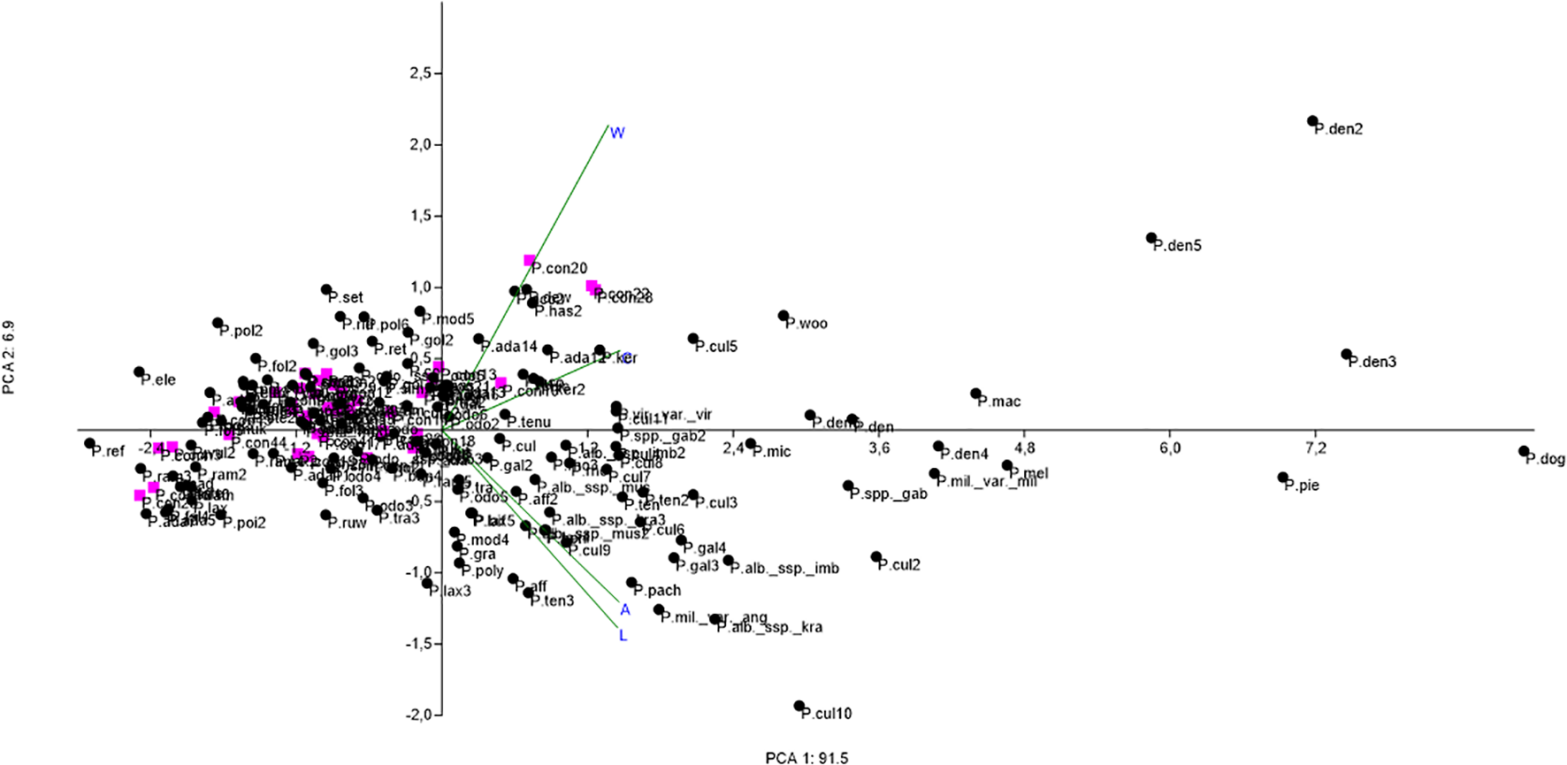
PCA ordination diagram of the studied *Polystachya* taxa based on biometric seed traits (abbreviations as in **Supplementary Table 1**).

Based on the principal component analysis (PCA), no clear differentiation among the examined samples was observed in the ordination space (**Figure 6**; **Supplementary Material 1**). In the PCA plot, the horizontal axis (PCA 1) explains 91.5% of the total variance, while the vertical axis (PCA 2) accounts for 6.9%. Most of the analyzed samples cluster in the central part of the plot, indicating that the measured seed metric traits do not substantially differentiate the individual taxa. On the right side of the ordination diagram, two small groups of points are separated from the main data cloud. The first of these groups, slightly shifted from the center, includes species such as *Polystachya dendrobiiflora* (P. den4), *P. cultriformis* (P. cul2), *P. melanantha* (P. mel), *P. maculata* P.J. Cribb (P. mac), *P. mildbraedii* var. *mildbraedii* (P. mil. var. mil), and *P. odorata* ssp. *gabonensis* (P. spp. gab). These species differ from those grouped in the center primarily with respect to seed length and width. The distribution of *P. concreta* seed measurements overlaps broadly with that of the other species included in our analysis. Therefore, *P. concreta* cannot be distinguished as a morphometrically distinct group based on the examined seed traits.

## 4 Discussion

### 4.1 Seed diversity in the genus *Polystachya*

In most orchids, seed length ranges from about 100-150 μm up to even 6000 μm, with a significant portion of species having seeds sized between 300-800 μm (Barthlott et al., 2014; Molvray and Kores, 1995). In the case of the genus *Polystachya* seed length ranged between 300-500 μm (Dressler, 1993; Barthlott et al., 2014). The results of the conducted analysis showed that seeds can reach sizes much smaller and larger than previously thought. The smallest average seed length recorded was 115.29 μm (*P. reflexa* – P. ref), and the largest was 609.39 μm (*P. doggettii* – P. dog). The minimum value from all measurements was about 180 μm smaller, and the maximum was about 160 μm larger than that given in the literature. The obtained results also contradicted previous data regarding seed length of previously studied *Polystachya* species. Ochora (2001) obtained for *P. adansoniae* an average seed length of 258.8 μm and width of 87.9 μm for four seeds analyzed. In our studies, the arithmetic mean of length and width of seeds for ten specimens of this species was 209.3 μm and 61.2 μm, respectively. Lallana et al. (2019) reported an average seed length of 289.5 μm and width of 81.8 μm for *P. concreta*. This study showed that these values calculated for 39 specimens were 194.5 μm and 61.7 μm.

Previous studies have shown that the appearance of the periclinal wall can have significant value in the systematics of orchids (Barthlott, 1976; Ortunez et al., 2006). Among the analyzed *Polystachya* species, most were characterized by an invisible periclinal walls. This feature allows one to distinguish the genus from others within the subtribe Polystachyinae, such as *Hederorkis* Thou. or *Neobenthamia* Rolfe (Barthlott et al., 2014). However, the literature shows that this is not the only genus within Orchidaceae exhibiting an invisible periclinal wall. It is a typical trait for subtribes of the tribe Vandeae, to which the subtribe Polystachyinae also belongs. Other examples of subtribes are Aeridinae, Adrorhizinae, and Angraecinae (Barthlott et al., 2014; Gamarra et al., 2025; Ortúñez and Gamarra, 2023). Gamarra et al. (2025), analyzing seeds from the subtribe Angraecinae, demonstrated that the periclinal walls in 38 studied genera are sunken and barely visible, similarly to the observations of Ortúñez and Gamarra (2023) for the genus *Bromheadia* Lindl. belonging to the subtribe Adrorhizinae. Additionally, Gamarra et al. (2012) noted that narrow or invisible periclinal walls are common features among epiphytic orchids belonging to African Vandeae. However, this trait may have value in distinguishing it from other tribes and subtribes where the periclinal wall is visible, for example Chloraeeae, Cypripedieae, Orchideae, or Maxillariinae (Chemisquy et al., 2009; Gajda, 2024; Gamarra et al., 2018; Hariyanto, 2019). In cases where the periclinal wall was visible, it was characterized by a smooth structure, e.g. *P. dendrobiiflora* (P. den4) or *P. fractiflexa* (P. fra). Nonetheless, the absence of ornamentation can also be noticed among some genera belonging to the subtribes Dendrobiinae, Orchidinae, or Paphiopedilinae (Gamarra et al., 2018; Hariyanto, 2019; Rewicz et al., 2022). A smooth surface of the periclinal wall can, however, serve as a distinguishing feature from groups of orchids characterized by various types of ornamentation. For example, in studies on species of the tribe Chloraeeae, *Chloraea membranacea* Lindl. had disorganised, thin ridges on the periclinal cell wall (Chemisquy et al., 2009). Ornamentation can also be found in the genera *Camaridium* Lindl.*, Habenaria* Willd., and *Neotinea* Rchb.f., which have ring-like structures and thin, oblique ridges, respectively (Gajda, 2024; Gamarra et al., 2007; Rewicz et al., 2022). Considering the above analyses concerning *Polystachya* and other tribes and subtribes, it can be concluded that the periclinal wall is not a useful trait in taxonomy at the subtribe level and above. However, in taxonomic considerations, the structure of the periclinal wall can be successfully used at lower taxonomic levels and in combination with additional traits, such as seed size and the shape and arrangement of seed coat cells (Rewicz et al., 2022).

The analysis of qualitative traits showed that seed shape remains relatively constant only in a few cases within *Polystachya*. An attempt was made to verify the obtained information concerning the shape against available literature data. This turned out to be relatively problematic due to the poor knowledge of seeds from the genus. According to Ochora (2001), seed shape ranges from ellipsoid to ovoid for species: *P. adansoniae, P. bennettiana, P. cultriformis, P. dendrobiiflora, P. fusiformis, P. transvaalensis*. However, this work does not contain a list of species with assigned shapes but often generalizations for groups that formed one cluster or information for selected species. Ochora (2001) noted, however, that sometimes more than one seed shape occurs within the analyzed taxa. In our studies, the shape varied both between species and analyzed specimens. Within samples of one species, both ellipsoid, ellipsoid-ovoid, and fusiform-ellipsoid shapes could be distinguished. In the case of *P. transvaalensis*, both the ellipsoid shape, as noted by Ochora (2001), and fusiform-ellipsoid shape appeared. Comparative value for our results may also be provided by studies of Gamarra et al. (2018) and Lallana et al. (2019) concerning *P. fusiformis* and *P. concreta*, respectively. For *P. concreta*, seed shape was unified as oval or spherical. Our studies showed high variability within *P. concreta* specimens. Besides ovoid, they also had ellipsoid, ellipsoid-ovoid, and fusiform shapes. Also, the apex shape can vary from cuneate, cuneate-rounded, rounded to rectangular. Then Gamarra et al. (2018) described the seed shape of *P. fusiformis* as fusiform. However, for one sample of this species analyzed in our work, the shape was ellipsoid-ovoid. Therefore, as seen from various examples, the available literature data did not indicate a clear seed shape characteristic for a genus or even a species. The fact that various terminologies exist to describe seed shape, and often its incorrect interpretation by various authors, was emphasized by Barthlott et al. (2014) and because of this, these authors state that seed shape has little comparative value. However, there are also studies in the literature indicating that seed shape is important in classification and systematics due to its low variability (Arditti et al., 1979; Vij et al., 1992). Our results suggest that seed shape does not show stability at the species level, which limits its value as a taxonomically significant trait. Kurzweil (1993) shares a similar view, stating that seed shape is highly variable and has no taxonomic significance. However, it is worth noting that the genus *Polystachya* is very diverse, and in our opinion, this trait may have higher diagnostic value within other unstudied taxa.

In most of the samples studied, the shapes of the testa cells were elongated and regular or elongated and irregular in shape, and the number of cells along the axis of the test varied (from many to few). Interestingly, it often happened that the variation in these characteristics was observed among specimens of particular species. Examples included *Polystachya concreta* and *P. foliosa*, in which both varied in shape and number can be distinguished. The lack of taxonomic value of the shape and number of seed coat cells was also confirmed by the research of Rewicz et al. (2022). Analyzing seeds of the genus *Habenaria*, they found that the arrangement and shape of the cells do not allow for an unambiguous distinction between the taxa studied. In this study, as in the case of Polystachyinae, variation in the number of testa cells (many and few) was observed. However, these results contradicted the literature on the subject. Previous studies have indicated that among orchids, characteristics such as the number and shape of individual seed coat cell have high taxonomic value and may be helpful in differentiating between different taxonomic levels (Arditti et al., 1979; Molvray and Kores, 1995; Ramudu et al., 2020; Swamy et al., 2004). Variation in the number of testa cells among genera was also confirmed by Barthlott et al. (2014).

Finally, our results showed that the anticlinal wall is always straight and free of any additional structures. In the literature, the anticlinal wall of the genus was described as straight and additionally characterised as elevated and bearing broad marginal ridges (Barthlott et al., 2014). Ochora (2001) additionally stated that the transverse anticlinal walls in *Polystachya* are arched and occur as structures raised above the seed surface, except for *P. dendrobiiflora*, where they are flat. Furthermore, the longitudinal anticlinal wall, besides a straight form, may also take an irregularly elongated shape. Gamarra et al. (2018) described the longitudinal anticlinal wall as thickened with distinct ridges, and the transverse wall as curved. The stability of the trait was observed in our study, which means that there were no additional structures observed. However, a straight anticlinal wall without additional structures was also found by Gajda (2024) in the subtribe Maxillariinae, which calls into question the diagnostic value of this trait at the subtribe level. *Polystachya* seed can be distinguished from taxa whose anticlinal wall is characterized by additional structures, e.g. in subtribes Oncidiinae and Angraecinae. Some species of these groups had elongations present on the anticlinal walls (Chase and Pippen, 1988; Gamarra et al., 2025). It may be nodular endings throughout the seed, as in *Oncidium variegatum* (Sw.) Sw., or hook-shaped structures only at the seed poles, e.g., *Psygmorchis pusilla* (L.) Dodson & Dressler (Chase and Pippen, 1988). Gamarra et al. (2025) also noted variation of these elongations among Angraecinae species depending on the pole. Some had hair-like/trichome structures on the basal pole, e.g. *Dendrophylax porrectus* (Rchb.f.) Carlsward & Whitten, while others had hook-like extensions on the apical pole, e.g. *Solenangis scandens* (Schltr.) Schltr.

### 4.2 Implication in the systematic of the genus

The current taxonomy of the genus *Polystachya* has been the subject of numerous discussions and studies over the years (Kraenzlin, 1926; Summerhayes, 1947; Podzorski and Cribb, 1979; Stévart and Nguema, 2004; Russell et al., 2010, 2011, Mytnik-Ejsmont, 2011; de Abreu et al., 2018). The division of the genus into sections has also remained problematic. Initially, according to Kraenzlin (1926) classification, 12 sections were distinguished. Later, with the discovery of new species and new classifications published by Cribb (1978) and Summerhayes (1942, 1947), the number of sections increased to 15, and now, according to the latest division by Mytnik-Ejsmont (2011), it has decreased to 13. However, Mytnik-Ejsmont (2011) suggests distinguishing from *Polystachya s.l.* a few smaller genera, usually classified in the sectional level.

Our study included only a limited number of *Polystachya* species that were classified by Mytnik-Ejsmont (2011) within the newly proposed genera, such as *Chelystachya*, *Dendrobianthe*, *Epiphorella*, and *Unguiculabia*.

No carpological analyses of representatives of these genera have been recorded in the literature. All analyzed samples showed to be diverse and heterogeneous like within the rest of *Polystachya*. These differences were noticeable also within seed apices. Results indicated that for *P. dendrobiiflora* (=*Dendrobianthe dendrobiiflora* (Rchb.f.) Mytnik), both rounded and wedge-shaped seed apices may occur. Similarly, species classified under genus *Unguiculabia* (Mytnik-Ejsmont, 2011), two seed apex shapes can be distinguished: elliptical (*P. caduca* – P. cad) and elliptical-oval (*P. elegans –* P. ele). Analyzing seed lengths, it can be noted that their variation, in some cases, can serve as a basis for effectively distinguishing these taxa. The samples that clearly standed out from the others were those classified within *Dendrobianthe sensu* Mytnik-Ejsmont (2011) and had the longest seeds. The arithmetic mean for six taxa was 421.3 μm, whereas for 177 taxa of *Polystachya s.s.* it was 232.7 μm.

Numerous studies, such as those by Barthlott (1984) and Karcz and Tomczok (1987), confirm that seed surface and anatomical features can provide valuable information on taxonomy and phylogenetic relationships of many plant groups, as they are less influenced by environmental factors. It has also been shown that seed micromorphology is a valuable source of systematic traits that can be used to identify subgeneric groups and formulate hypotheses about relationships between species within a genus (Larry, 1995; Matthews and Levins, 1986). Analyses of orchid seed morphology confirming their taxonomic usefulness have been carried out by Barthlott et al. (2014); Ramudu et al. (2020), and Rewicz et al. (2022). Our study confirm that *Polystachya* seeds have limited potential to be a valuable tool in species identification, however more extensive sampling is needed to confirm this.

### 4.3 Limitations of this study and future research

The results of this study emphasize the importance of both quantitative and qualitative seed traits from the genus *Polystachya*, which can be successfully used to support taxonomic revision at various levels. The trait with the greatest taxonomic potential for representatives of the genus appears to be the absence of visibility or, in some cases, the smooth structure of the periclinal wall. However, the next study should be expanded to include additional samples that better represent the remaining sections of the genus, as well as groups of species that are endemics, such as *Polystachya* occurring in Madagascar or in Asia. Furthermore, it would be valuable to increase the species that represent genera as outlined in Mytnik-Ejsmont (2011), such as *Chelystachya*, *Dendrobianthe*, *Epiphorella*, and *Unguiculabia* (included in this study), as well as the genera not represented here: *Neoburttia* Mytnik, Szlach. & Baranow, *Isochilostachya* Mytnik & Szlach., *Disperanthoceros* Mytnik & Szlach., and *Szlachetkoella* Mytnik. Especially in the light of preliminary evidence suggesting significant differences in seed structure among the representatives of these genera.

As much as possible, future studies should be expanded to include both new herbarium material and fresh material obtained from seed collections in the field, since herbarium specimens often were unsuitable for any analysis. Equally important for the taxonomy of *Polystachya* seems to be the issue of the embryo, specifically its measurement and volume calculation, which can also help determine the value of the air chamber. Future work should consider other methods of embryo visualization, such as the FAST9 method with 60% iohexol, which according to the authors, may assist in imaging plant embryos (Attuluri et al., 2022). The results obtained could represent a significant differentiating feature for both genera and species within this subtribe Polystachyinae.

Any observed differences within the analyzed specimens of individual species may be related to the different seed collection times, their degree of maturity, and the diversity of ecological conditions in the areas from which the samples originate. Therefore, it is advisable to extend analyses to include climatic, soil, moisture, and physiological factors, which will allow for a better understanding of orchid seed biology and enable more precise taxonomic classification. This would be especially significant in the case of *Polystachya concreta*. It is worth recalling that the analyzed taxa, through hierarchical cluster analysis, did not form one clear group against other samples but were dispersed across different clusters. Such a distribution suggests a lack of unequivocal morphometric distinctness of this taxon compared to the other studied species. This may result from the fact that as the only pantropical species, *P. concreta* determines the pantropical range of the entire nominal section *Polystachya*, which may influence the close similarity of traits with other members of this section (Lok et al., 2011; Mytnik-Ejsmont, 2011). *Polystachya concreta* is an extremely variable, widely distributed species occupying different habitats, which may also affect the lack of conservativeness of morphometric traits within this species. Therefore, the introduction of ecological analyses could help resolve this issue. As noted by Jeeja and Ansari (1994), although seed morphological traits alone do not provide much information regarding tribal classification and genus relationships, a clear pattern in orchid seed morphology is now being recognized. If this pattern is thoroughly analyzed and combined with other traits, such as ecology, it may prove helpful in establishing relationships among taxa and thus contribute to improving the existing classification.

Finally, additional analyses should be conducted on other subtribes of the tribe Vandeae. At this stage, the greatest gap remains with the numerous and highly diverse subtribe Aeridinae. Filling this gap and incorporating the results presented here, together with data from the subtribe Angraecinae, will make it possible to understand the diversity of seed morphology within the tribe and its taxonomic significance.

## 5 Conclusion

The examination of seed morphology in *Polystachya* demonstrates that several seed characters, particularly dimensions and the architecture of testa cells, exhibit meaningful variation that can support taxonomic interpretation. Although some characters are widely shared across species, others display patterns that align with natural groupings within the genus and therefore hold diagnostic value. These findings show that seed morphology enriches the available evidence for understanding species boundaries and evolutionary relationships in *Polystachya*.

Incorporating these characters into broader systematic studies may contribute to more robust and better-resolved taxonomic frameworks for the genus.

## Data Availability

The original contributions presented in the study are included in the article/**Supplementary Material**. Further inquiries can be directed to the corresponding author.
