# Supplementary material for: Application of seed micromorphology in taxonomy of the genus Polystachya Hook. (Vandeae, Orchidaceae)
Source: Front Plant Sci. 2026 May 18;17:1761768. doi: 10.3389/fpls.2026.1761768 (PMC13223086; doi:10.3389/fpls.2026.1761768)
Supplement: Supplementary file 1 [file DataSheet1.pdf]

Supplementary Figures

Scanning Electron Microscopy photographs of studies samples.

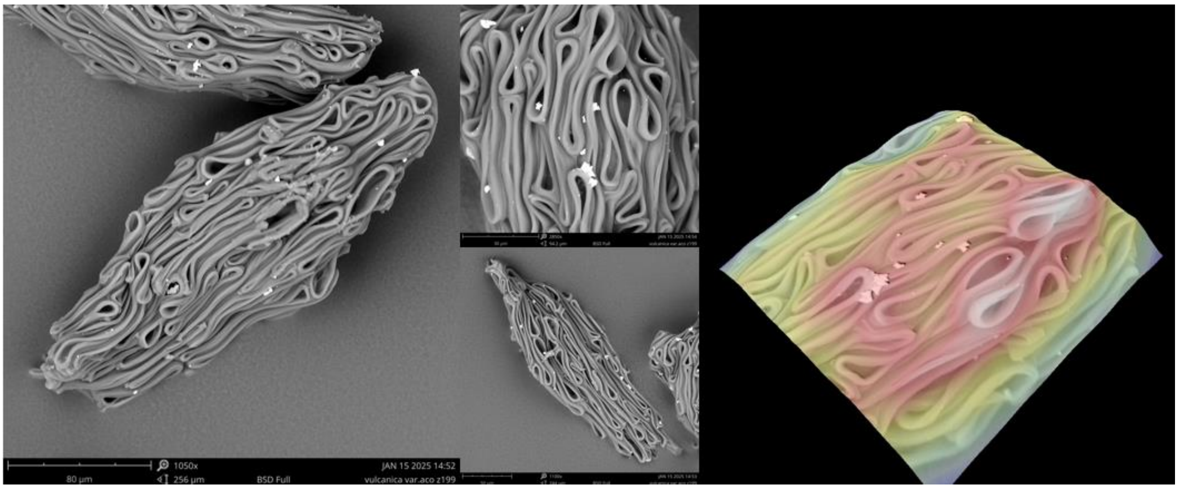

Fig.S1. *Polystachya aconitiflora* Summerh. (P. aco)

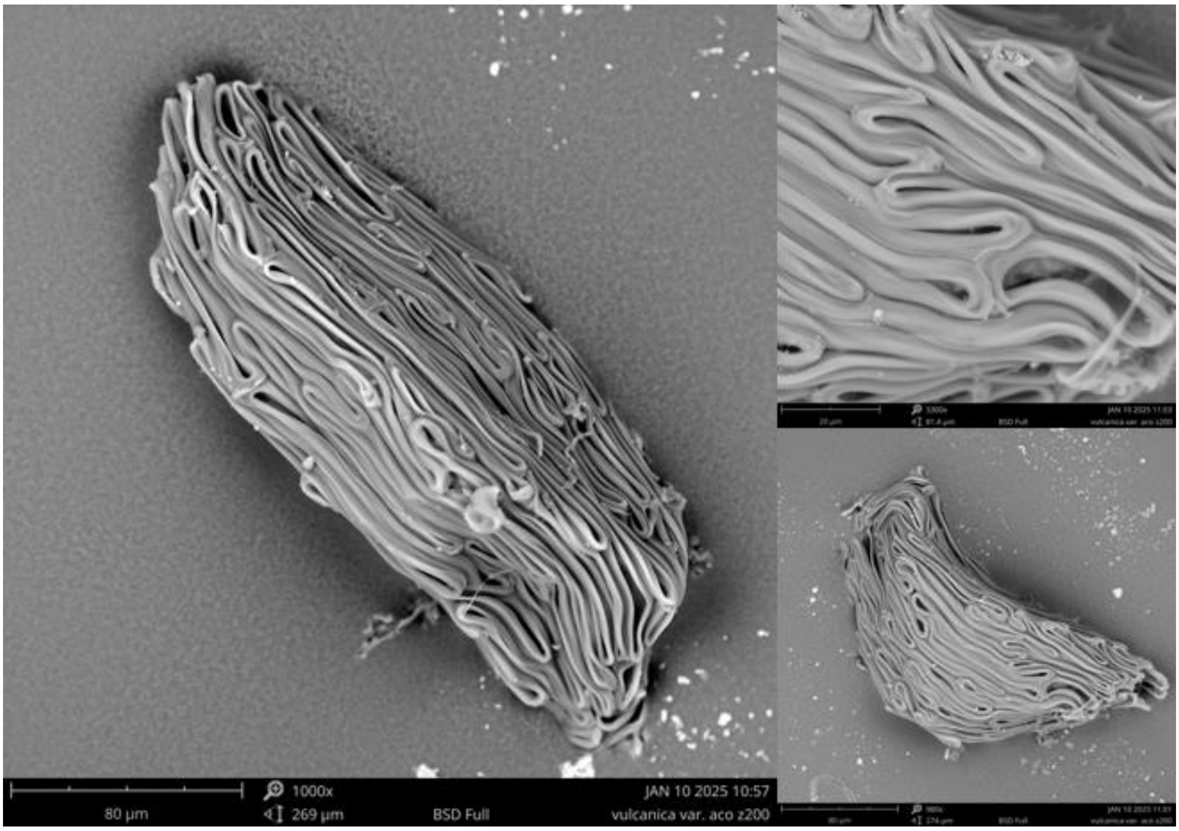

Fig.S2. *Polystachya aconitiflora* Summerh. (P. aco2)

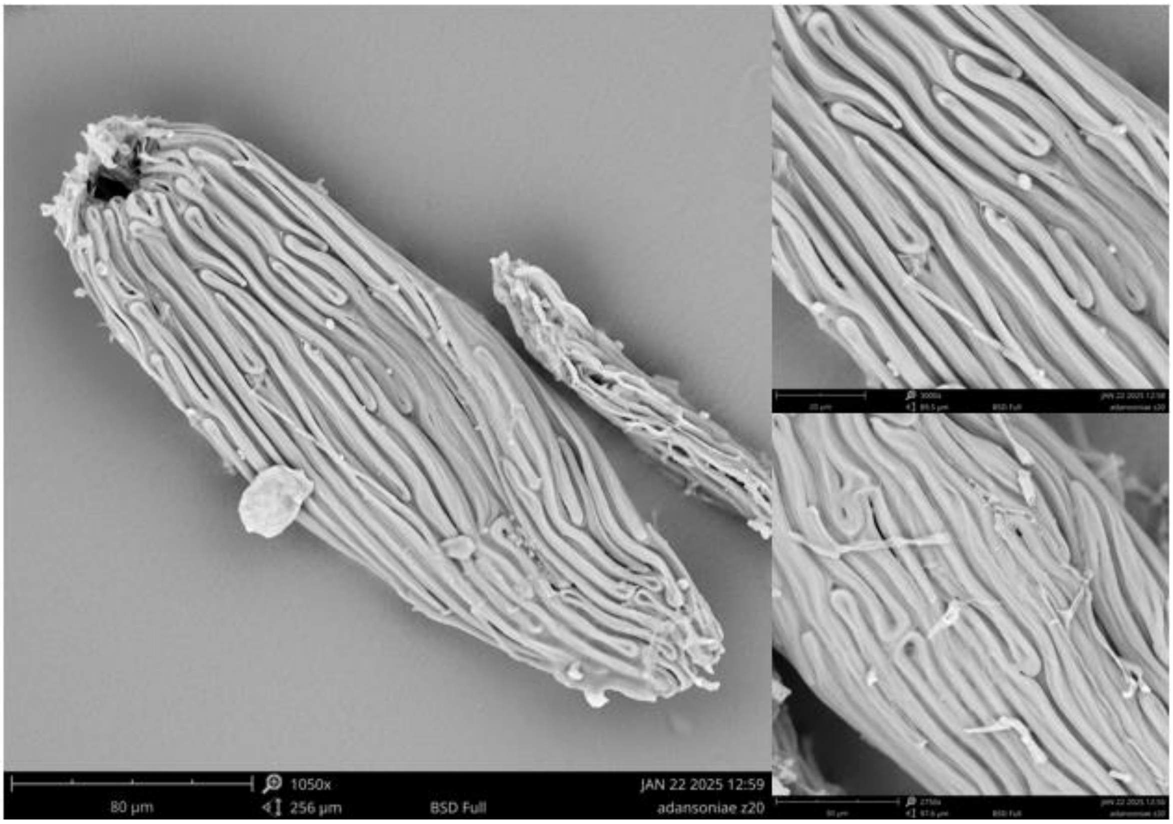

Fig.S3. *Polystachya adansoniae* Rchb.f. (P. ada)

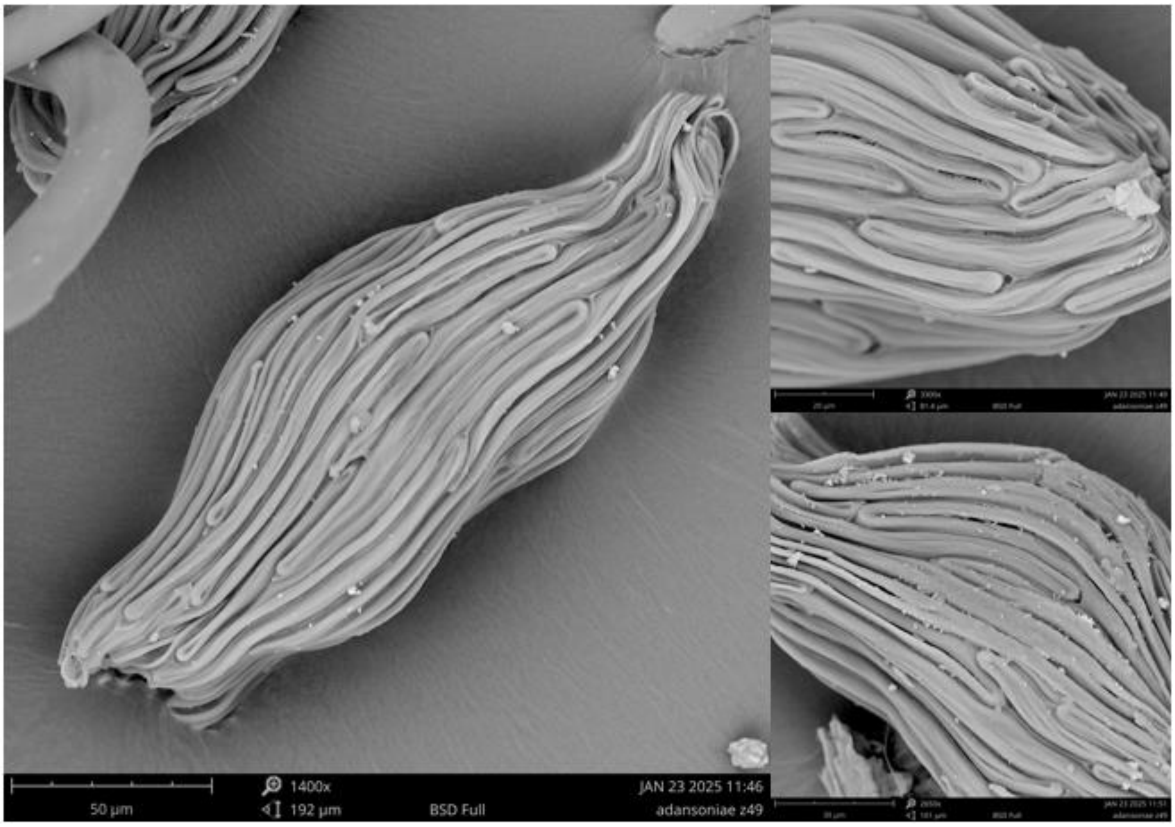

Fig.S4. *Polystachya adansoniae* Rchb.f. (P. ada2)

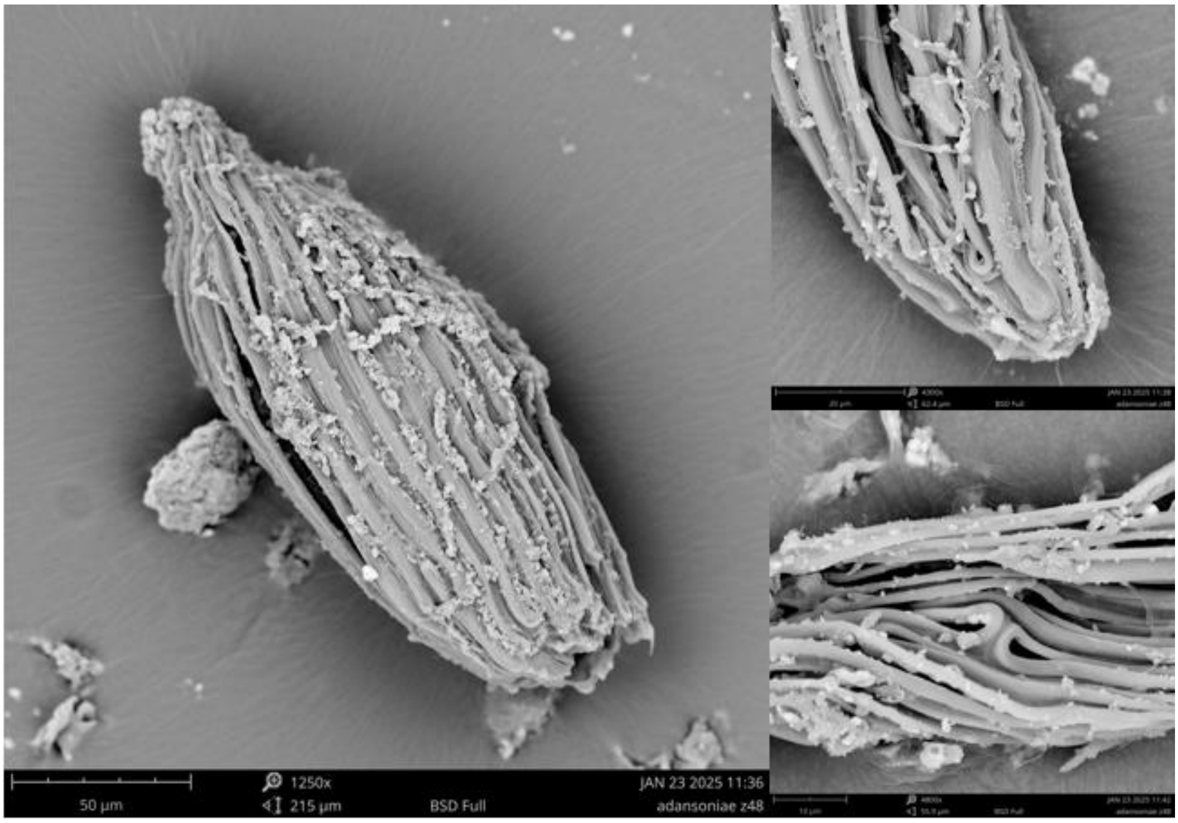

Fig.S5. *Polystachya adansoniae* Rchb.f. (P. ada3)

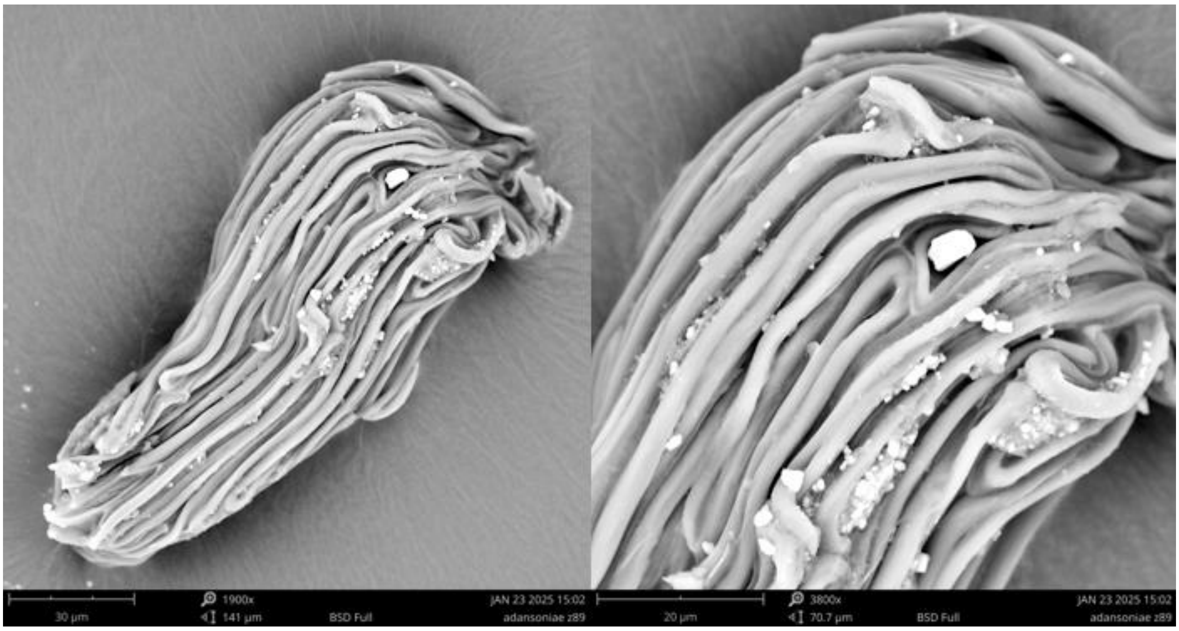

Fig.S6. *Polystachya adansoniae* Rchb.f. (P. ada4)

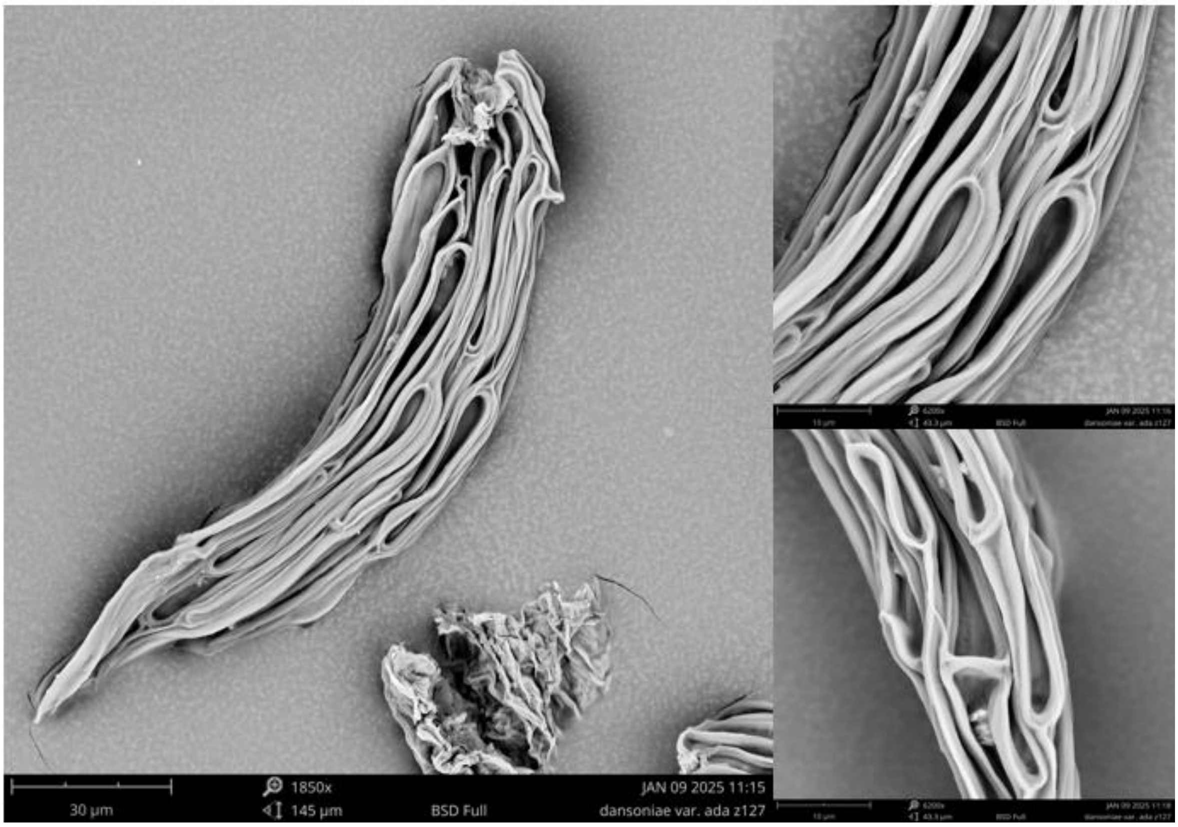

Fig.S7. *Polystachya adansoniae* Rchb.f. (P. ada5)

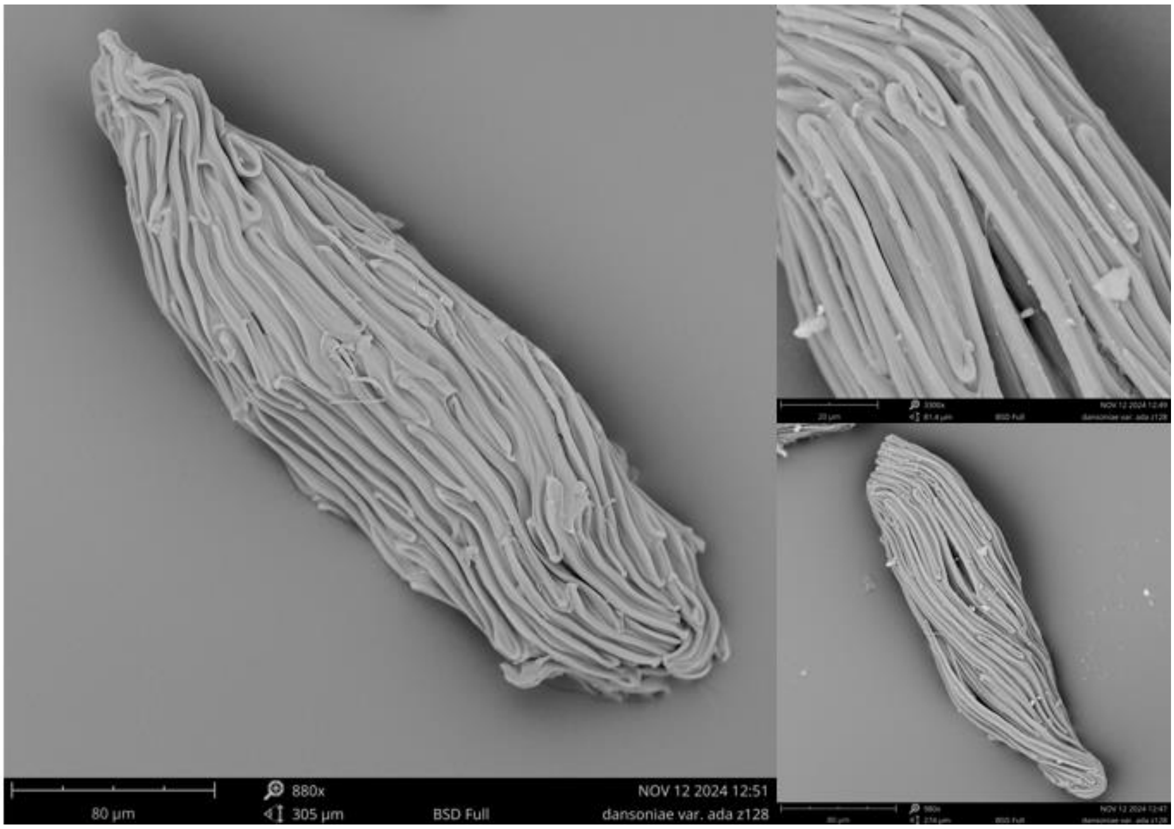

Fig.S8. *Polystachya adansoniae* Rchb.f. (P. ada6)

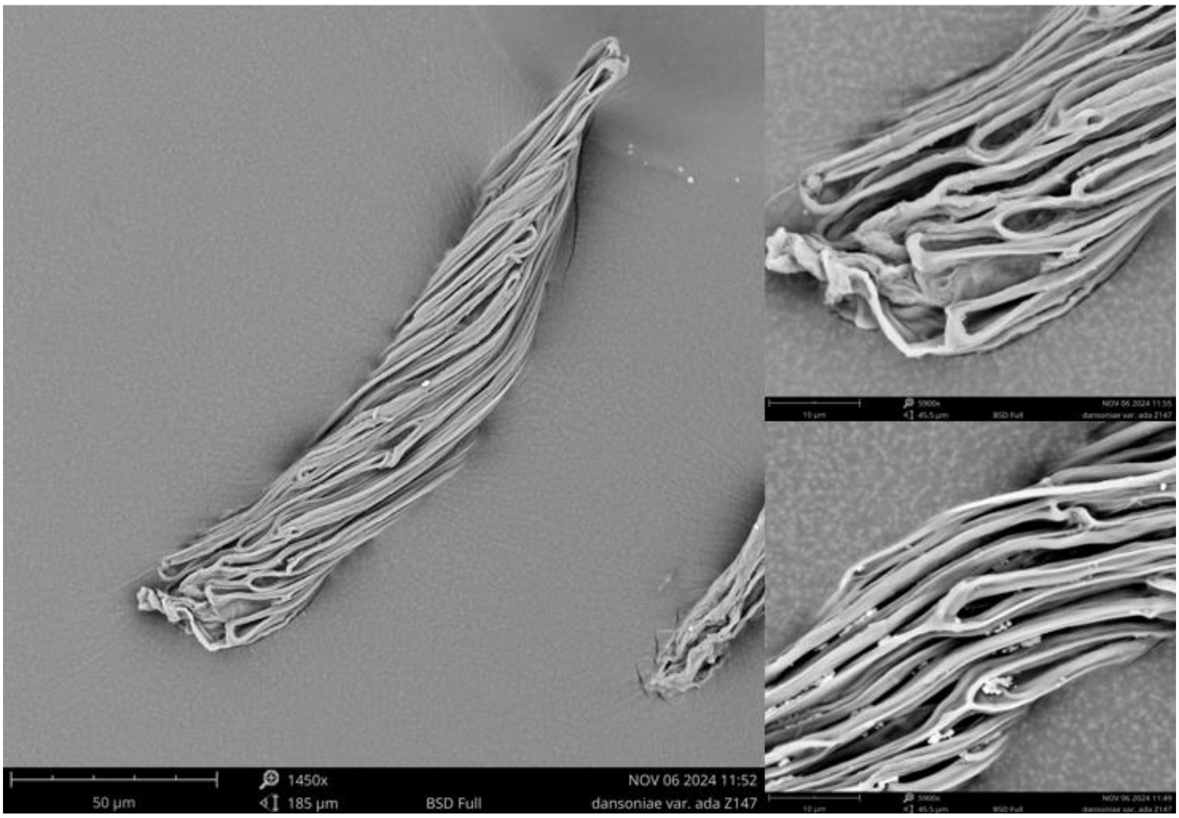

Fig.S9. *Polystachya adansoniae* Rchb.f. (P. ada7)

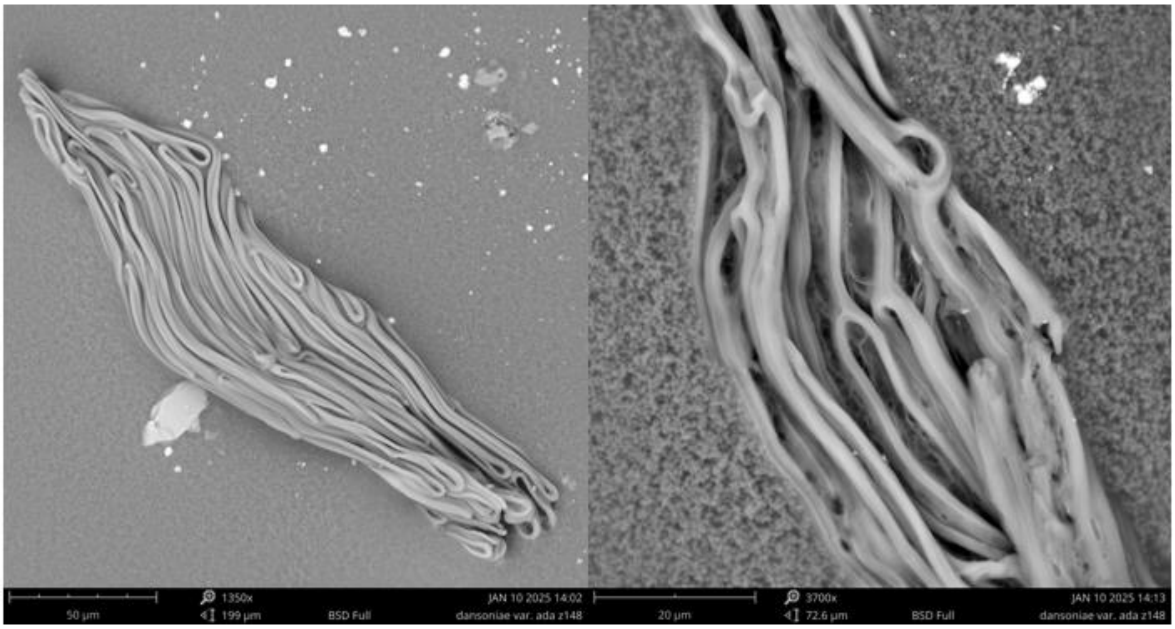

Fig.S10. *Polystachya adansoniae* Rchb.f. (P. ada8)

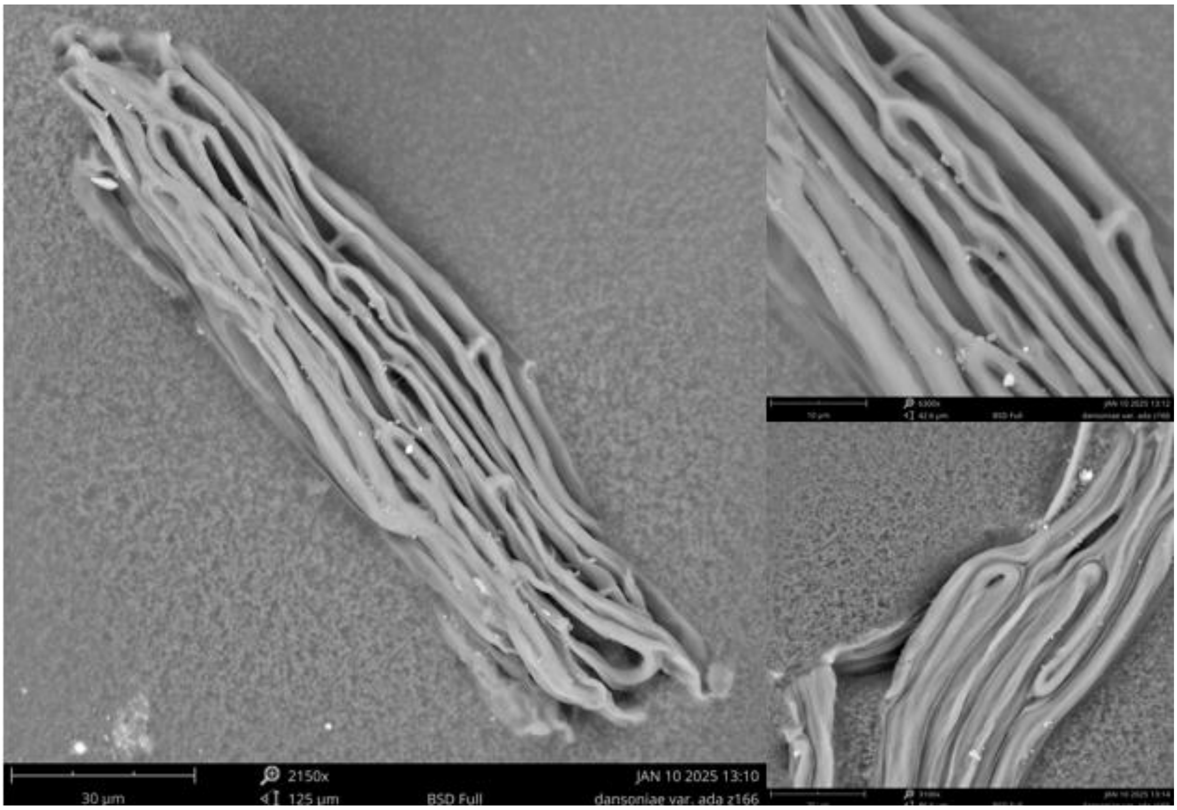

Fig.S11. *Polystachya adansoniae* Rchb.f. (P. ada9)

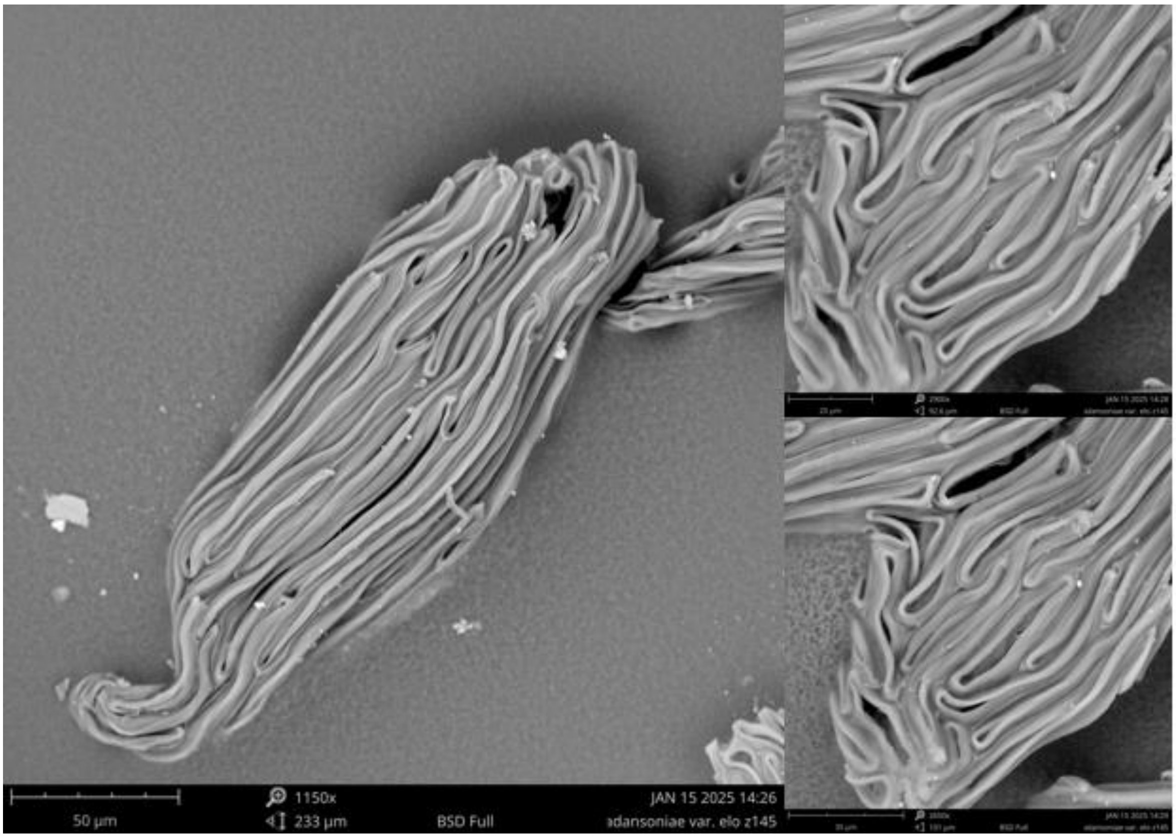

Fig.S12. *Polystachya adansoniae* Rchb.f. (P. ada10)

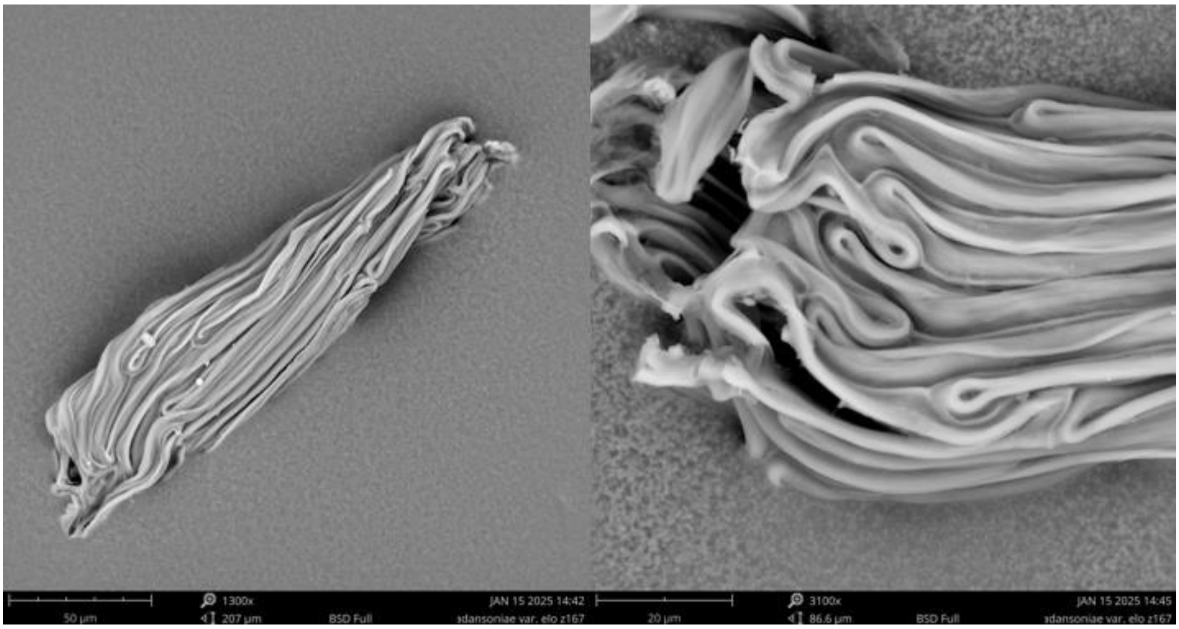

Fig.S13. *Polystachya adansoniae* Rchb.f. (P. ada11)

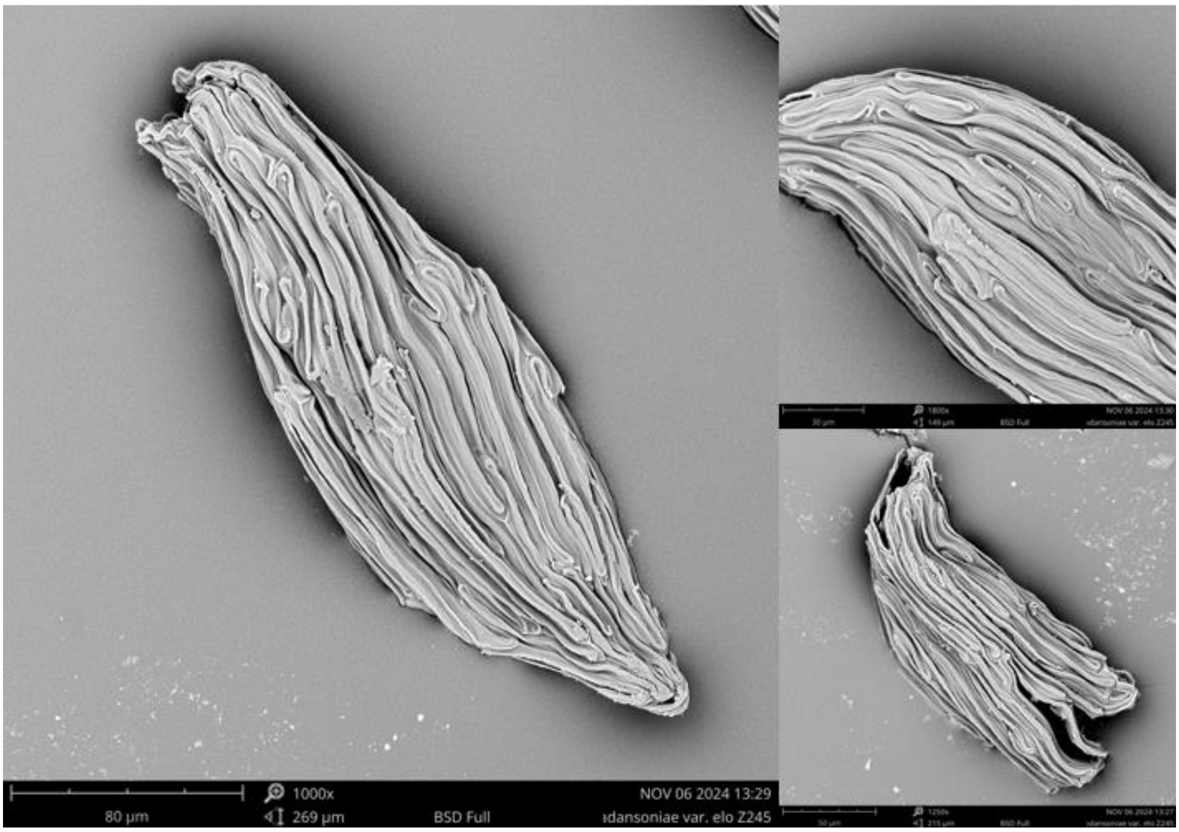

Fig.S14. *Polystachya adansoniae* Rchb.f. (P. ada12)

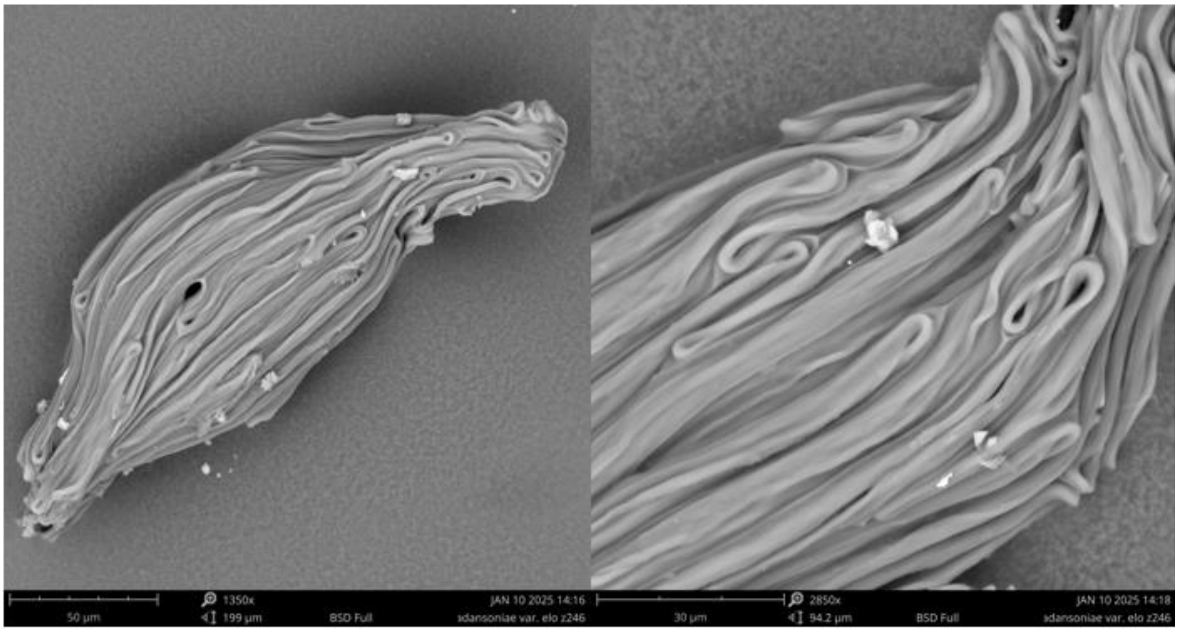

Fig.S15. *Polystachya adansoniae* Rchb.f. (P. ada13)

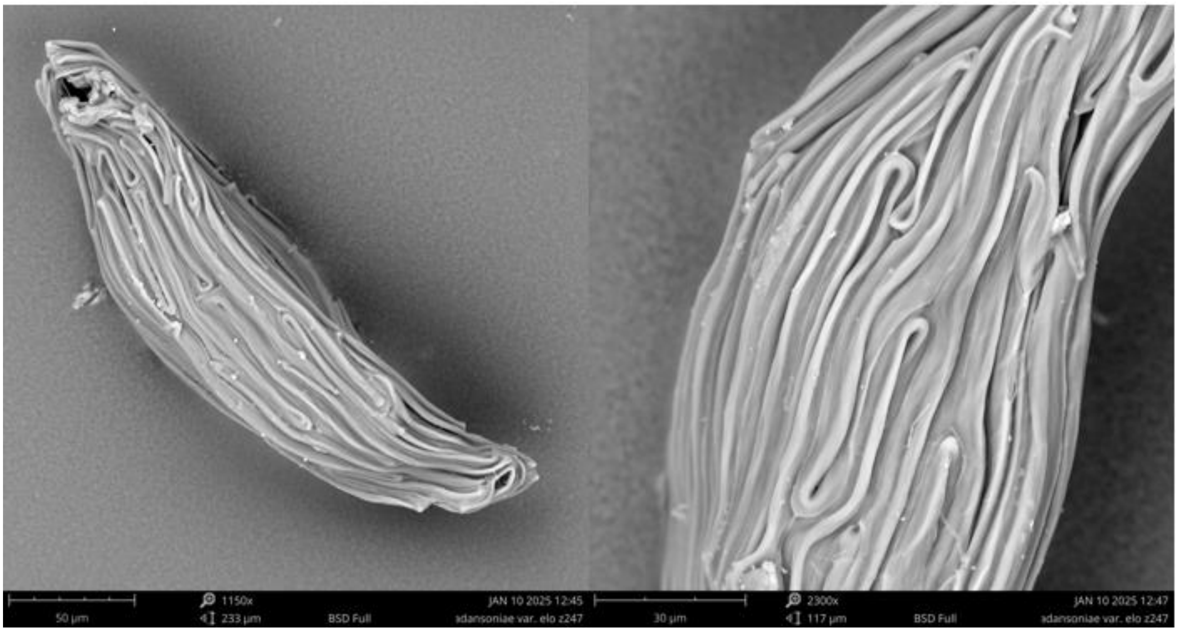

Fig.S16. *Polystachya adansoniae* Rchb.f. (P. ada14)

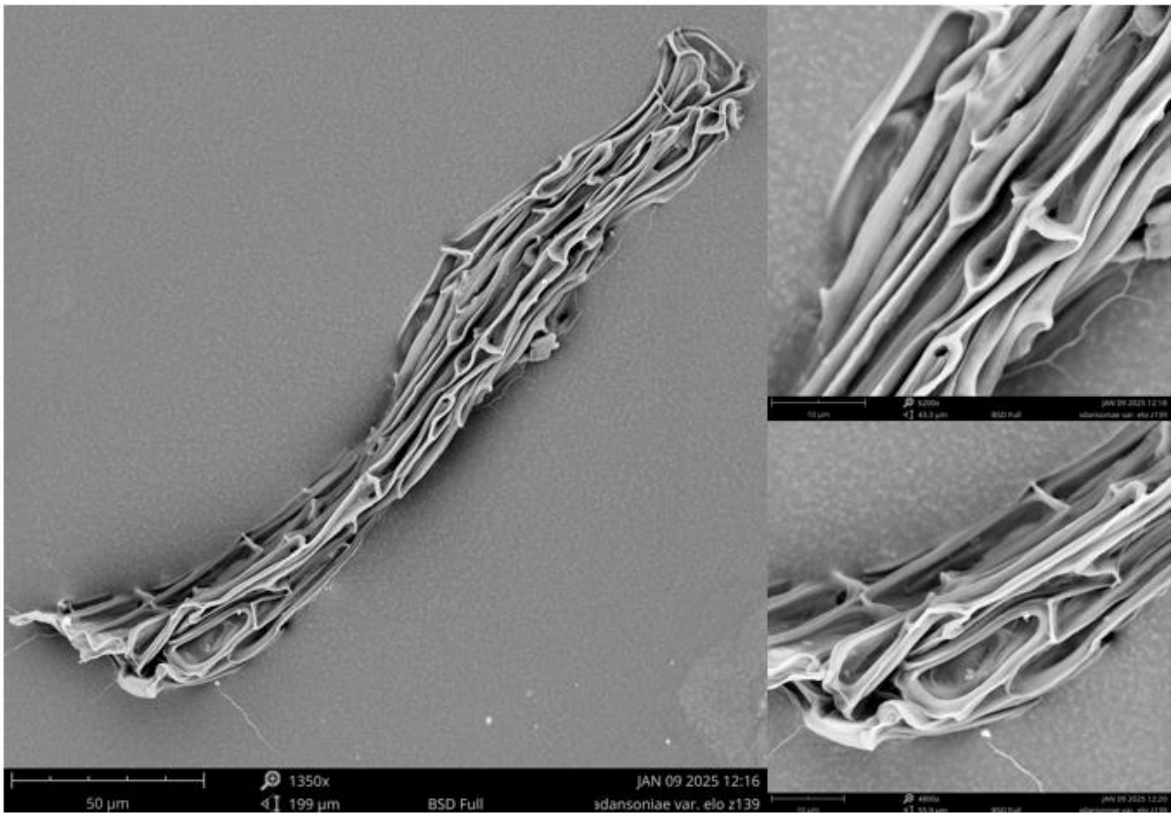

Fig.S17. *Polystachya adansoniae* Rchb.f. (P. ada15)

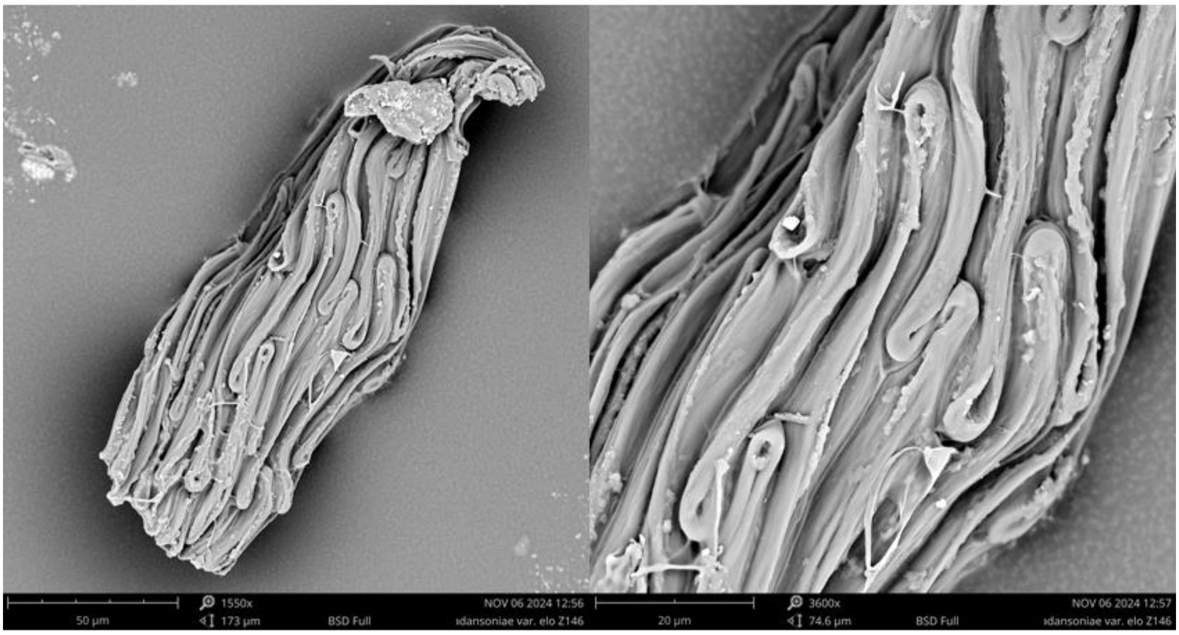

Fig.S18. *Polystachya adansoniae* Rchb.f. (P. ada16)

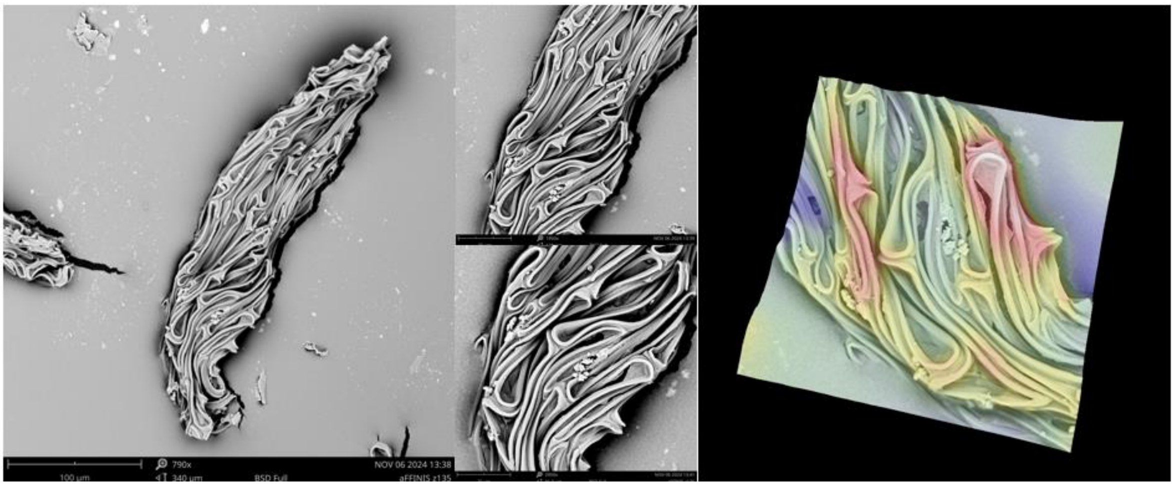

Fig.S19. *Polystachya affinis* Lindl. (P. aff)

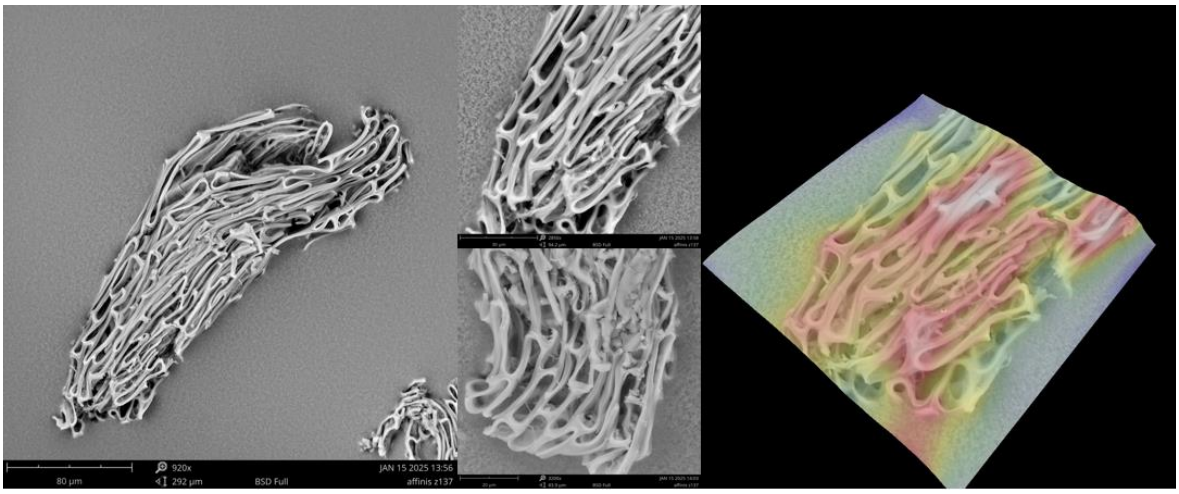

Fig.S20. *Polystachya affinis* Lindl. (P. aff2)

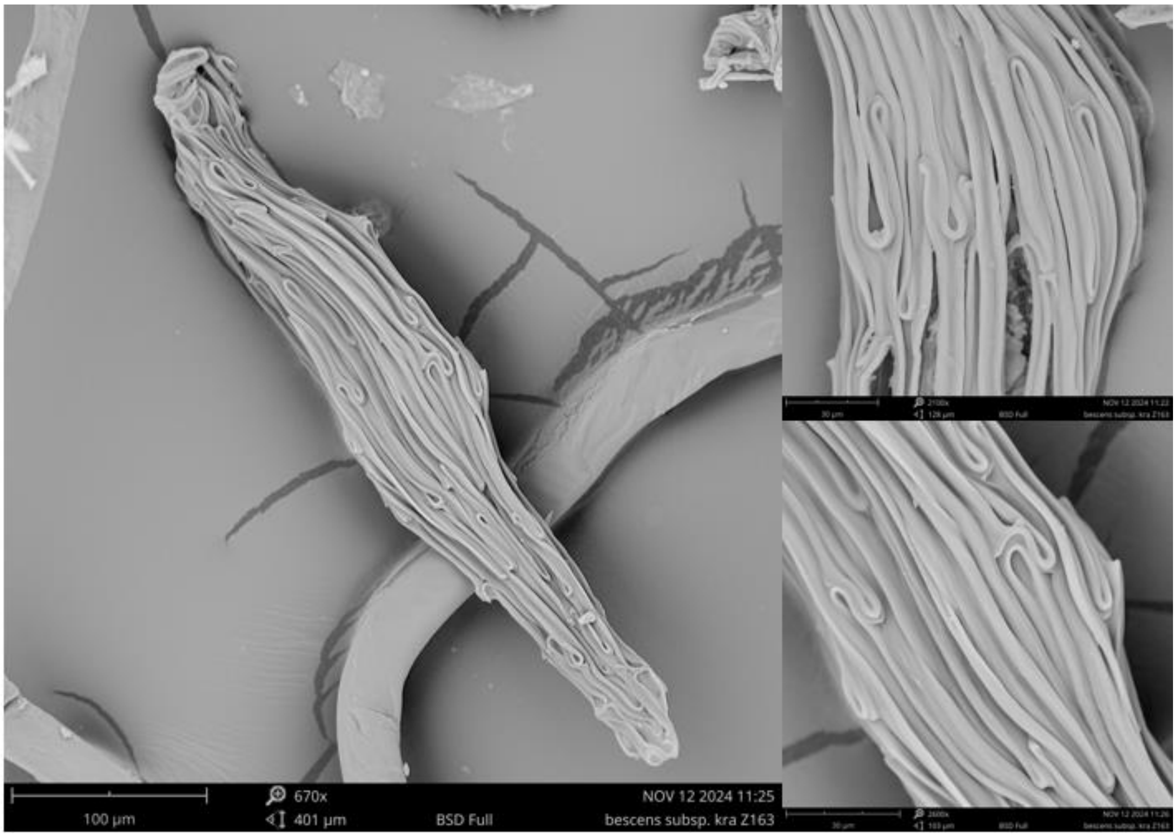

Fig.S21. *Polystachya albescens* ssp. *imbricata* Rolfe (P. alb. ssp. imb)

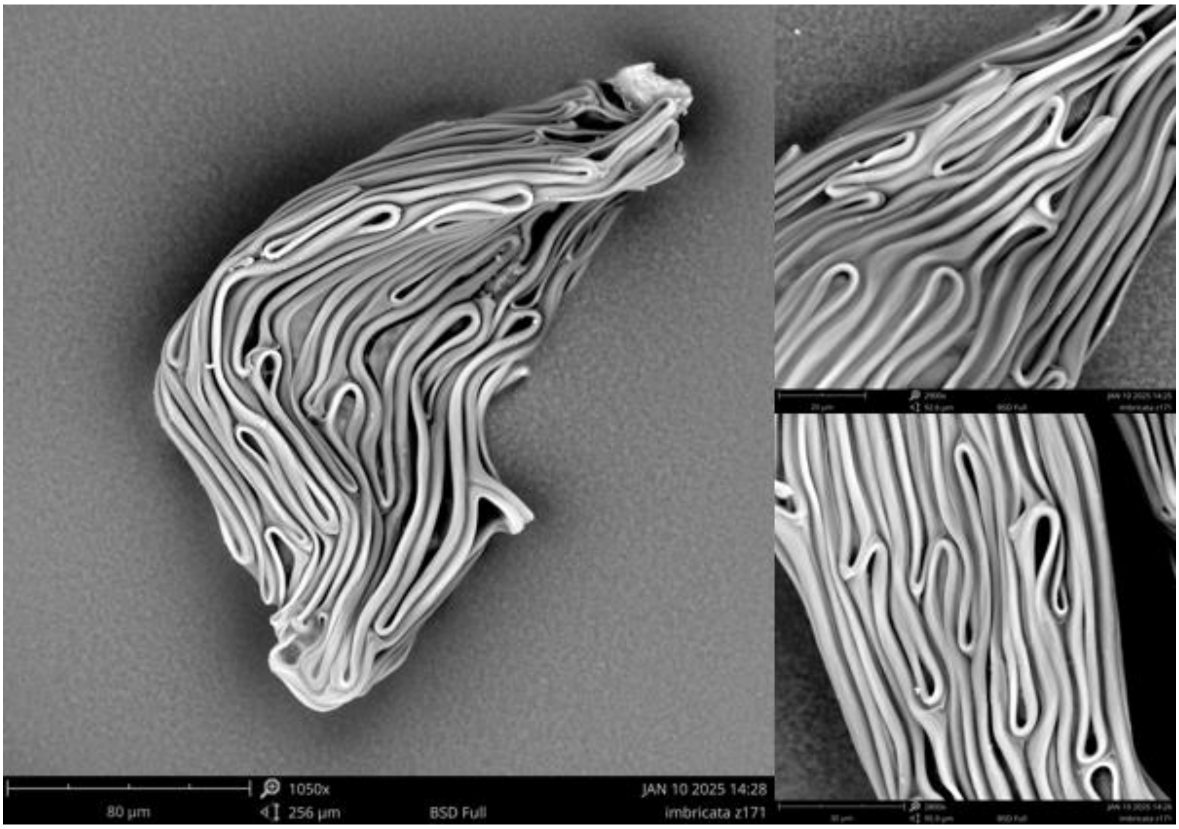

Fig.S22. *Polystachya albescens* ssp. *imbricata* Rolfe (P. alb. ssp. imb2)

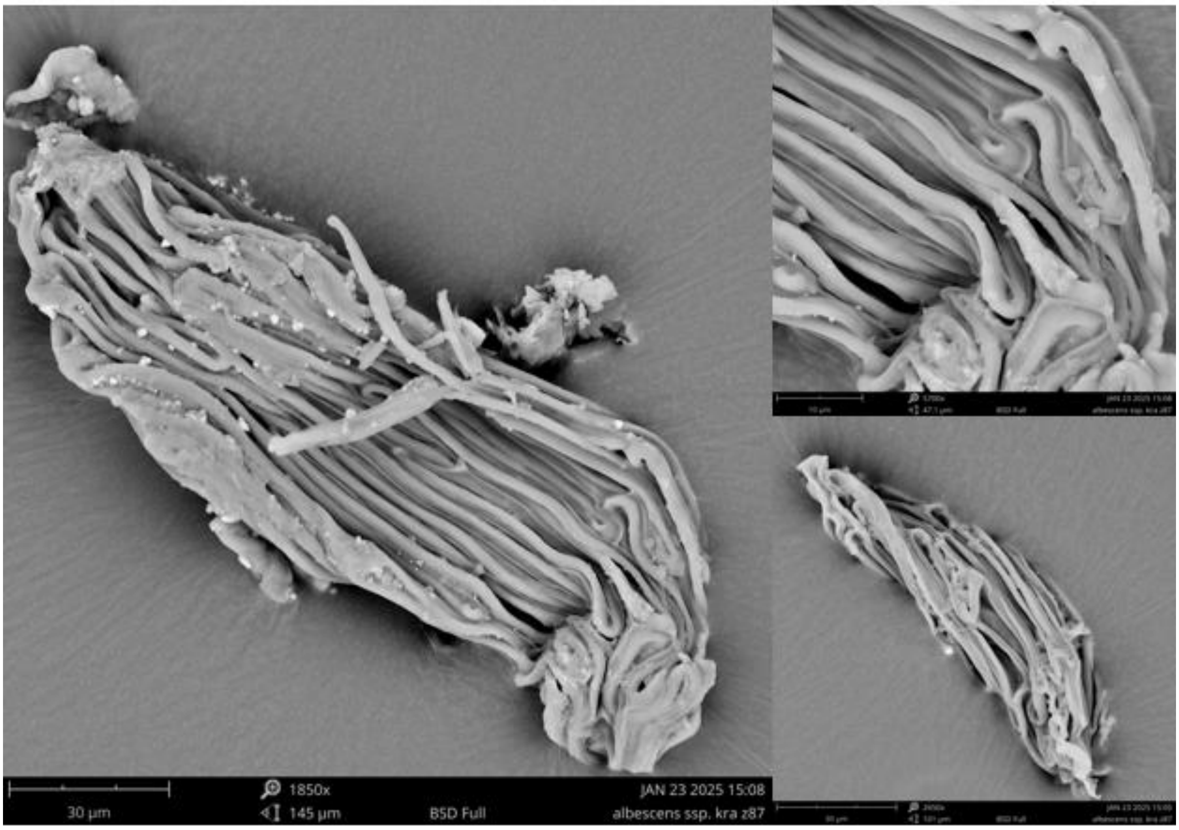

Fig.S23. *Polystachya albescens* ssp. *kraenzlinii* (Rolfe) Summerh. (P. alb. ssp. kra2)

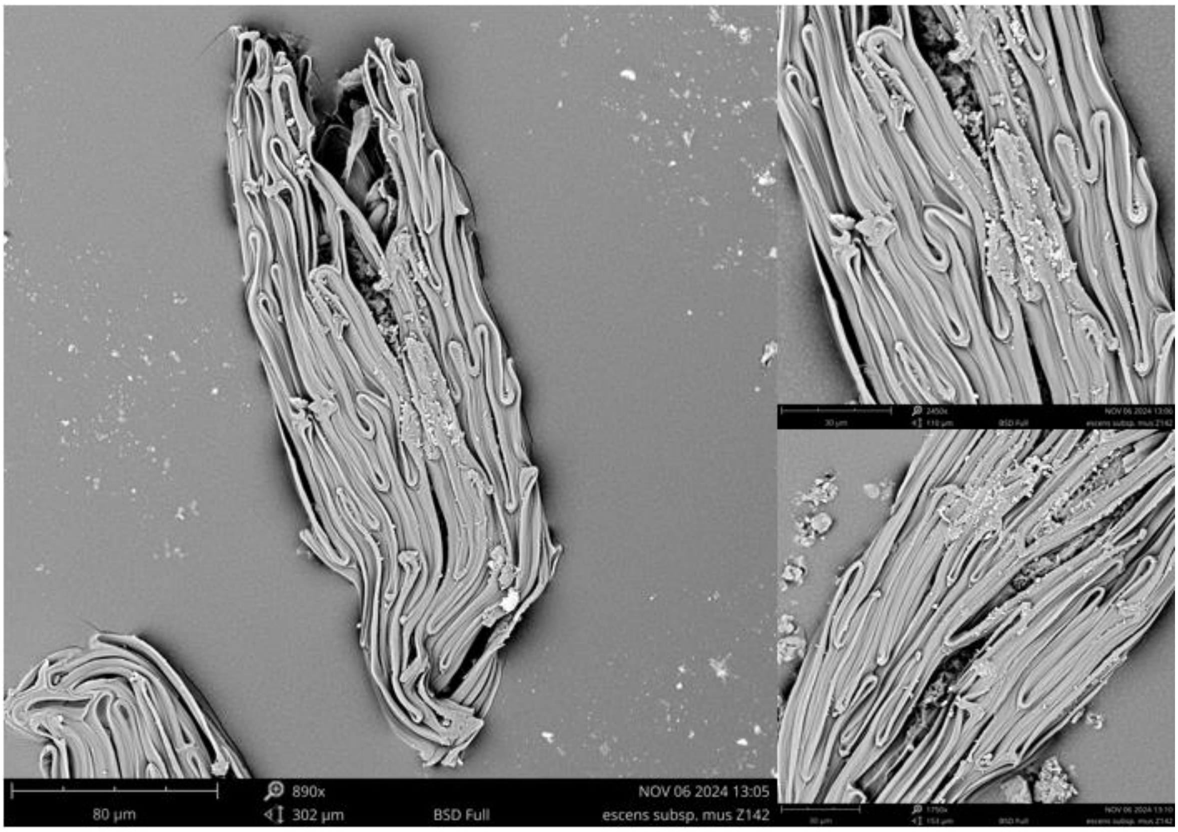

Fig.S24. *Polystachya albescens* ssp. *musozensis* (Rendle) Summerh. (P. alb. ssp. mus)

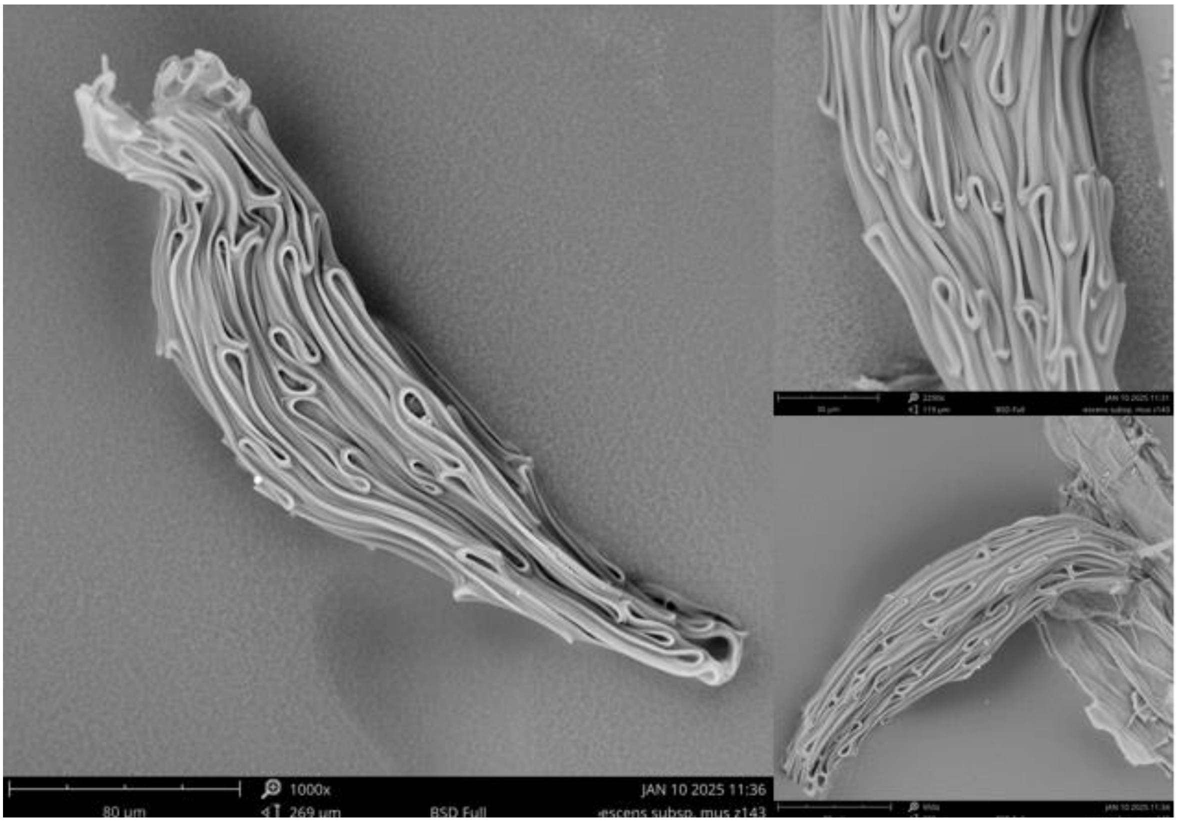

Fig.S25. *Polystachya albescens* ssp. *musozensis* (Rendle) Summerh. (P. alb. ssp. mus2)

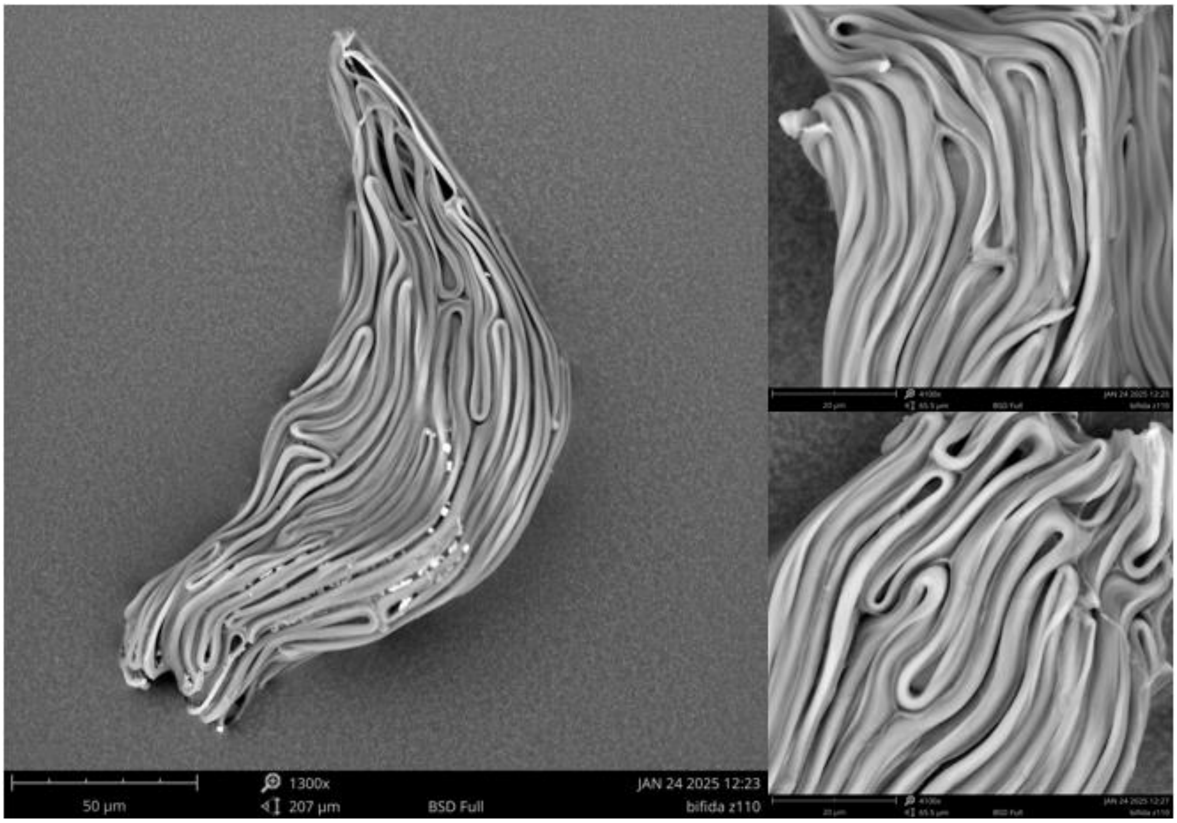

Fig.S26. *Polystachya albsescens* ssp. *polyphylla* (Summerh.) Stévart (P. alb. ssp. pol)

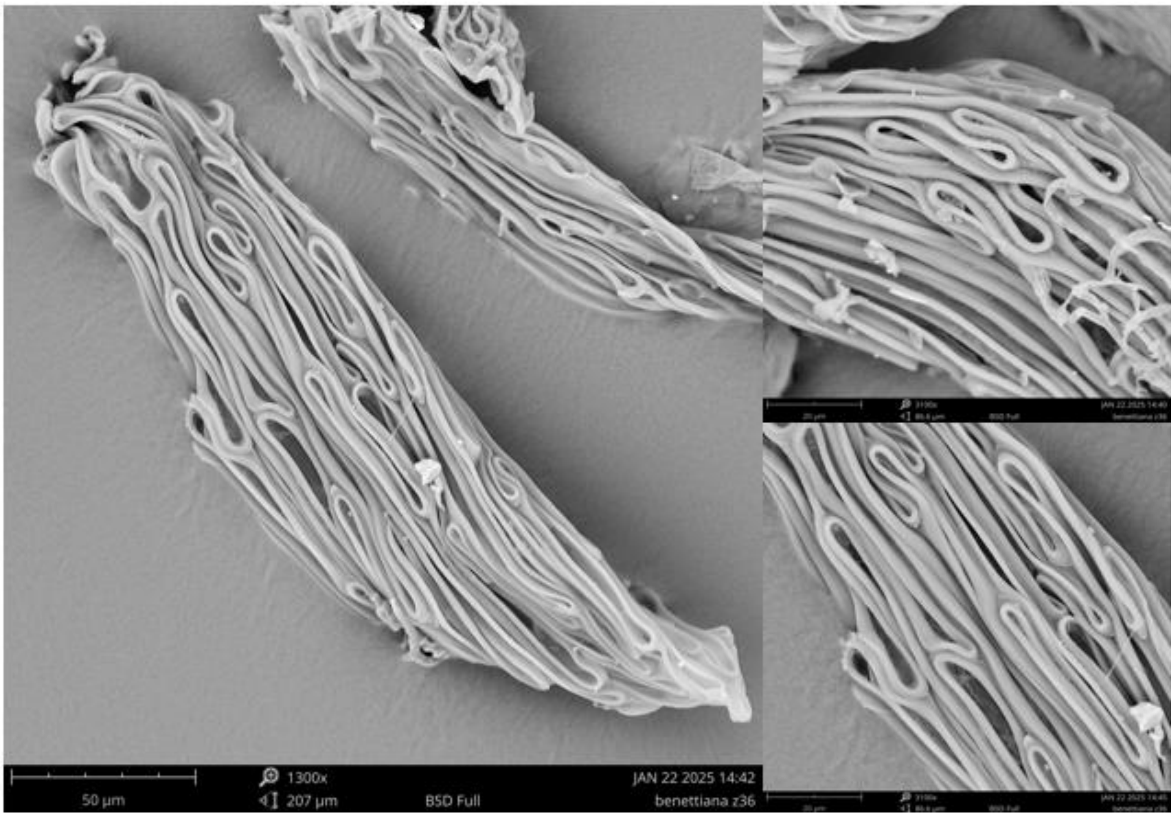

Fig.S27. *Polystachya bennettiana* Rchb.f. (P. ben2)

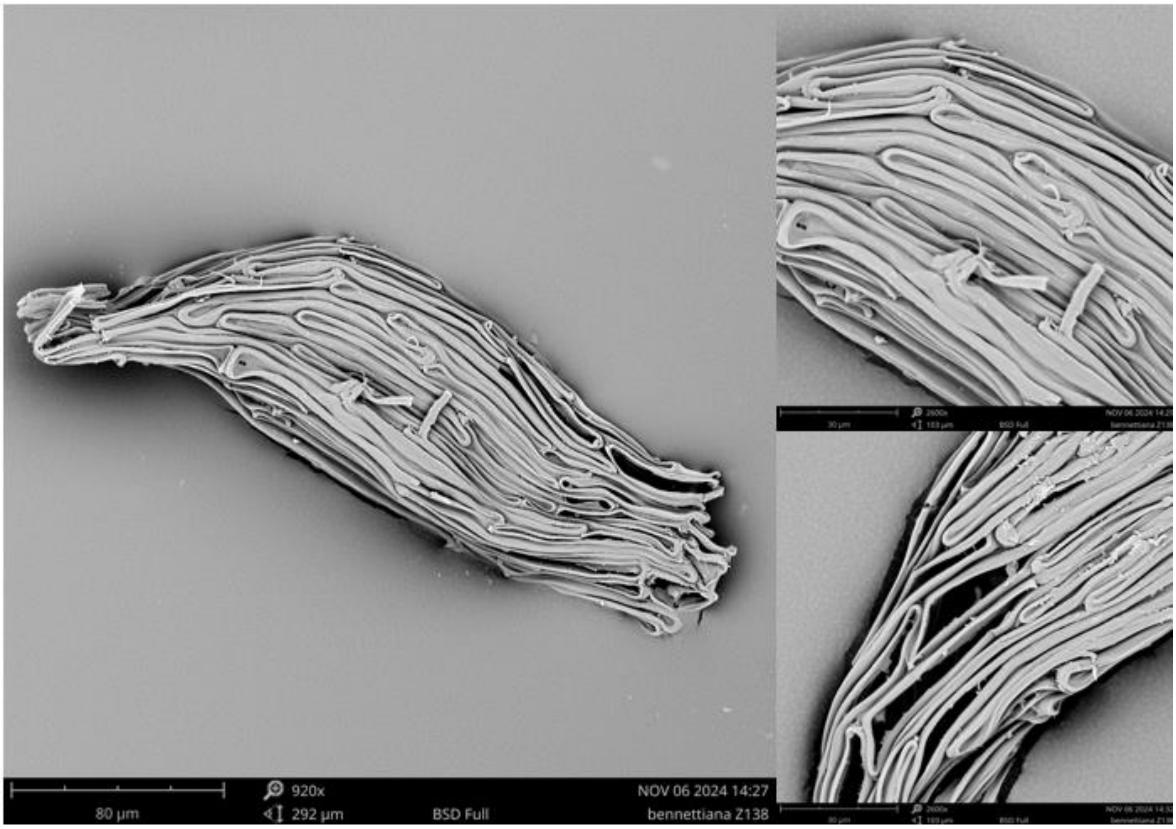

Fig.S28. *Polystachya bennettiana* Rchb.f. (P. ben4)

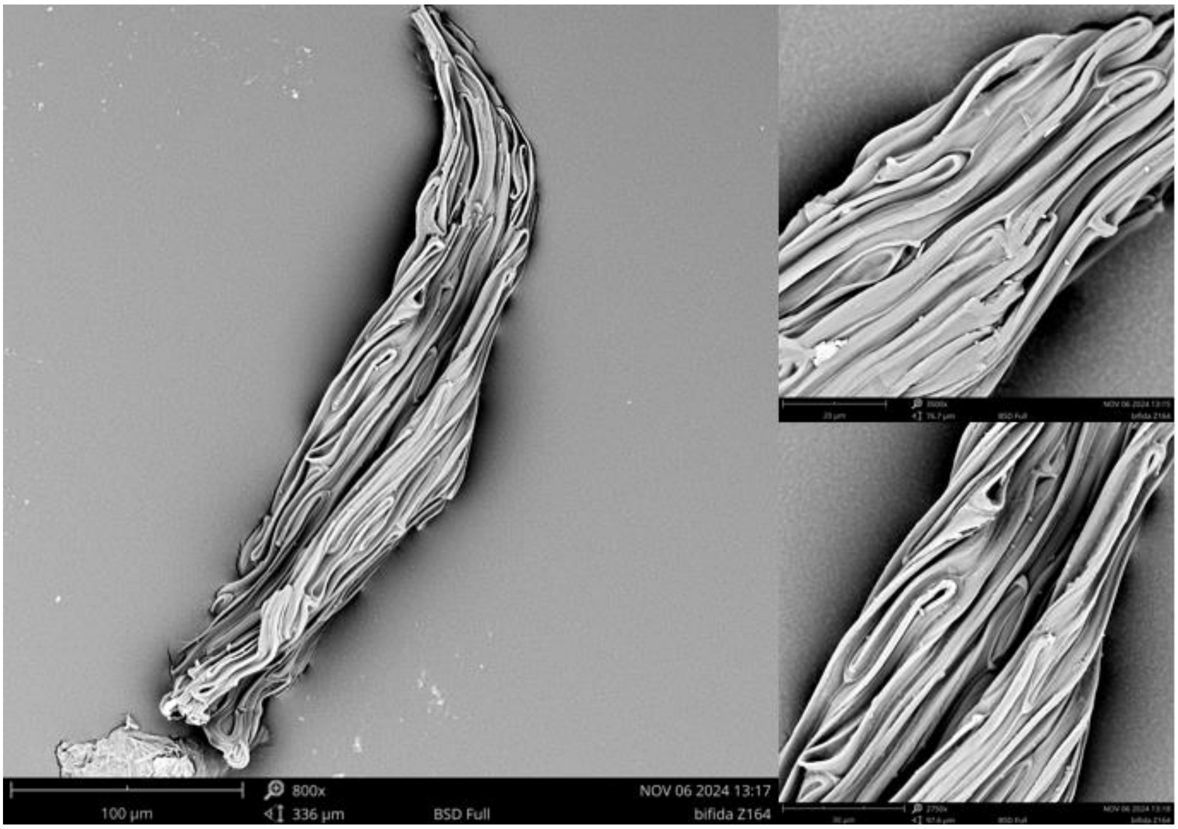

Fig.S29. *Polystachya bifida* Lindl. (P. bif)

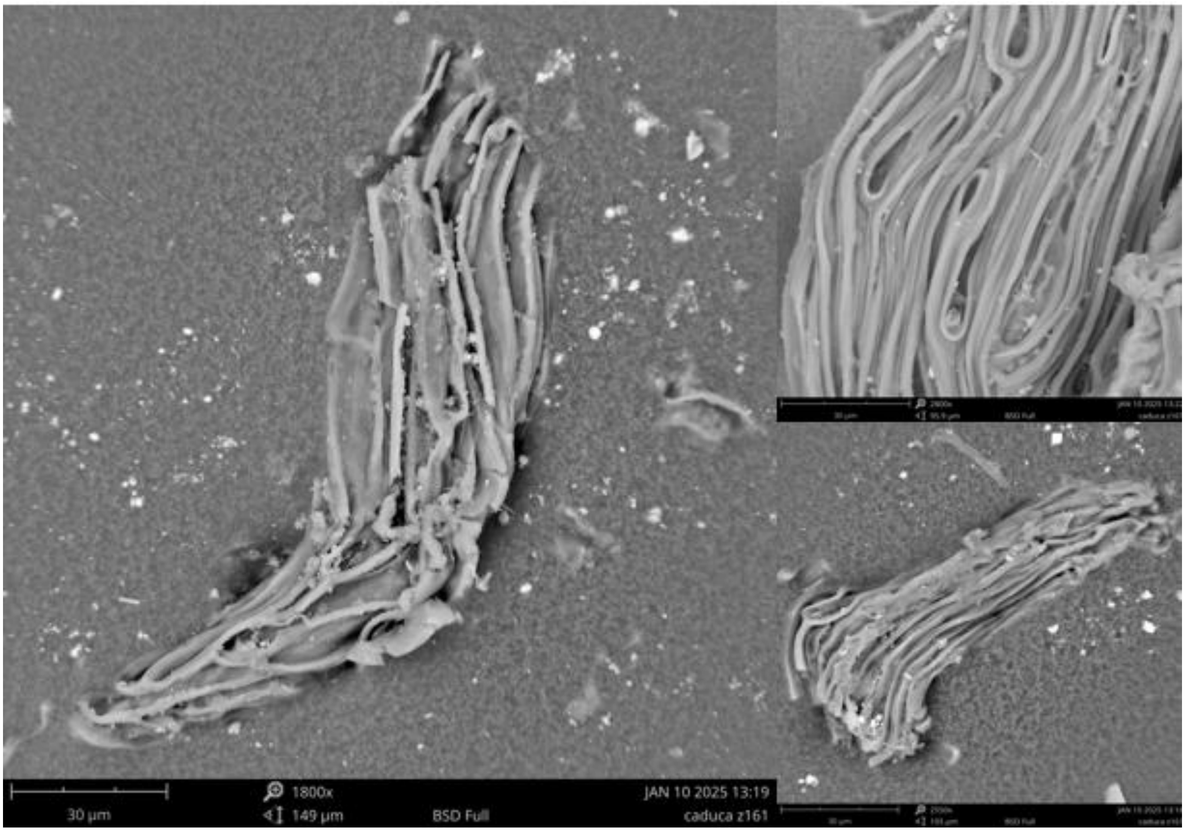

Fig.S30. *Polystachya caduca* Rchb.f. (P. cad)

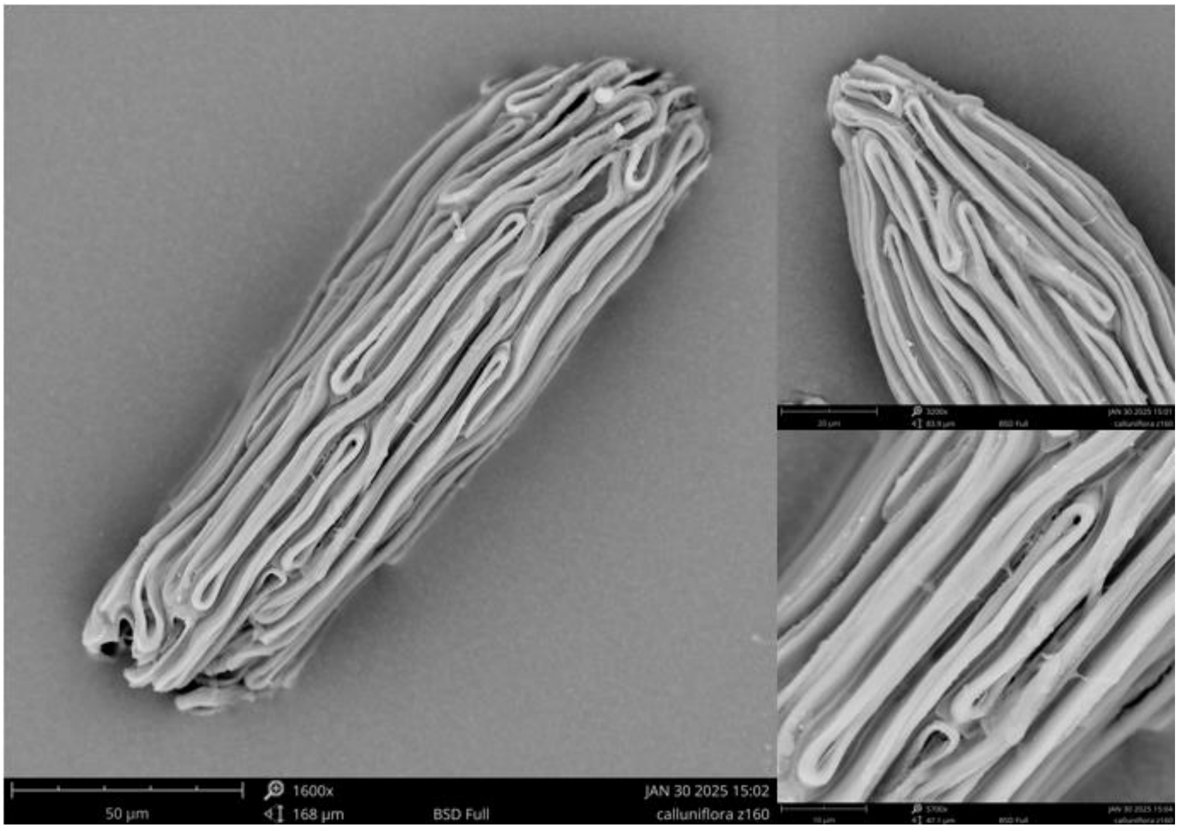

Fig.S31. *Polystachya calluniflora* Kraenzl. (P. cal)

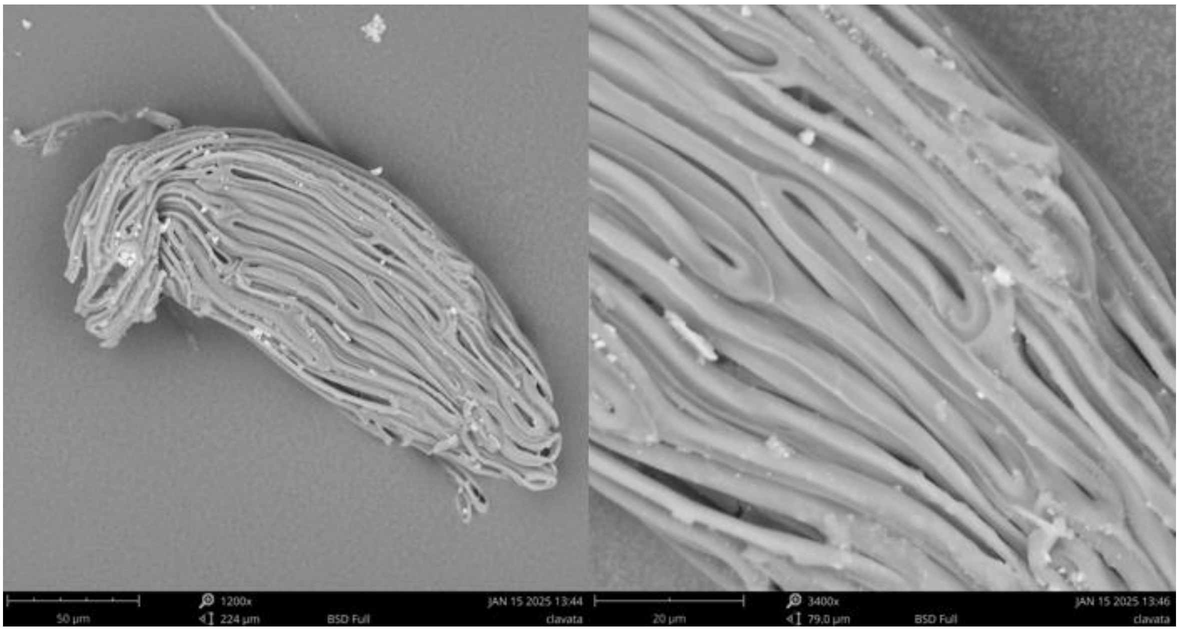

Fig.S32. *Polystachya clavata* Lindl. (P. cla)

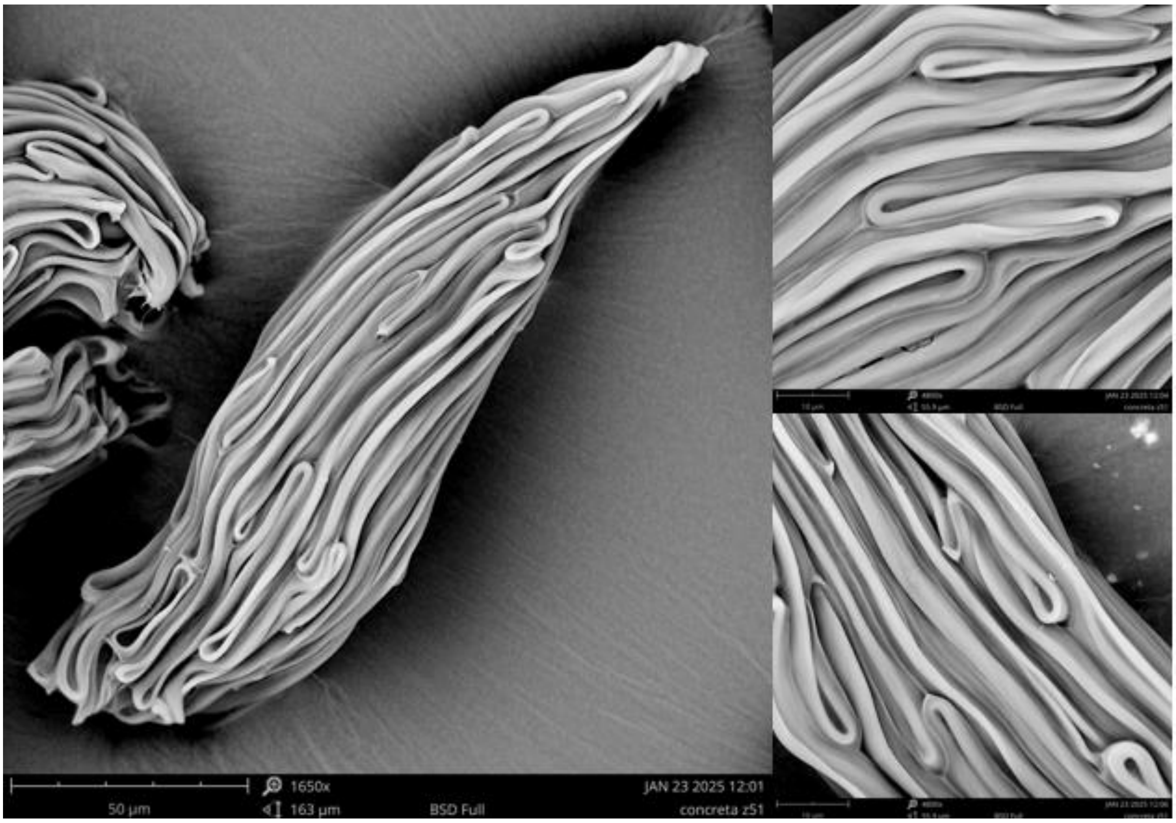

Fig.S33. *Polystachya concreta* (Jacq.) Garay & H.R.Sweet (P. con3)

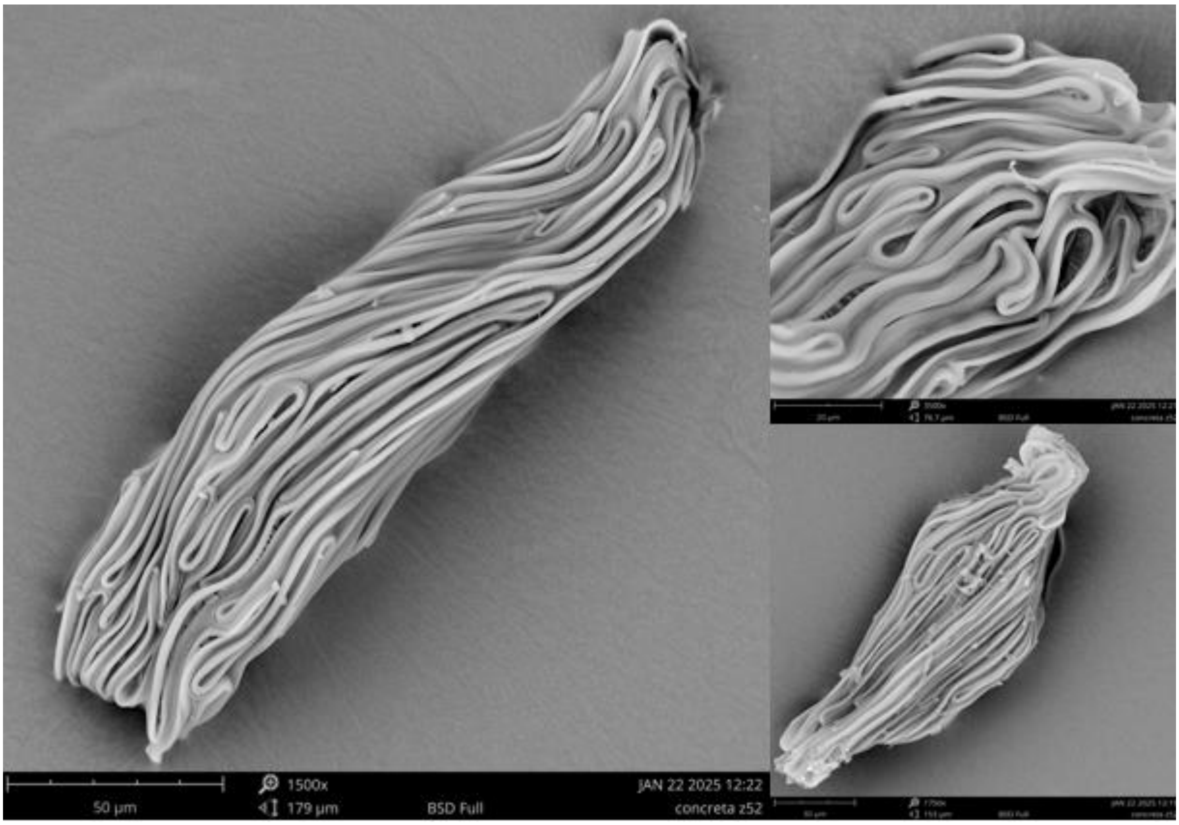

Fig.S34. *Polystachya concreta* (Jacq.) Garay & H.R.Sweet (P. con4)

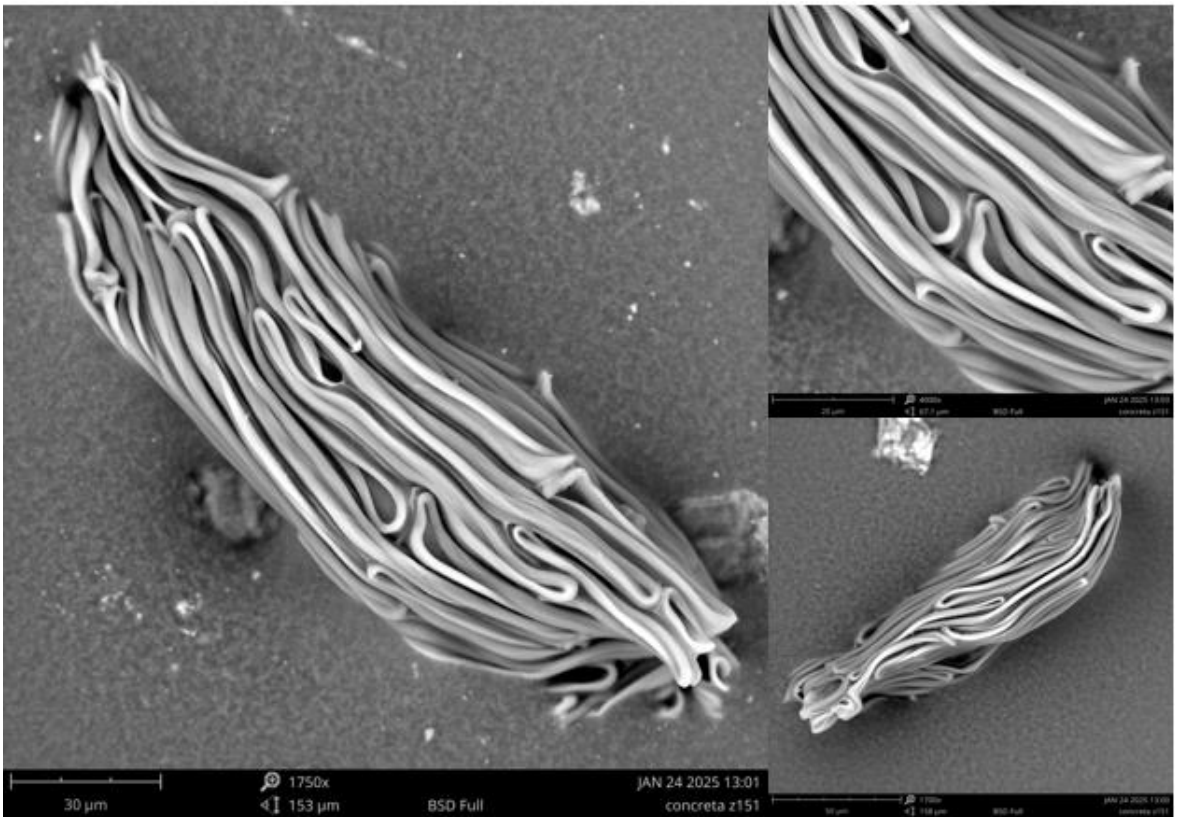

Fig.S35. *Polystachya concreta* (Jacq.) Garay & H.R.Sweet (P. con6)

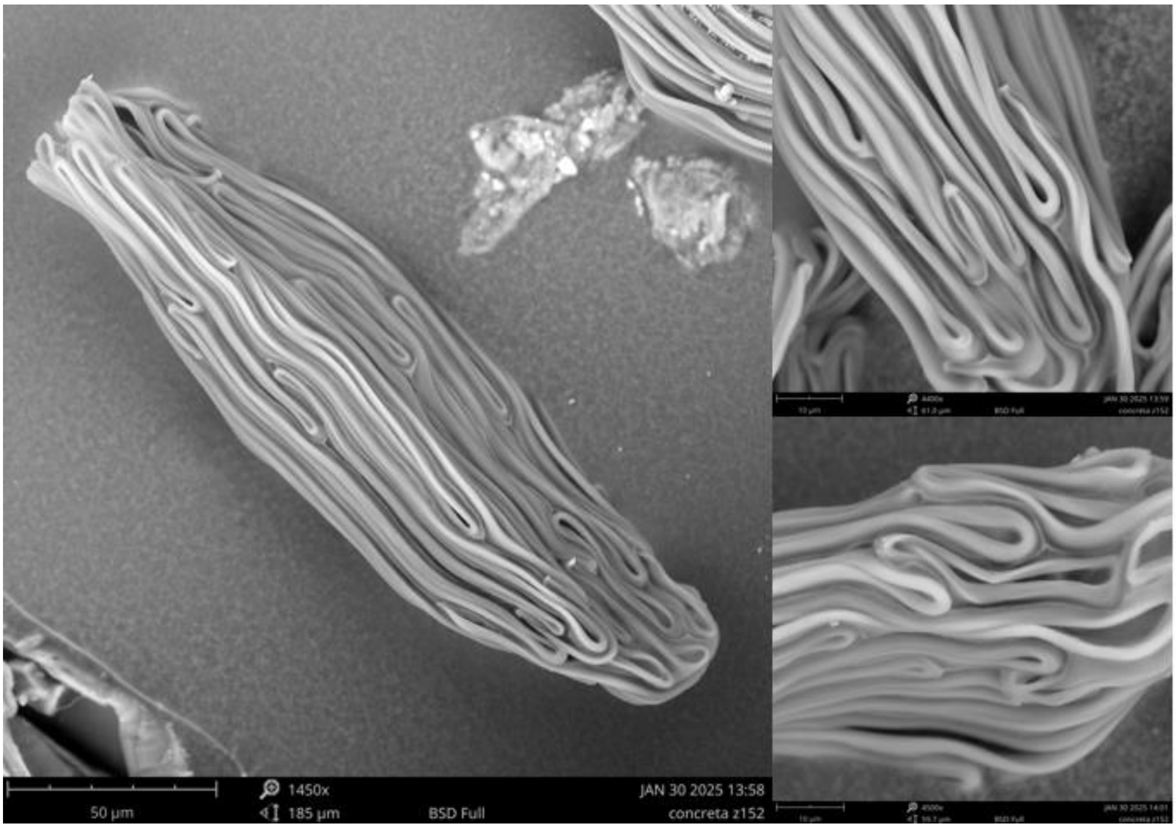

Fig.S36. *Polystachya concreta* (Jacq.) Garay & H.R.Sweet (P. con7)

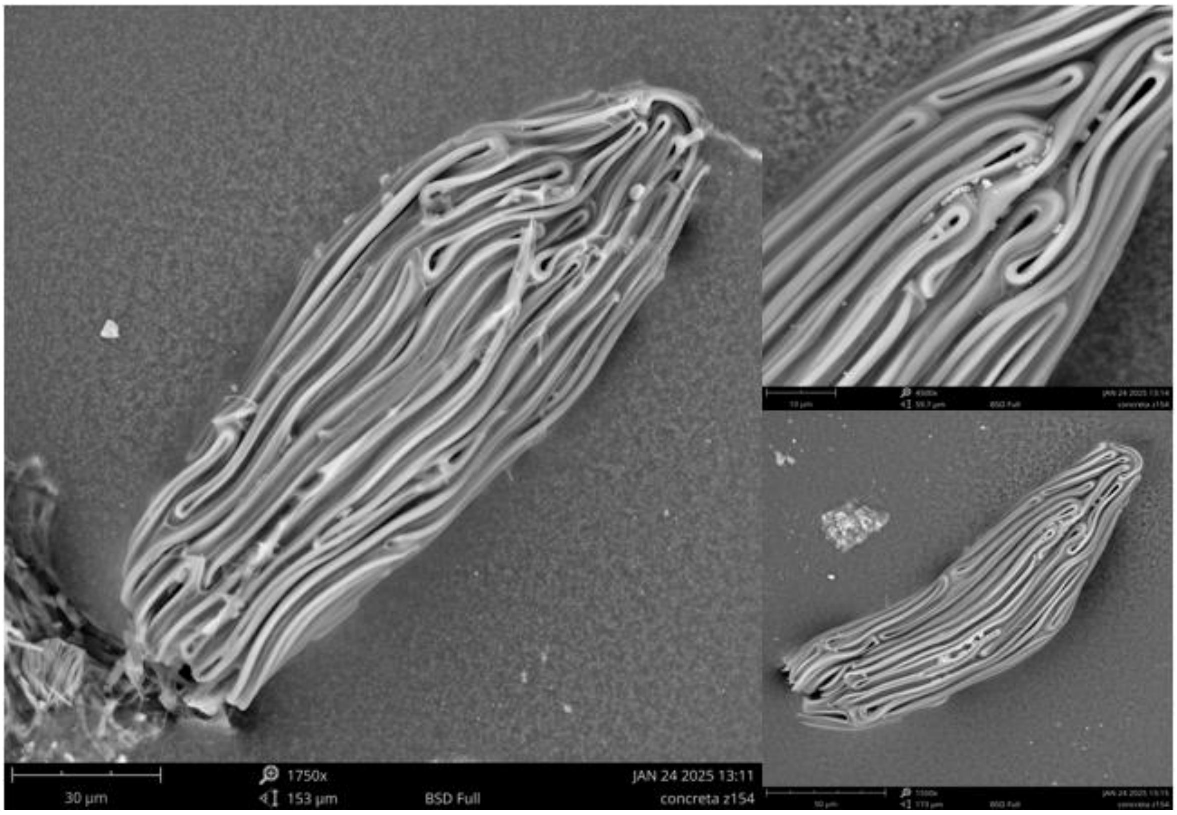

Fig.S37. *Polystachya concreta* (Jacq.) Garay & H.R.Sweet (P. con9)

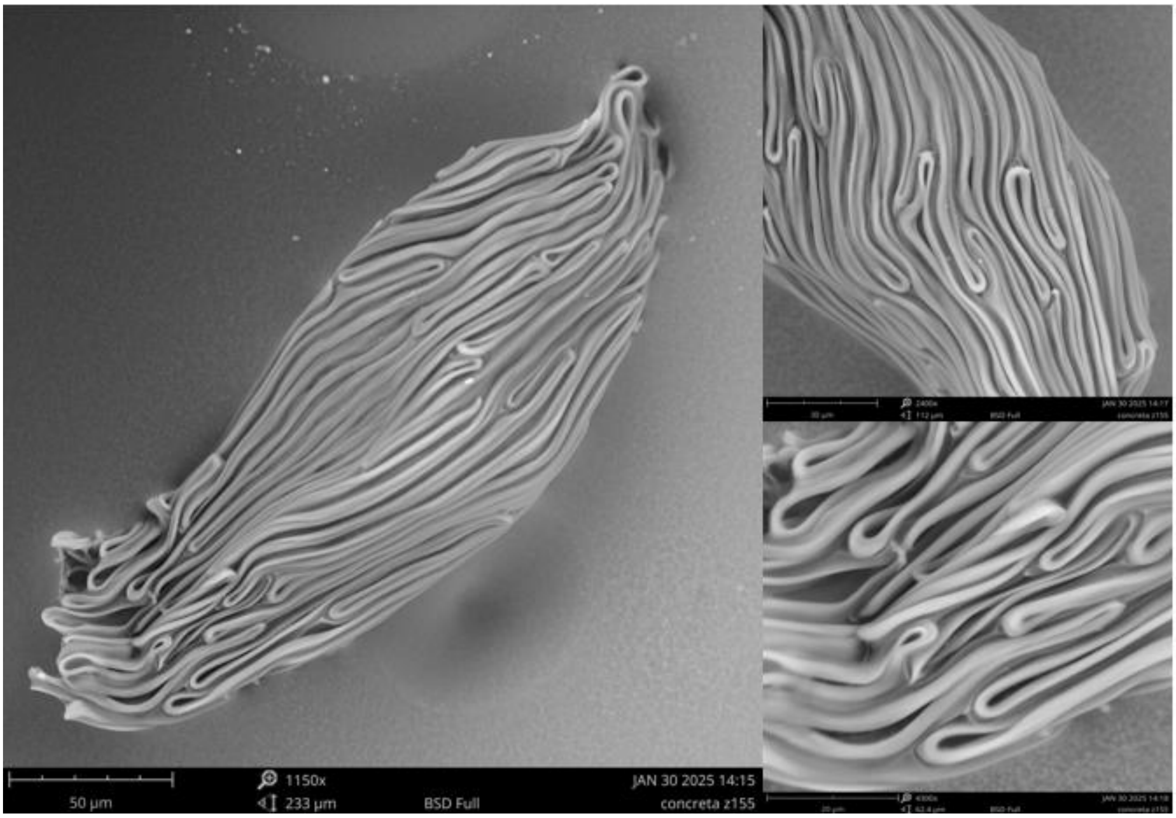

Fig.S38. *Polystachya concreta* (Jacq.) Garay & H.R.Sweet (P. con10)

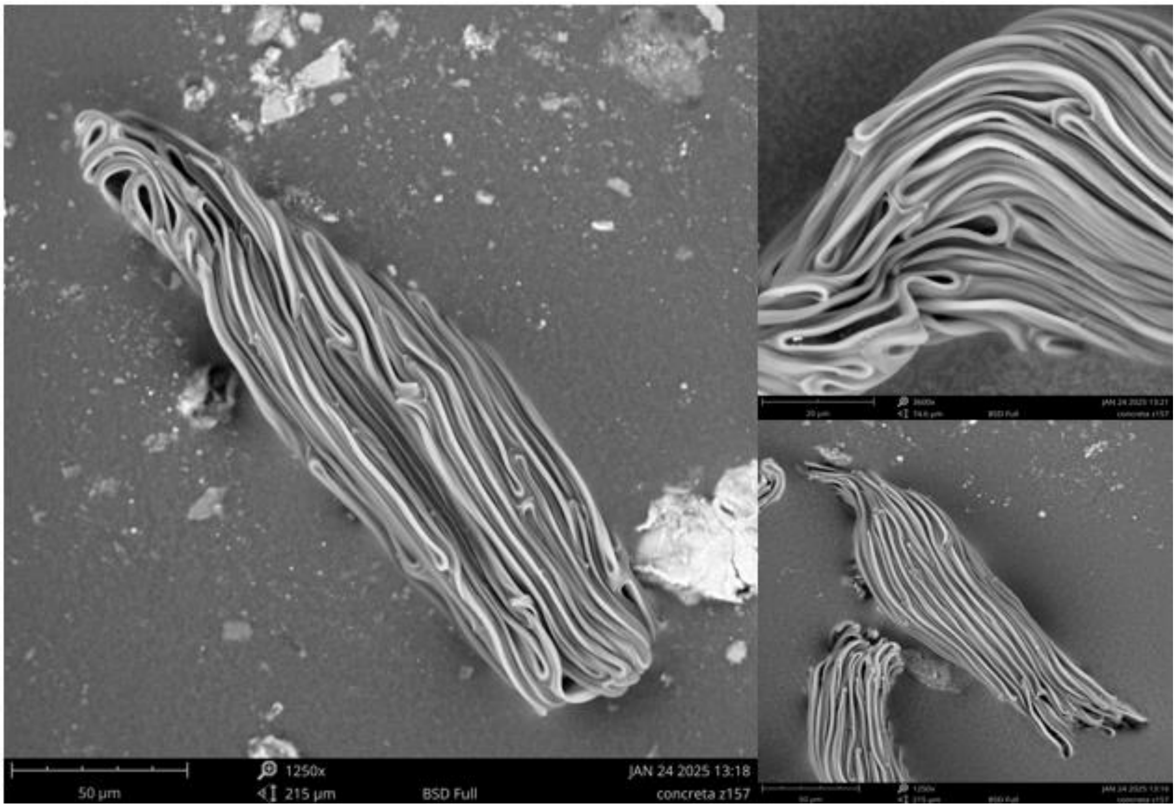

Fig.S39. *Polystachya concreta* (Jacq.) Garay & H.R.Sweet (P. con11)

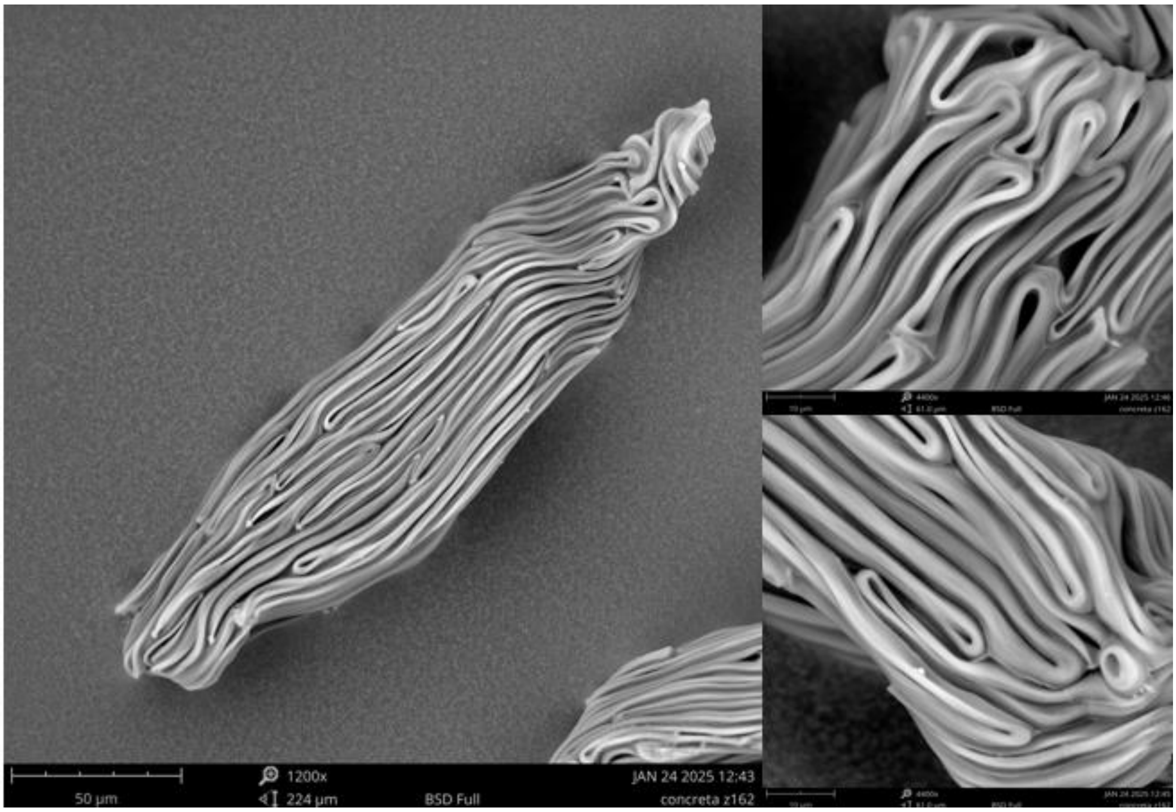

Fig.S40. *Polystachya concreta* (Jacq.) Garay & H.R.Sweet (P. con13)

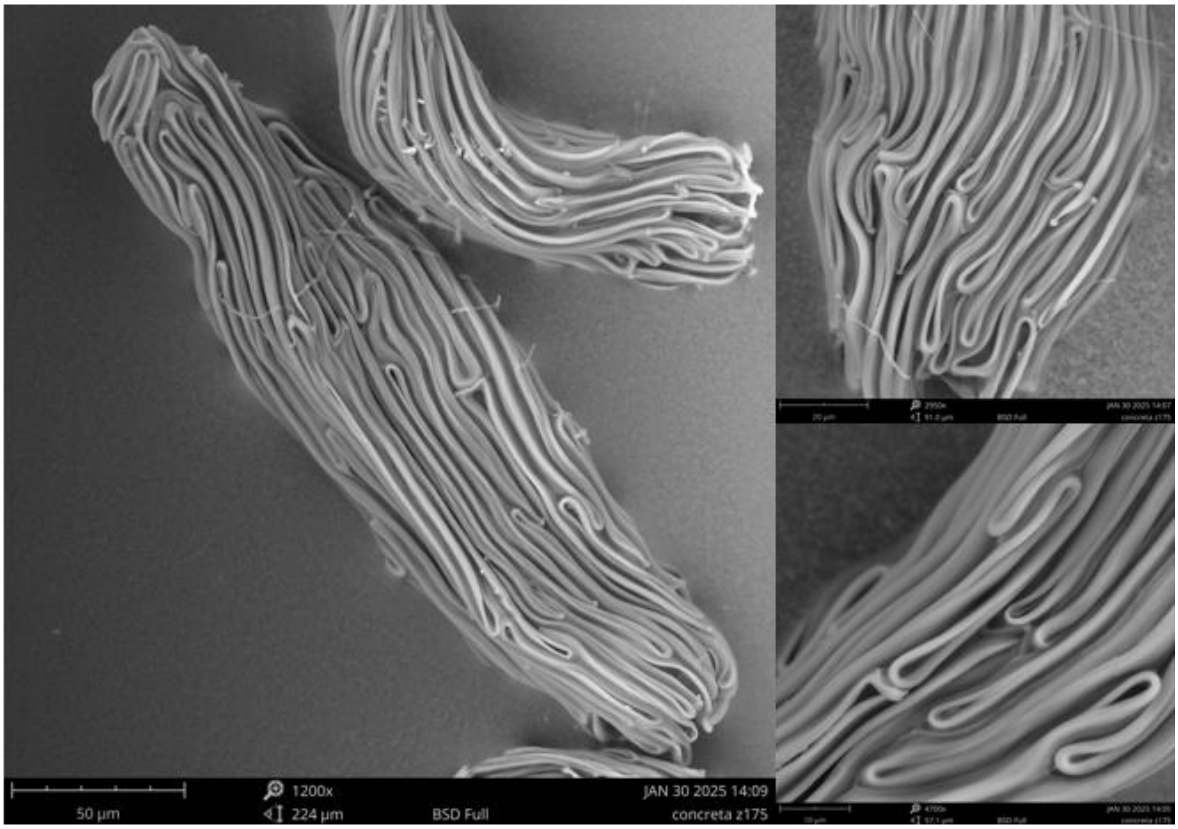

Fig.S41. *Polystachya concreta* (Jacq.) Garay & H.R.Sweet (P. con14)

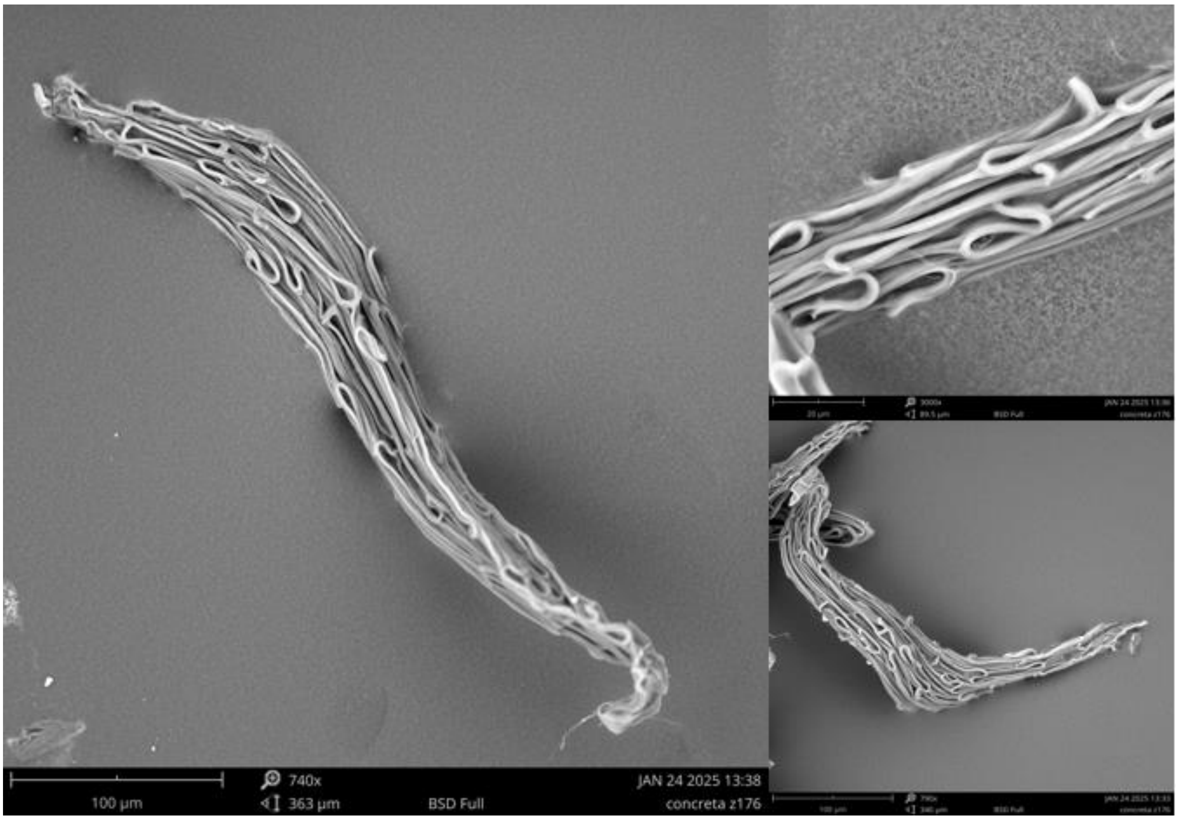

Fig.S42. *Polystachya concreta* (Jacq.) Garay & H.R.Sweet (P. con15)

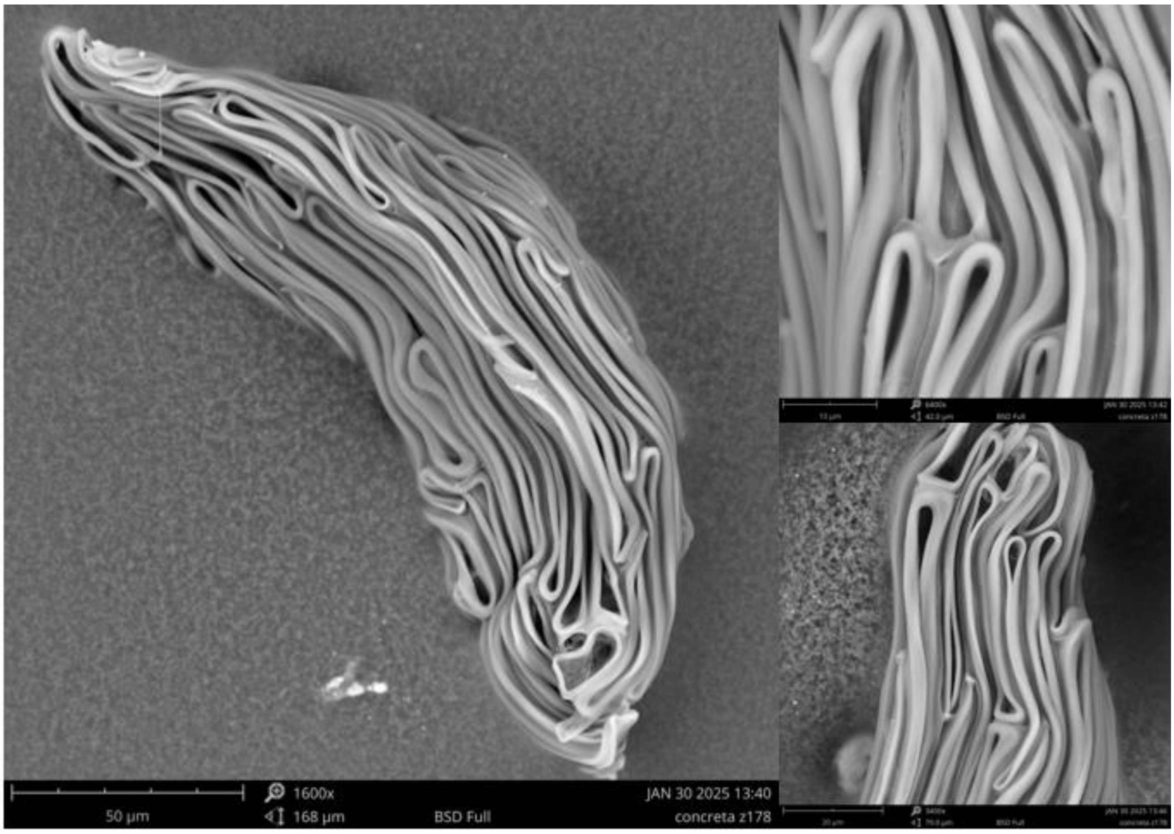

Fig.S43. *Polystachya concreta* (Jacq.) Garay & H.R.Sweet (P. con17)

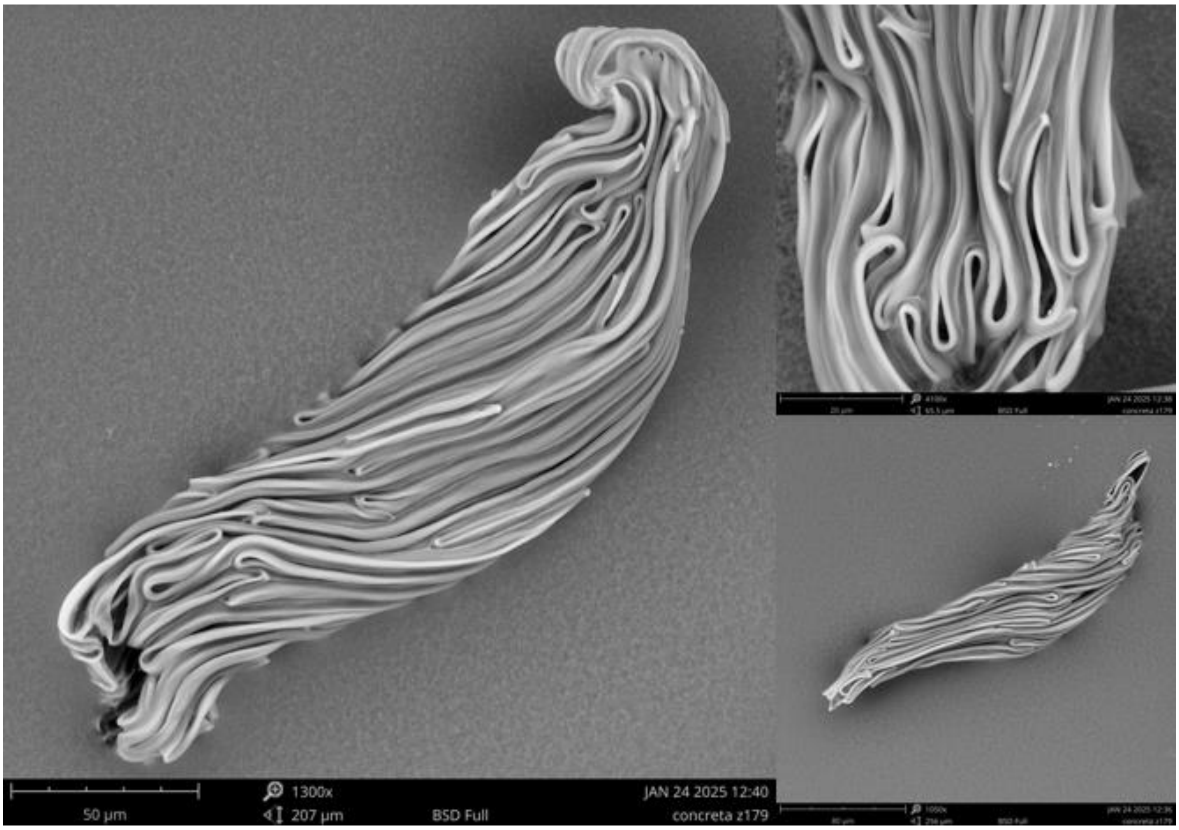

Fig.S44. *Polystachya concreta* (Jacq.) Garay & H.R.Sweet (P. con18)

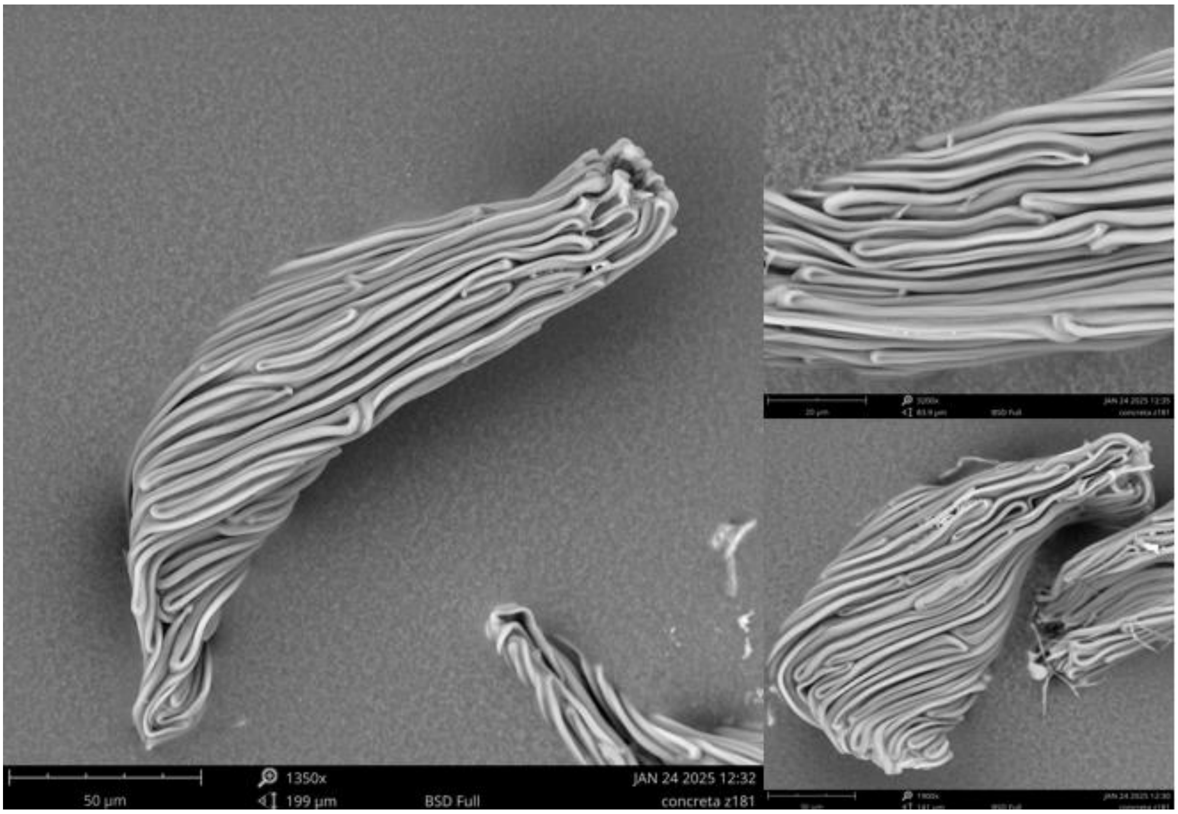

Fig.S45. *Polystachya concreta* (Jacq.) Garay & H.R.Sweet (P. con19)

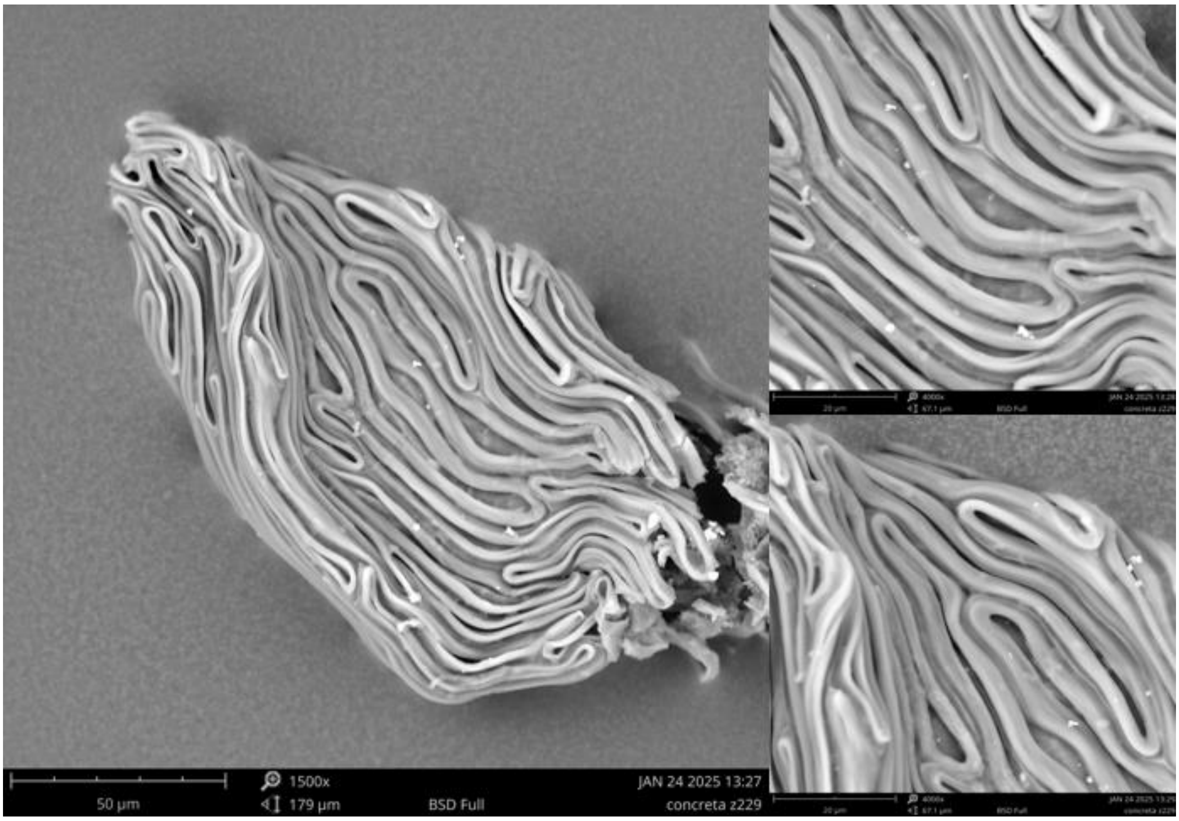

Fig.S46. *Polystachya concreta* (Jacq.) Garay & H.R.Sweet (P. con23)

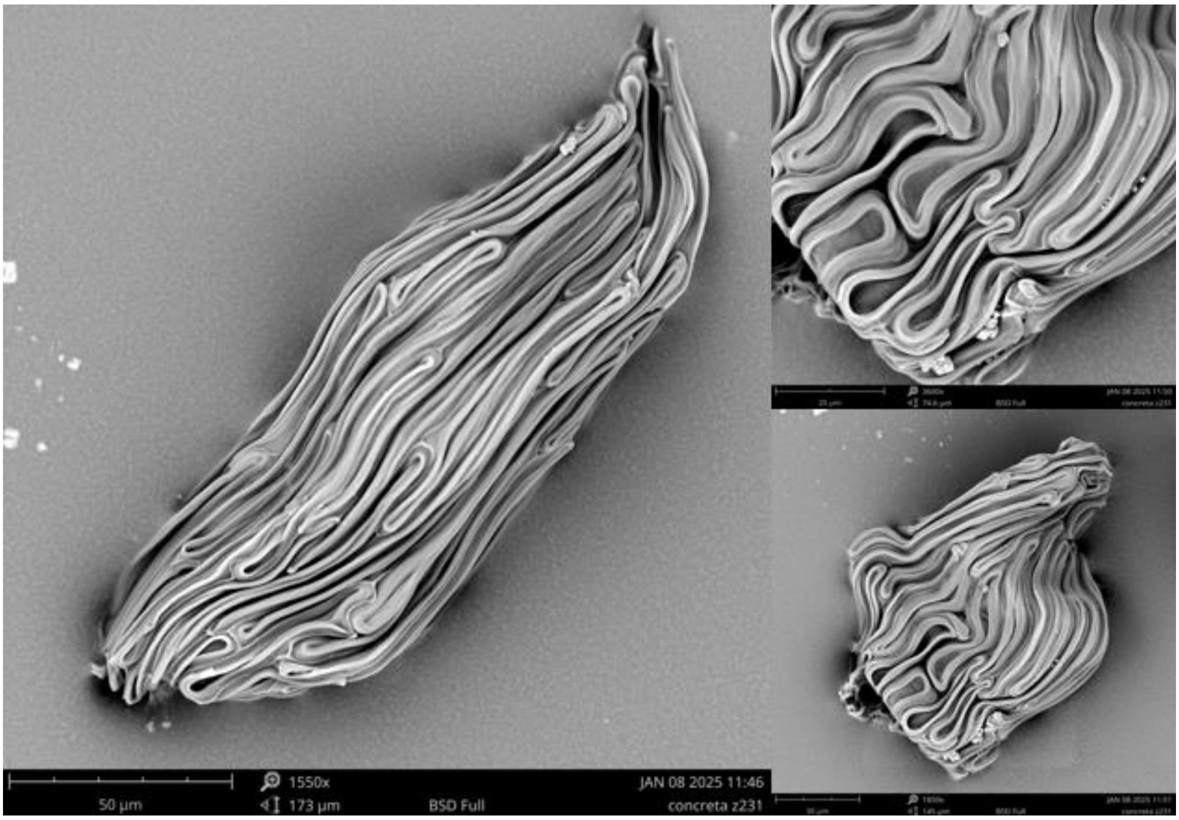

Fig.S47. *Polystachya concreta* (Jacq.) Garay & H.R.Sweet (P. con25)

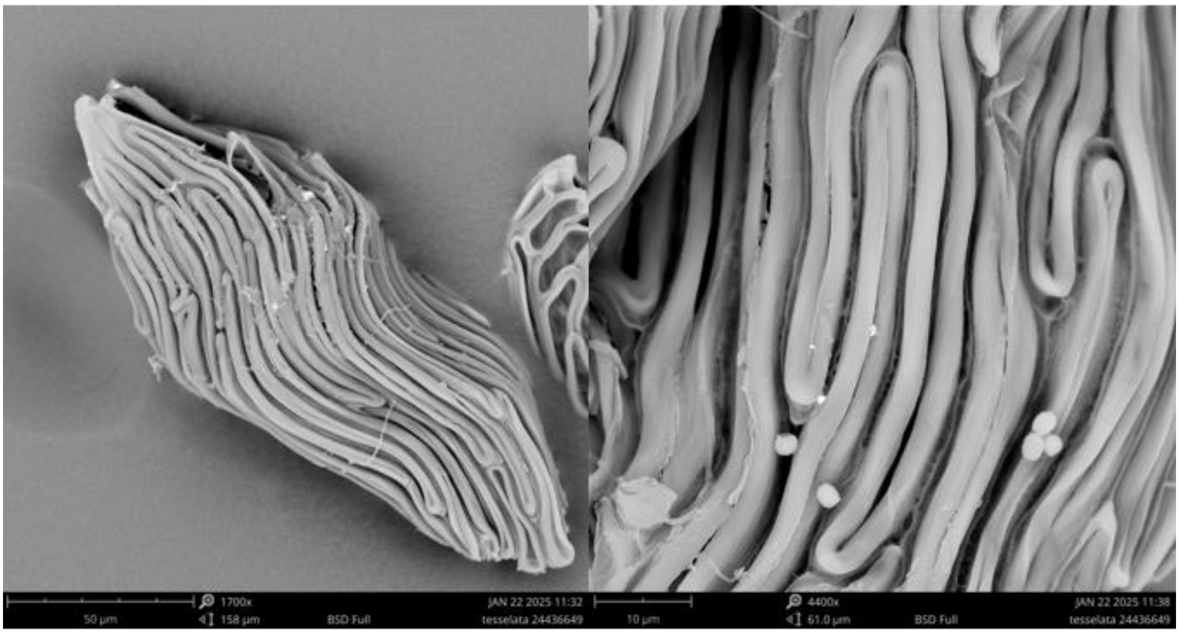

Fig.S48. *Polystachya concreta* (Jacq.) Garay & H.R.Sweet (P. con29)

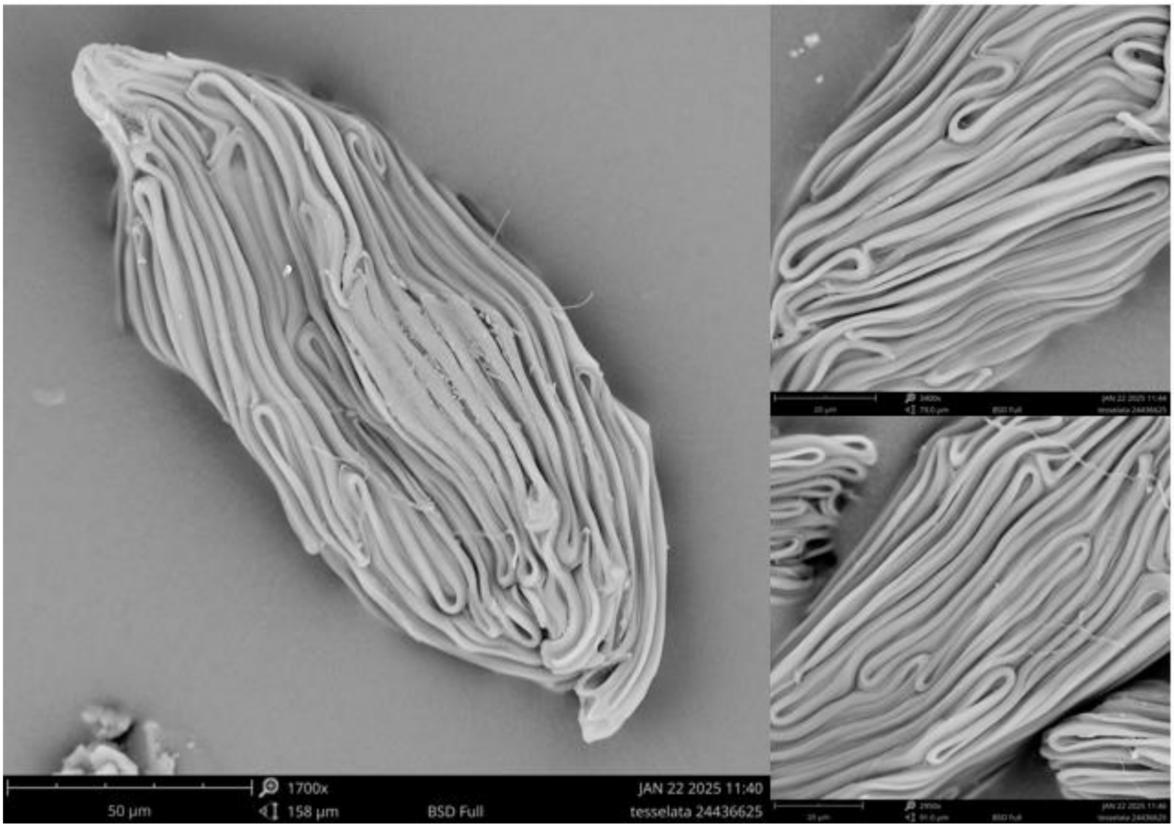

Fig.S49. *Polystachya concreta* (Jacq.) Garay & H.R.Sweet (P. con30)

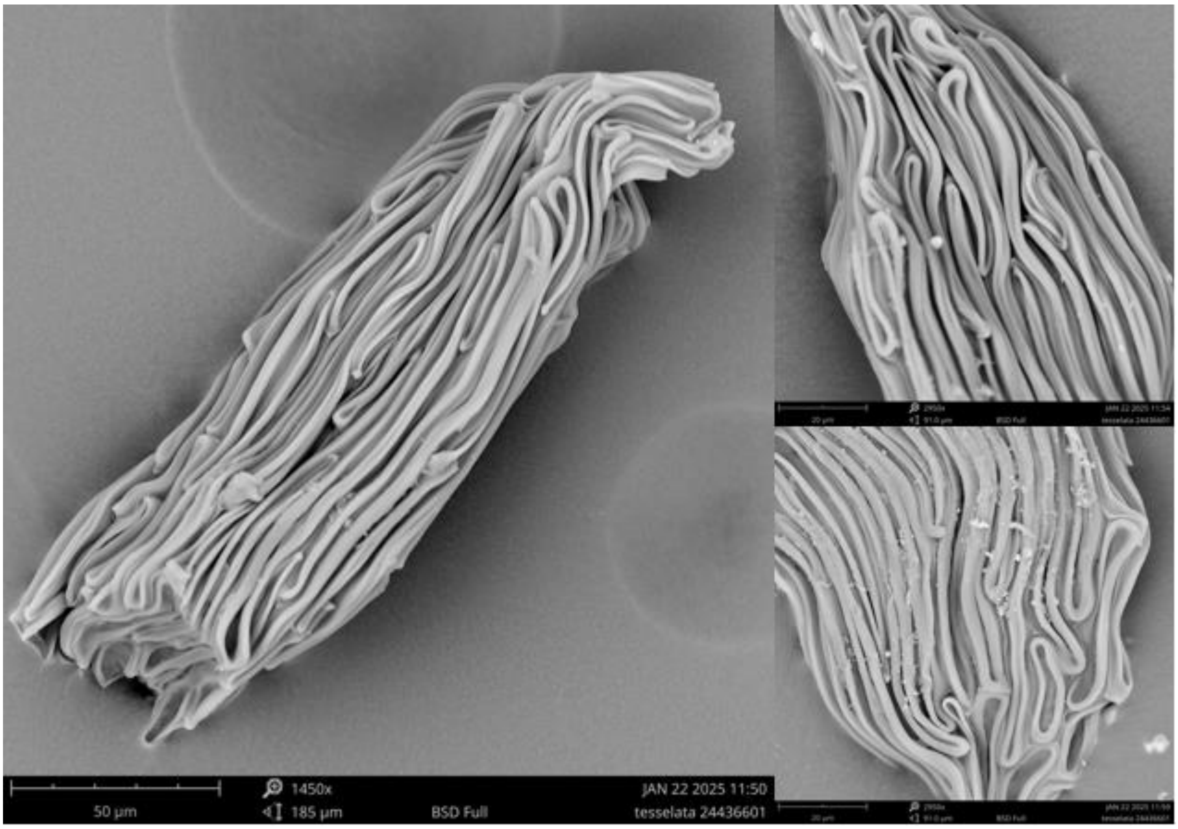

Fig.S50. *Polystachya concreta* (Jacq.) Garay & H.R.Sweet (P. con32)

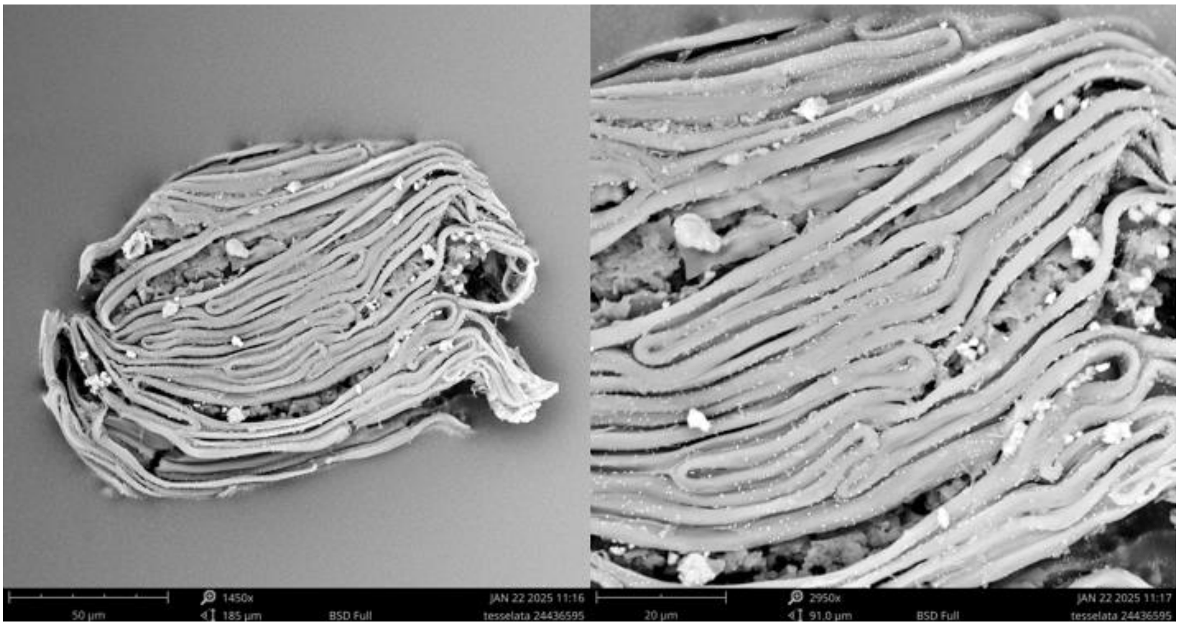

Fig.S51. *Polystachya concreta* (Jacq.) Garay & H.R.Sweet (P. con33)

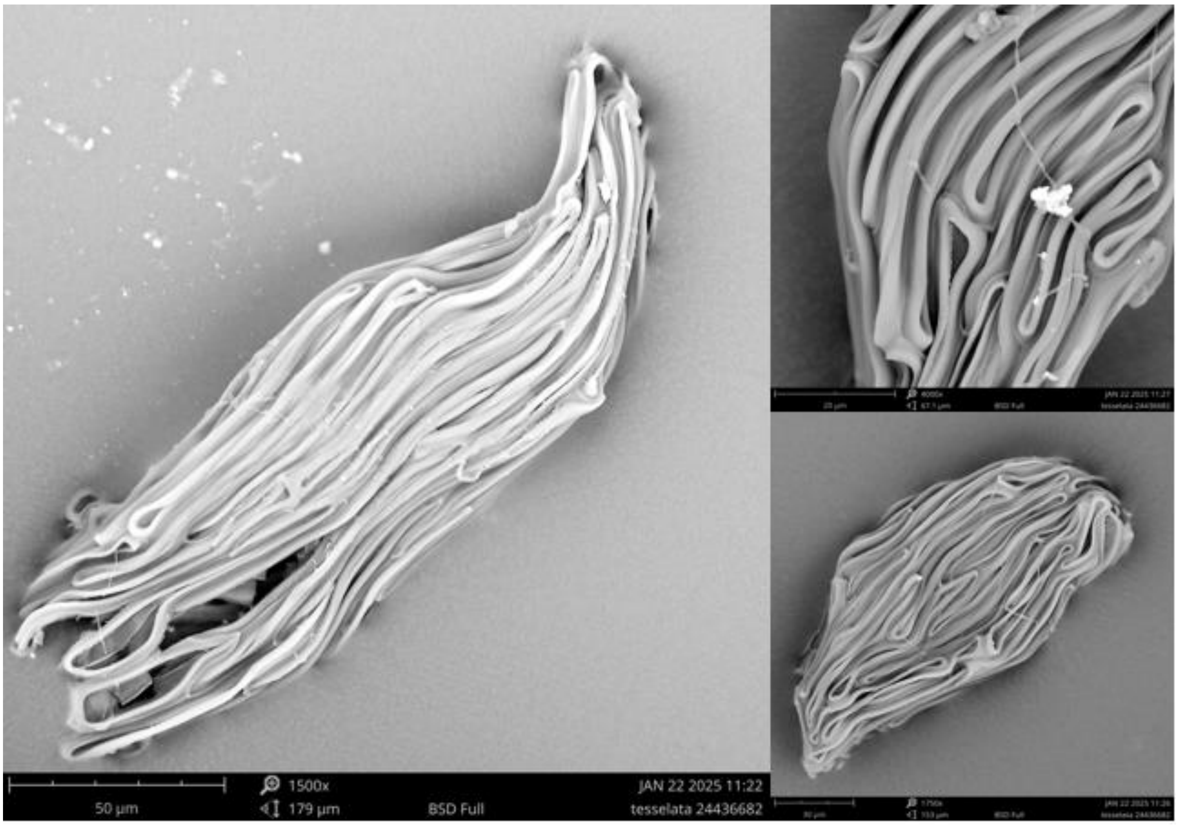

Fig.S52. *Polystachya concreta* (Jacq.) Garay & H.R.Sweet (P. con34)

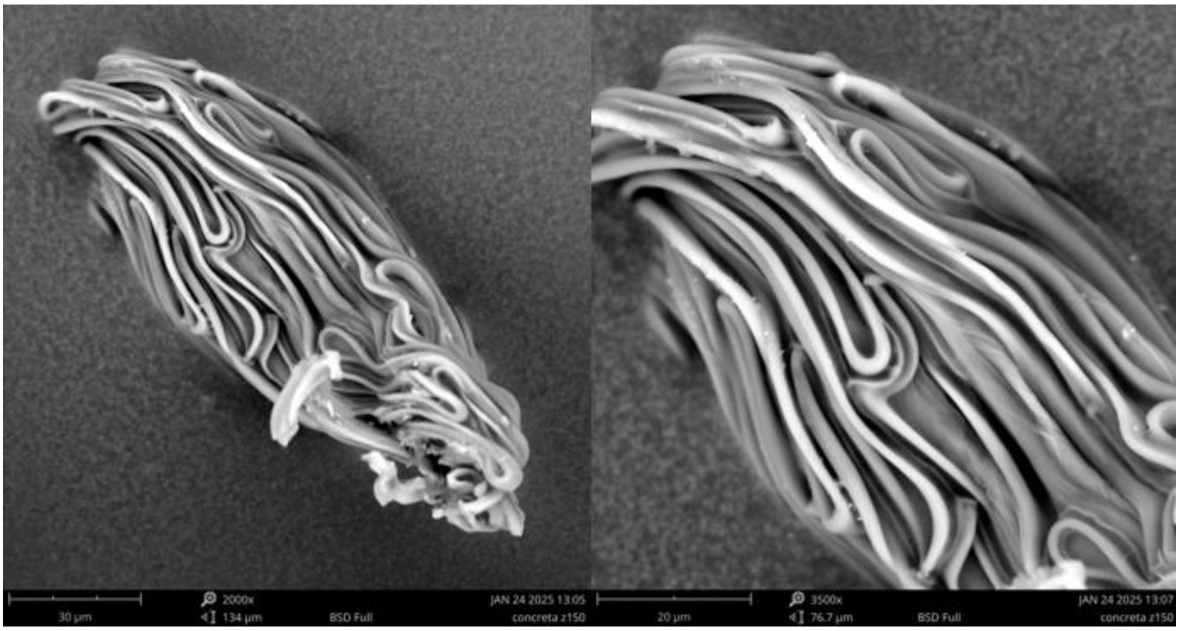

Fig.S53. *Polystachya concreta* (Jacq.) Garay & H.R.Sweet (P. con35)

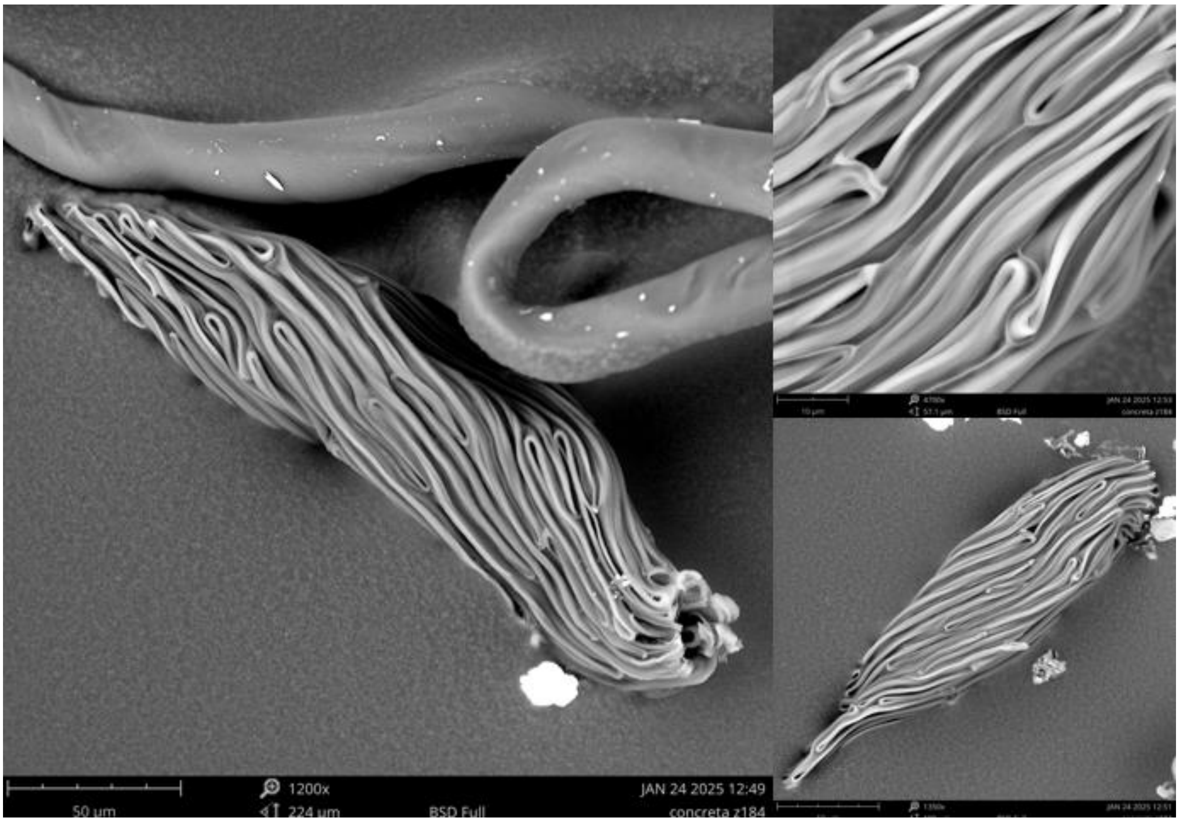

Fig.S54. *Polystachya concreta* (Jacq.) Garay & H.R.Sweet (P. con36)

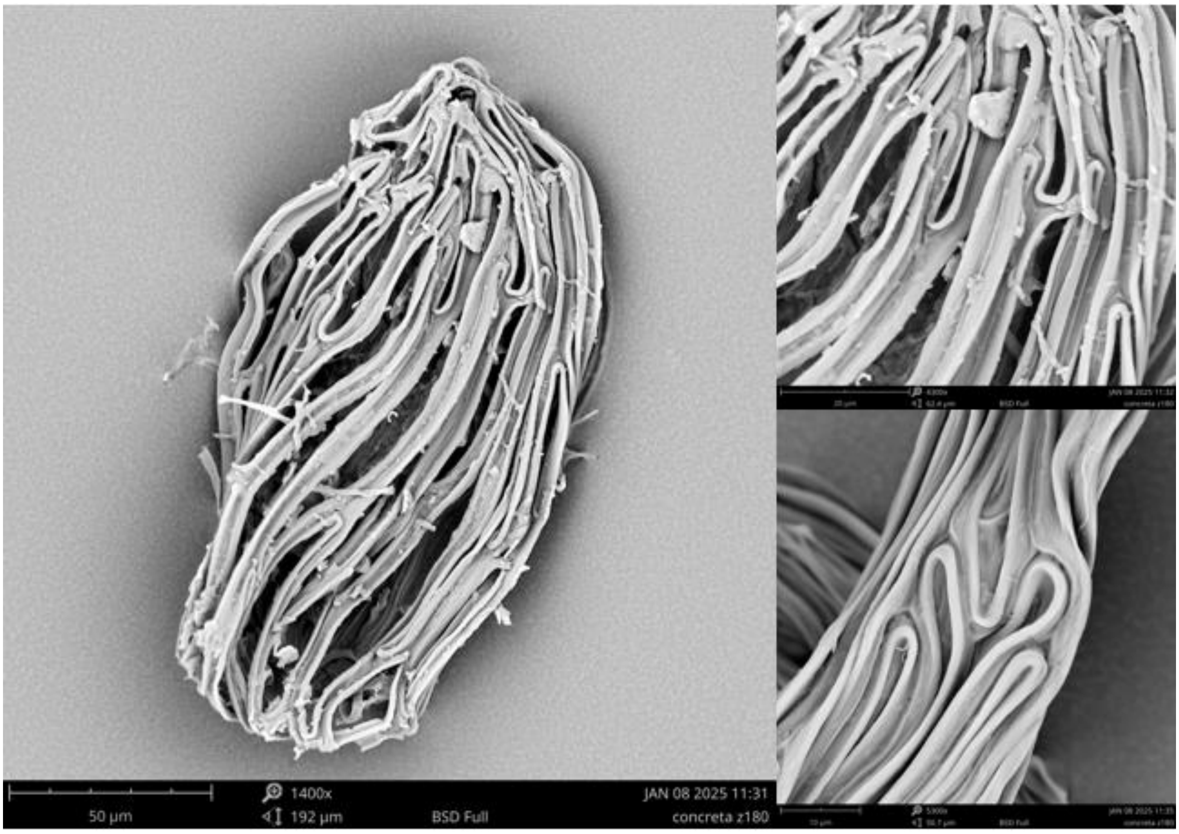

Fig.S55. *Polystachya concreta* (Jacq.) Garay & H.R.Sweet (P. con37)

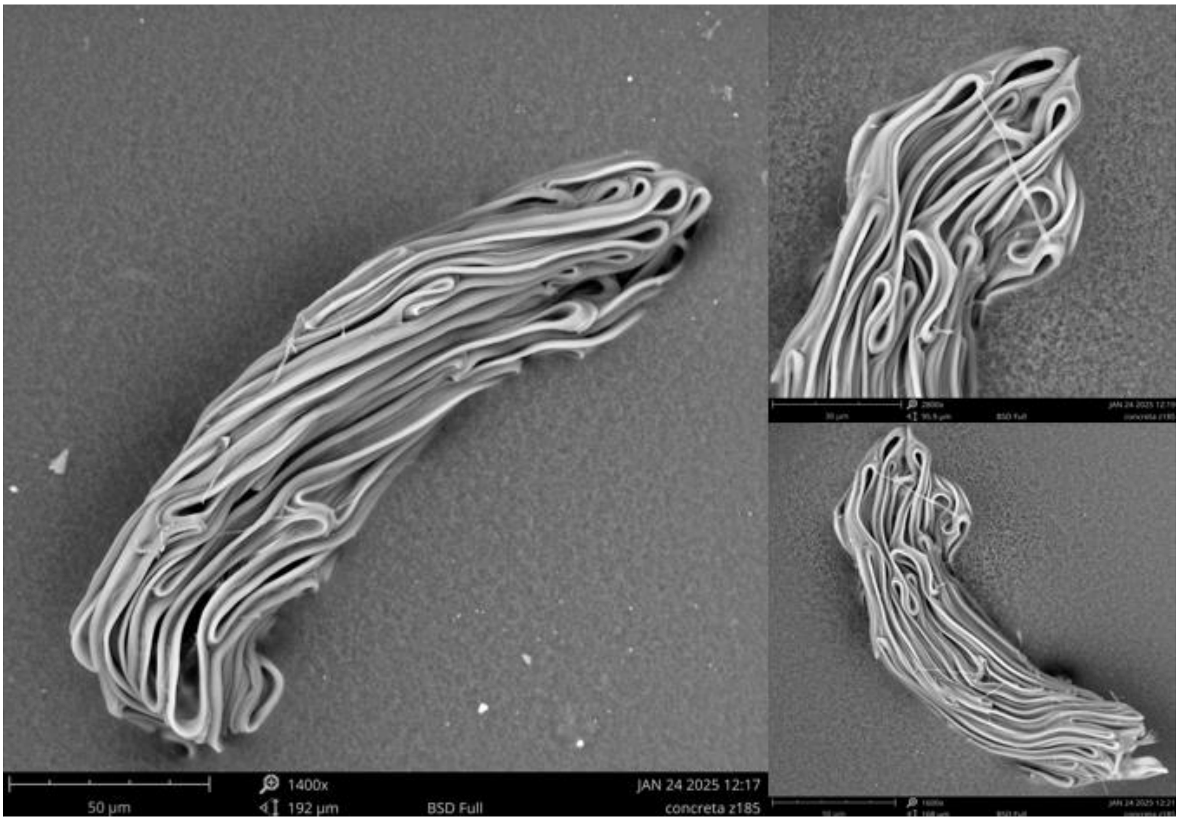

Fig.S56. *Polystachya concreta* (Jacq.) Garay & H.R.Sweet (P. con38)

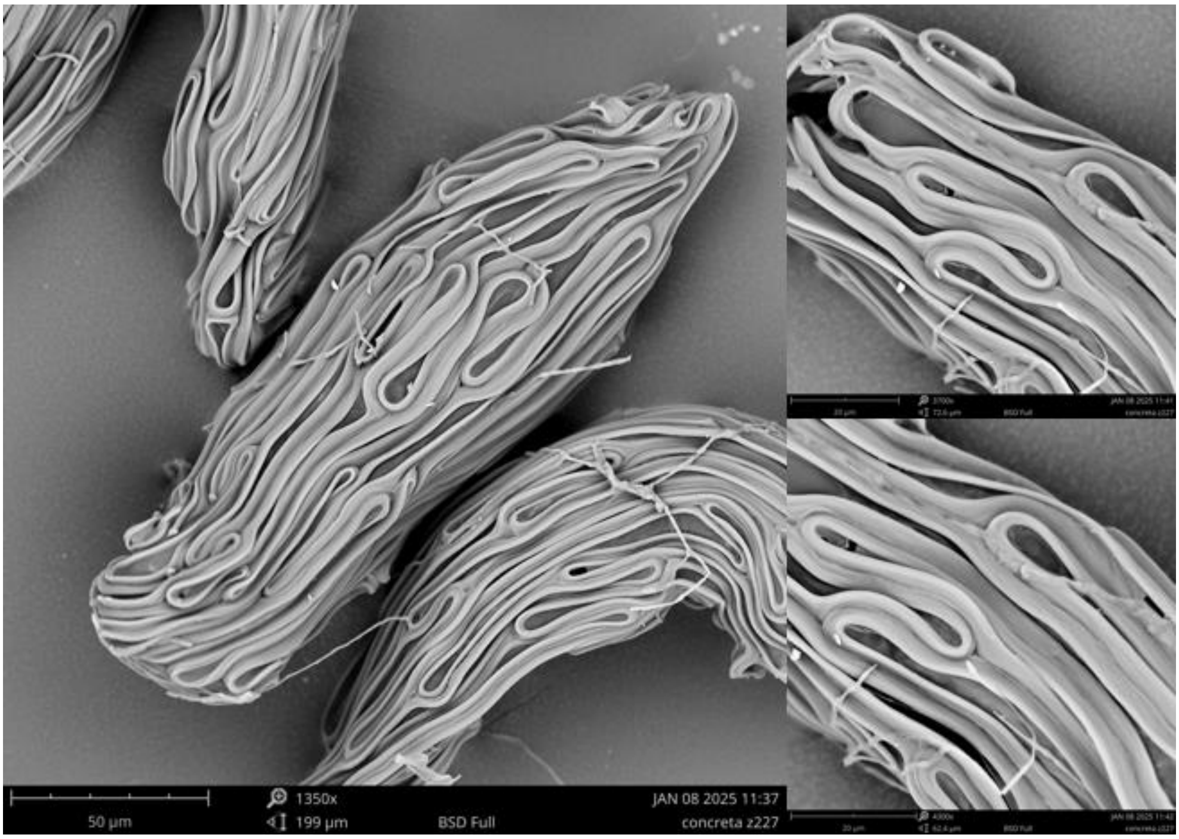

Fig.S57. *Polystachya concreta* (Jacq.) Garay & H.R.Sweet (P. con39)

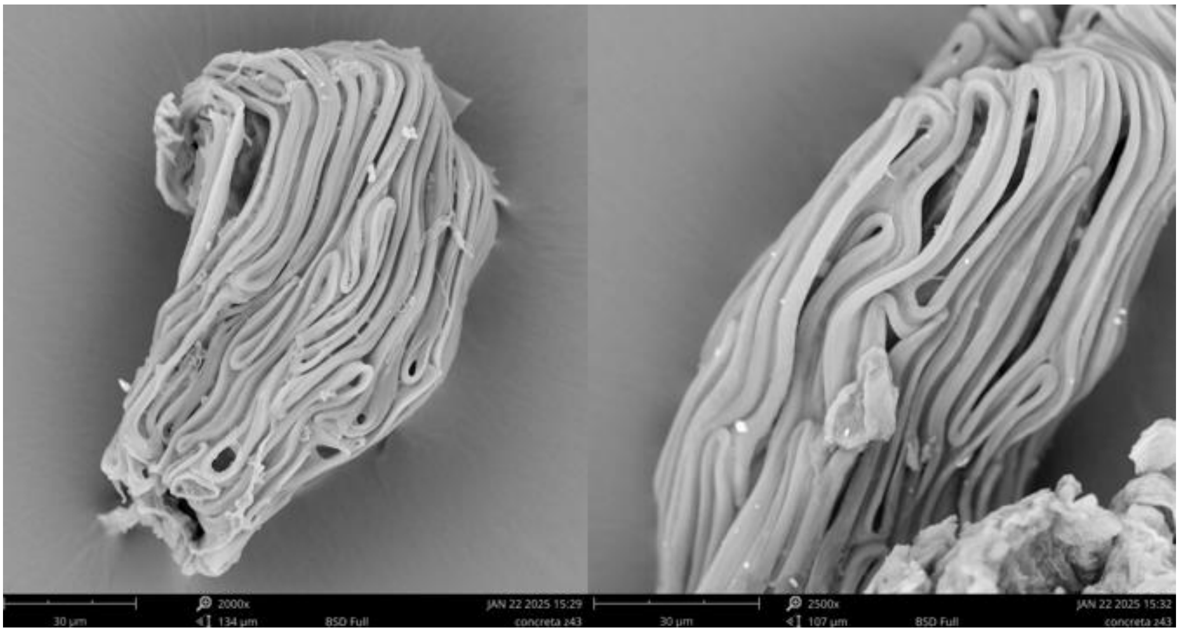

Fig.S58. *Polystachya concreta* (Jacq.) Garay & H.R.Sweet (P. con40)

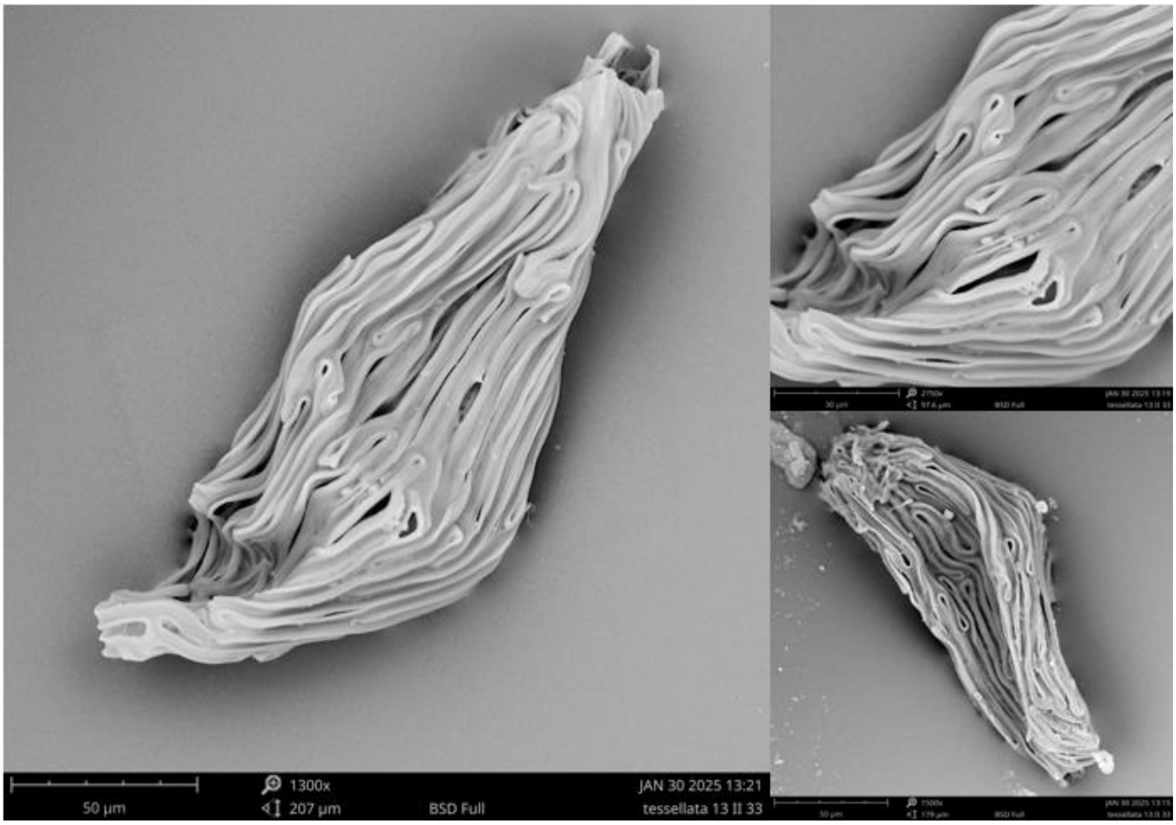

Fig.S59. *Polystachya concreta* (Jacq.) Garay & H.R.Sweet (P. con41)

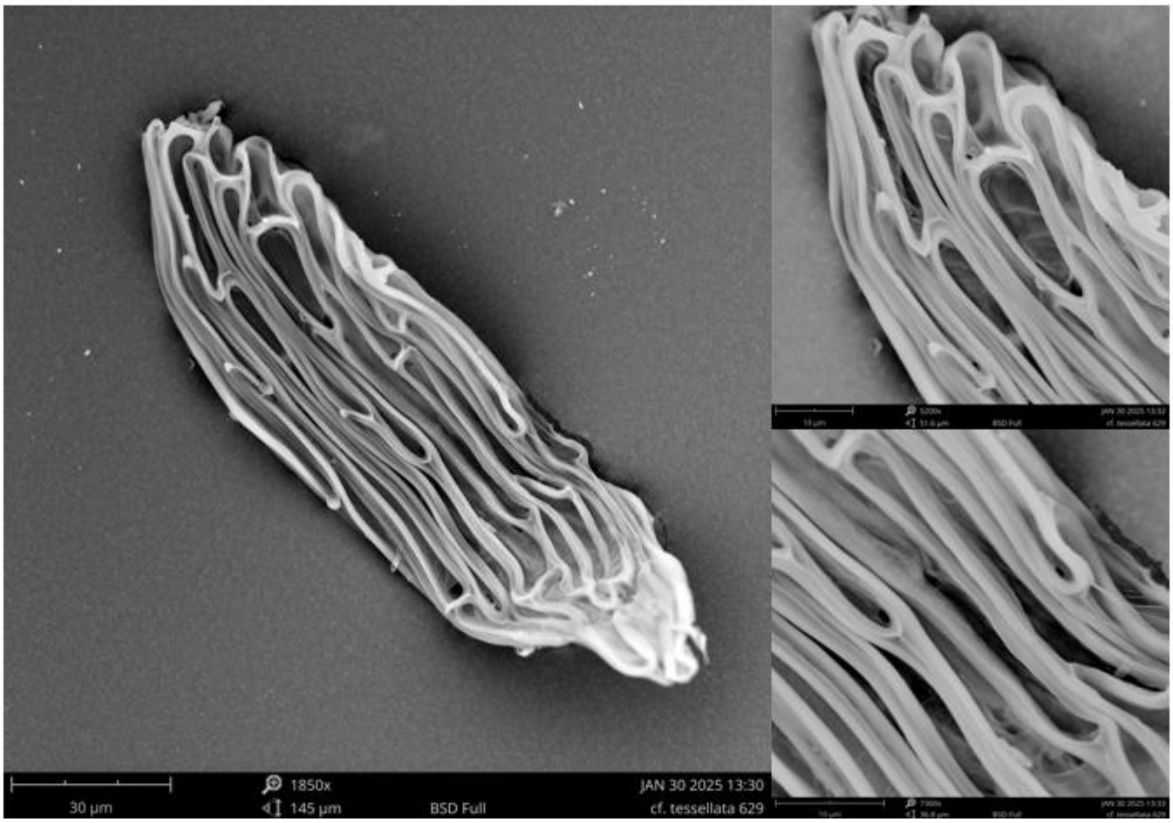

Fig.S60. *Polystachya concreta* (Jacq.) Garay & H.R.Sweet (P. con42)

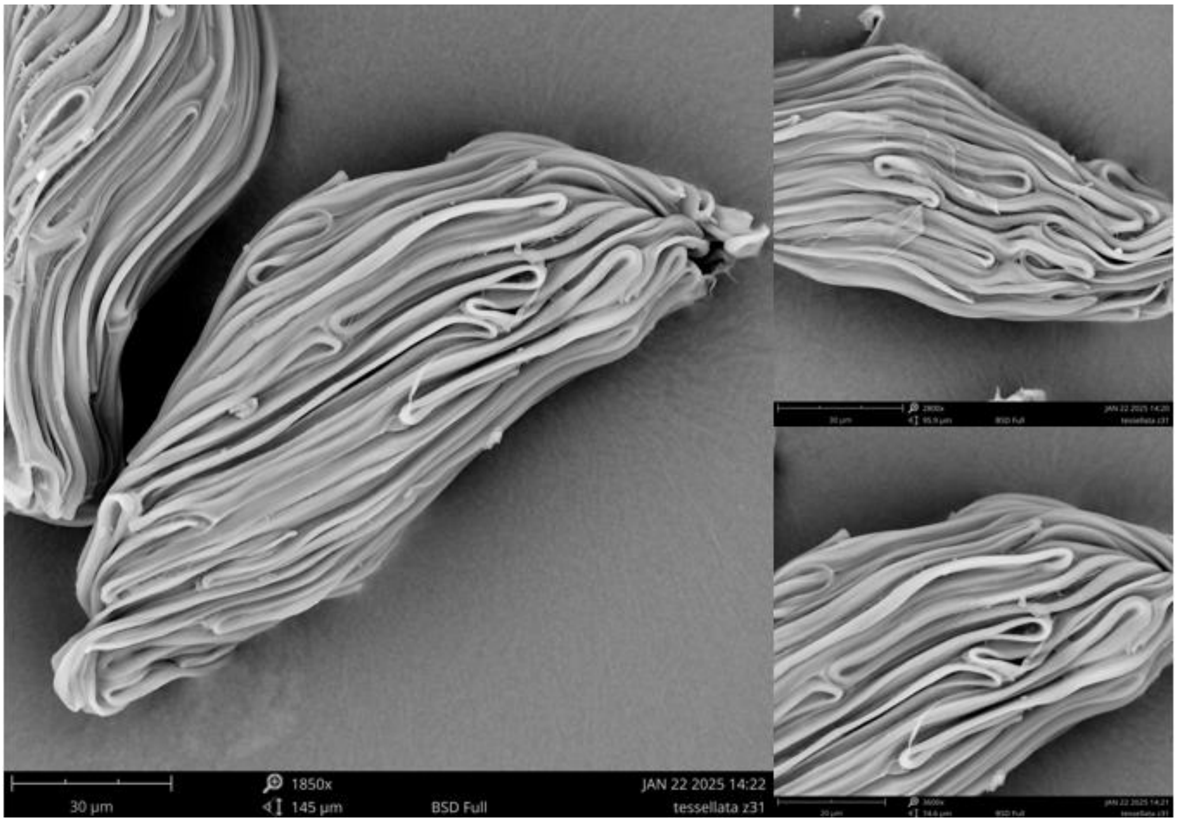

Fig.S61. *Polystachya concreta* (Jacq.) Garay & H.R.Sweet (P. con44)

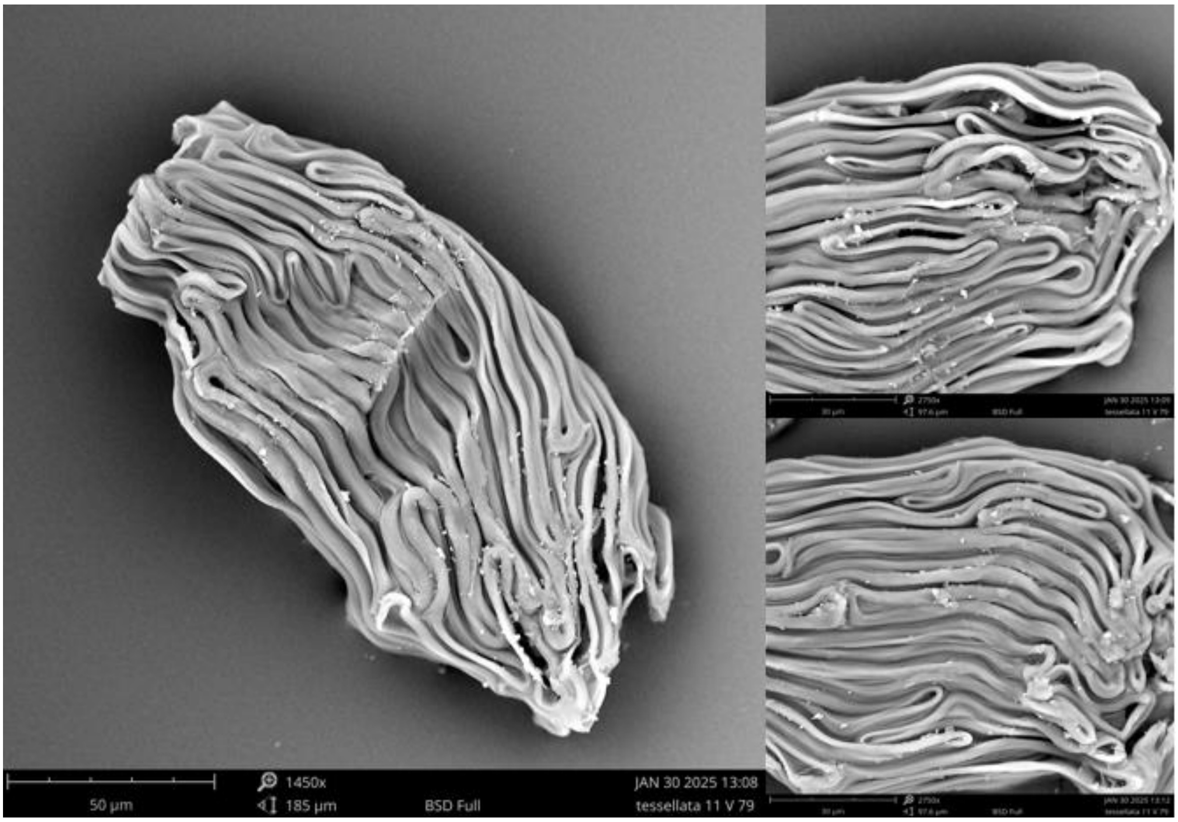

Fig.S62. *Polystachya concreta* (Jacq.) Garay & H.R.Sweet (P. con45)

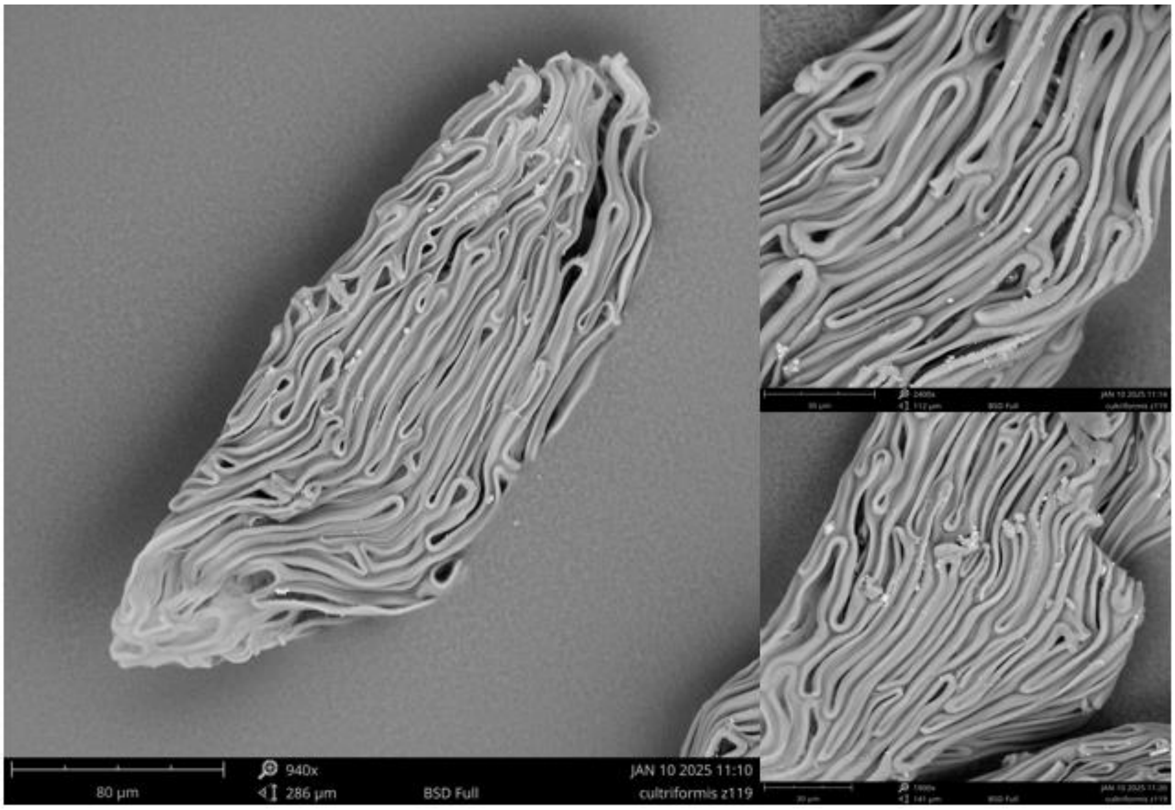

Fig.S63. *Polystachya cultriformis* (Thouars) Spreng. (P. cul5)

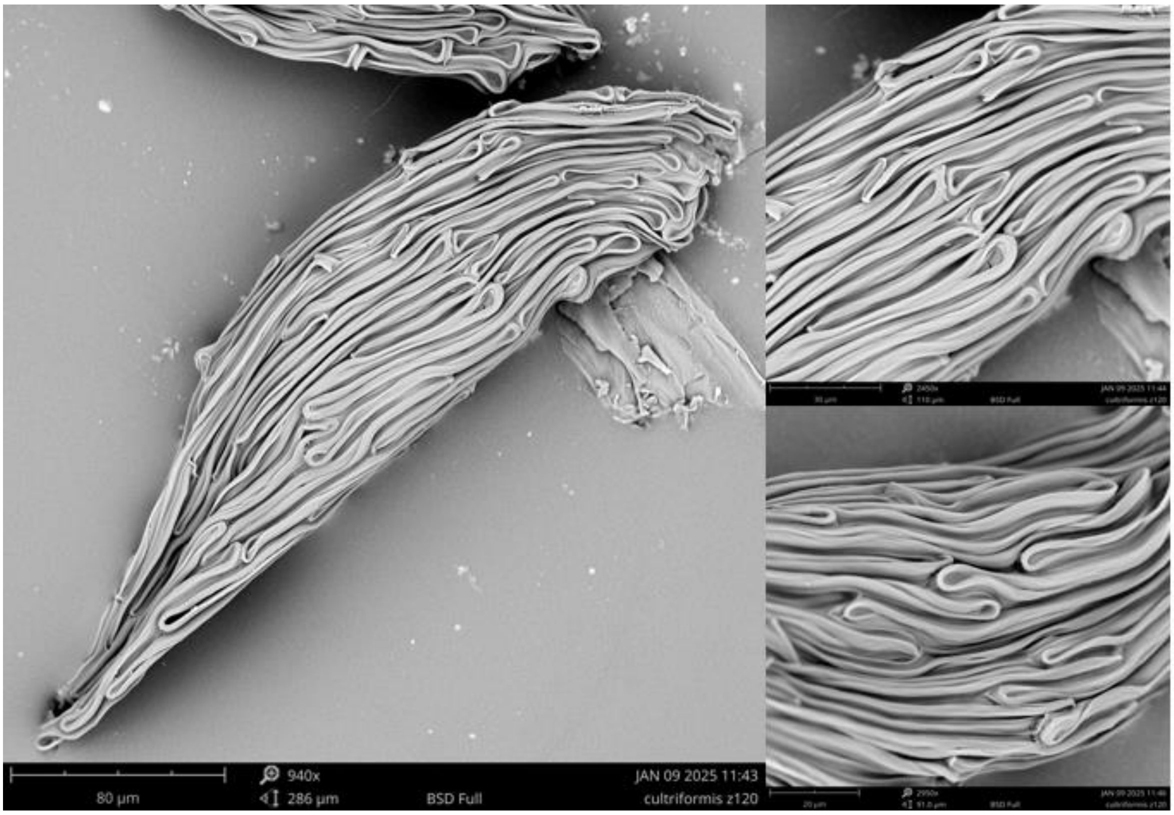

Fig.S64. *Polystachya cultriformis* (Thouars) Spreng. (P. cul6)

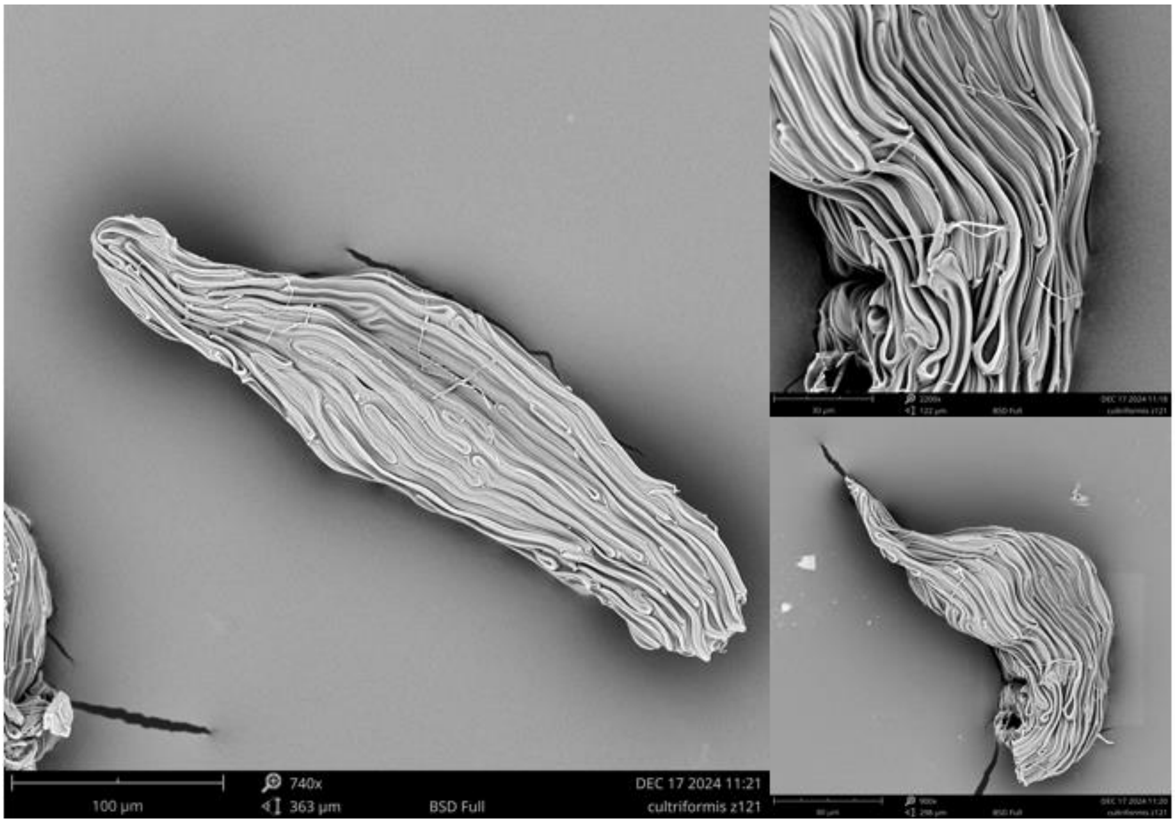

Fig.S65. *Polystachya cultriformis* (Thouars) Spreng. (P. cul7)

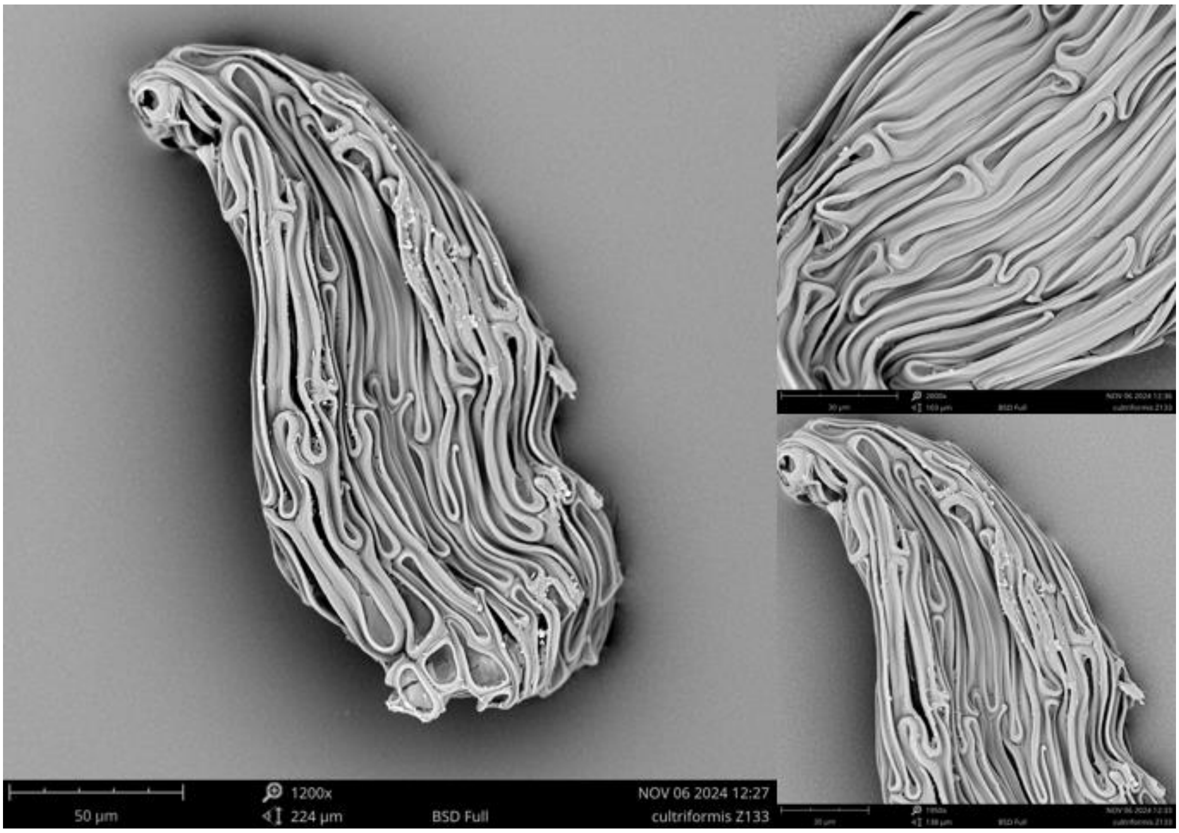

Fig.S66. *Polystachya cultriformis* (Thouars) Spreng. (P. cul8)

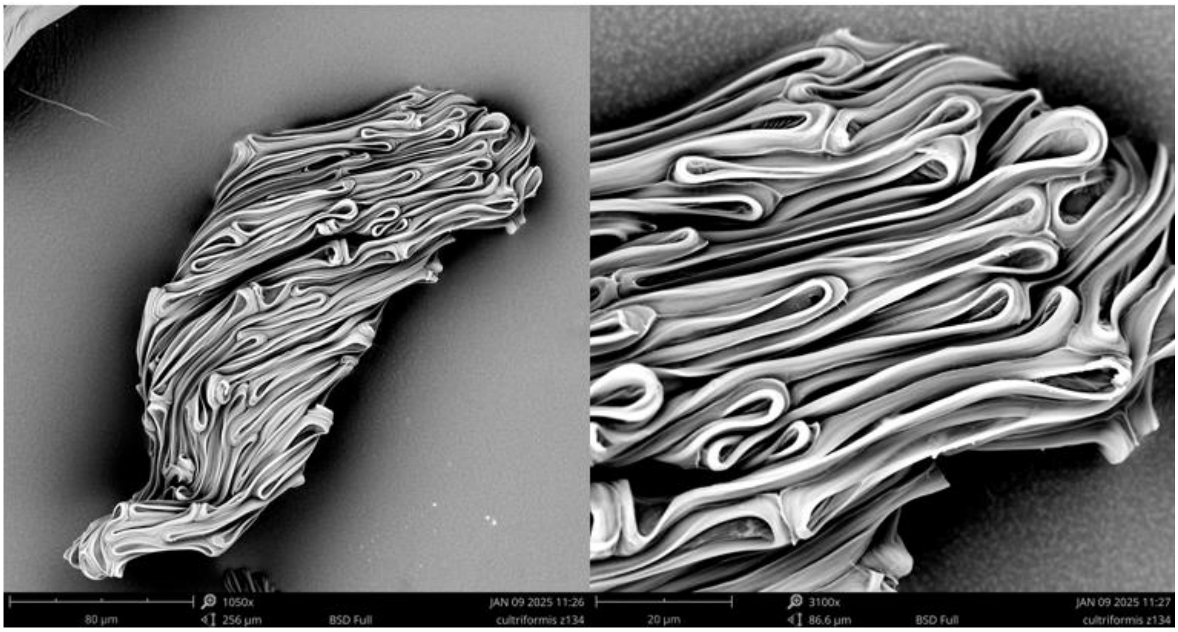

Fig.S67. *Polystachya cultriformis* (Thouars) Spreng. (P. cul9)

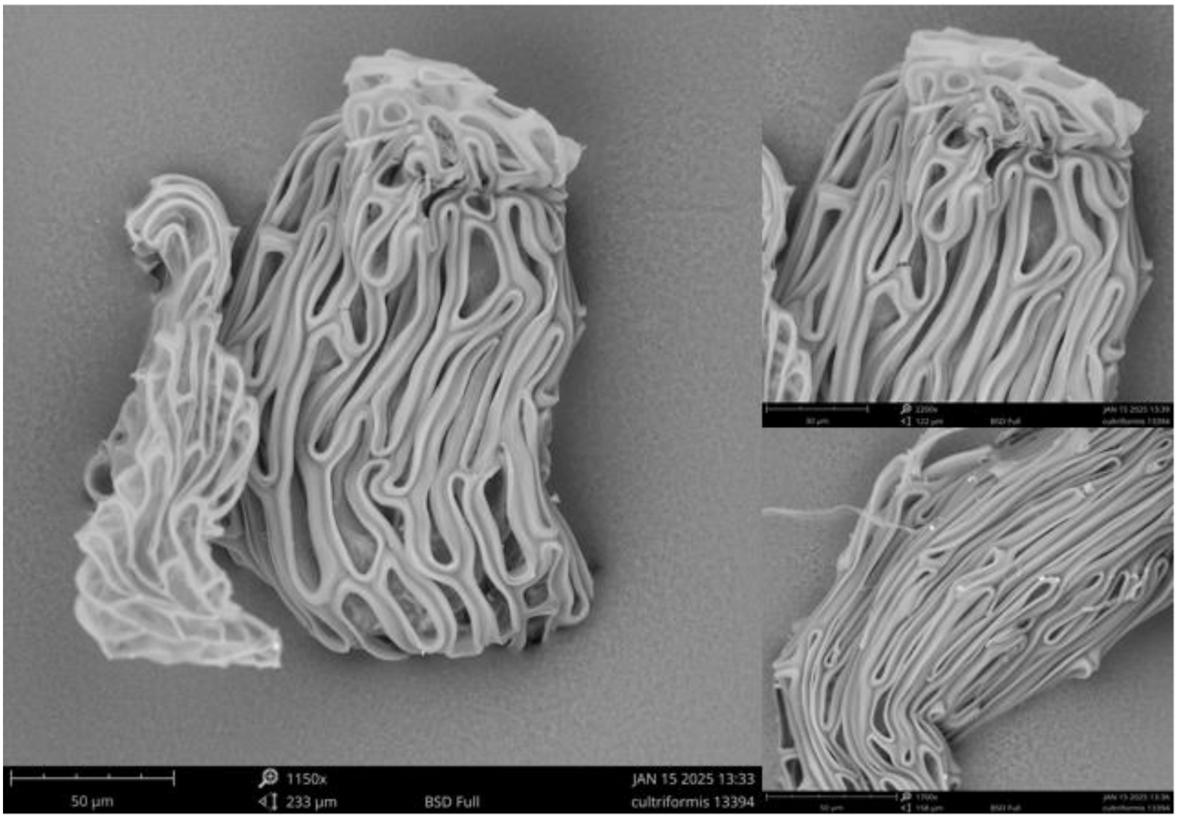

Fig.S68. *Polystachya cultriformis* (Thouars) Spreng. (P. cul12)

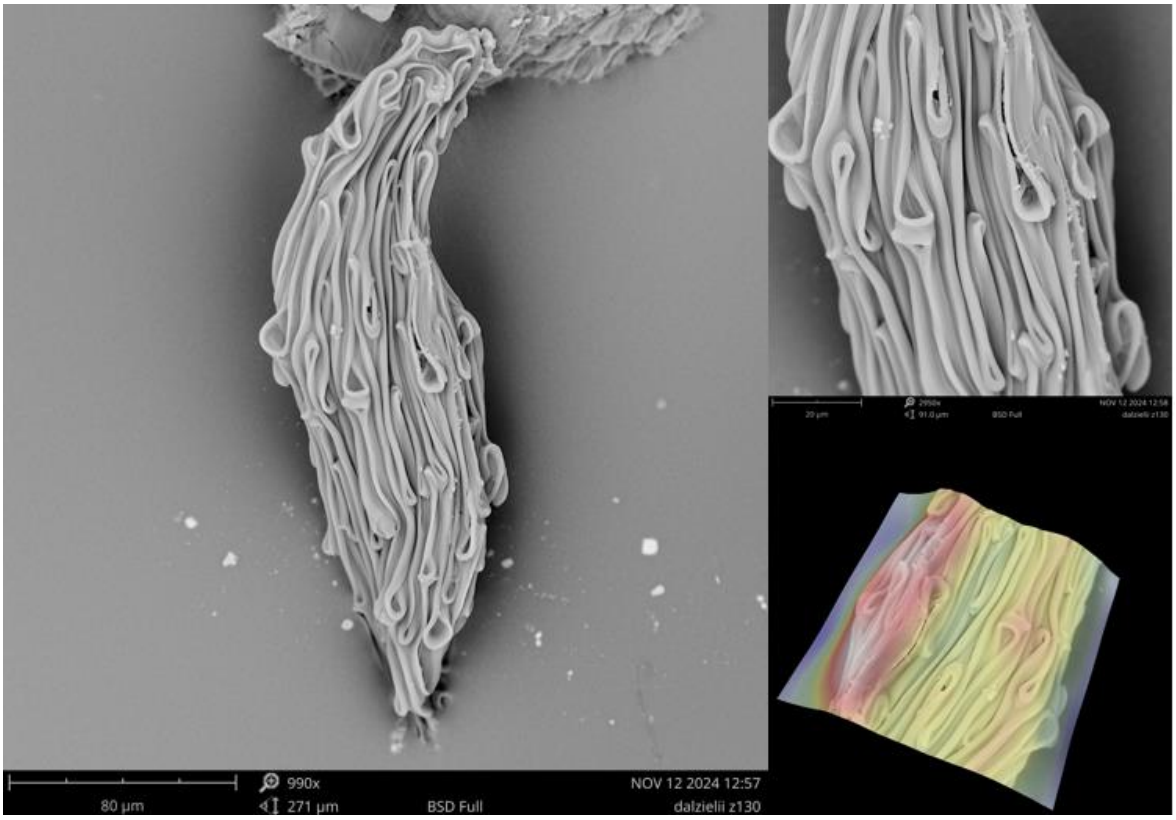

Fig.S69. *Polystachya dalzielii* Summerh. (P. dal)

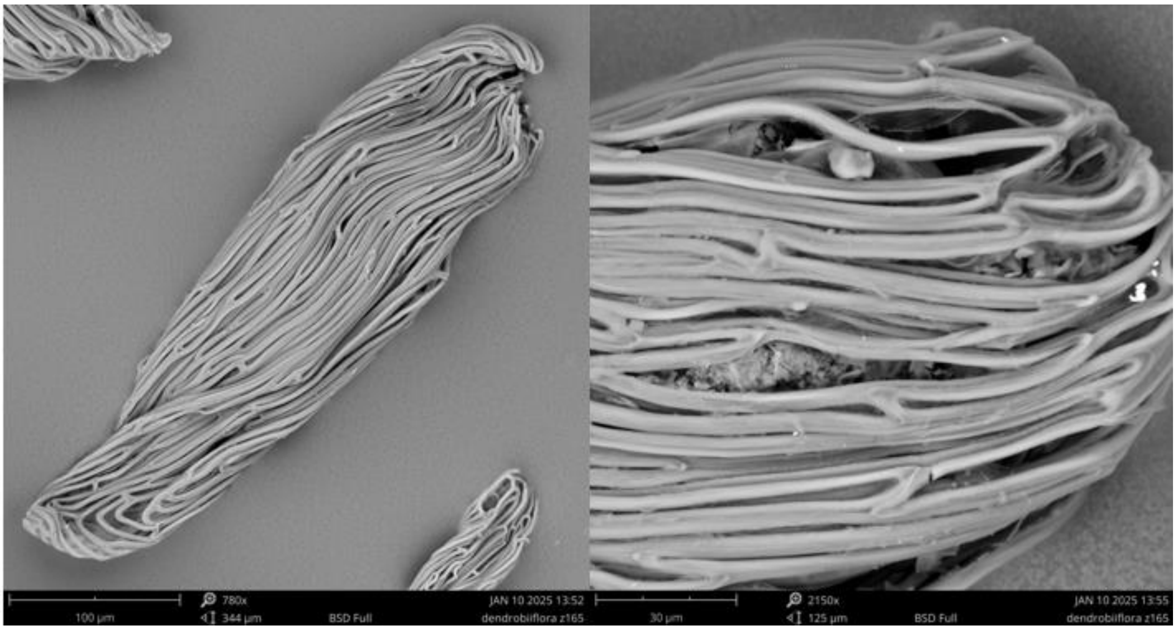

Fig.S70. *Polystachya dendrobiiflora* Rchb.f. (P. den)

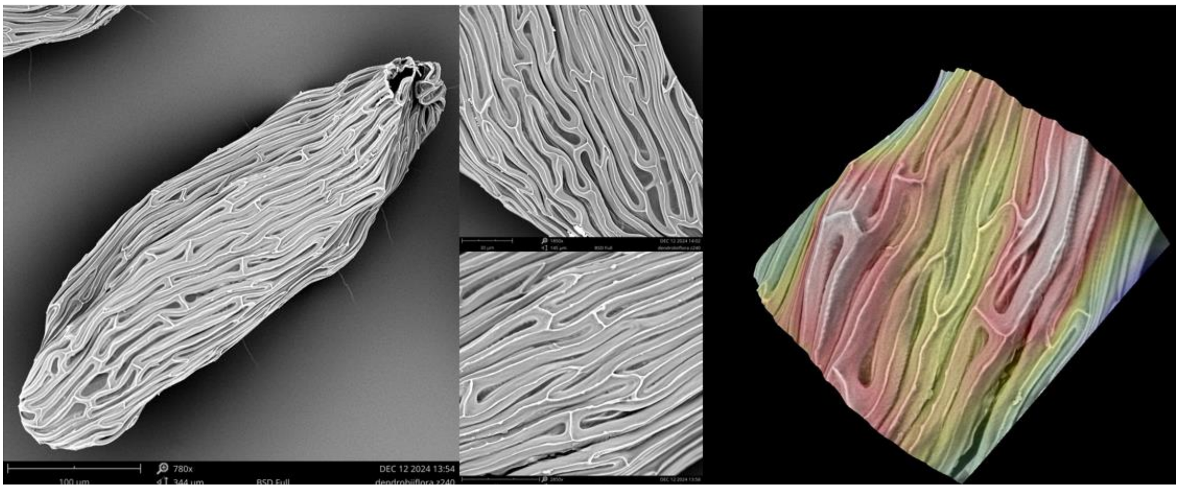

Fig.S71. *Polystachya dendrobiiflora* Rchb.f. (P. den2)

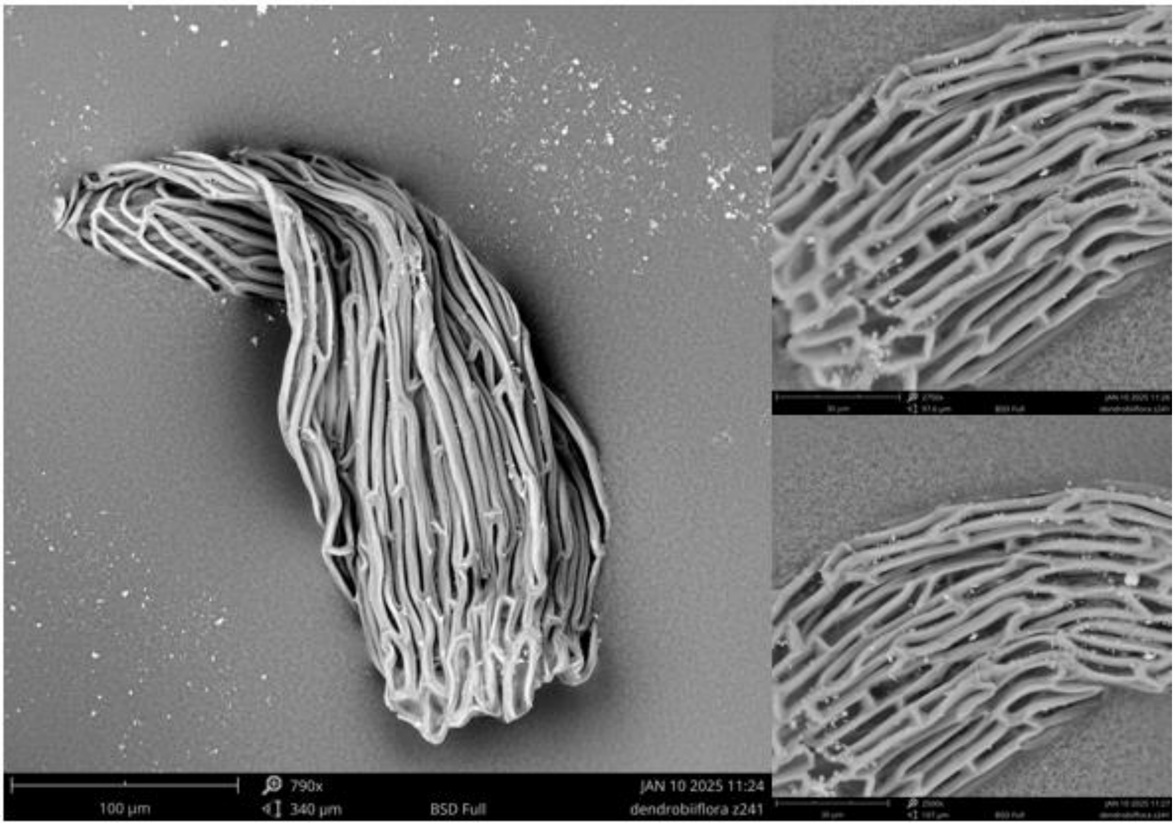

Fig.S72. *Polystachya dendrobiiflora* Rchb.f. (P. den3)

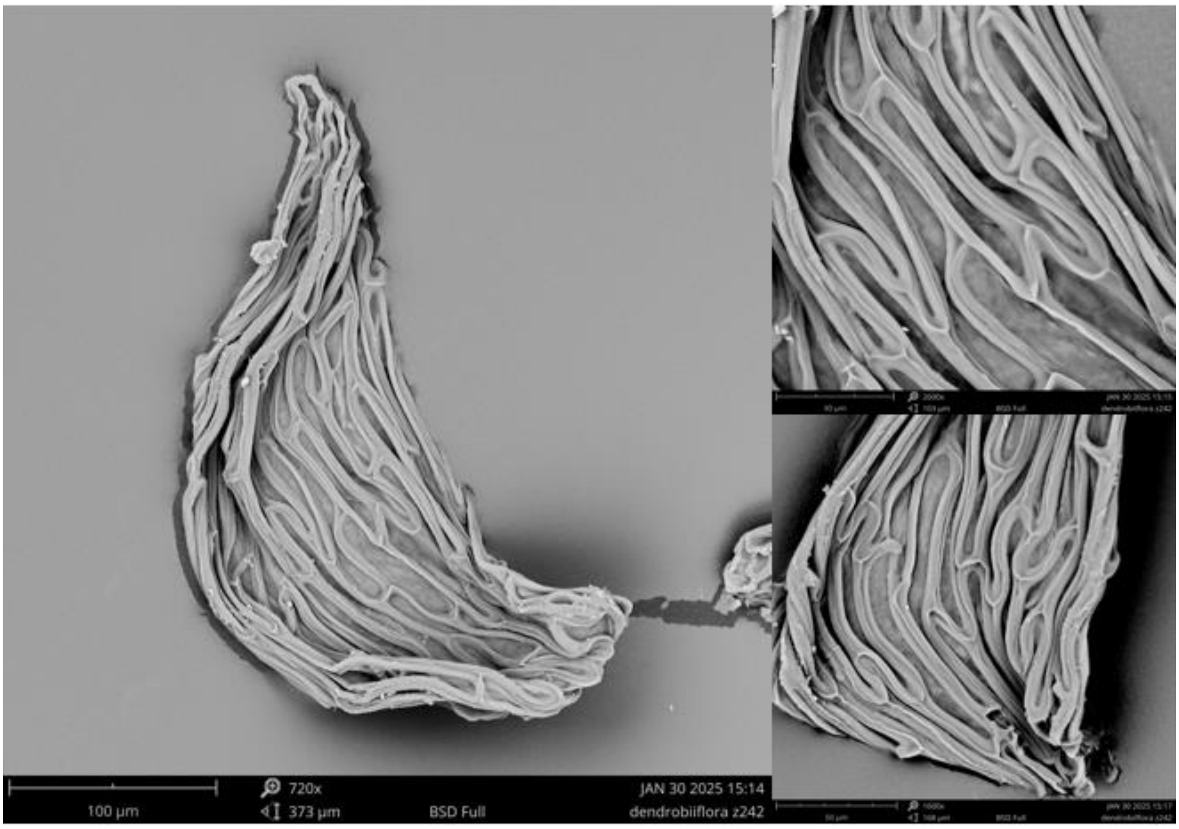

Fig.S73. *Polystachya dendrobiiflora* Rchb.f. (P. den4)

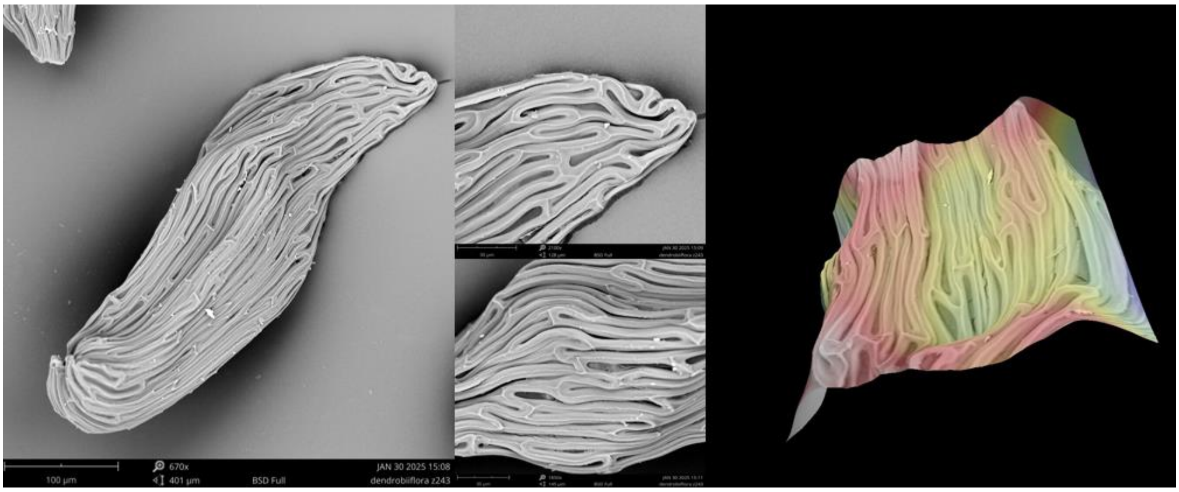

Fig.S74. *Polystachya dendrobiiflora* Rchb.f. (P. den5)

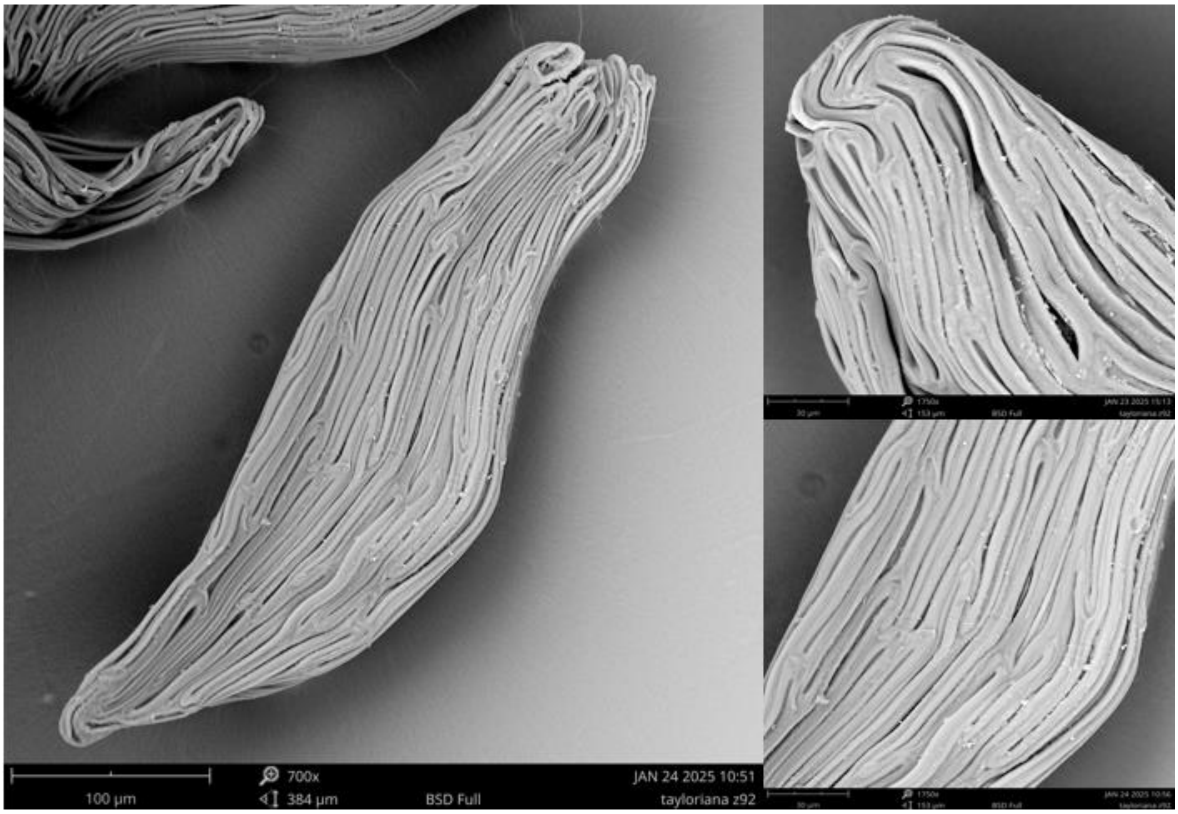

Fig.S75. *Polystachya dendrobiiflora* Rchb.f. (P. den6)

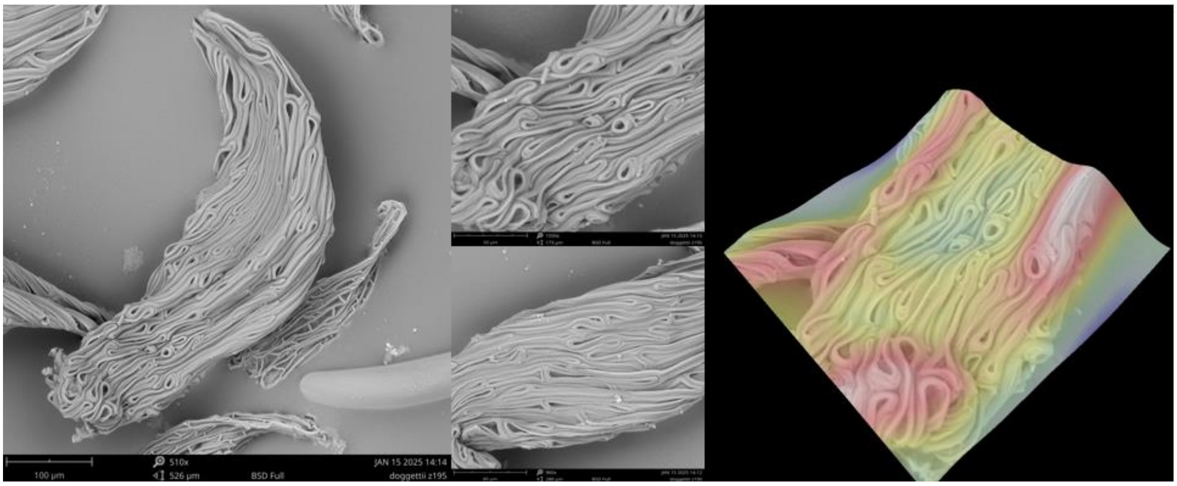

Fig.S76. *Polystachya doggettii* Rendle & Rolfe (P. dog)

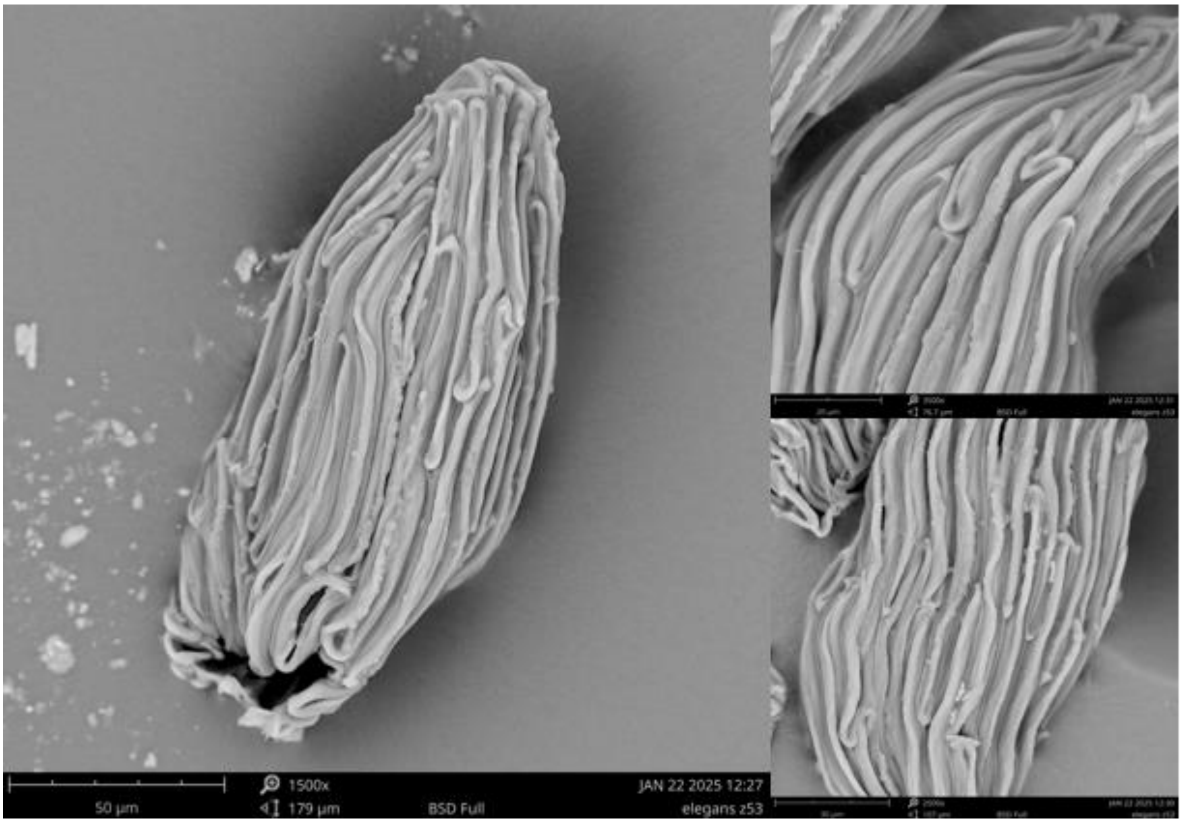

Fig.S77. *Polystachya elegans* Rchb.f. (P. ele)

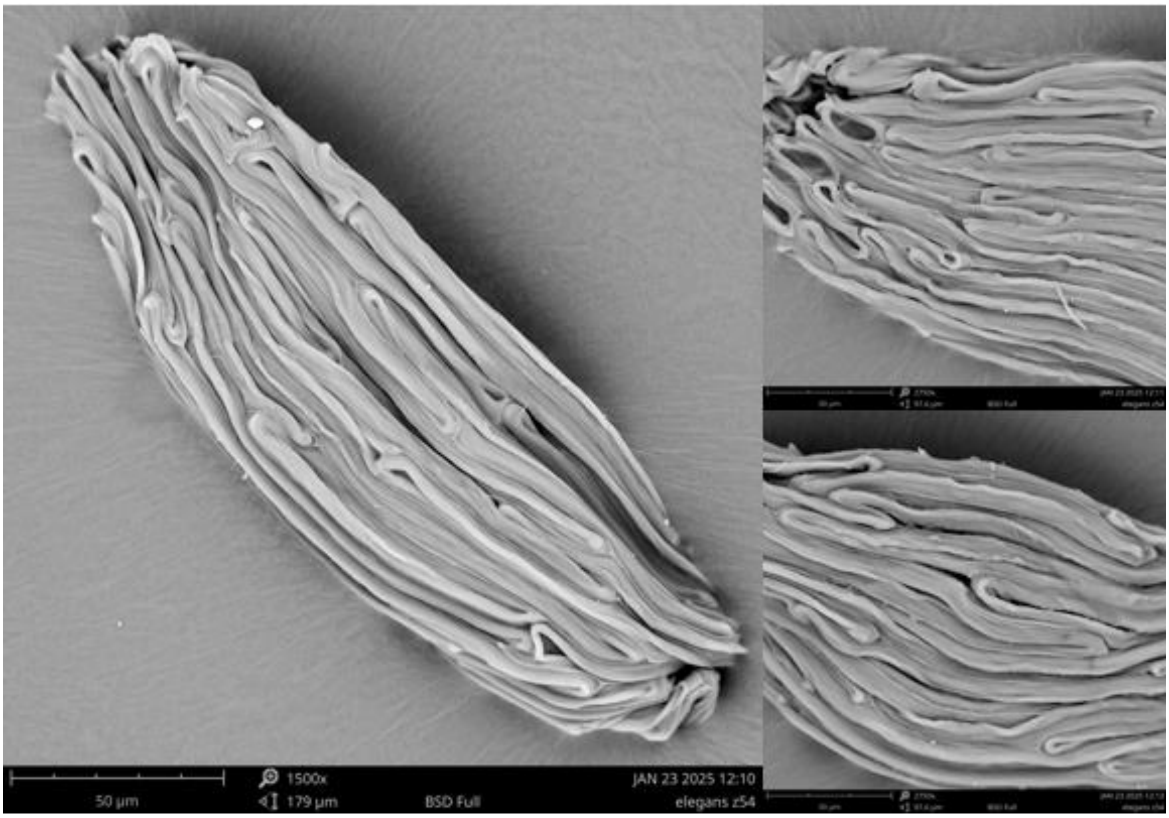

Fig.S78. *Polystachya elegans* Rchb.f. (P. ele2)

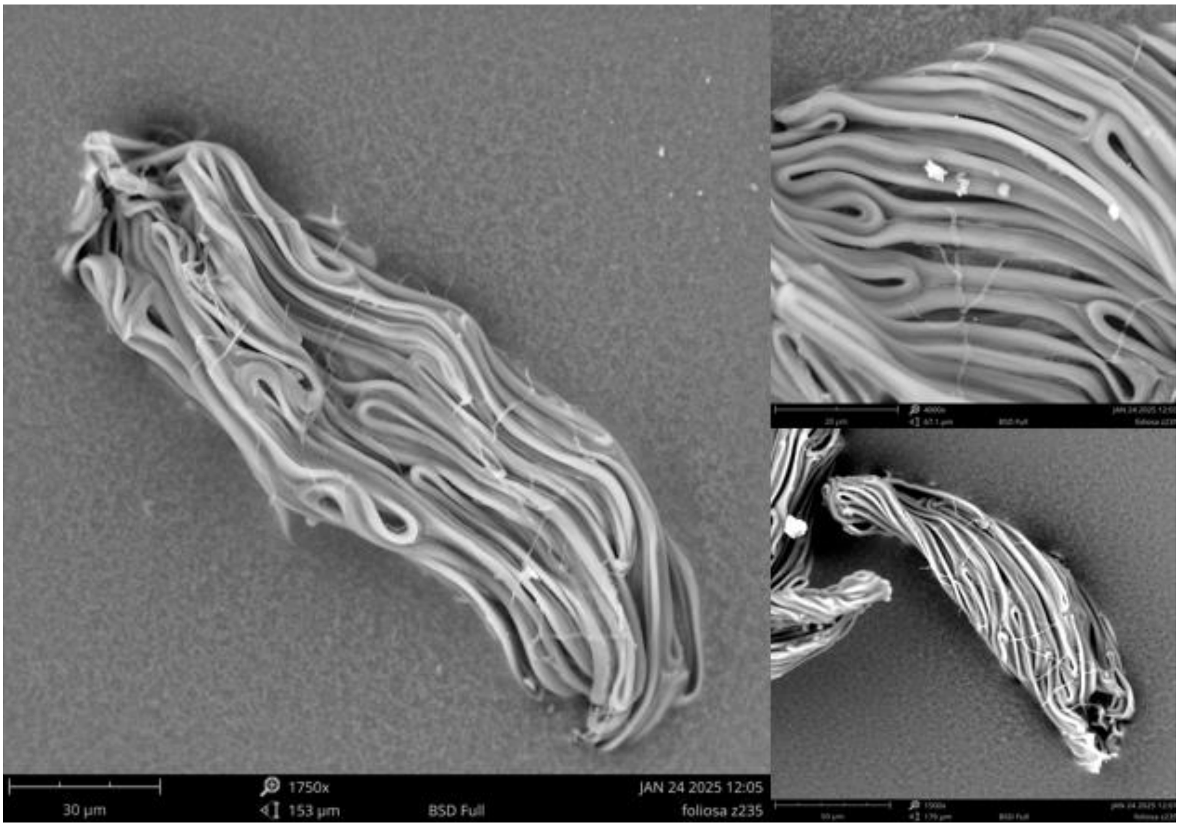

Fig.S79. *Polystachya foliosa* (Hook.) Rchb.f. (P. fol)

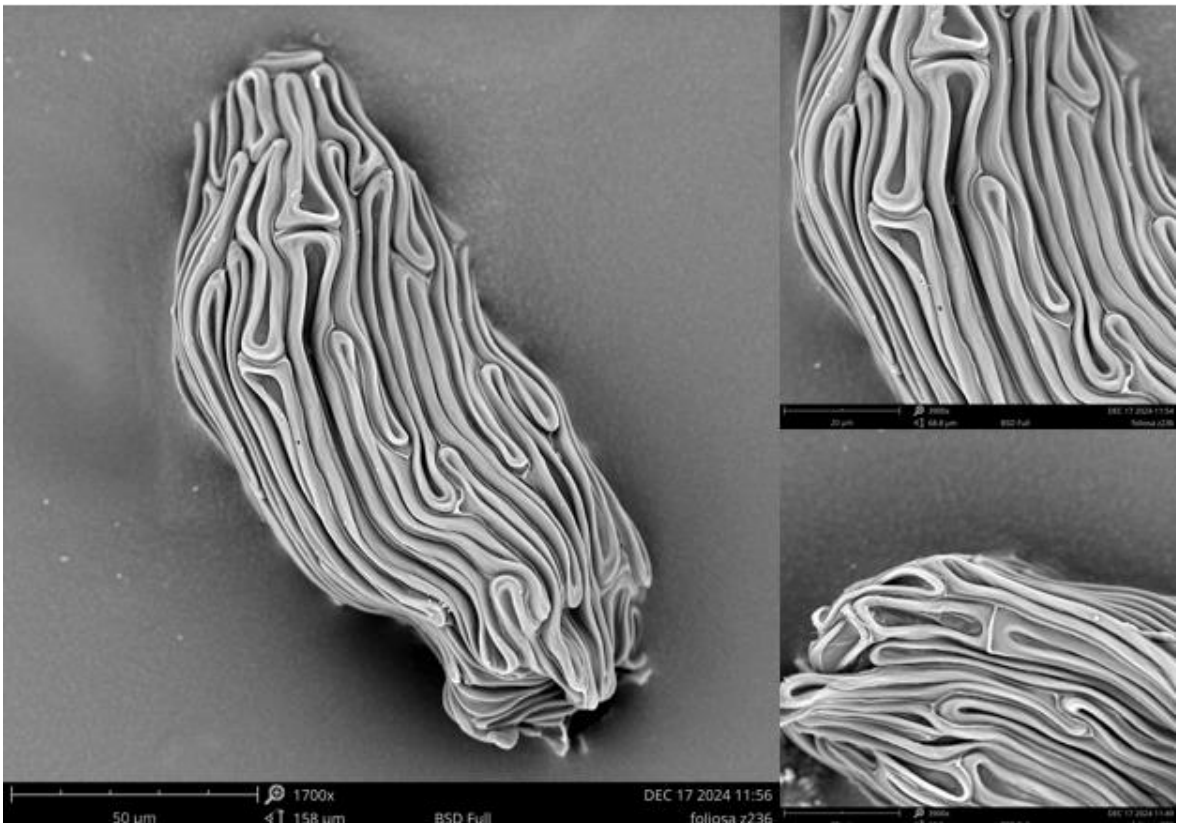

Fig.S80. *Polystachya foliosa* (Hook.) Rchb.f. (P. fol2)

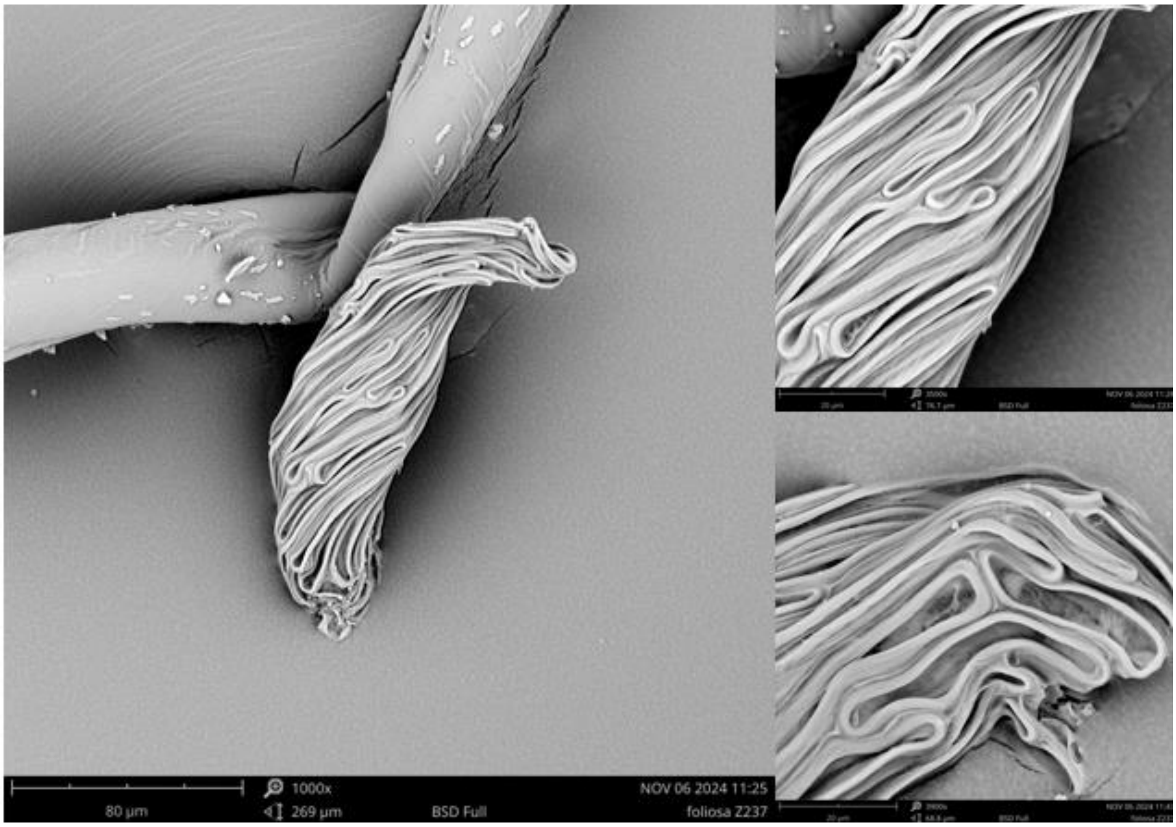

Fig.S81. *Polystachya foliosa* (Hook.) Rchb.f. (P. fol3)

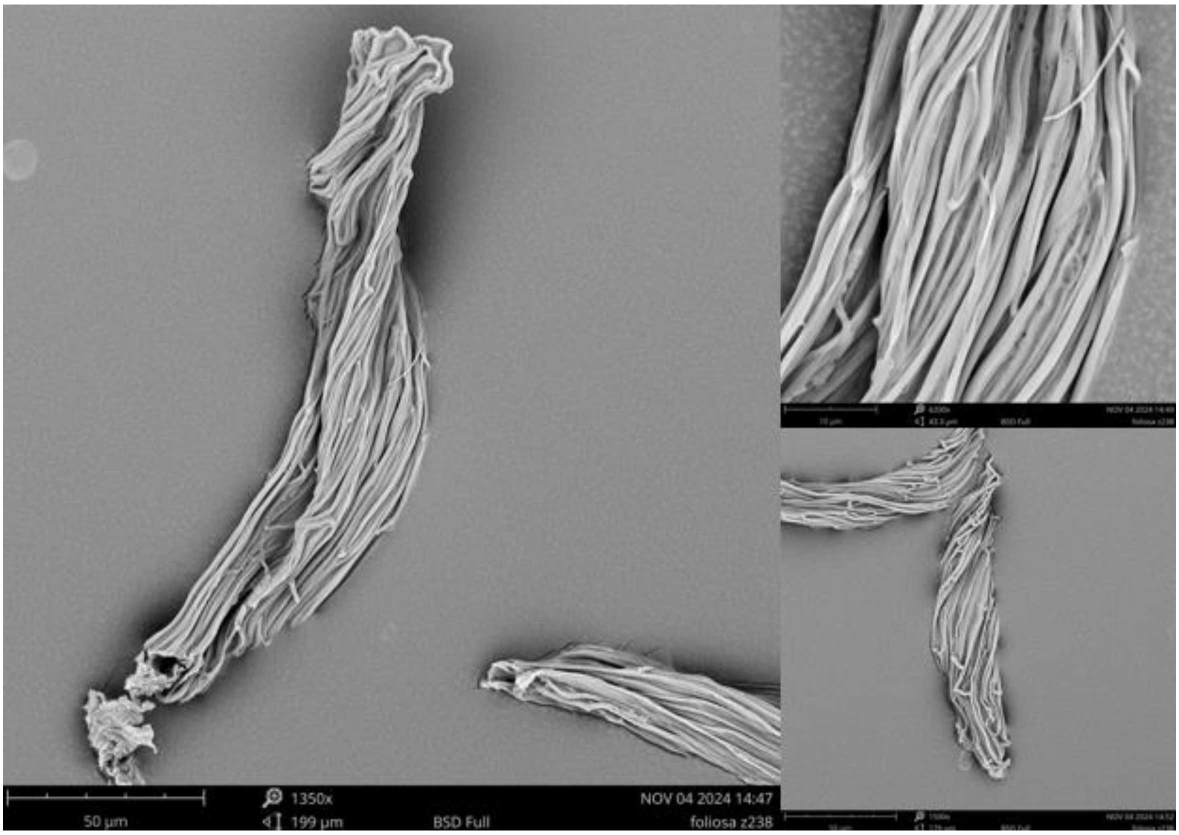

Fig.S82. *Polystachya foliosa* (Hook.) Rchb.f. (P. fol4)

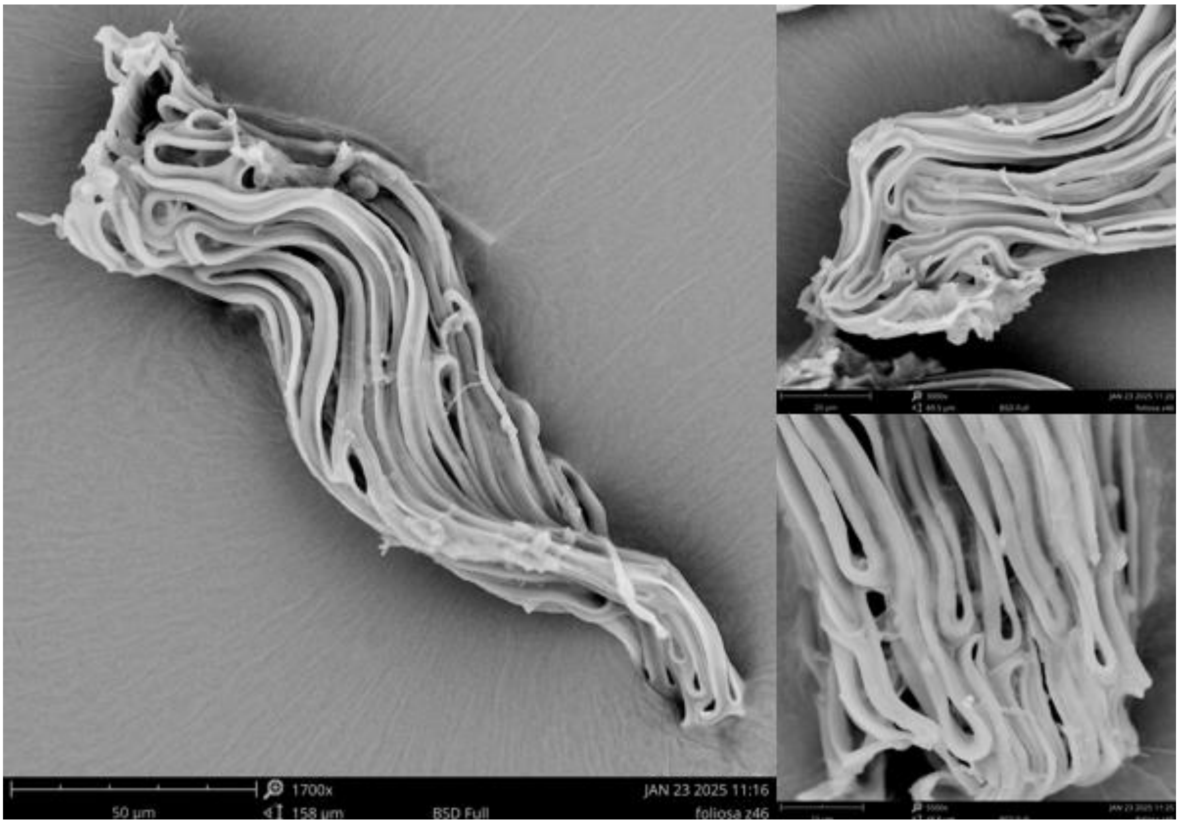

Fig.S83. *Polystachya foliosa* (Hook.) Rchb.f. (P. fol6)

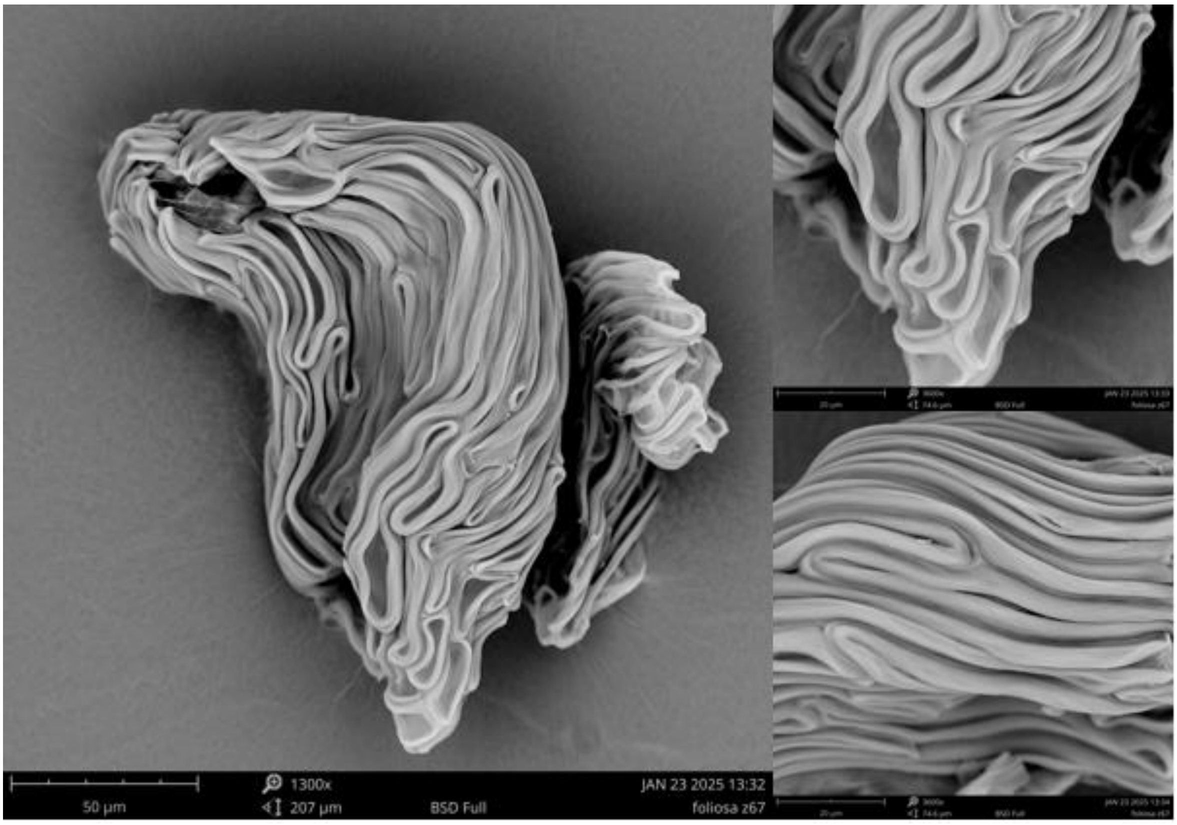

Fig.S84. *Polystachya foliosa* (Hook.) Rchb.f. (P. fol7)

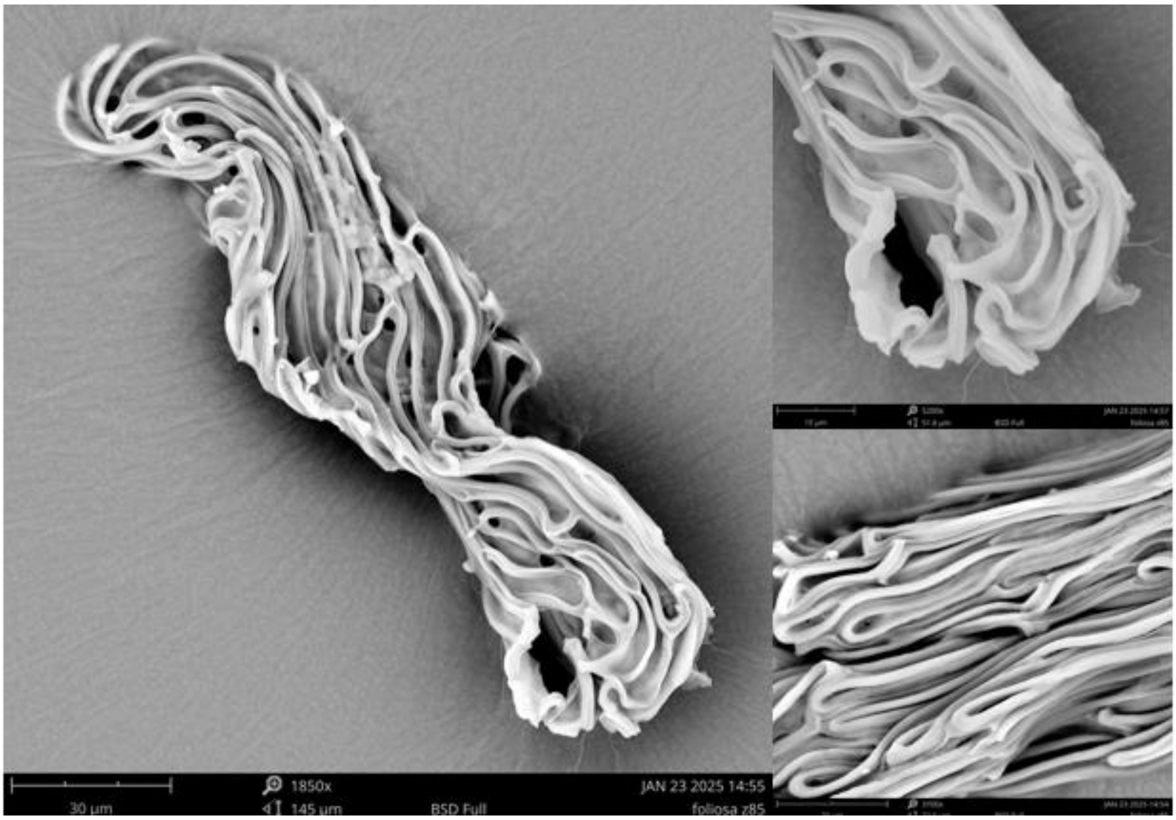

Fig.S85. *Polystachya foliosa* (Hook.) Rchb.f. (P. fol8)

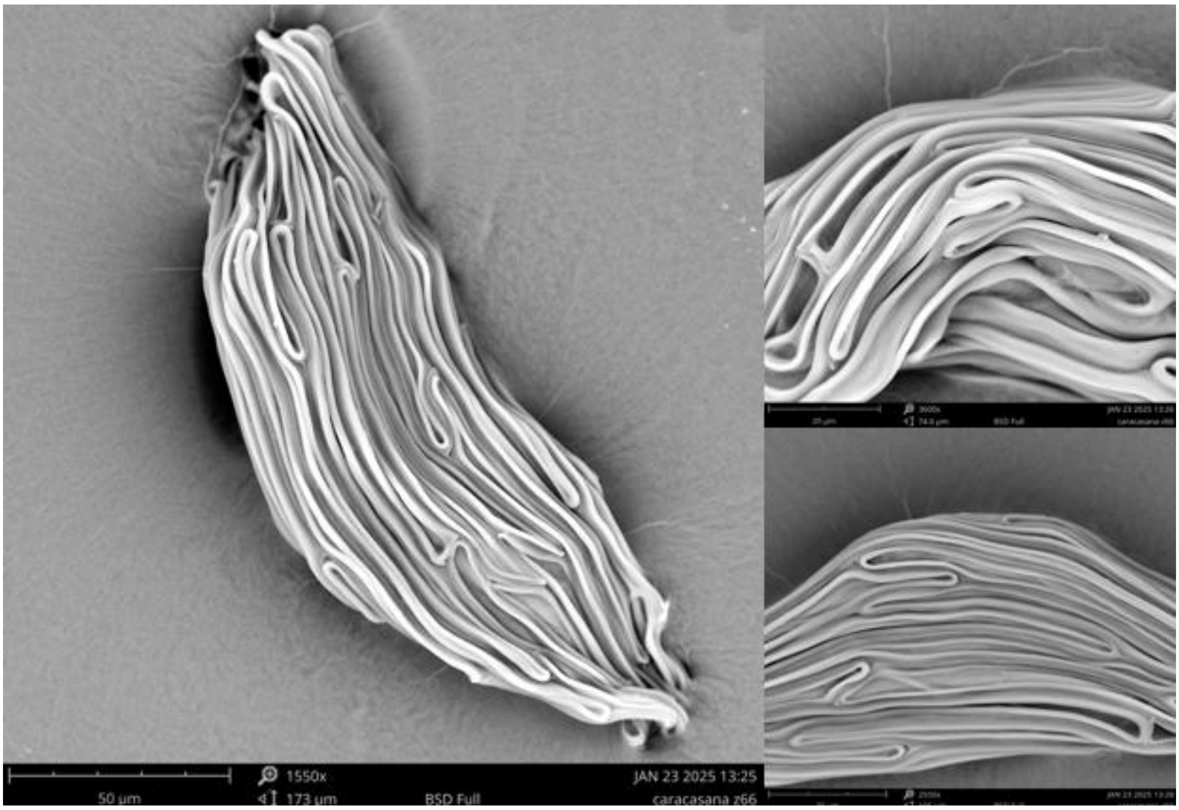

Fig.S86. *Polystachya foliosa* (Hook.) Rchb.f. (P. fol9)

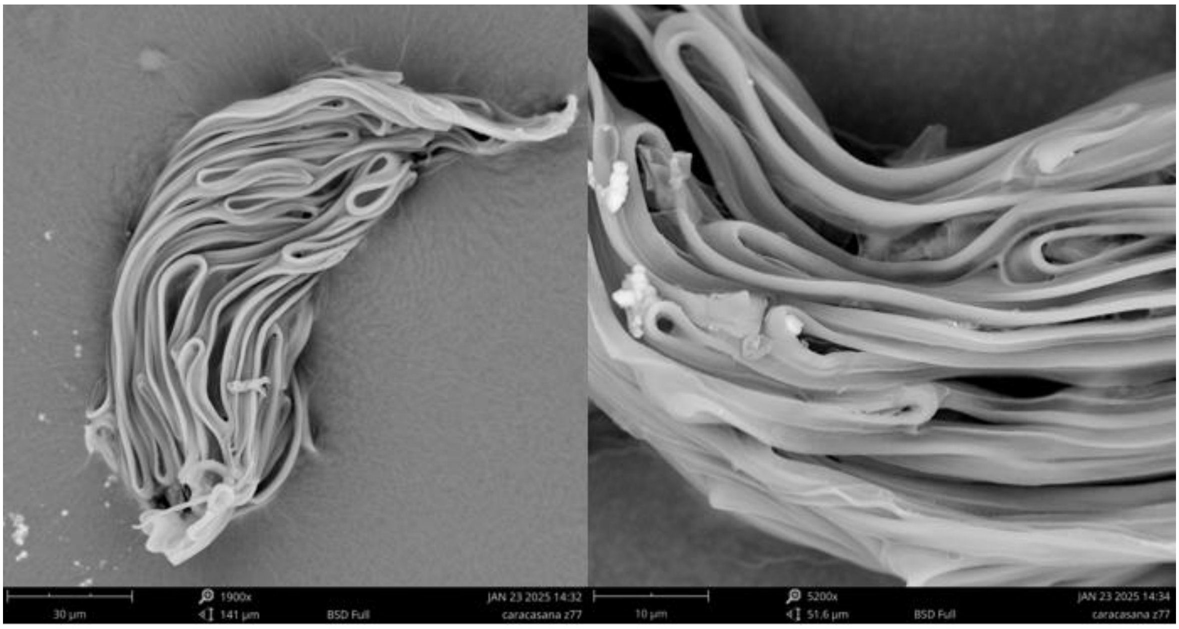

Fig.S87. *Polystachya foliosa* (Hook.) Rchb.f. (P. fol10)

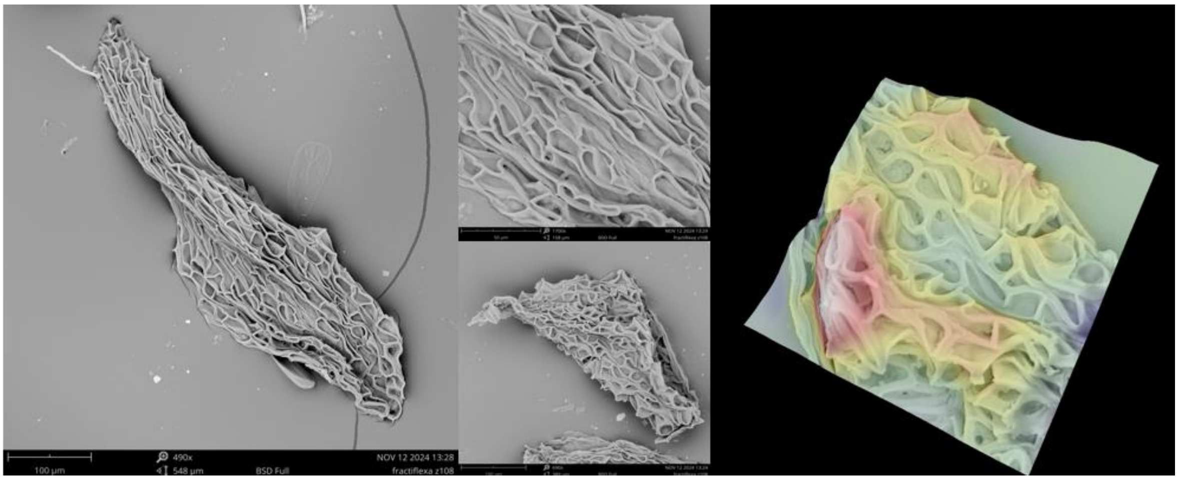

Fig.S88. *Polystachya fractiflexa* Summerh. (P. fra)

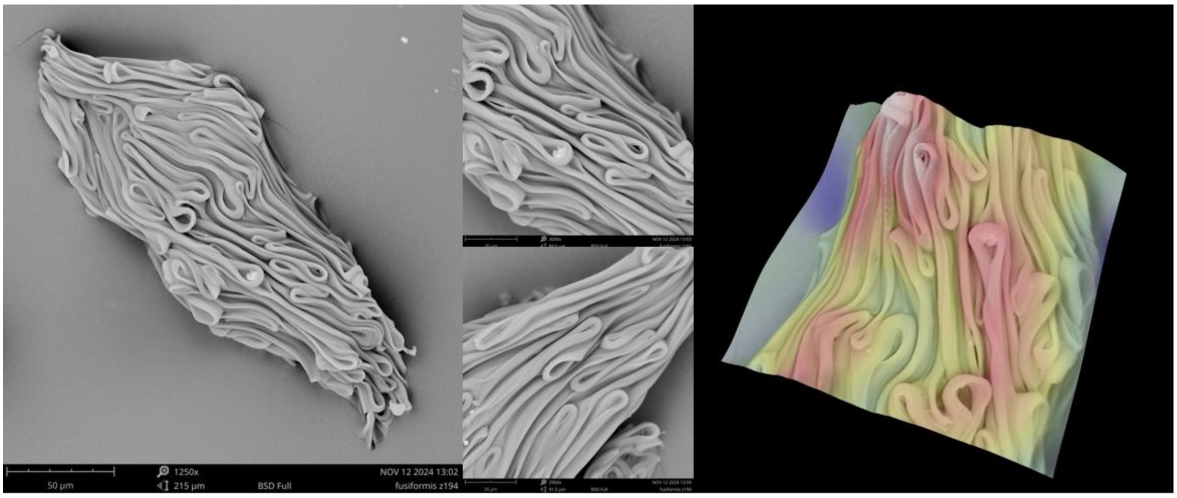

Fig.S89. *Polystachya fusiformis* (Thouars) Lindl. (P. fus)

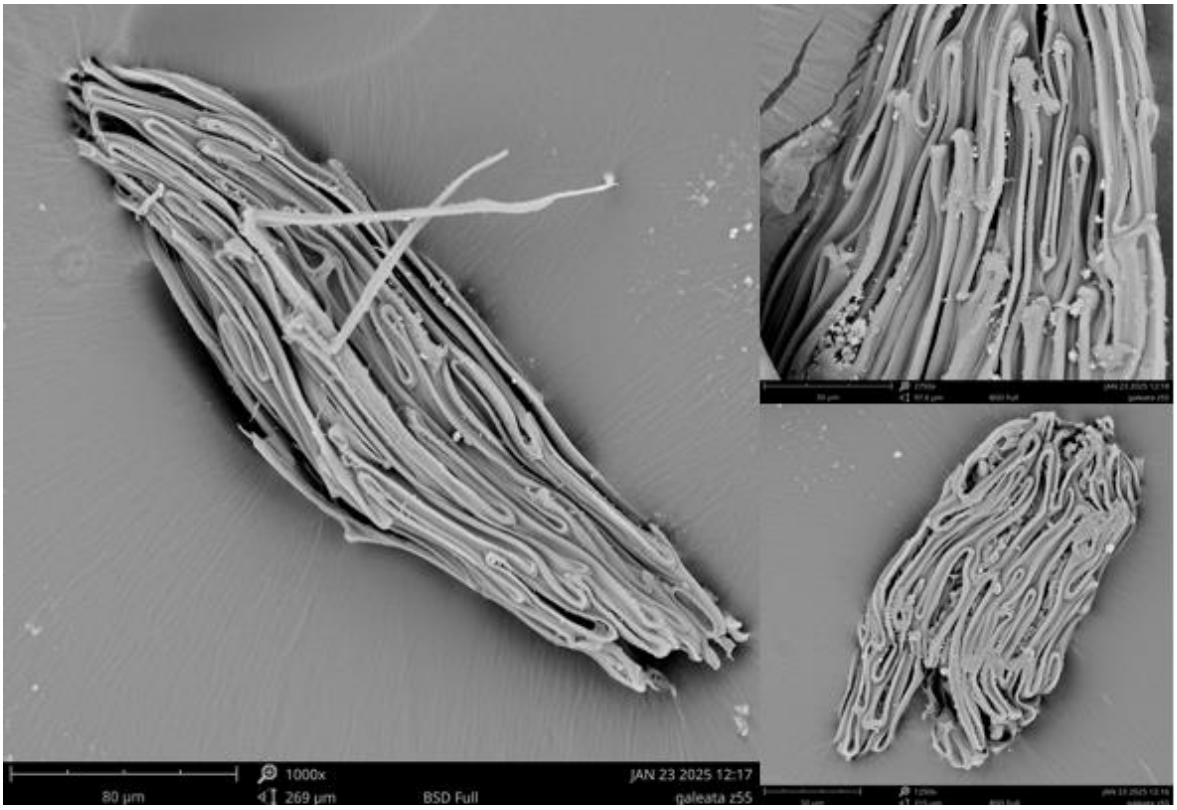

Fig.S90. *Polystachya galeata* (Sw.) Rchb.f. (P. gal)

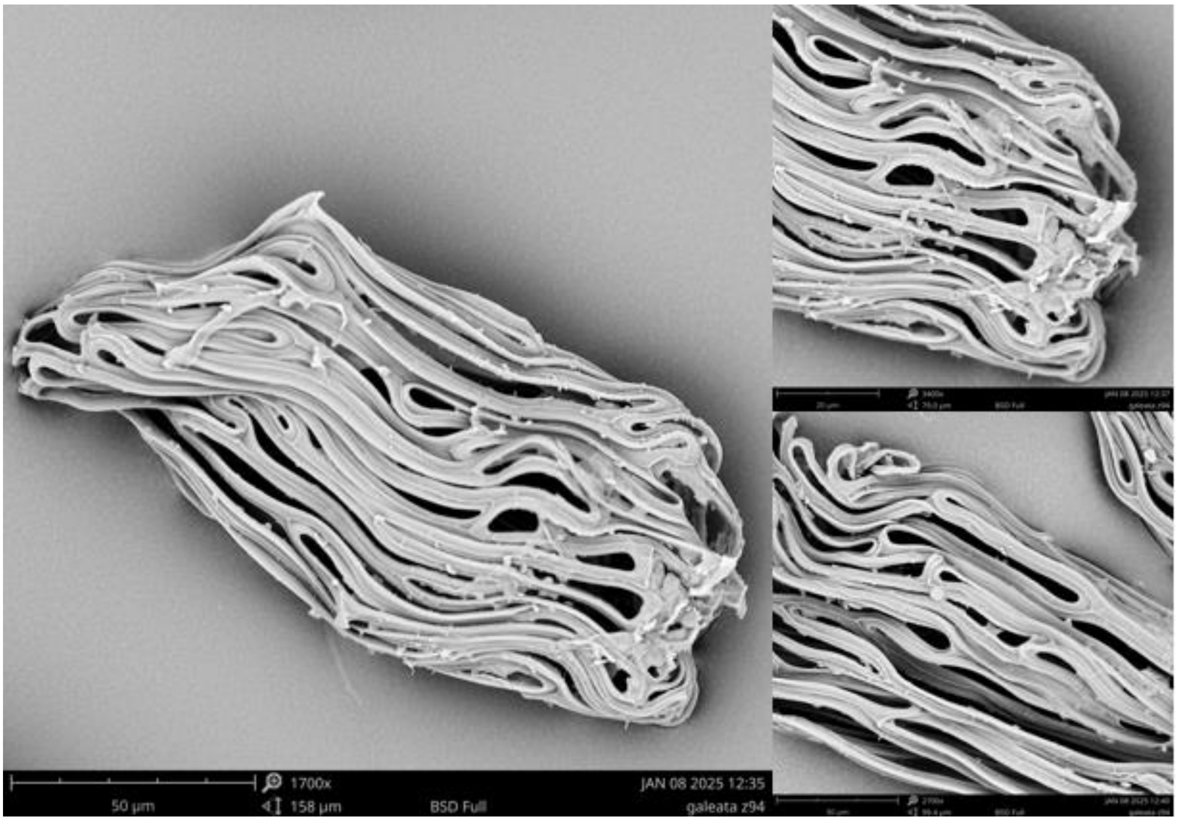

Fig.S91. *Polystachya galeata* (Sw.) Rchb.f. (P. gal2)

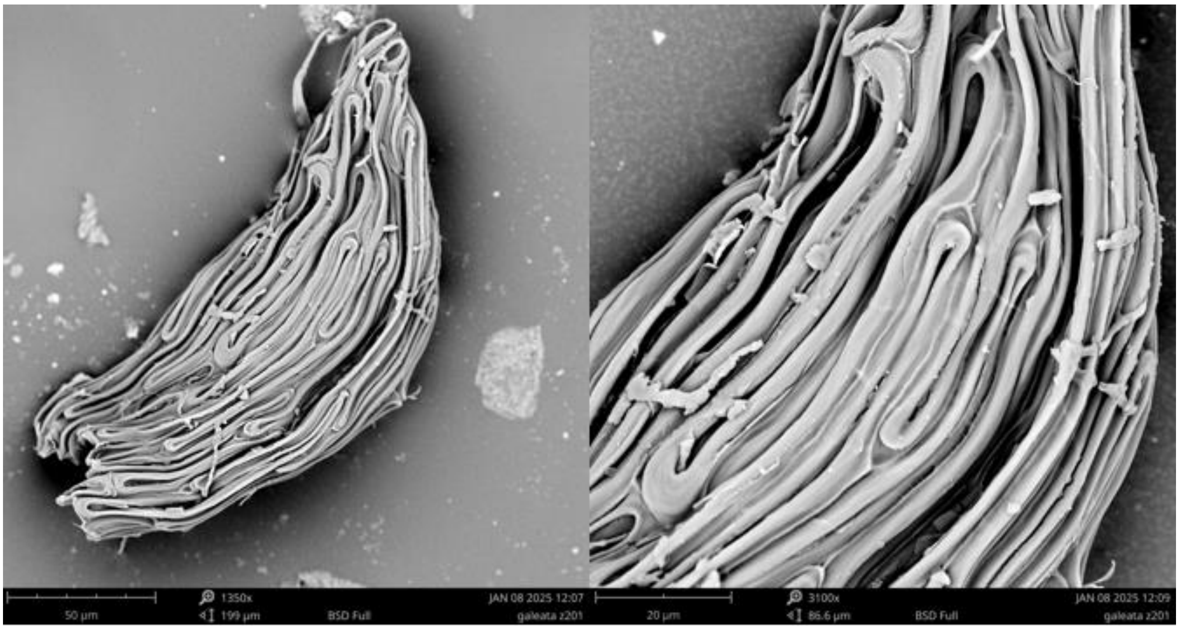

Fig.S92. *Polystachya galeata* (Sw.) Rchb.f. (P. gal3)

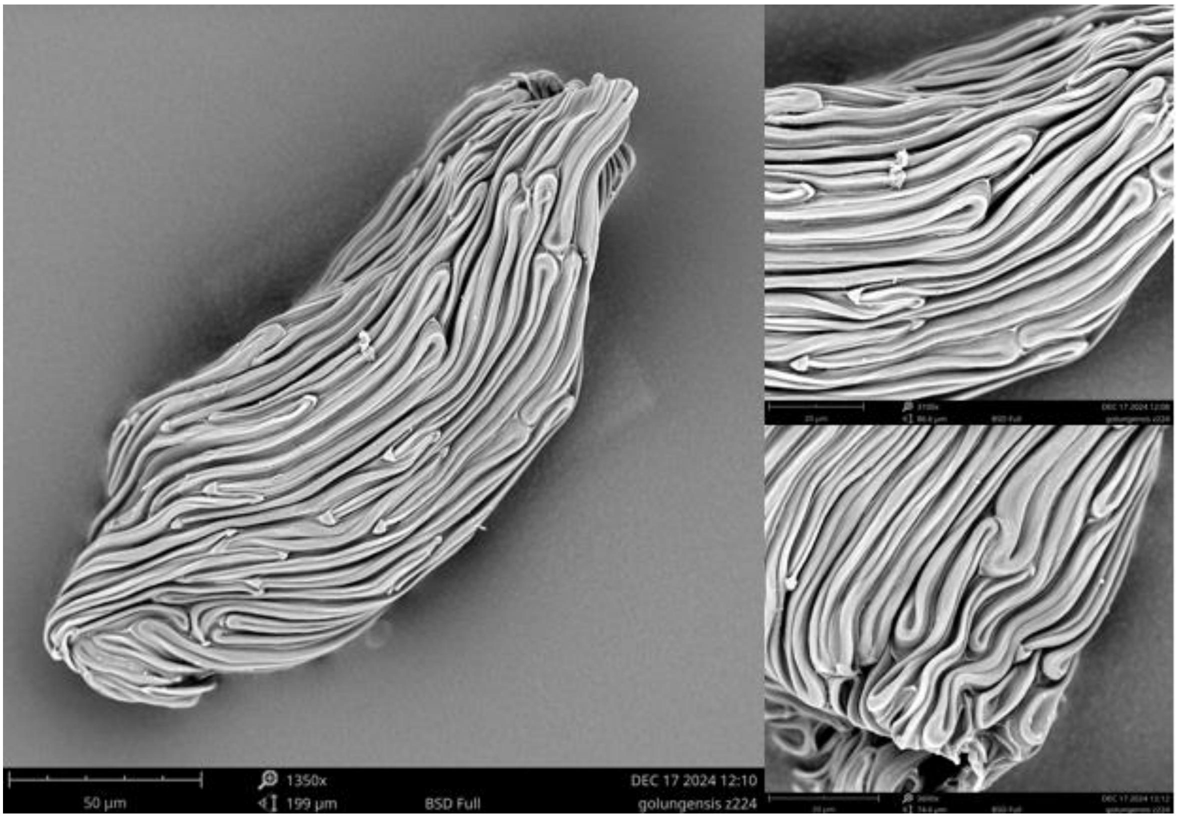

Fig.S93. *Polystachya golungensis* Rchb.f. (P. gol)

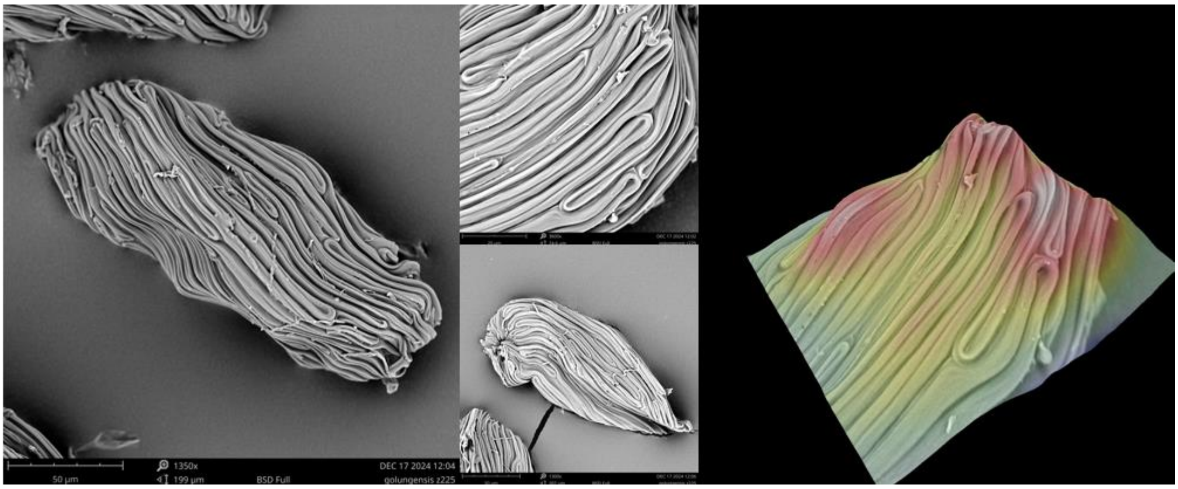

Fig.S94. *Polystachya golungensis* Rchb.f. (P. gol2)

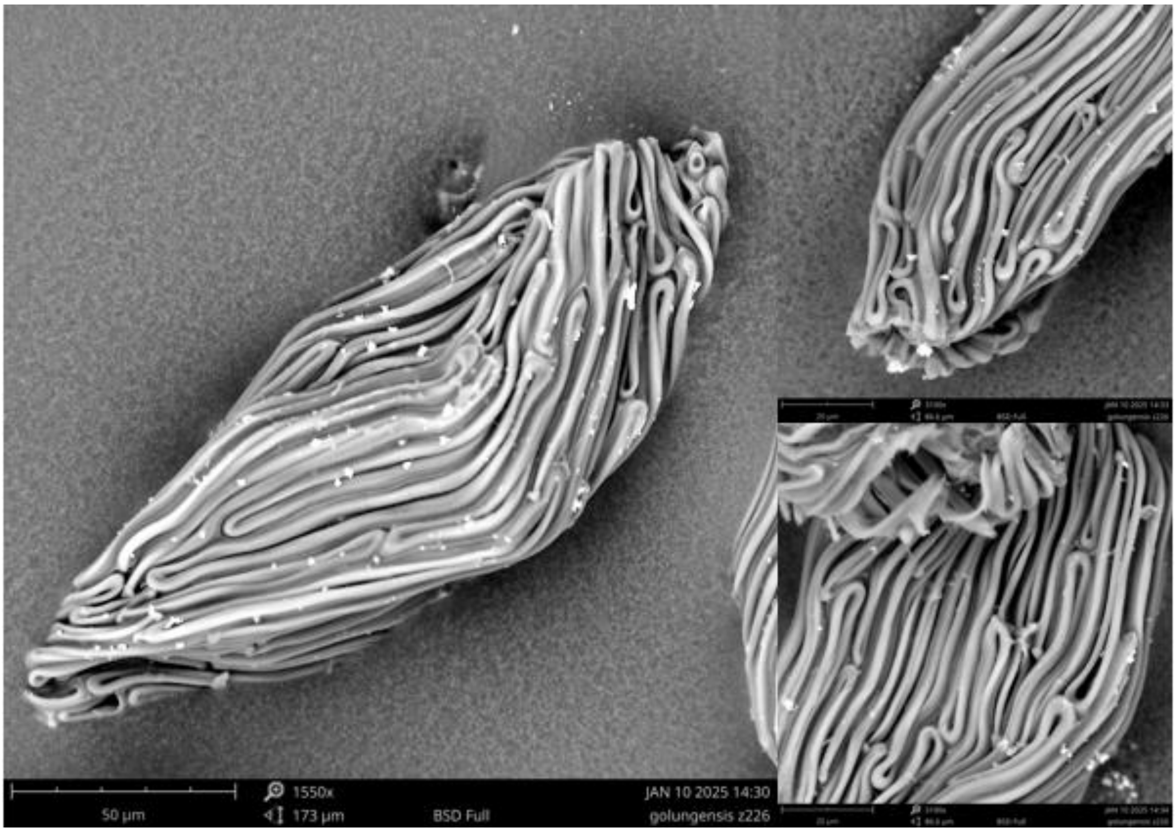

Fig.S95. *Polystachya golungensis* Rchb.f. (P. gol3)

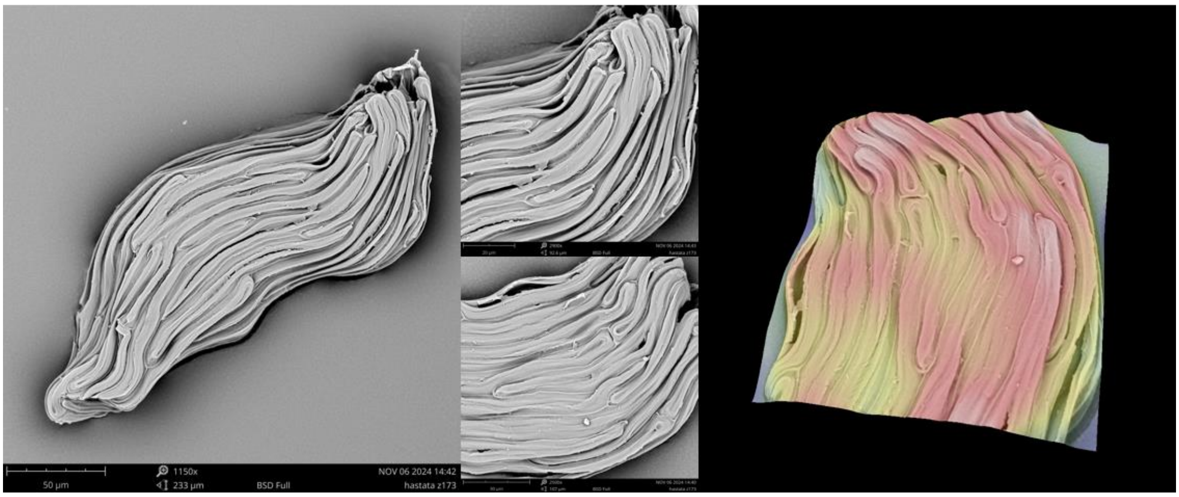

Fig.S96. *Polystachya hastata* Summerh. (P. has)

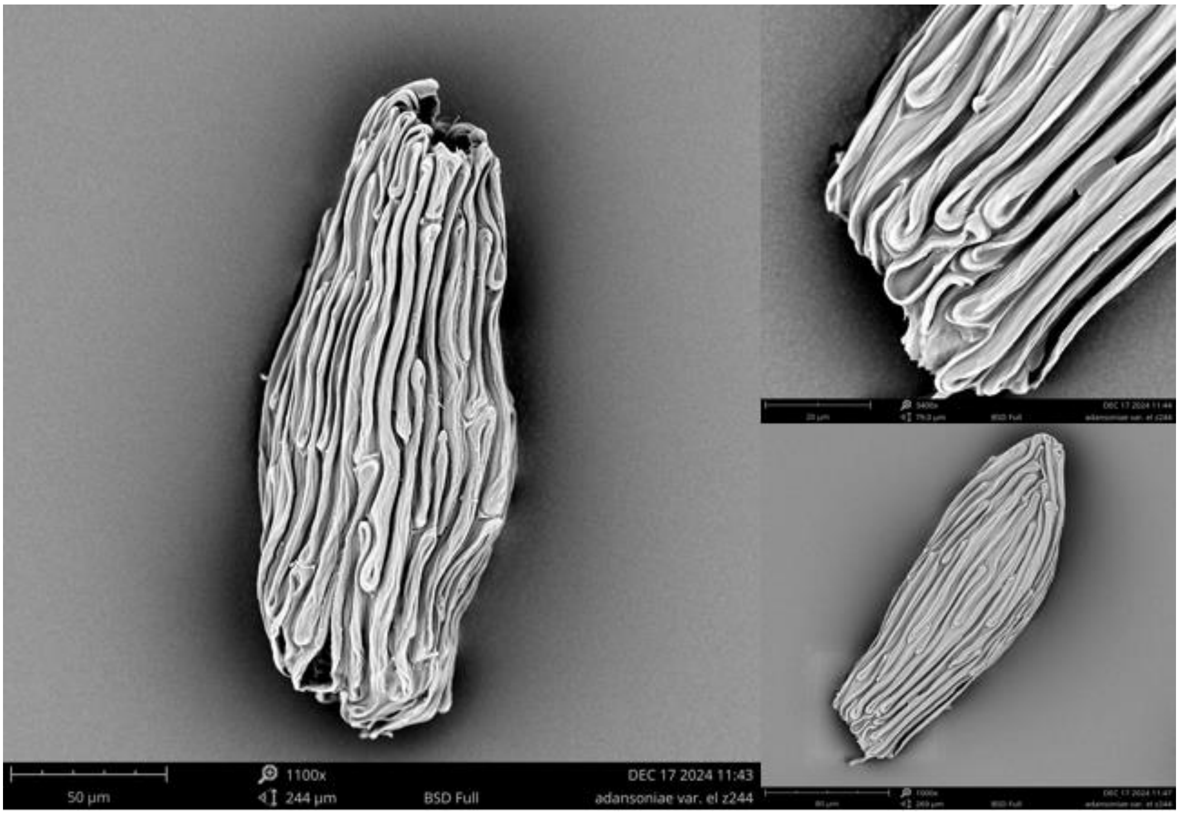

Fig.S97. *Polystachya hastata* Summerh. (P. has2)

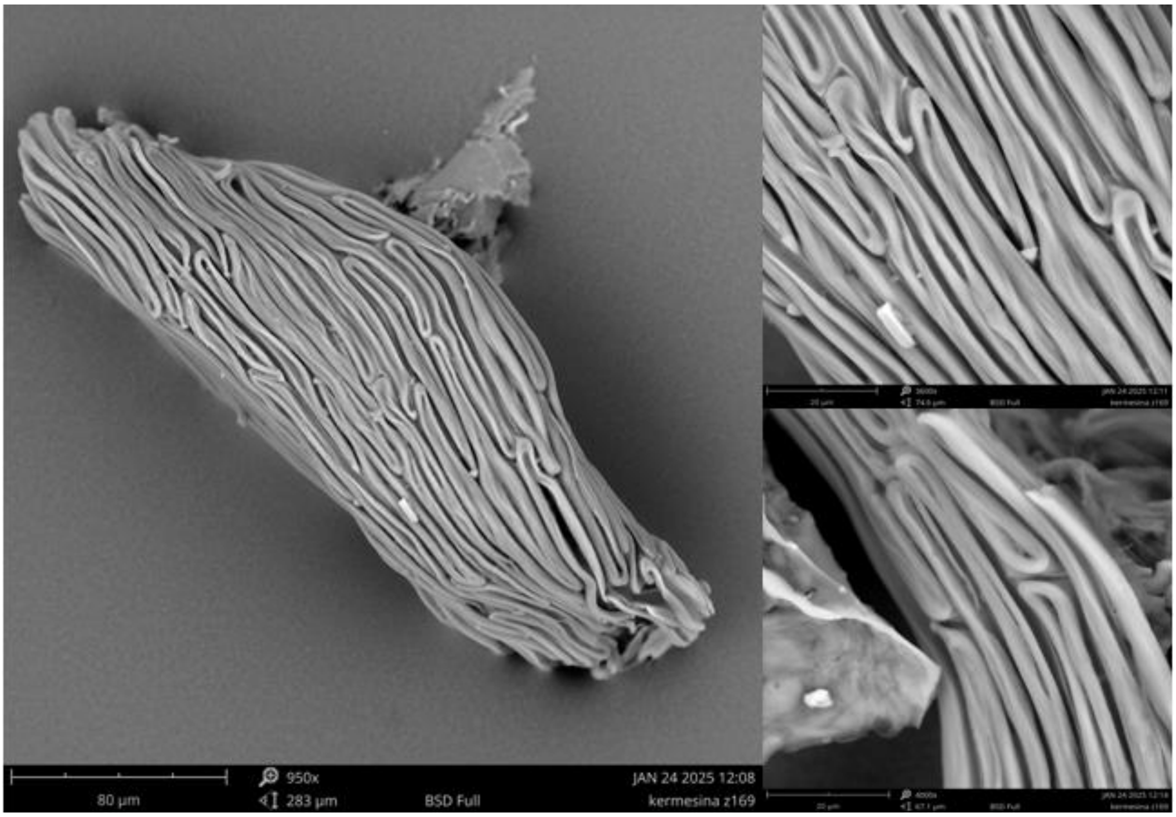

Fig.S98. *Polystachya kermesina* Kraenzl. (P. ker)

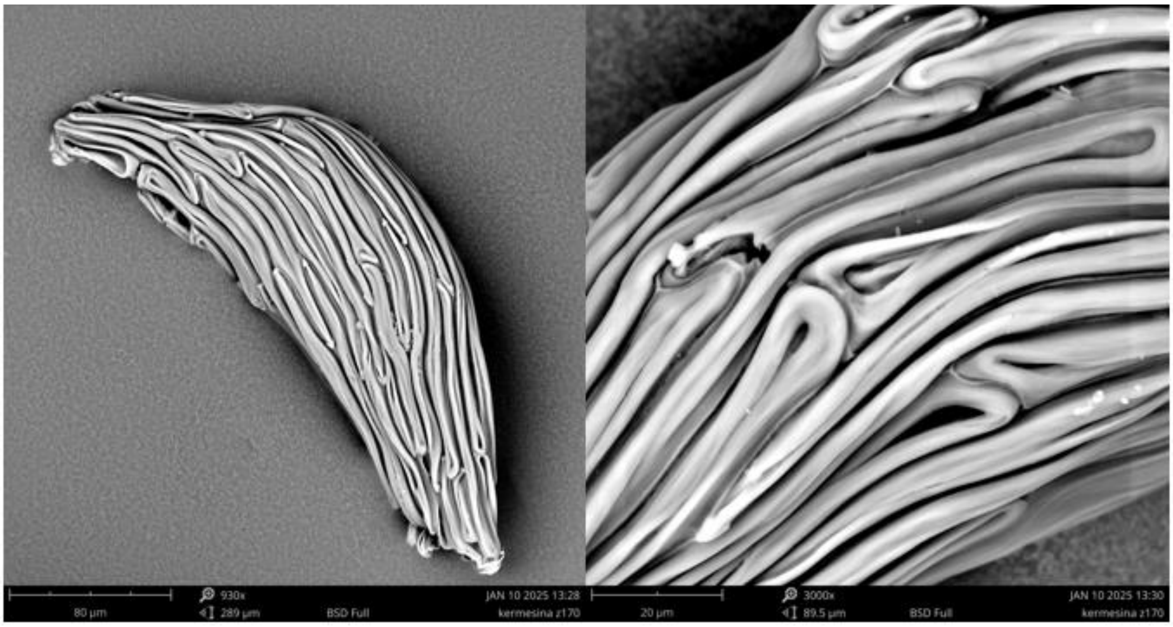

Fig.S99. *Polystachya kermesina* Kraenzl. (P. ker2)

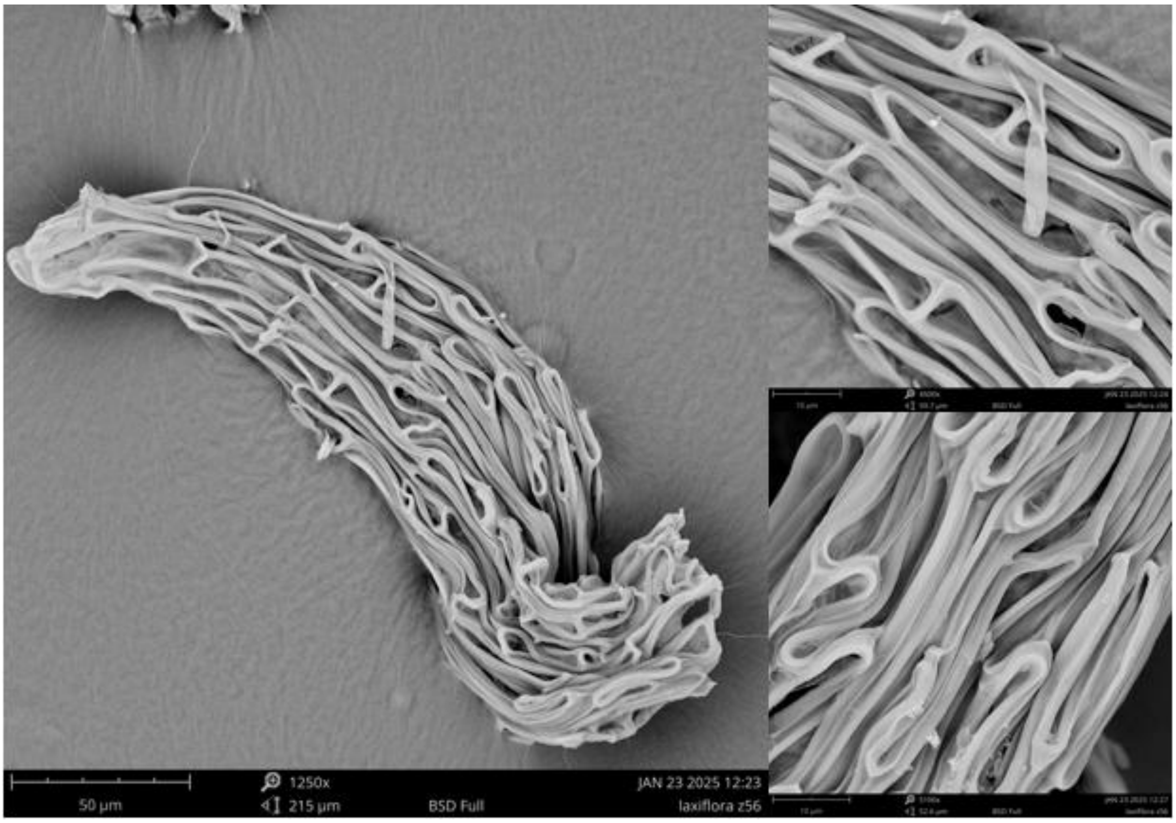

Fig.S100. *Polystachya laxiflora* Lindl. (P. lax)

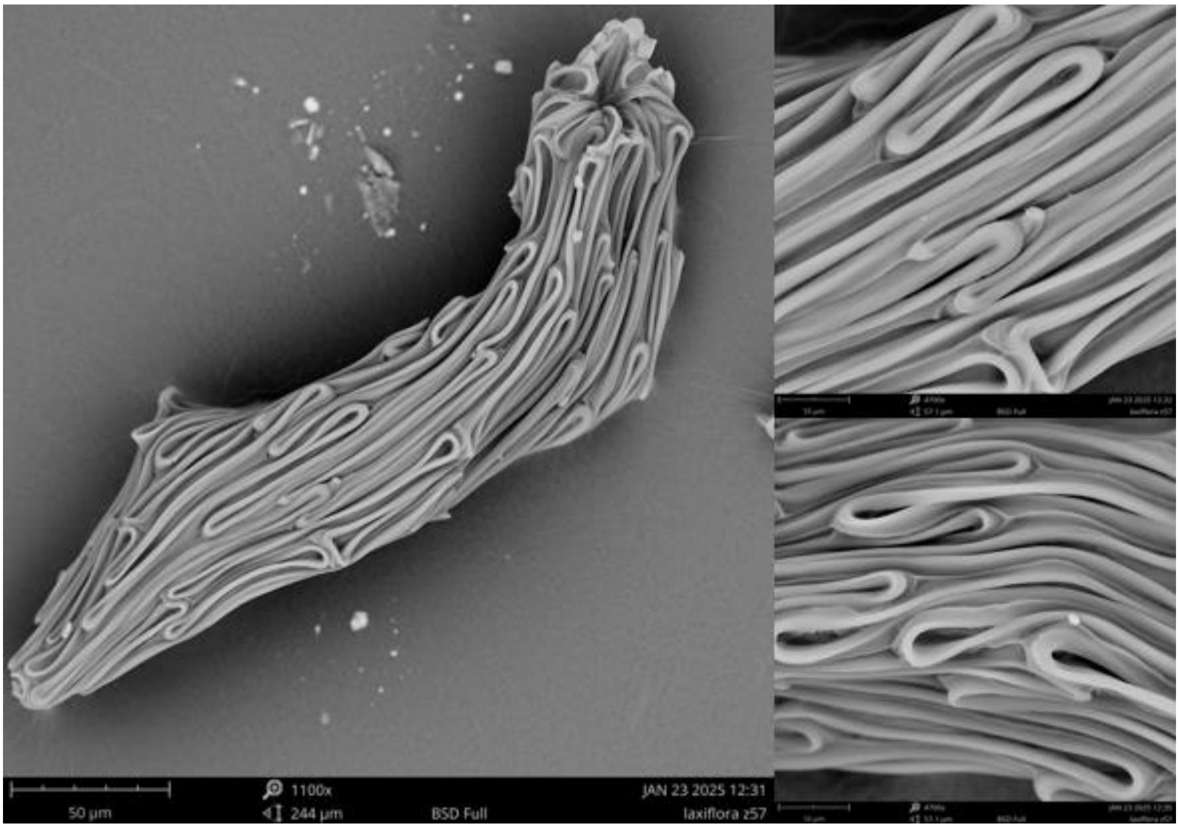

Fig.S101. *Polystachya laxiflora* Lindl. (P. lax2)

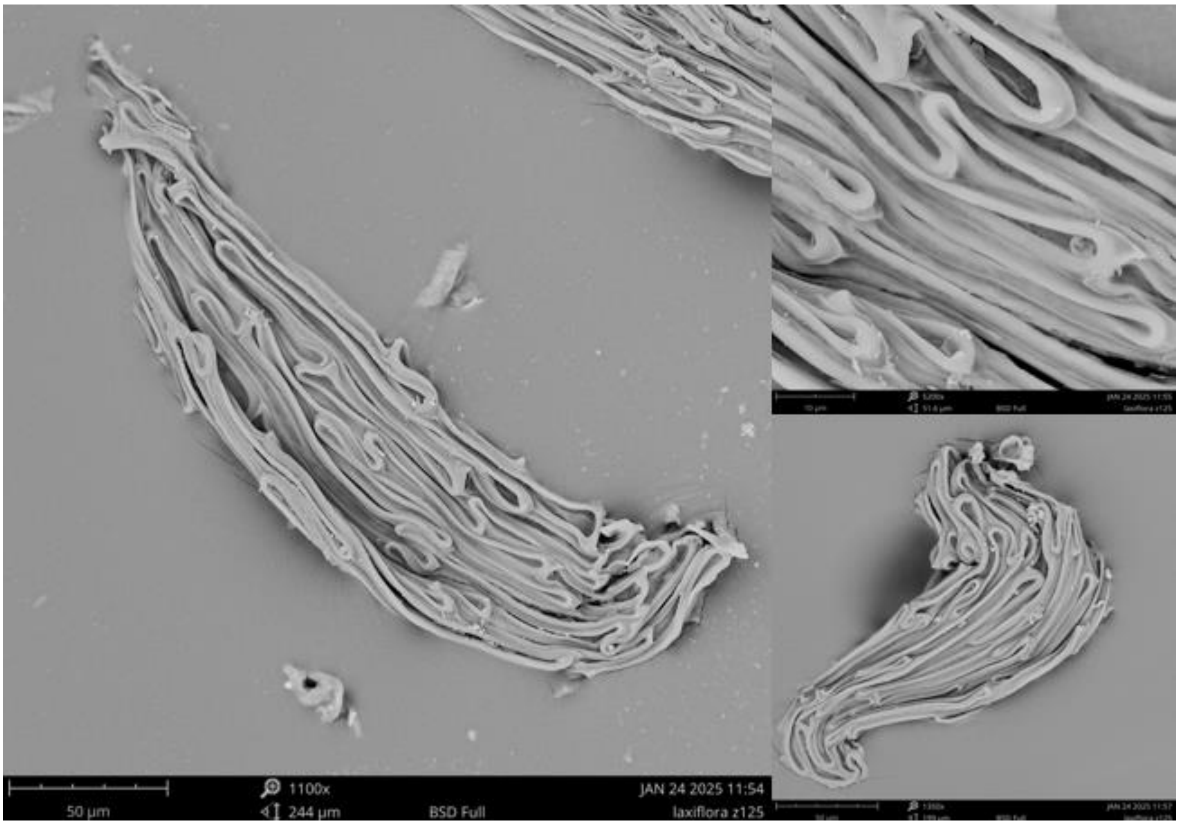

Fig.S102. *Polystachya laxiflora* Lindl. (P. lax3)

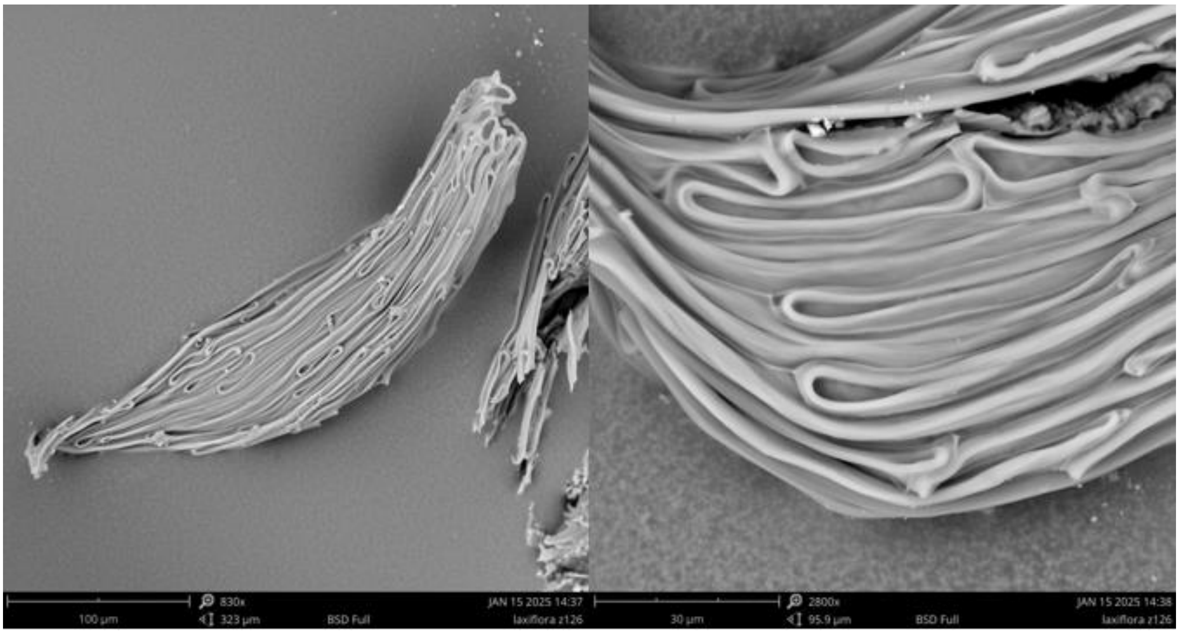

Fig.S103. *Polystachya laxiflora* Lindl. (P. lax4)

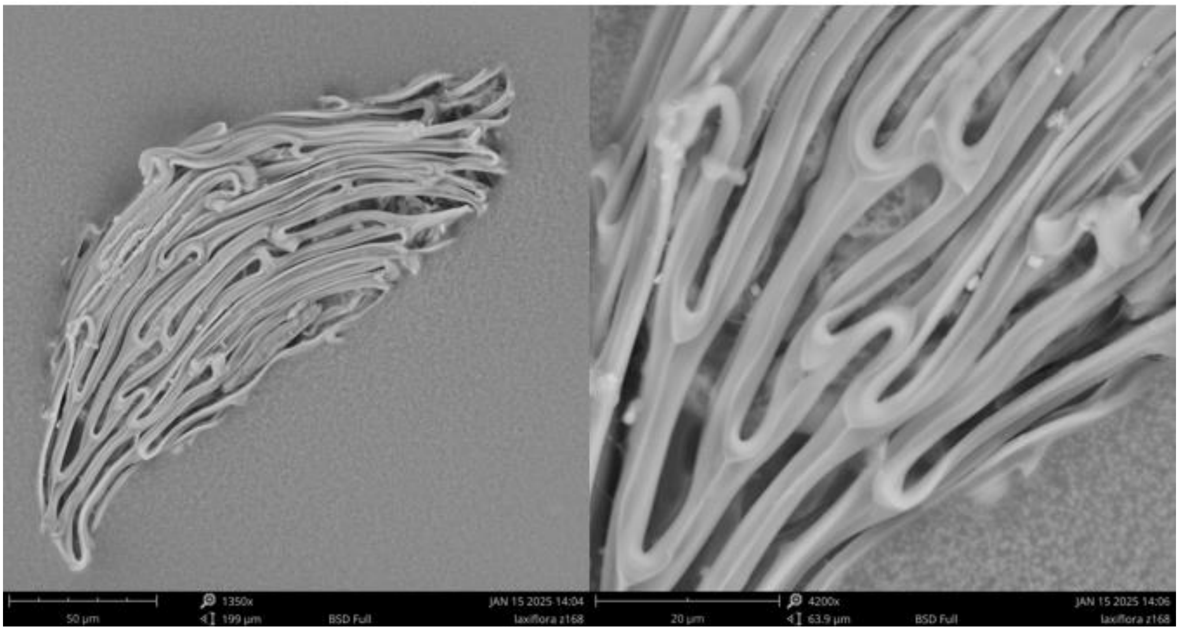

Fig.S104. *Polystachya laxiflora* Lindl. (P. lax5)

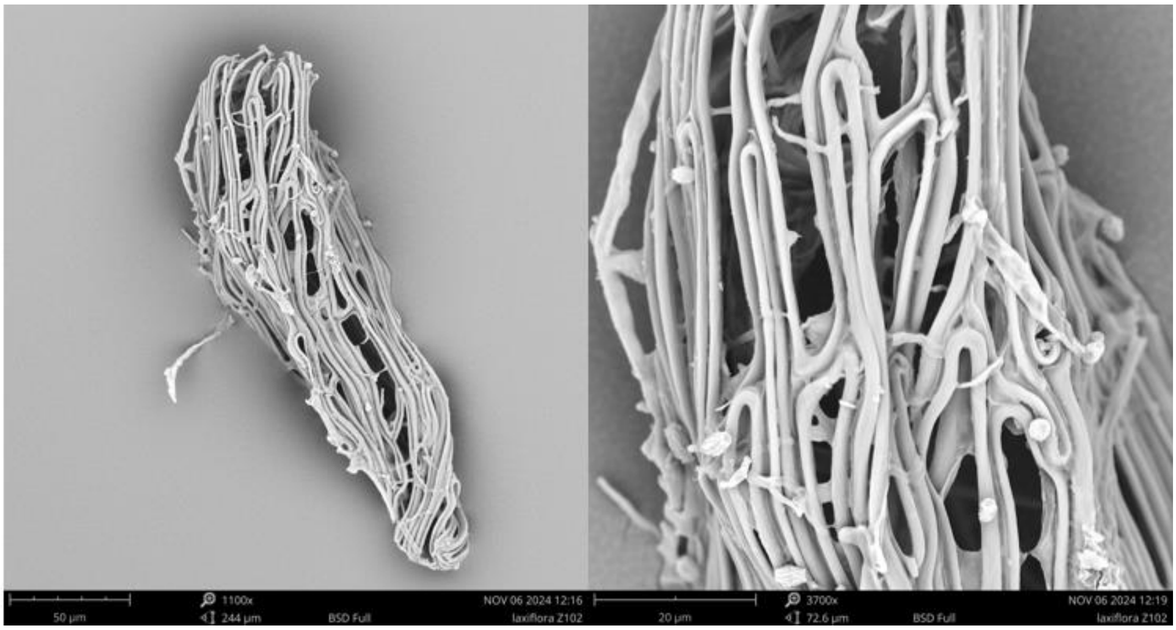

Fig.S105. *Polystachya laxiflora* Lindl. (P. lax6)

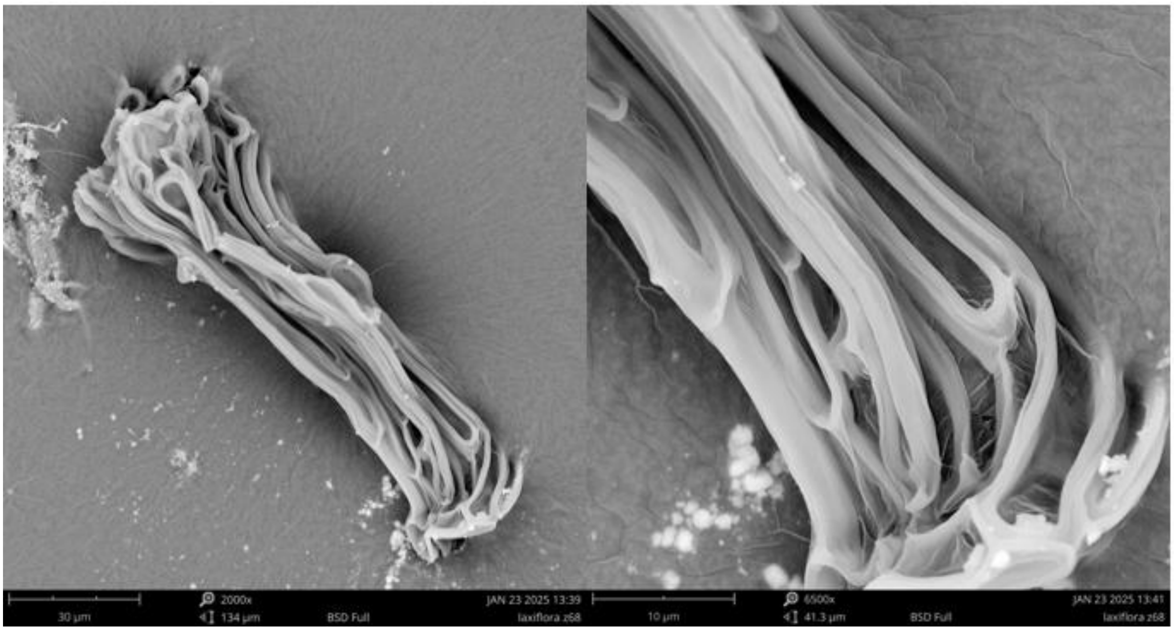

Fig.S106. *Polystachya laxiflora* Lindl. (P. lax7)

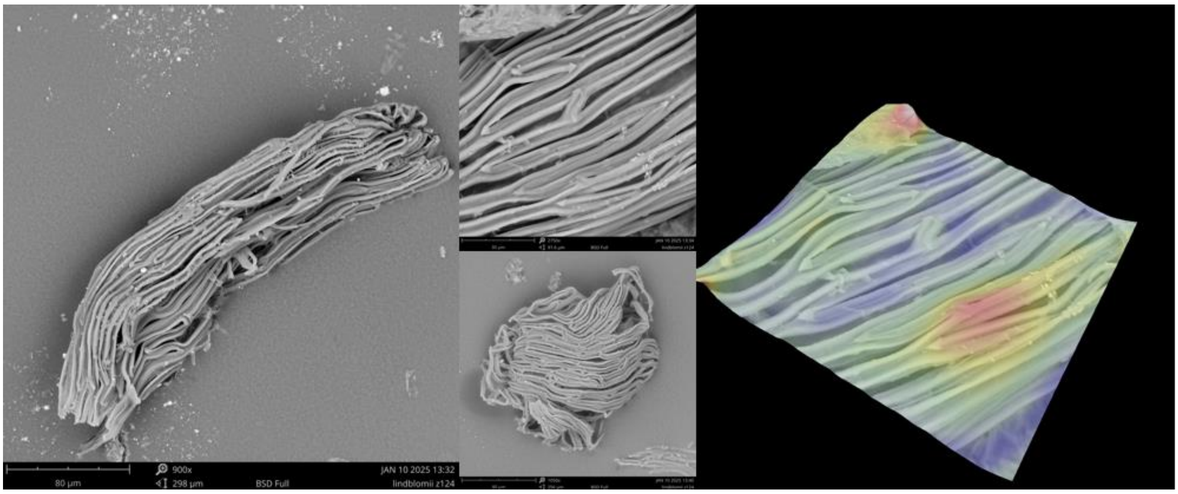

Fig.S107. *Polystachya lindblomii* Schltr. (P. lin)

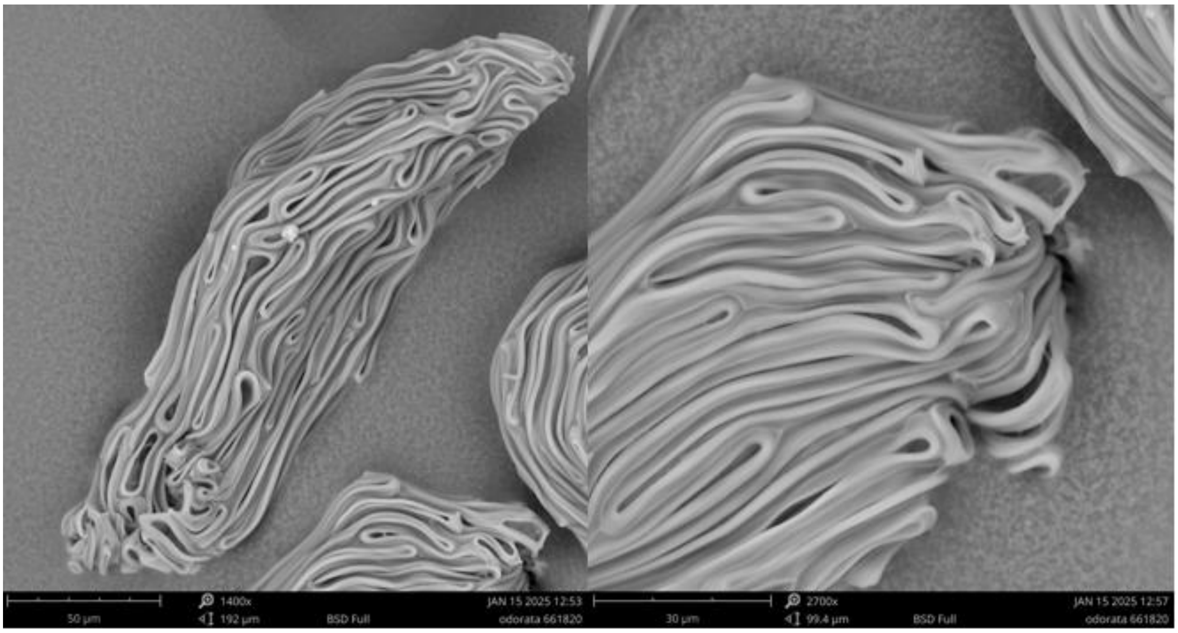

Fig.S108. *Polystachya maculata* P.J.Cribb (P. mac)

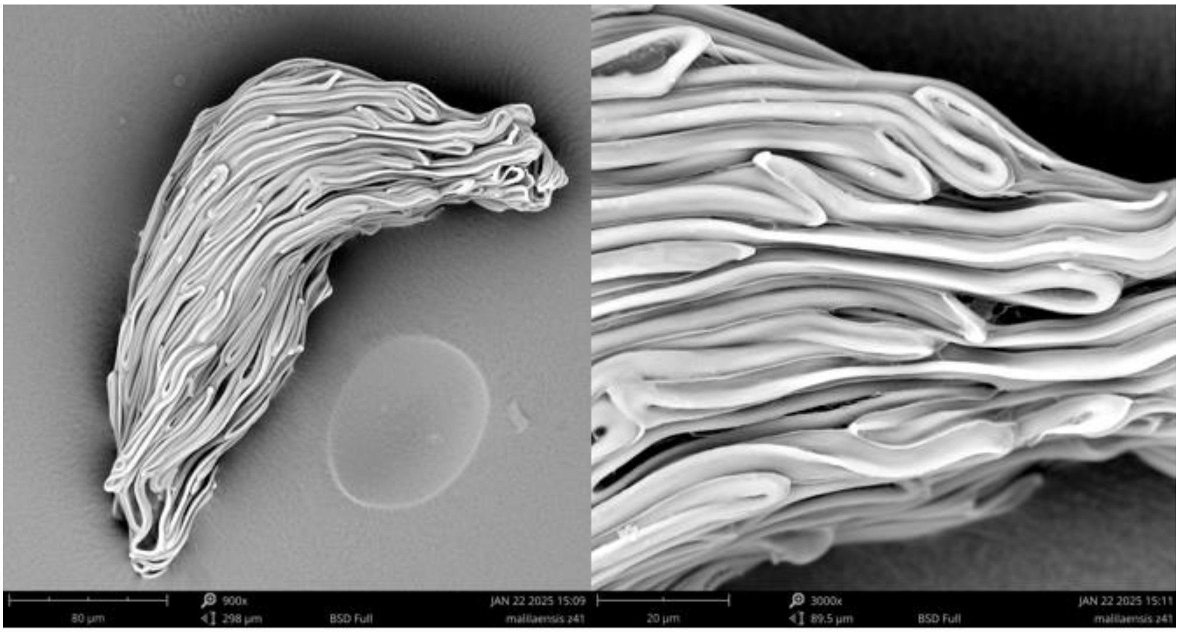

Fig.S109. *Polystachya malilaensis* Schltr. (P. mal)

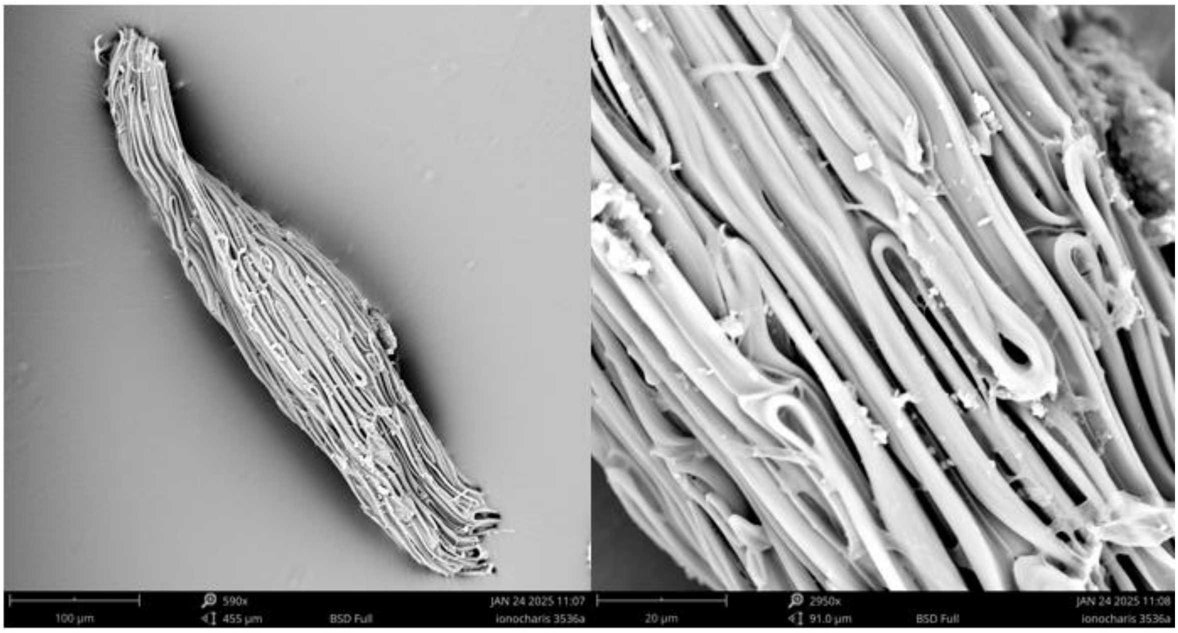

Fig.S110. *Polystachya melanantha* Schltr. Kraenzl. (P. mel)

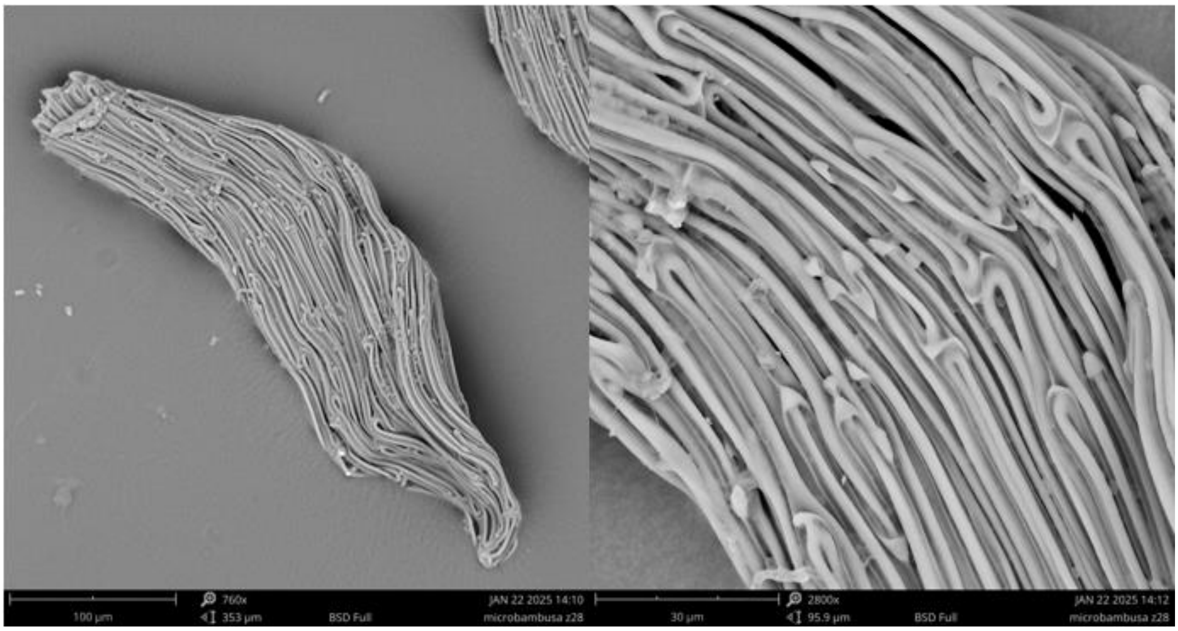

Fig.S111. *Polystachya microbambusa* Kraenzl. (P. mic)

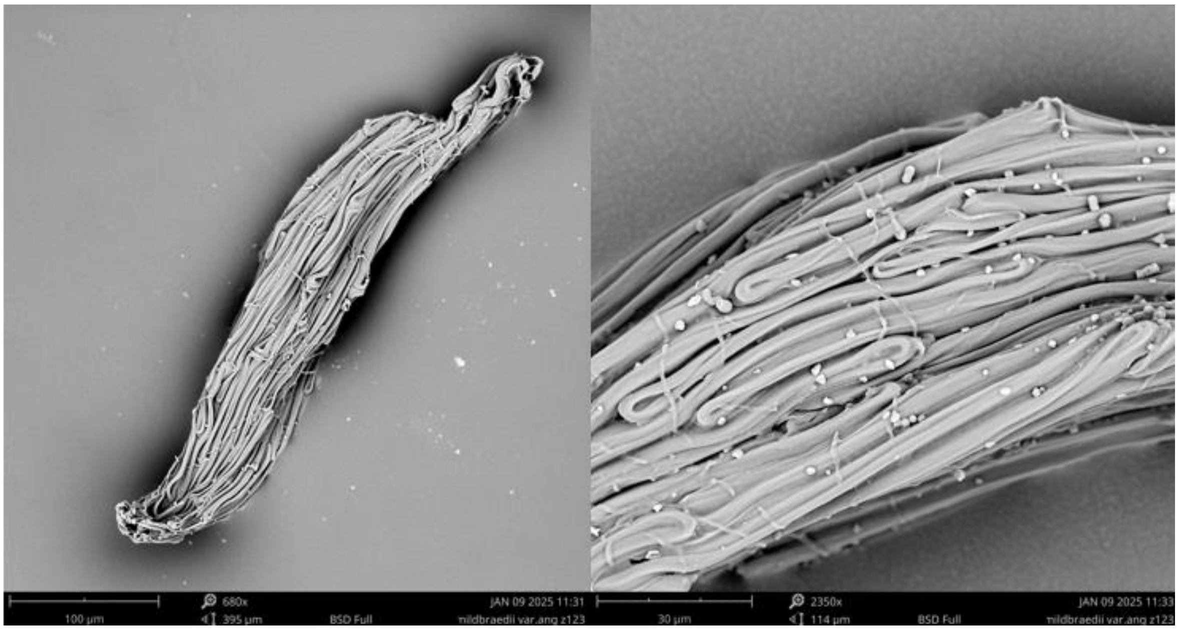

Fig.S112. *Polystachya mildbraedii* var. *angustifolia* (Summerh.) Geerinck (P. mil. var. ang)

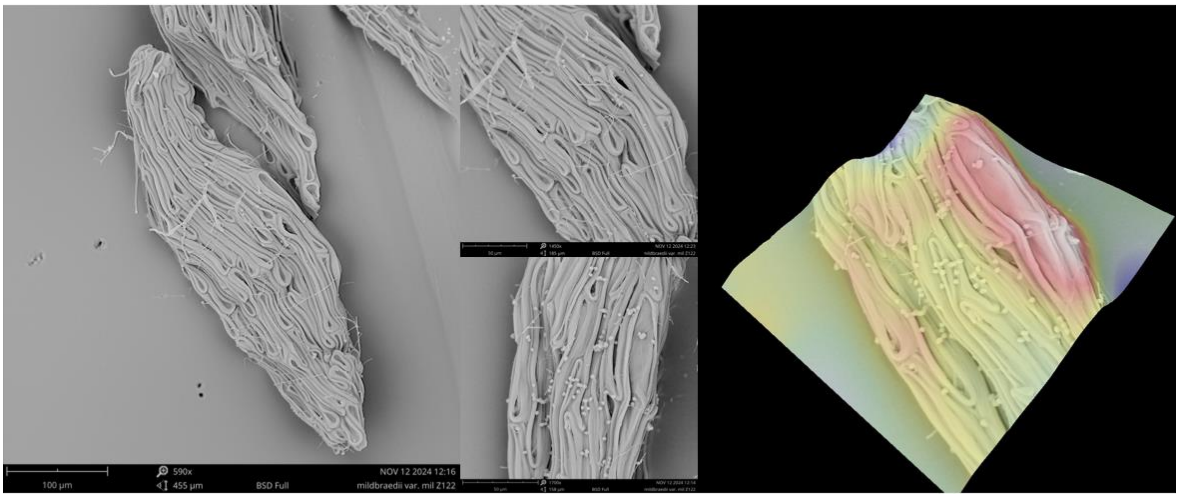

Fig.S113. *Polystachya mildbraedii* var. *mildbraedii* (P. mil. var. mil)

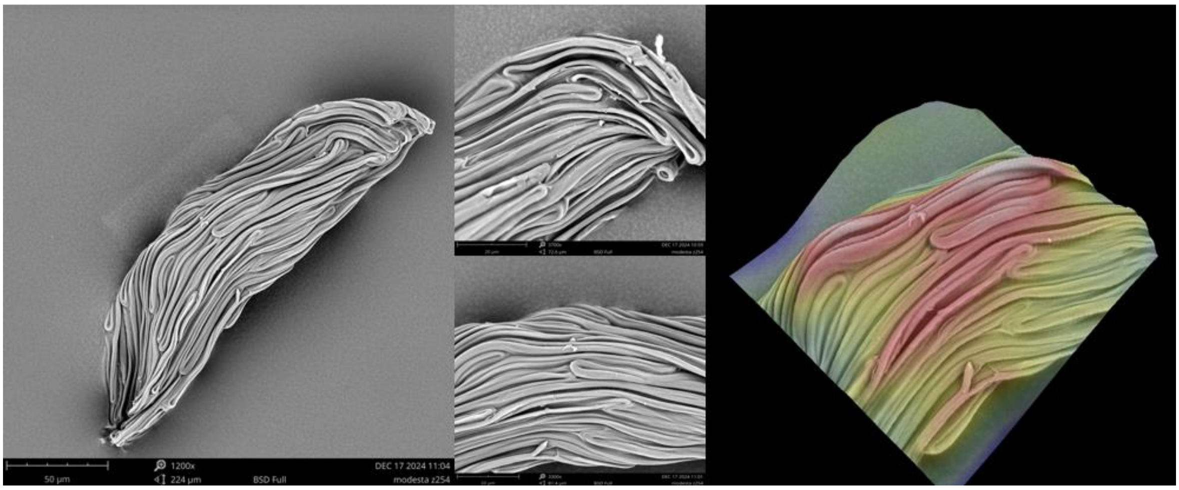

Fig.S114. *Polystachya modesta* Rchb.f. (P. mod2)

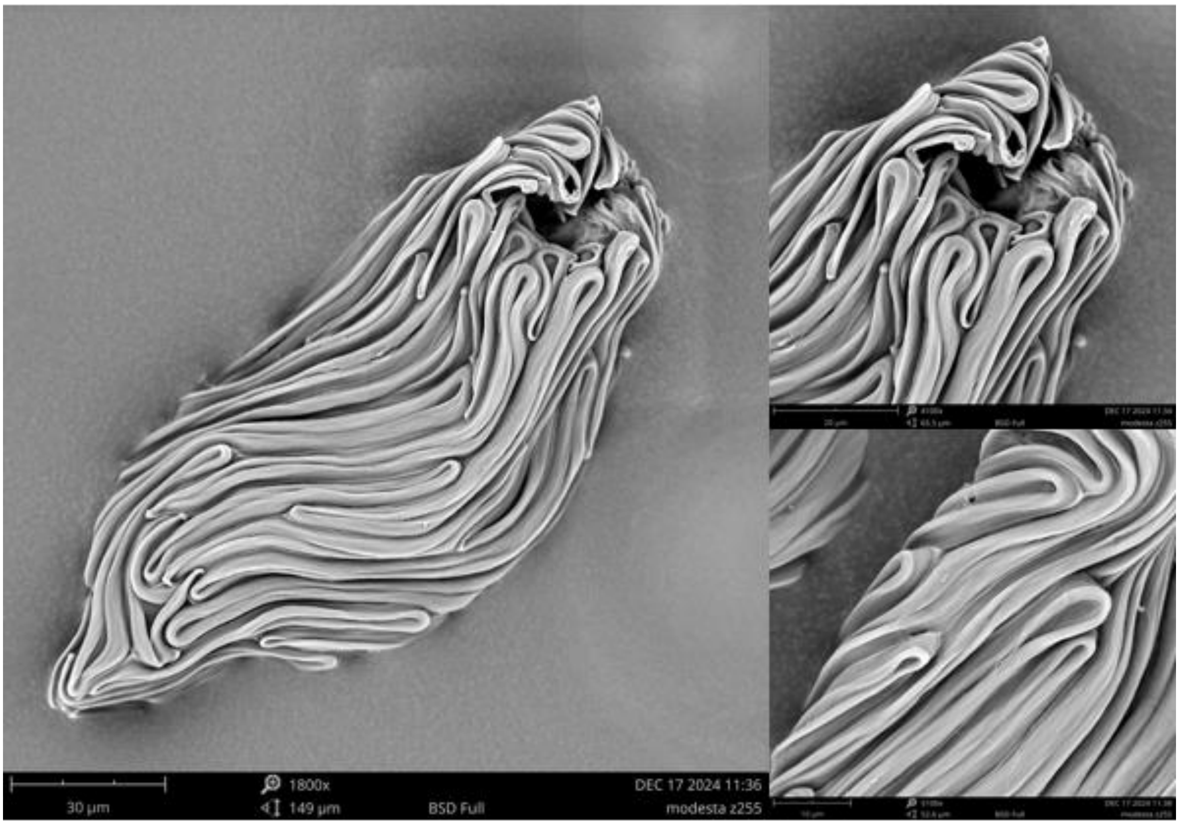

Fig.S115. *Polystachya modesta* Rchb.f. (P. mod3)

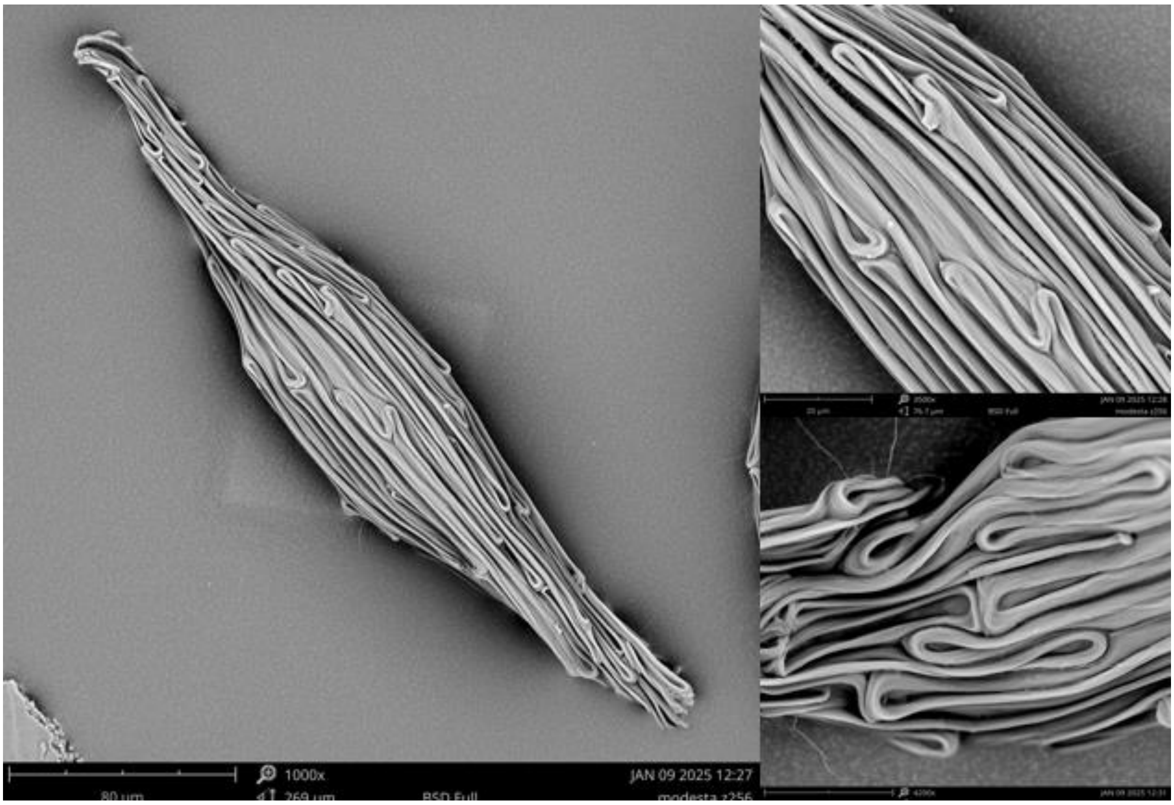

Fig.S116. *Polystachya modesta* Rchb.f. (P. mod4)

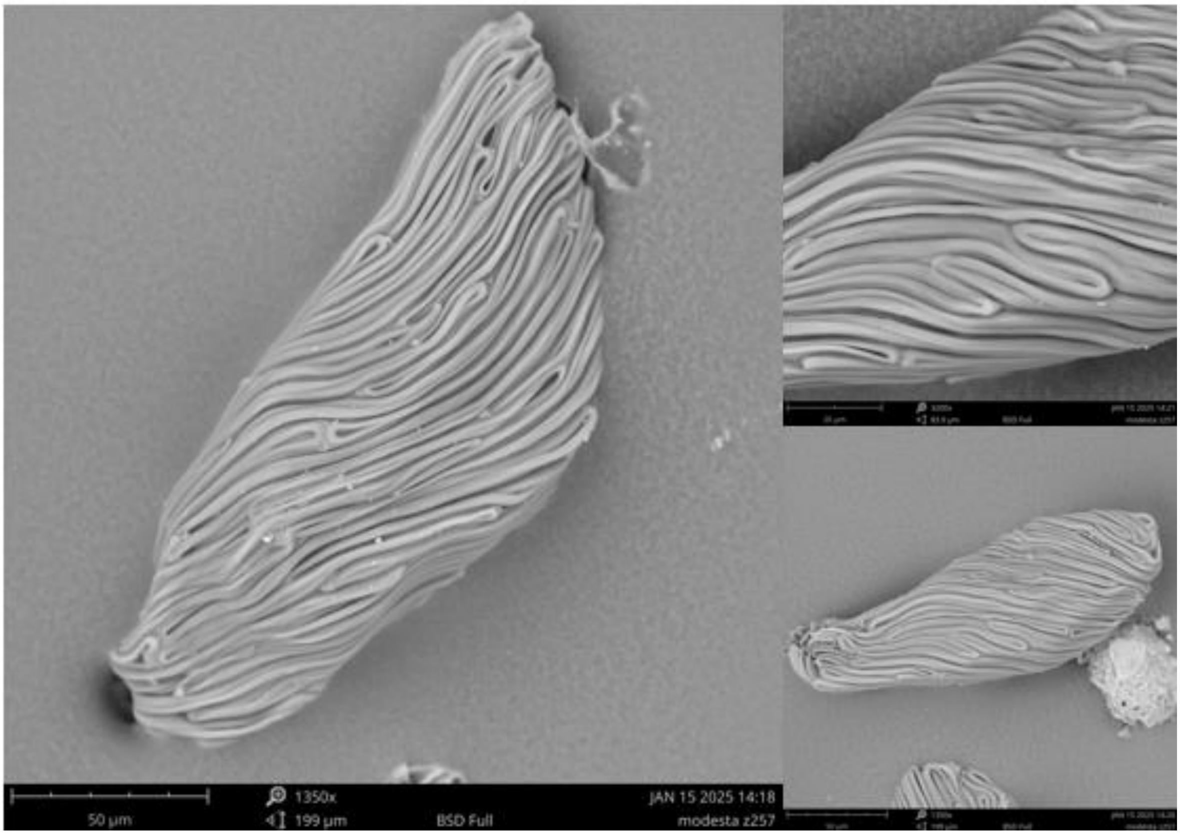

Fig.S117. *Polystachya modesta* Rchb.f. (P. mod5)

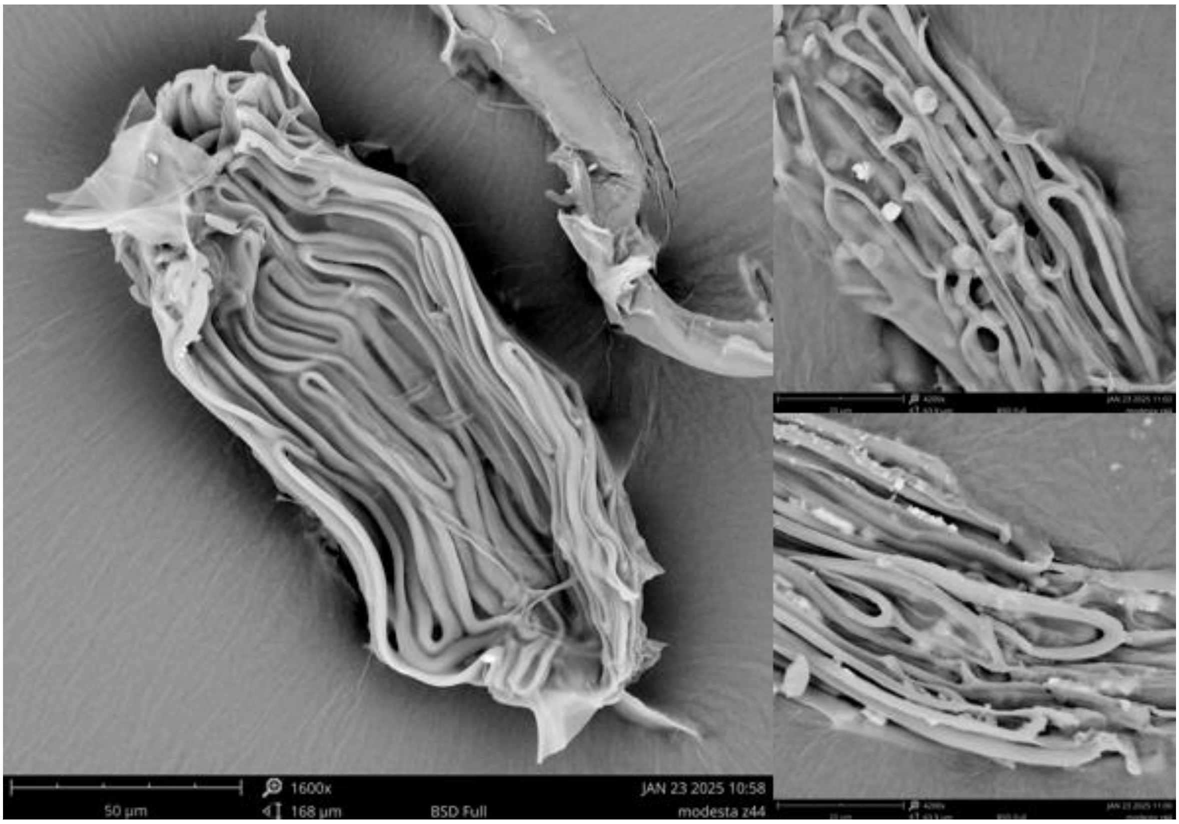

Fig.S118. *Polystachya modesta* Rchb.f. (P. mod6)

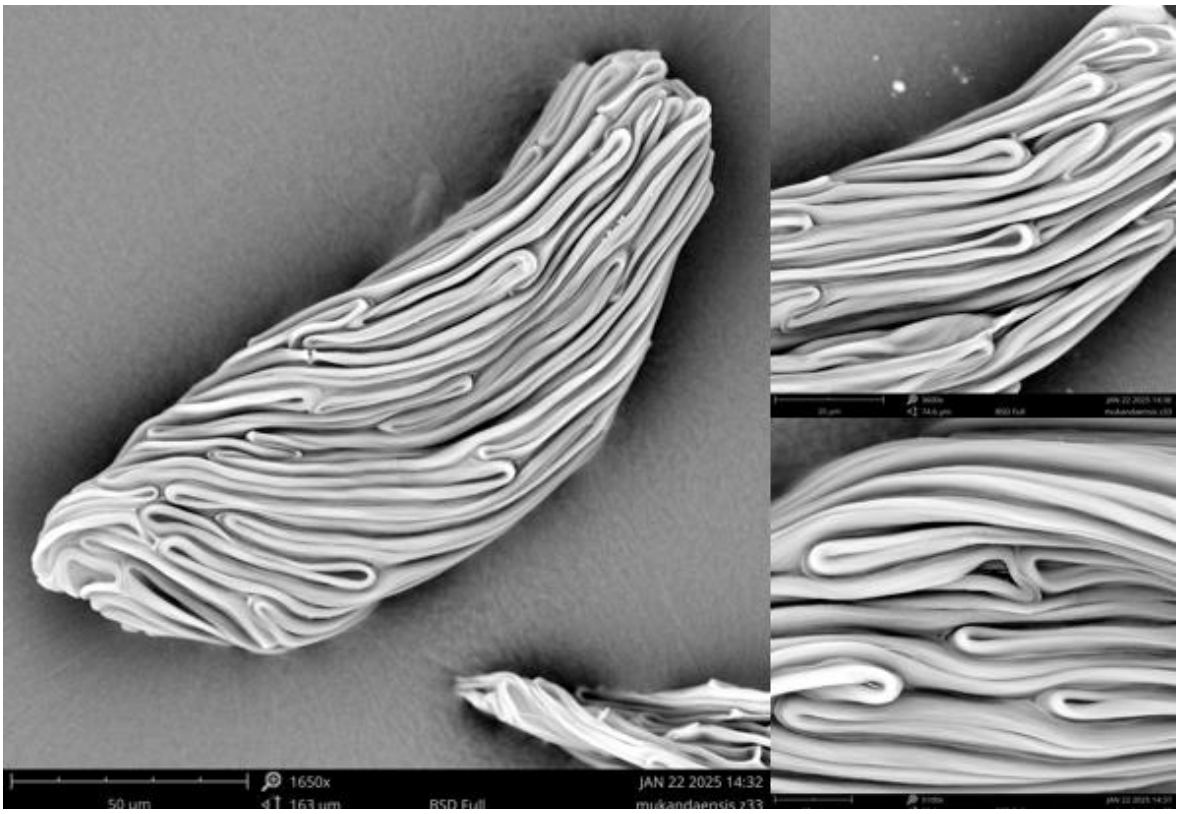

Fig.S119. *Polystachya mukandaensis* De Wild. (P. muk)

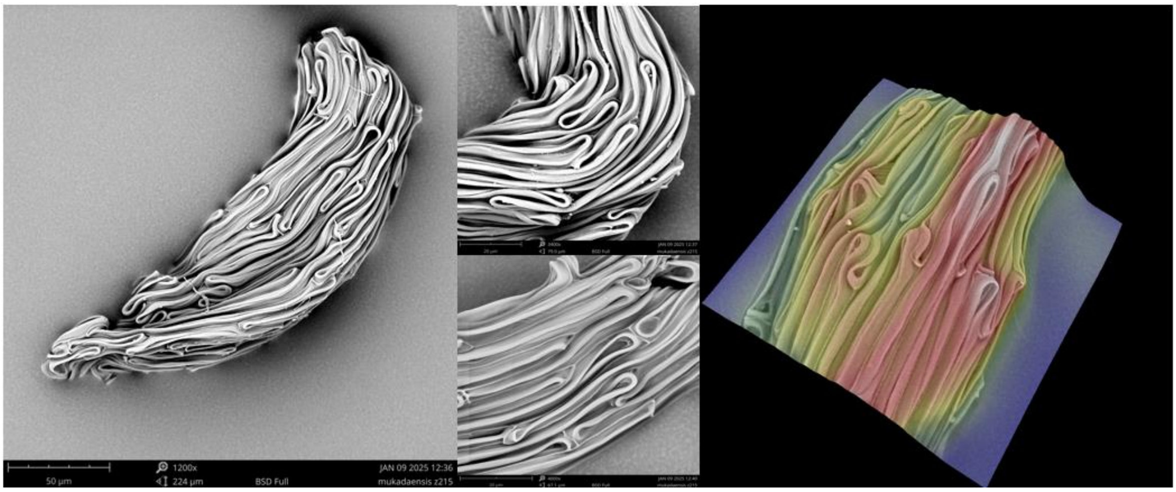

Fig.S120. *Polystachya mukandaensis* De Wild. (P. muk3)

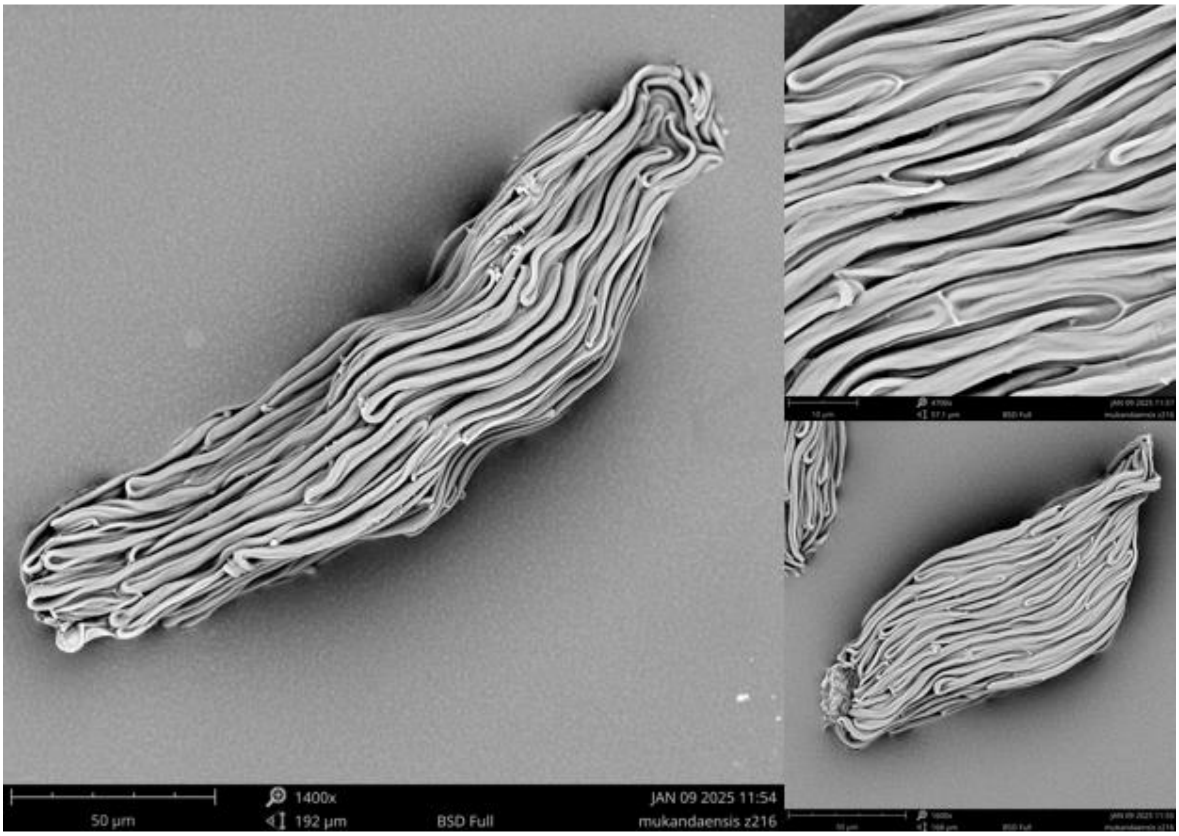

Fig.S121. *Polystachya mukandaensis* De Wild. (P. muk4)

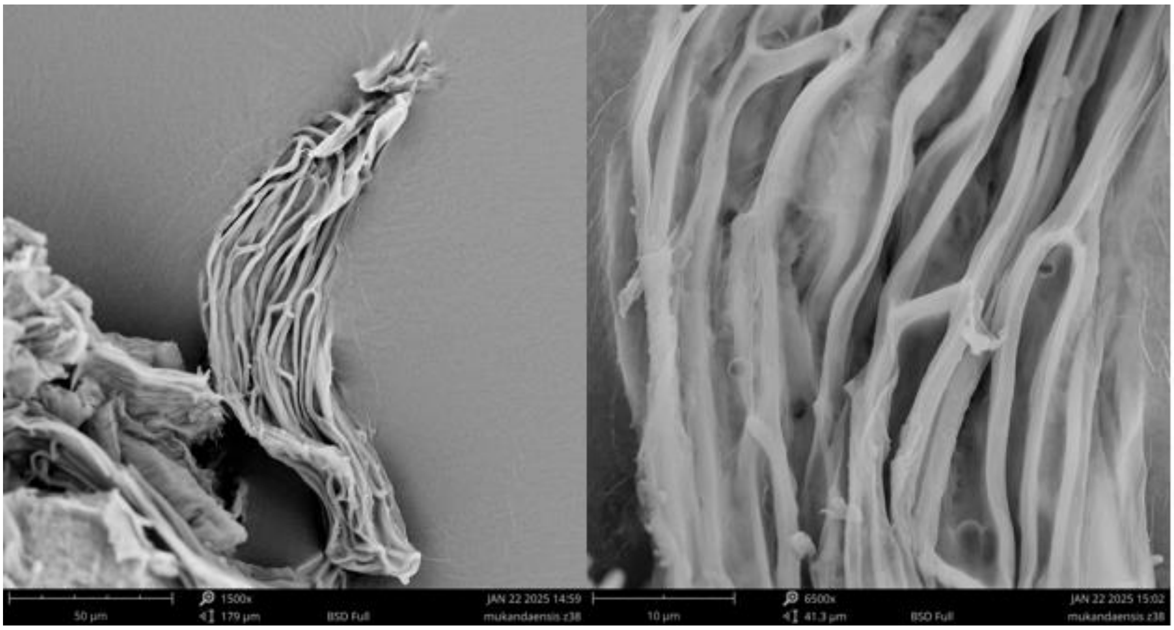

Fig.S122. *Polystachya mukandaensis* De Wild. (P. muk5)

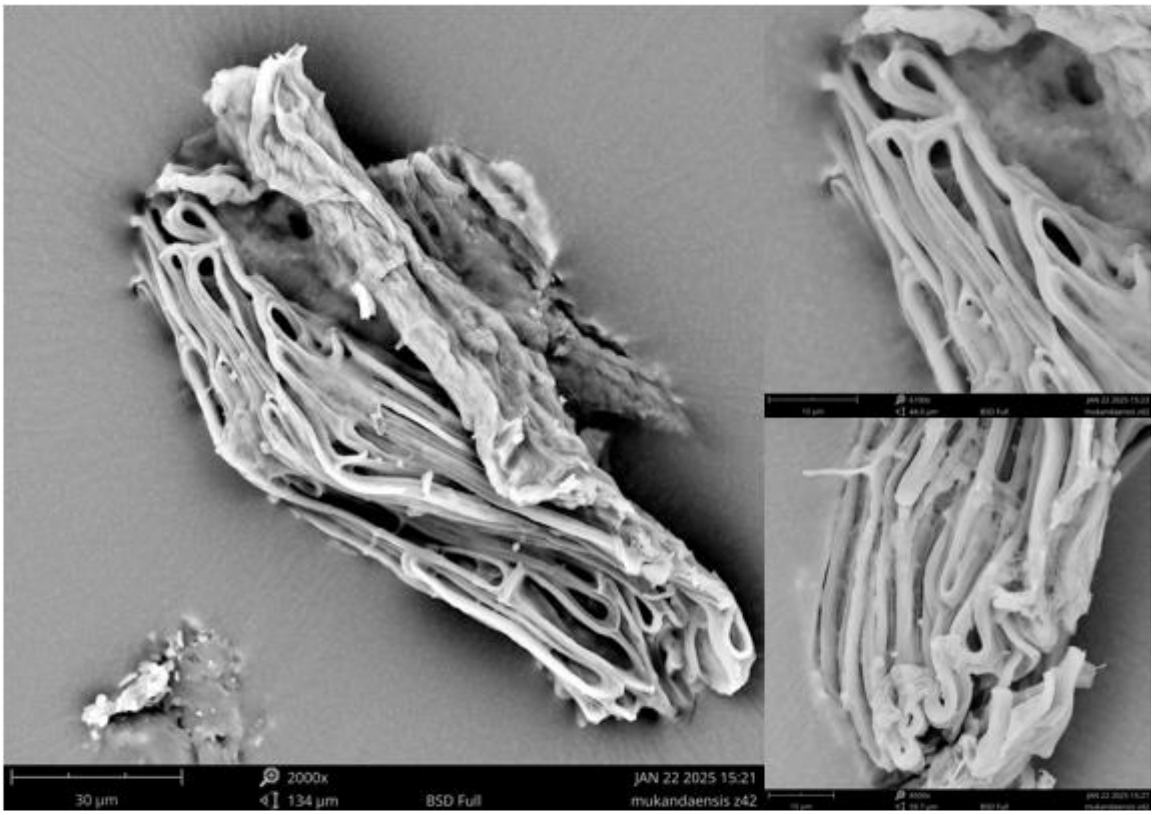

Fig.S123. *Polystachya mukandaensis* De Wild. (P. muk6)

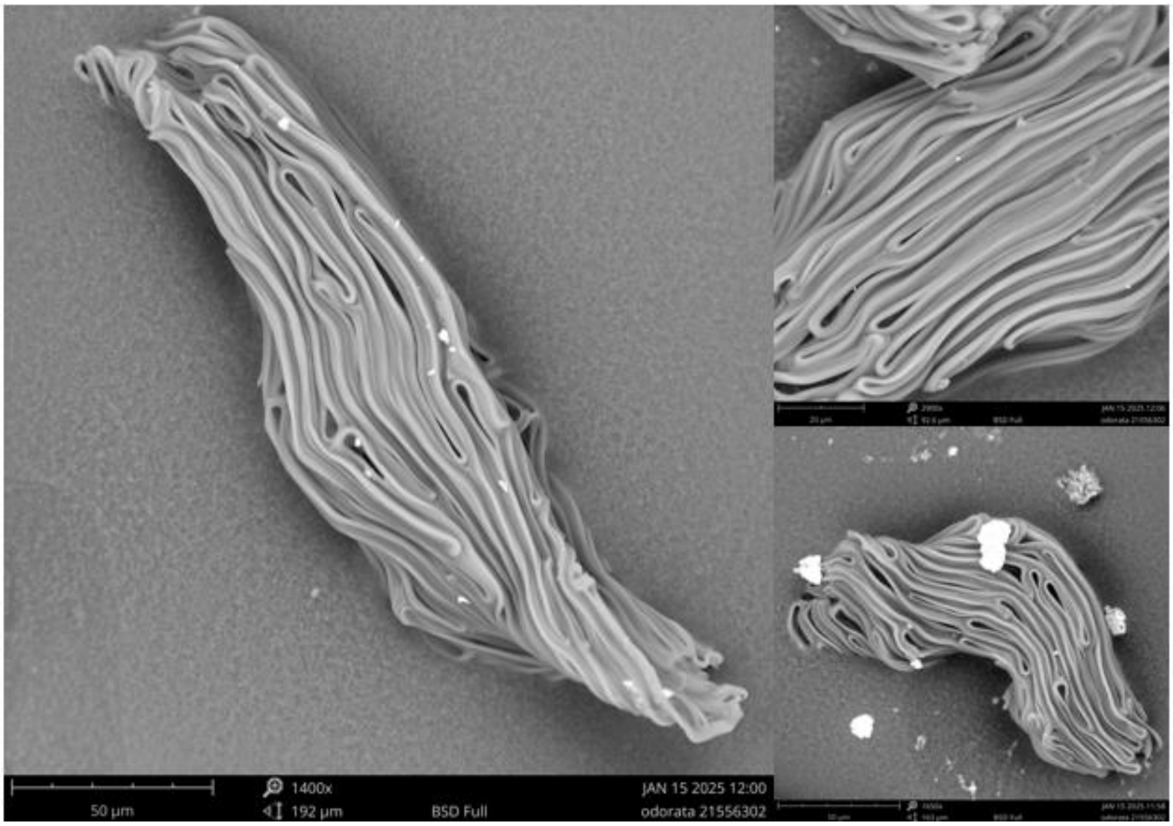

Fig.S124. *Polystachya odorata* Lindl. (P. odo)

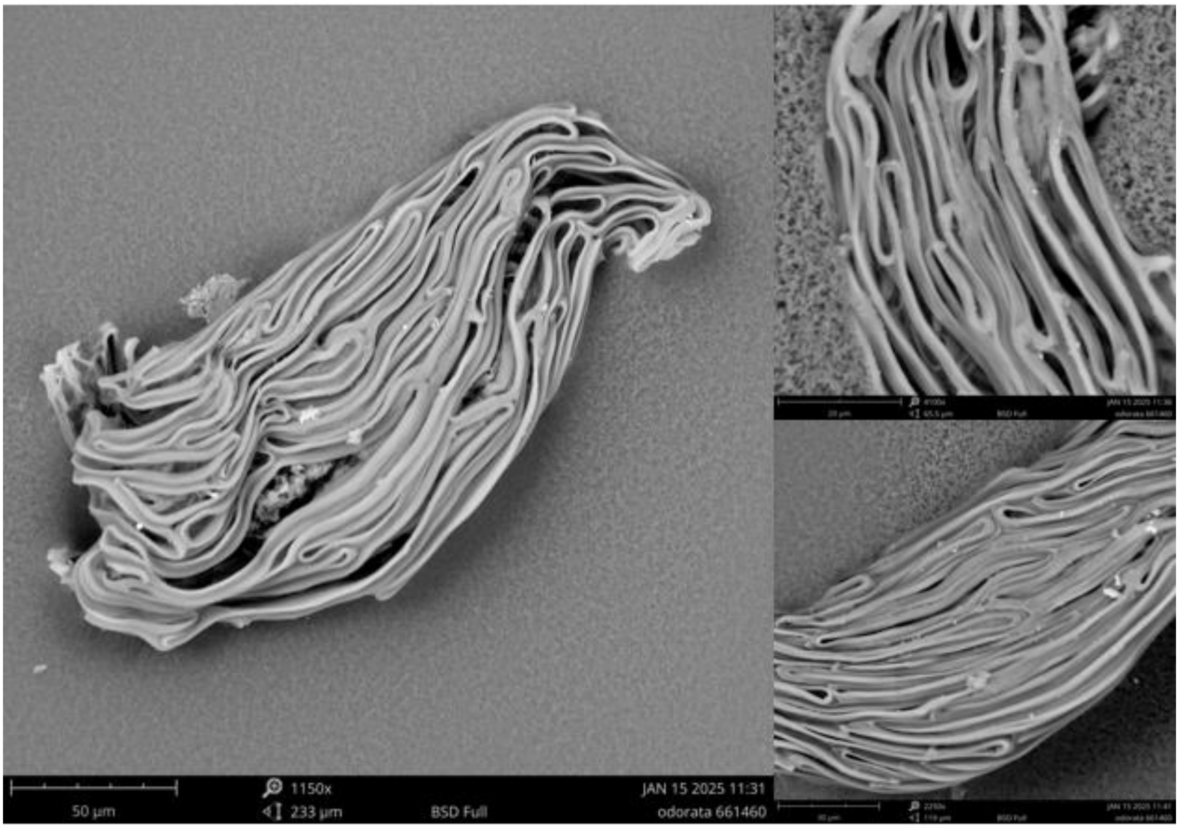

Fig.S125. *Polystachya odorata* Lindl. (P. odo2)

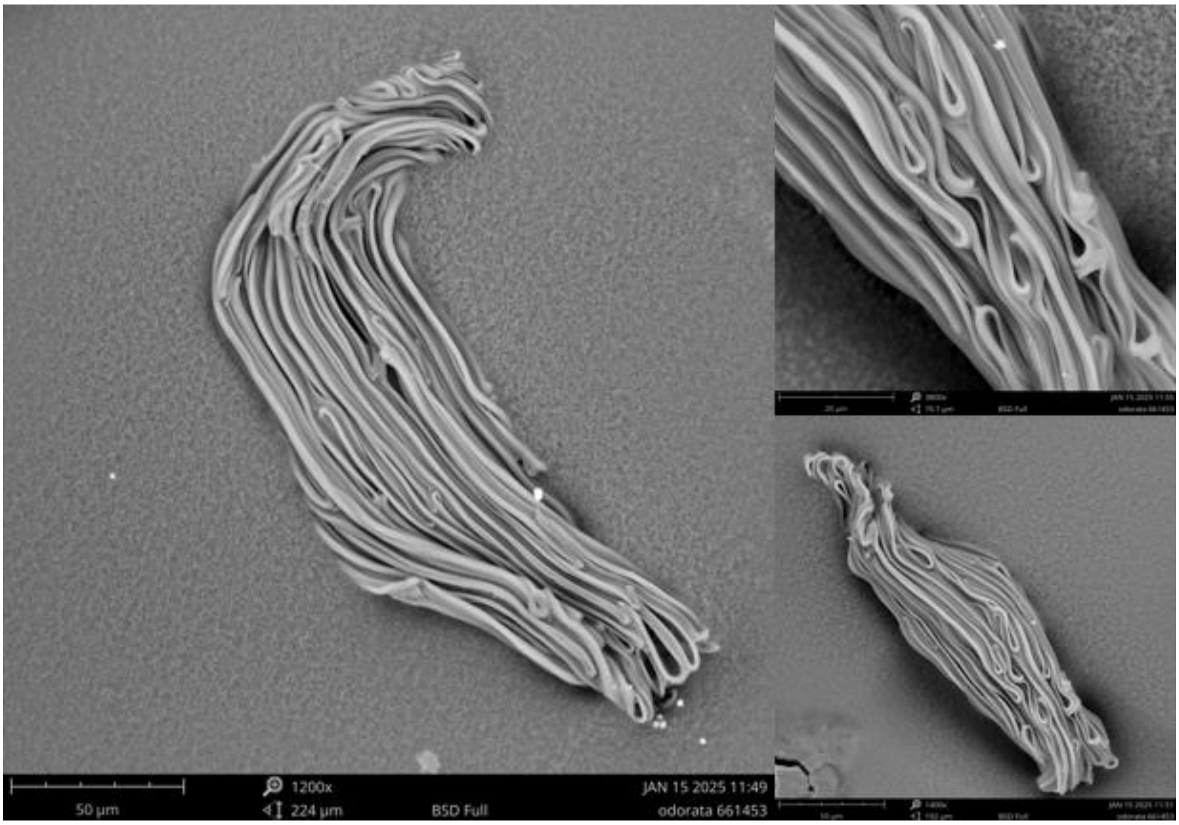

Fig.S126. *Polystachya odorata* Lindl. (P. odo3)

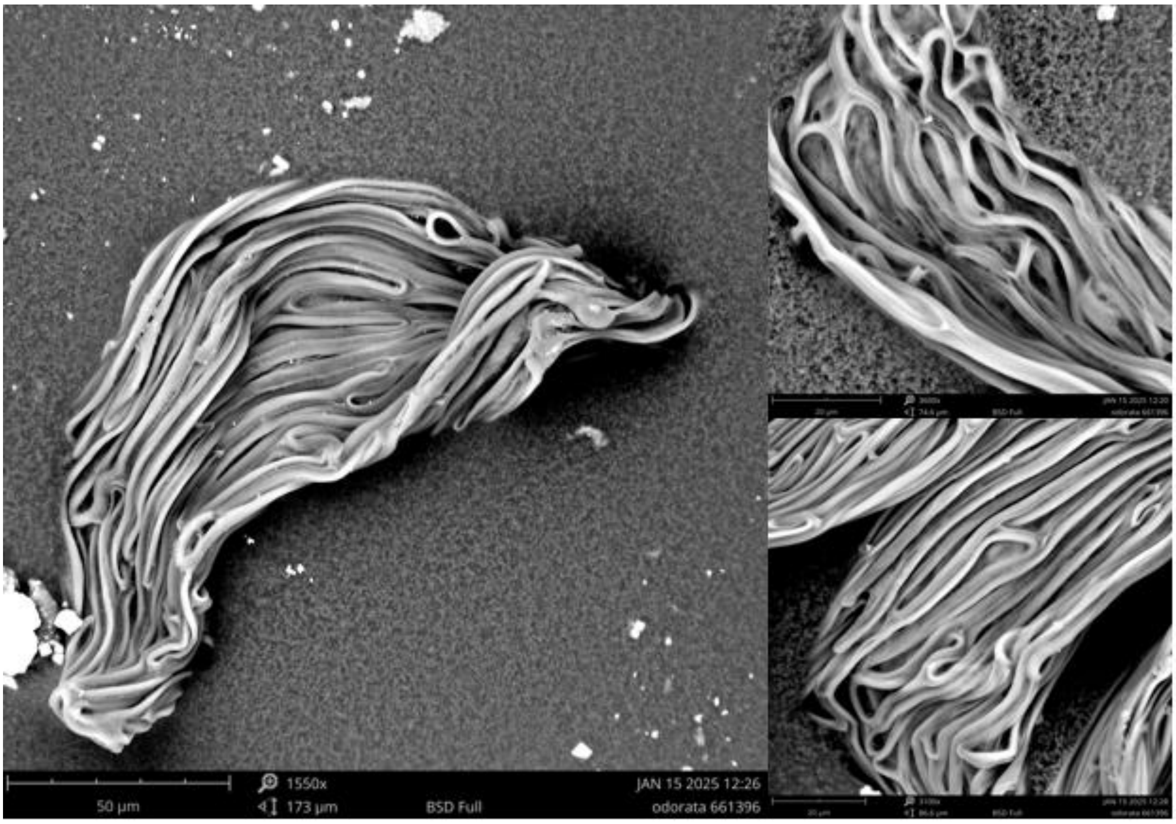

Fig.S127. *Polystachya odorata* Lindl. (P. odo4)

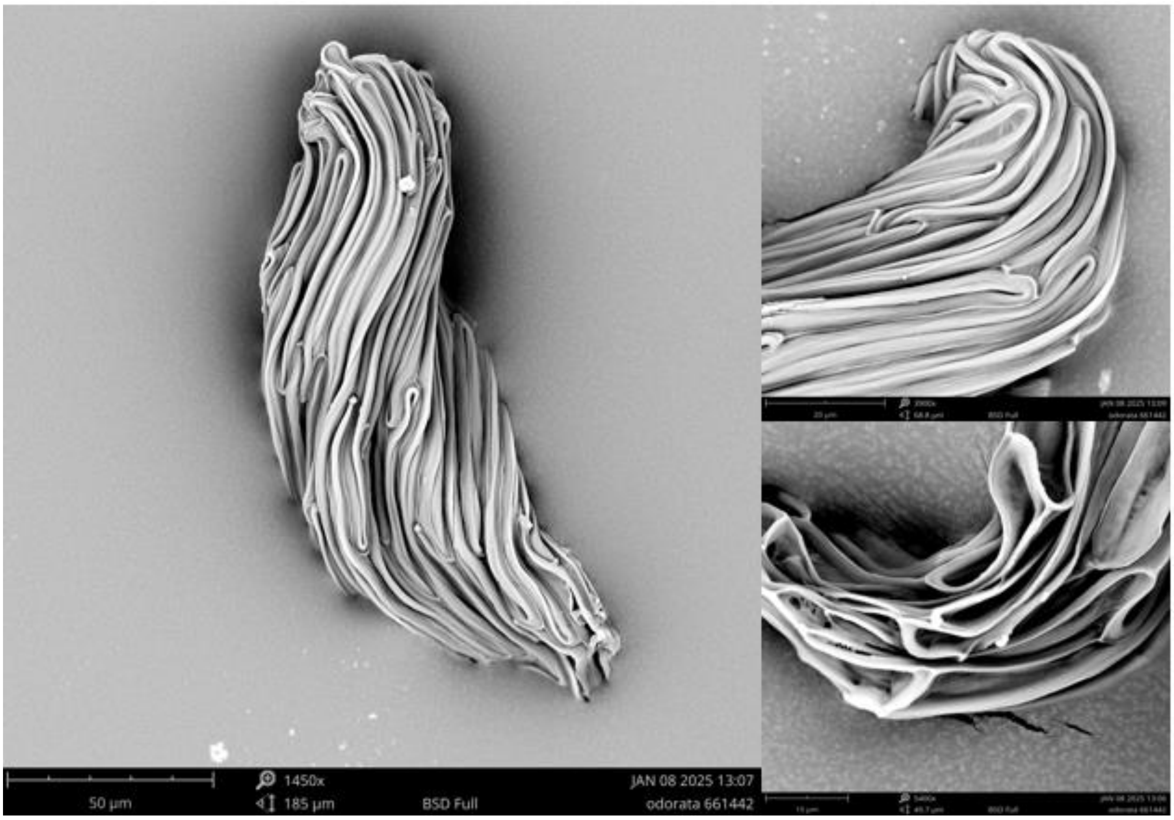

Fig.S128. *Polystachya odorata* Lindl. (P. odo5)

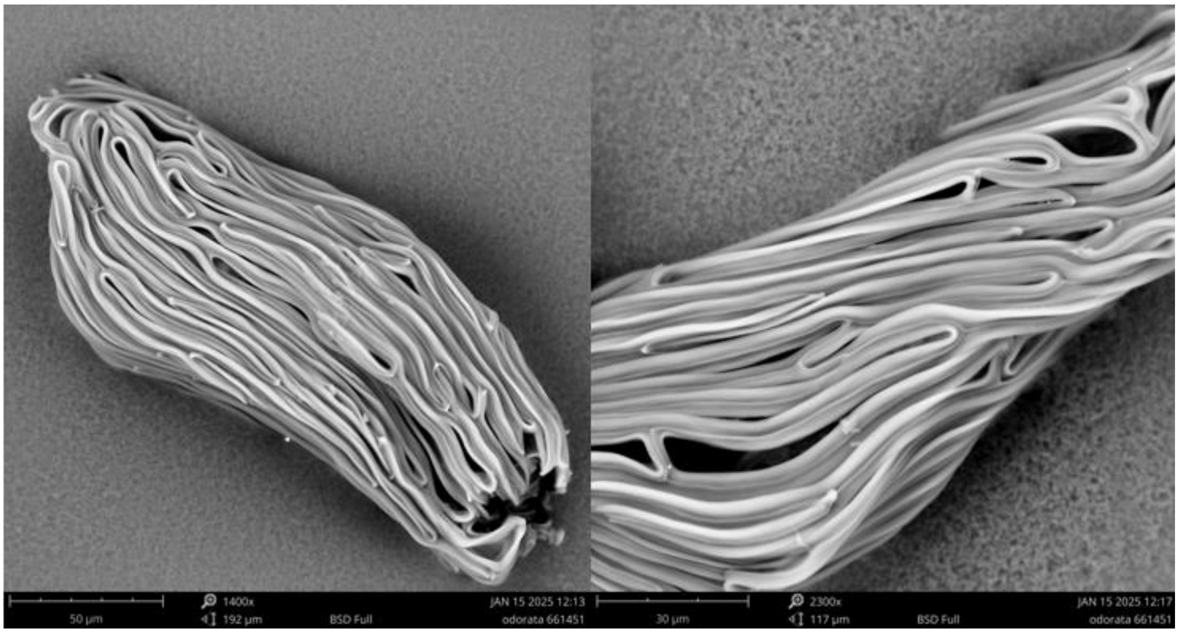

Fig.S129. *Polystachya odorata* Lindl. (P. odo6)

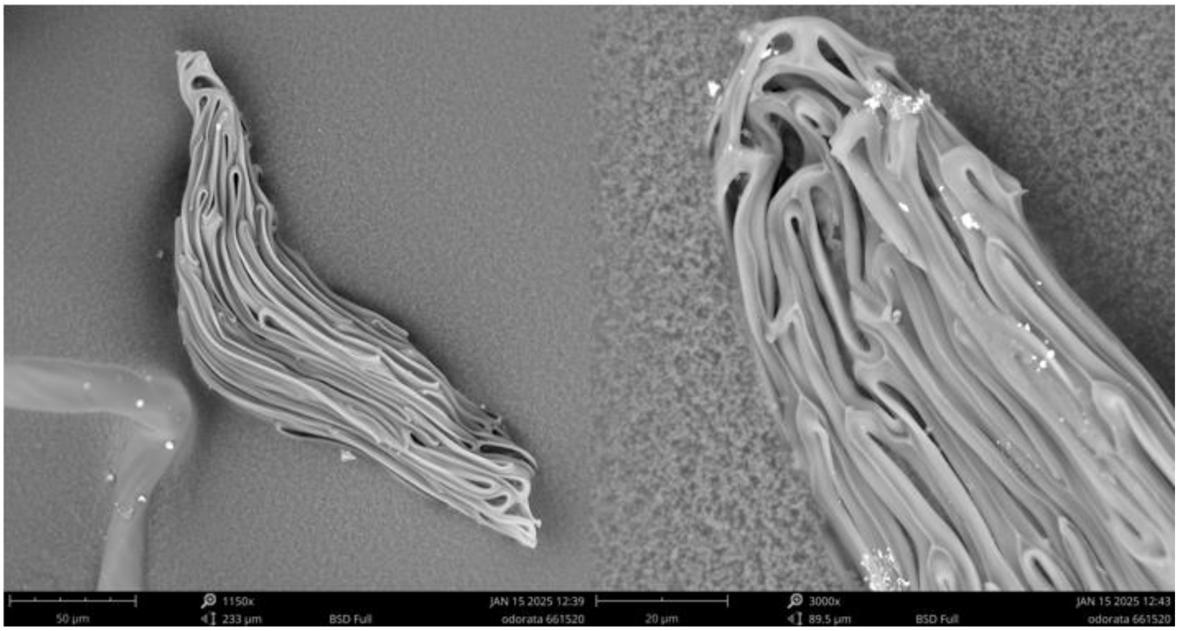

Fig.S130. *Polystachya odorata* Lindl. (P. odo7)

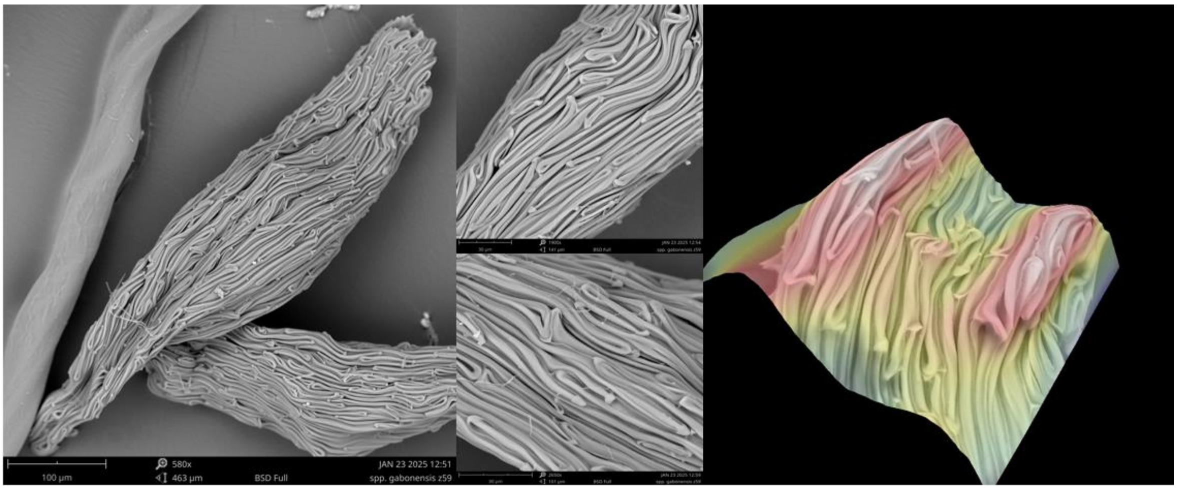

Fig.S131. *Polystachya odorata* ssp. *gabonensis* (Summerh.) Stévart (P. spp. gab)

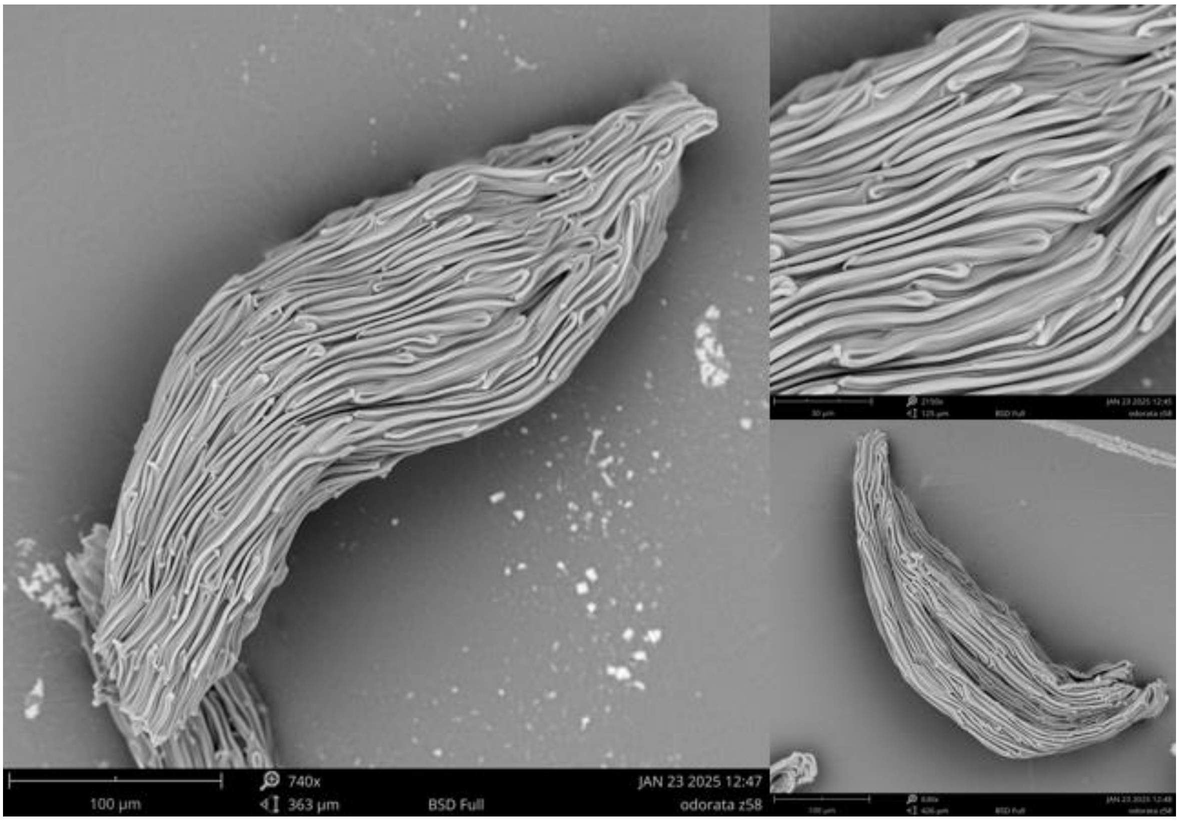

Fig.S132. *Polystachya odorata* ssp. *gabonensis* (Summerh.) Stévart (P. ssp. gab2)

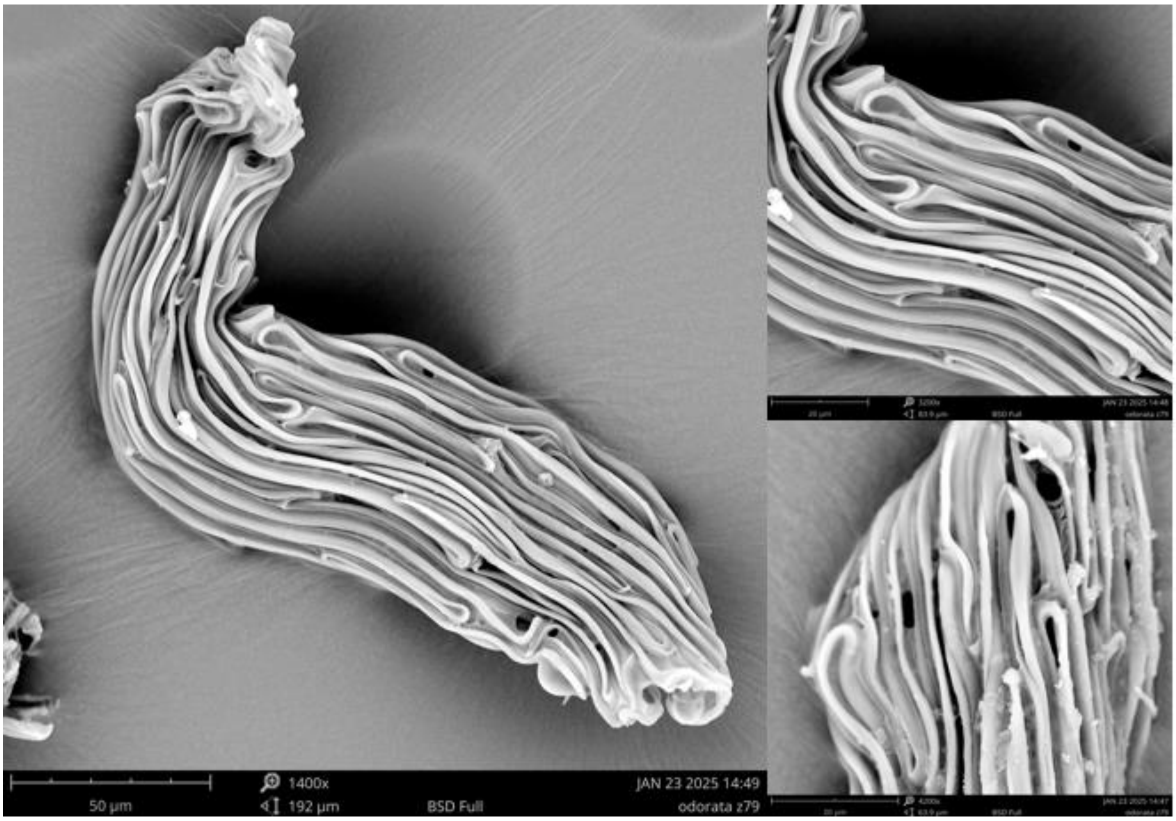

Fig.S133. *Polystachya odorata* ssp. *odorata* (P. odo. ssp. odo)

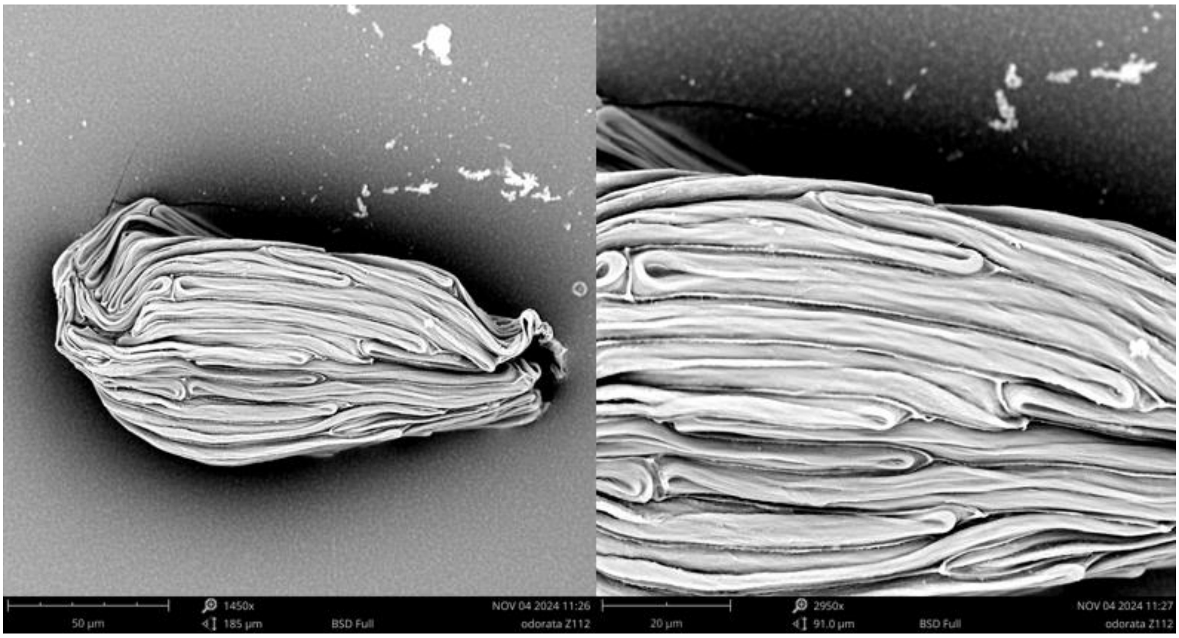

Fig.S134. *Polystachya odorata* ssp. *odorata* (P. odo. ssp. odo2)

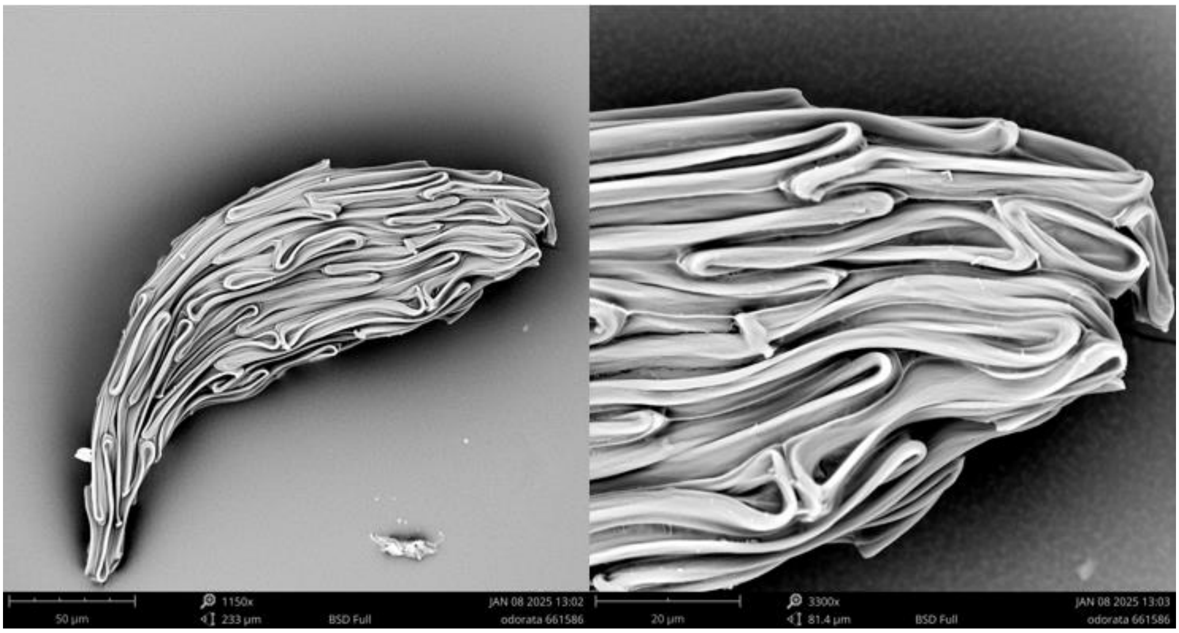

Fig.S135. *Polystachya odorata* ssp. *odorata* (P. odo. ssp. odo3)

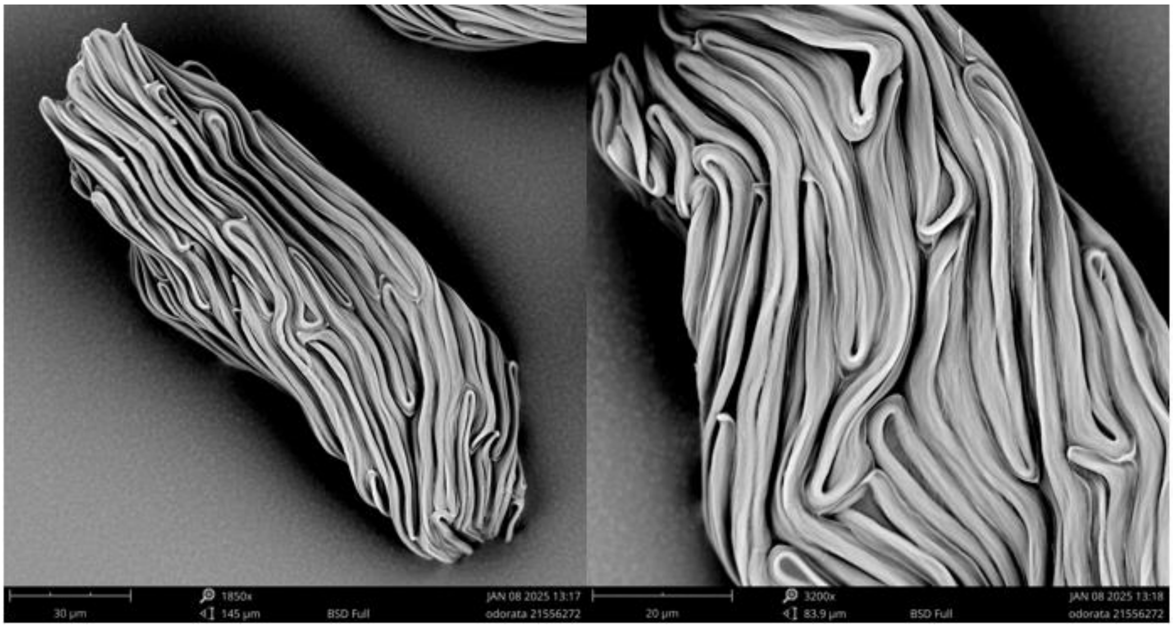

Fig.S136. *Polystachya odorata* ssp. *odorata* (P. odo. ssp. odo4)

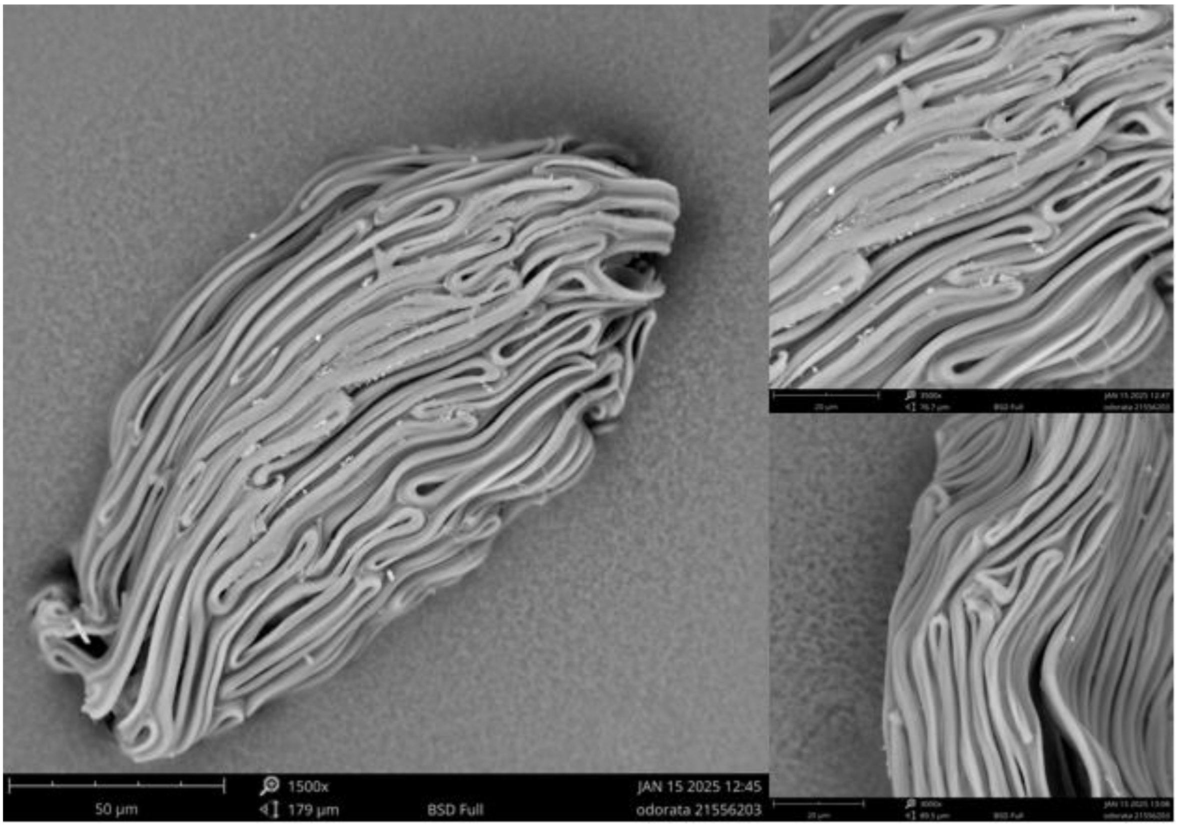

Fig.S137. *Polystachya odorata* ssp. *odorata* (P. odo. ssp. odo5)

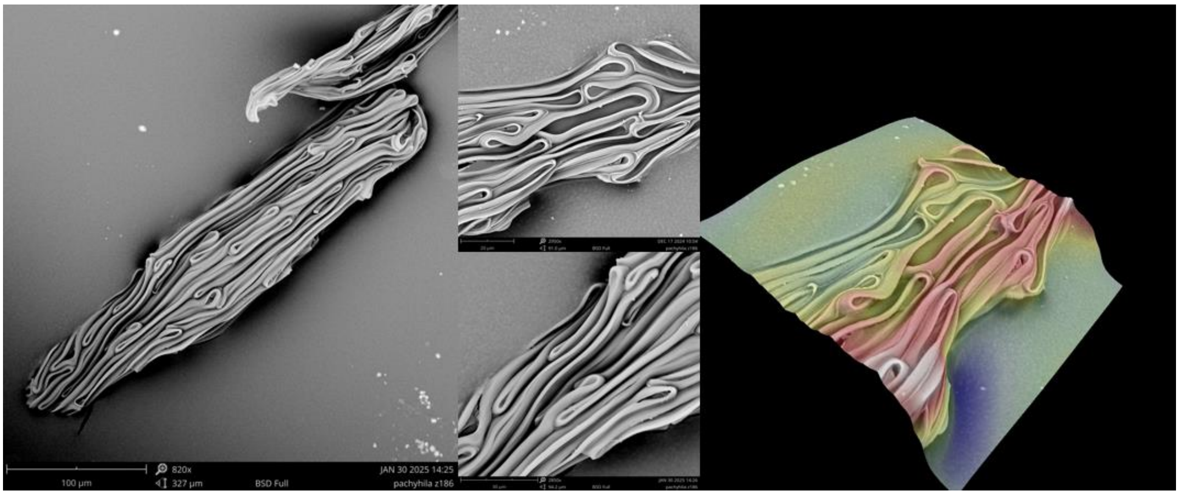

Fig.S138. *Polystachya pachychila* Summerh. (P. pach)

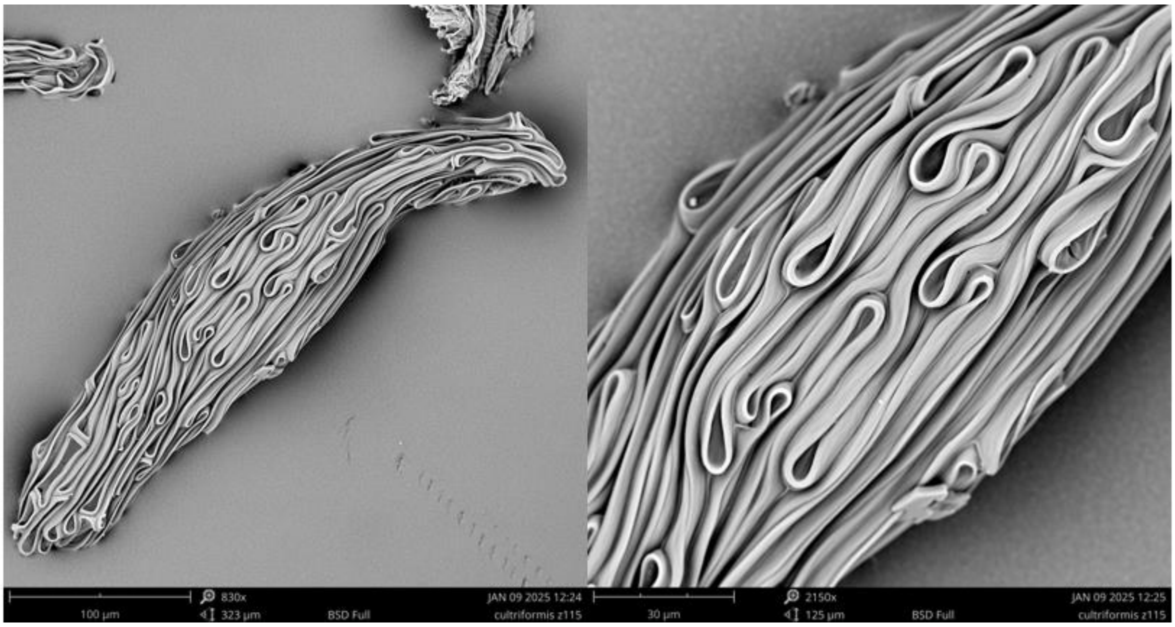

Fig.S139. *Polystachya pachychila* Summerh. (P. pach2)

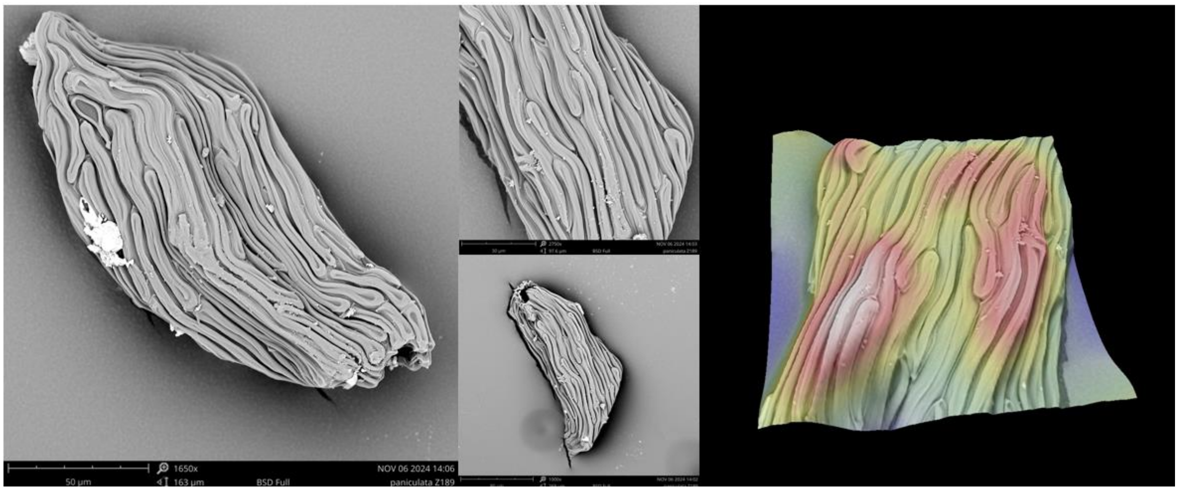

Fig.S140. *Polystachya paniculata* (Sw.) Rolfe (P. pan)

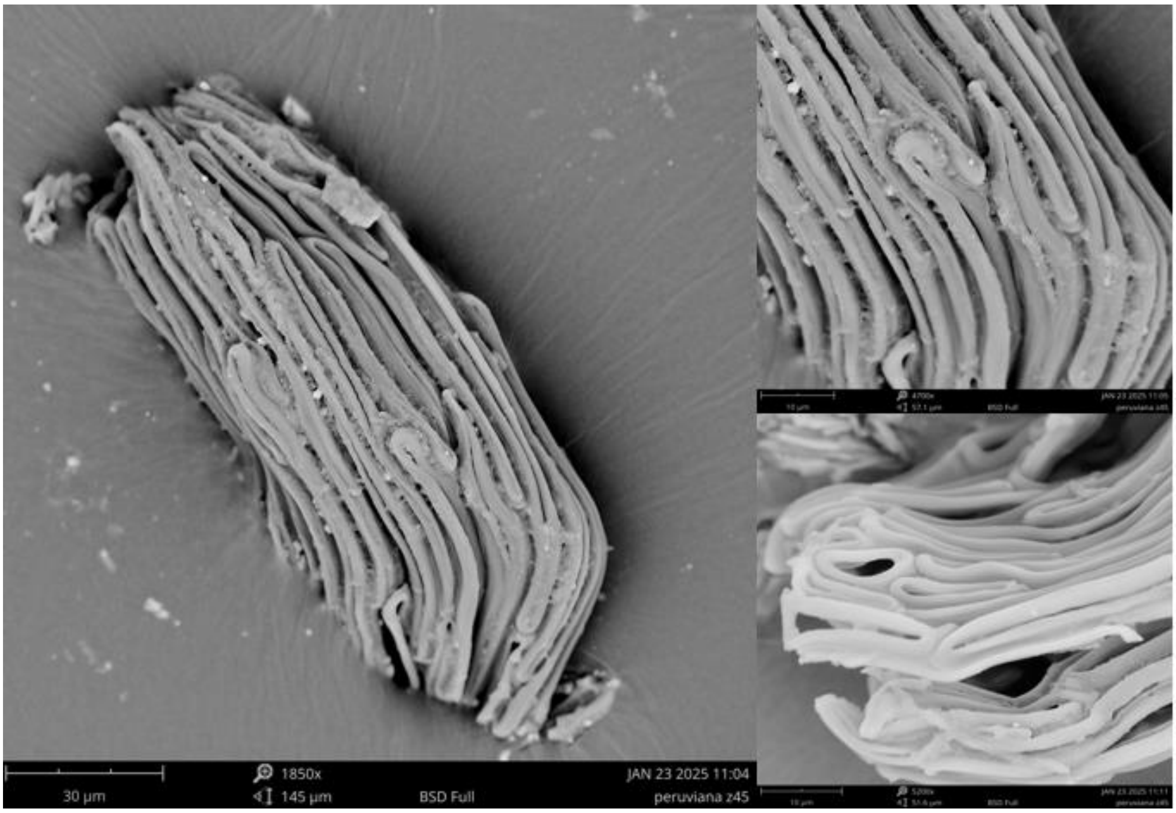

Fig.S141. *Polystachya peruviana* C.Nelson, J.Sutherl. & Fern.Casas (P. per)

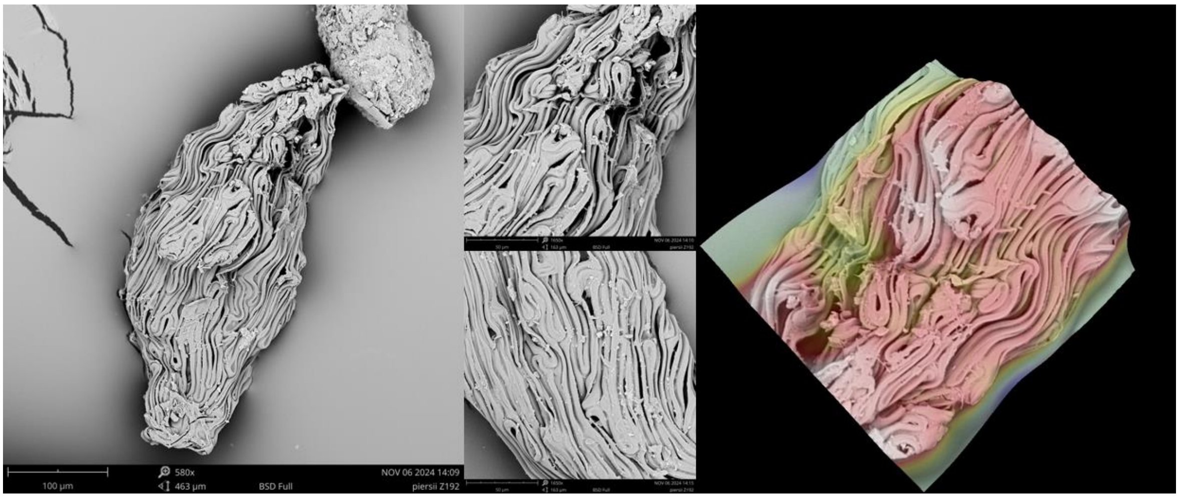

Fig.S142. *Polystachya piersii* P.J.Cribb (P. pie)

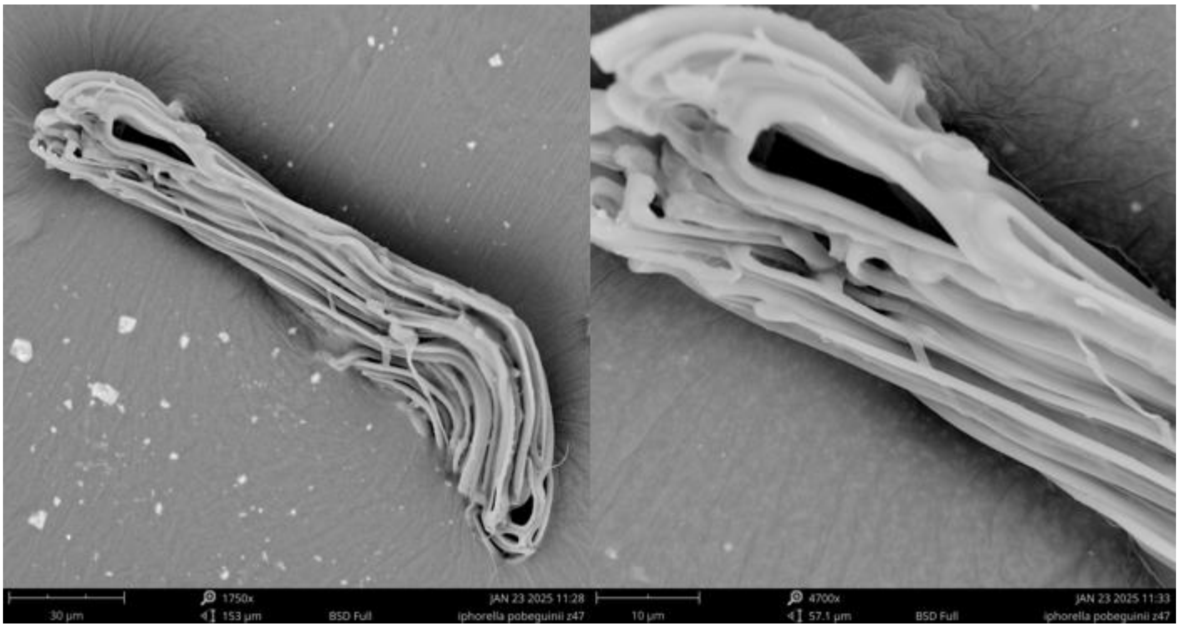

Fig.S143. *Polystachya pobeguinii* (Finet) Rolfe (P. pob)

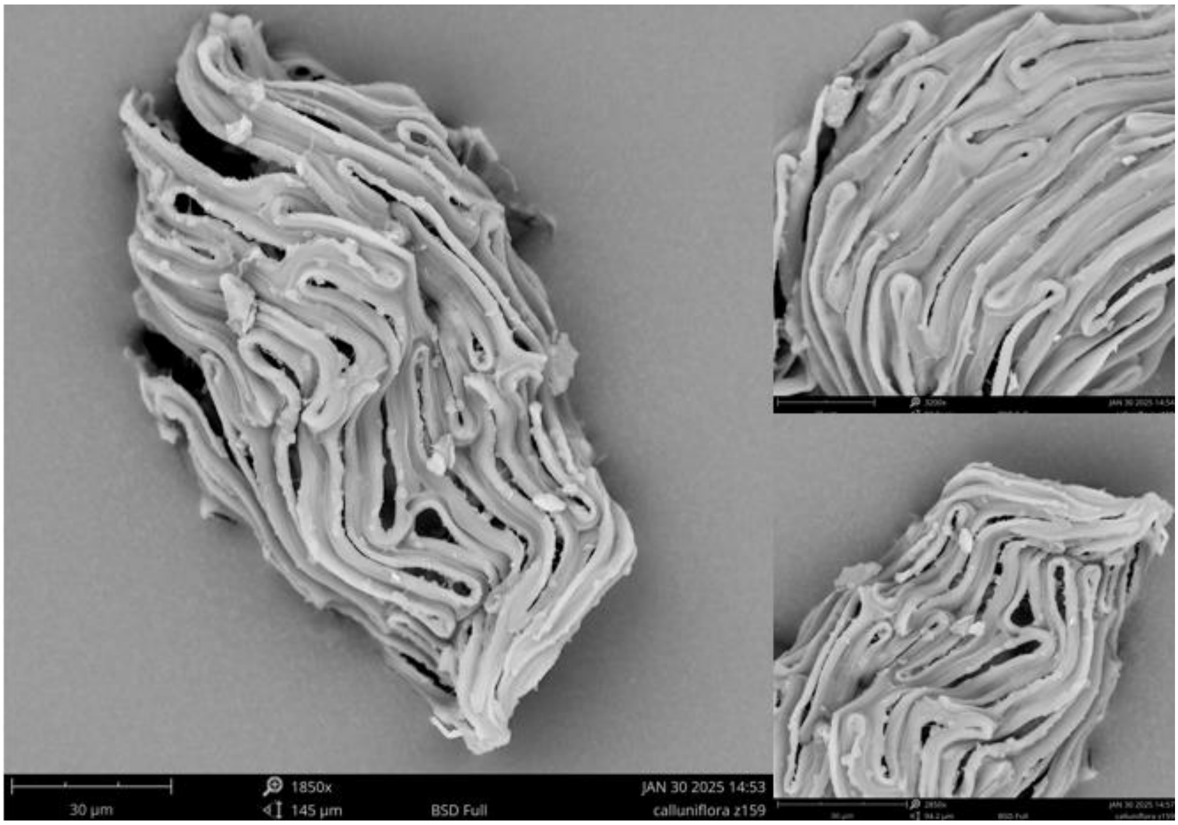

Fig.S144. *Polystachya poikilantha* var. *poikilantha* (P. poi. var. poi)

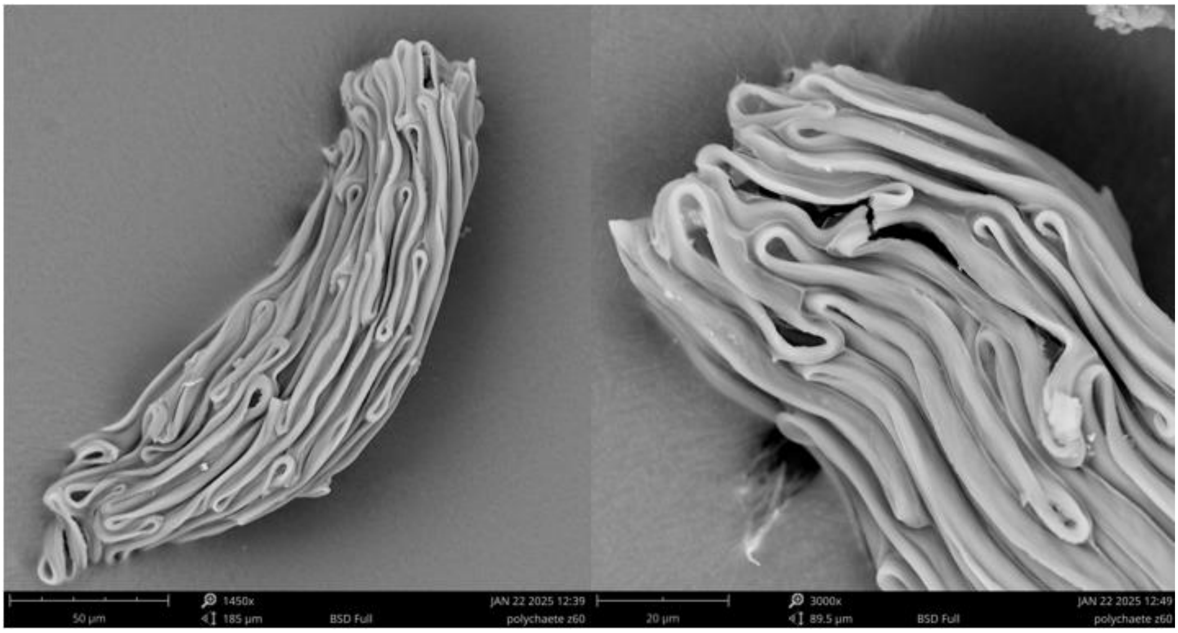

Fig.S145. *Polystachya polychaete* Kraenzl. (P. pol2)

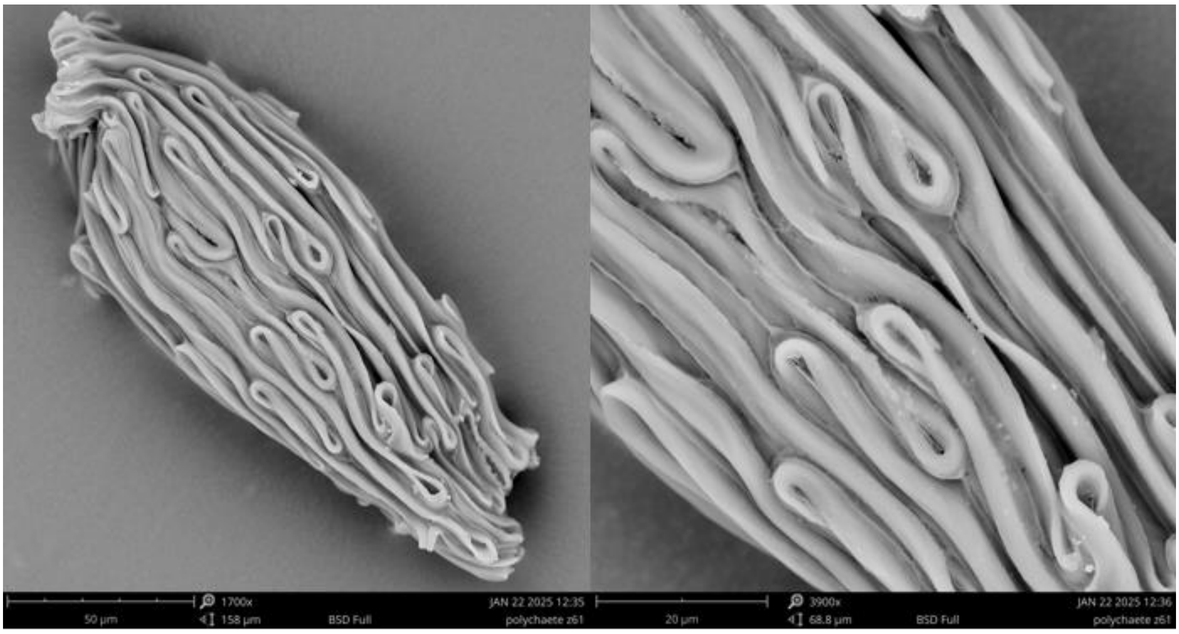

Fig.S146. *Polystachya polychaete* Kraenzl. (P. pol3)

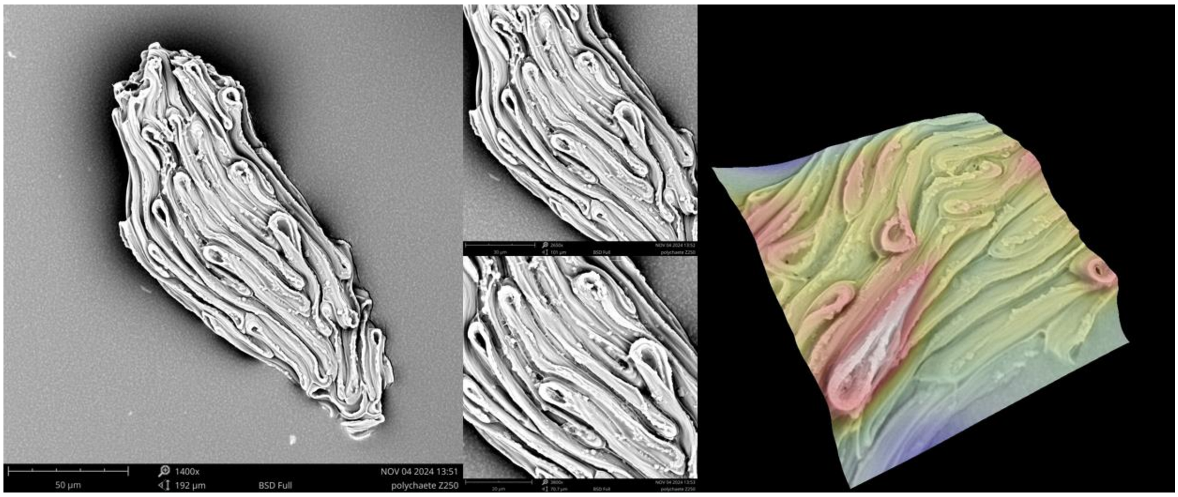

Fig.S147. *Polystachya polychaete* Kraenzl. (P. pol4)

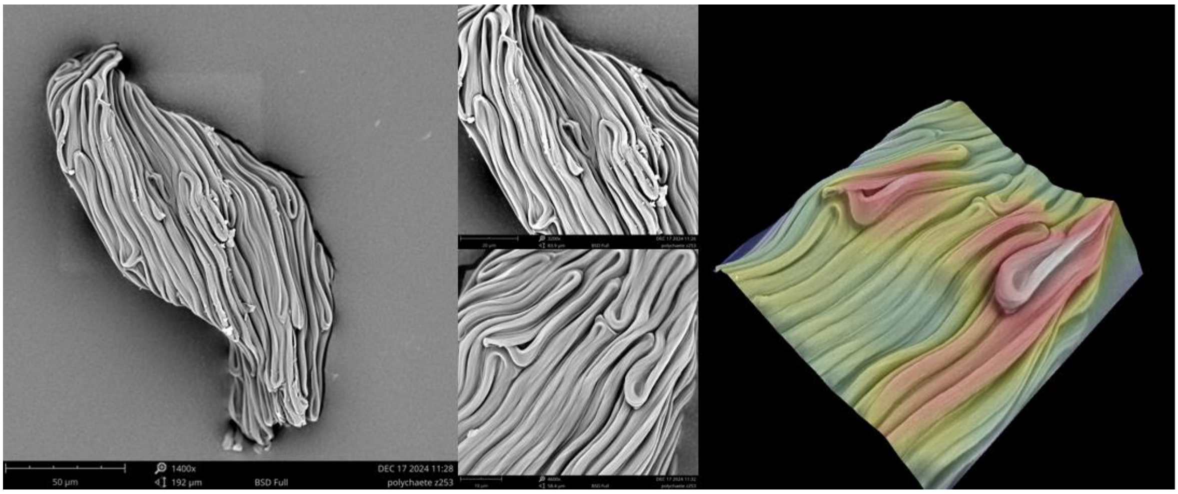

Fig.S148. *Polystachya polychaete* Kraenzl. (P. pol6)

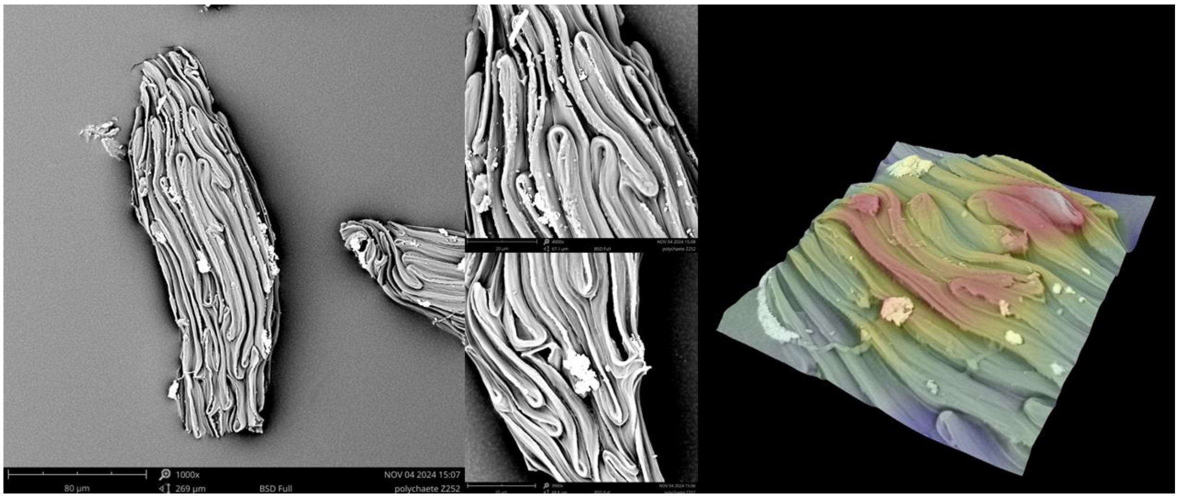

Fig.S149. *Polystachya polychaete* Kraenzl. (P. pol7)

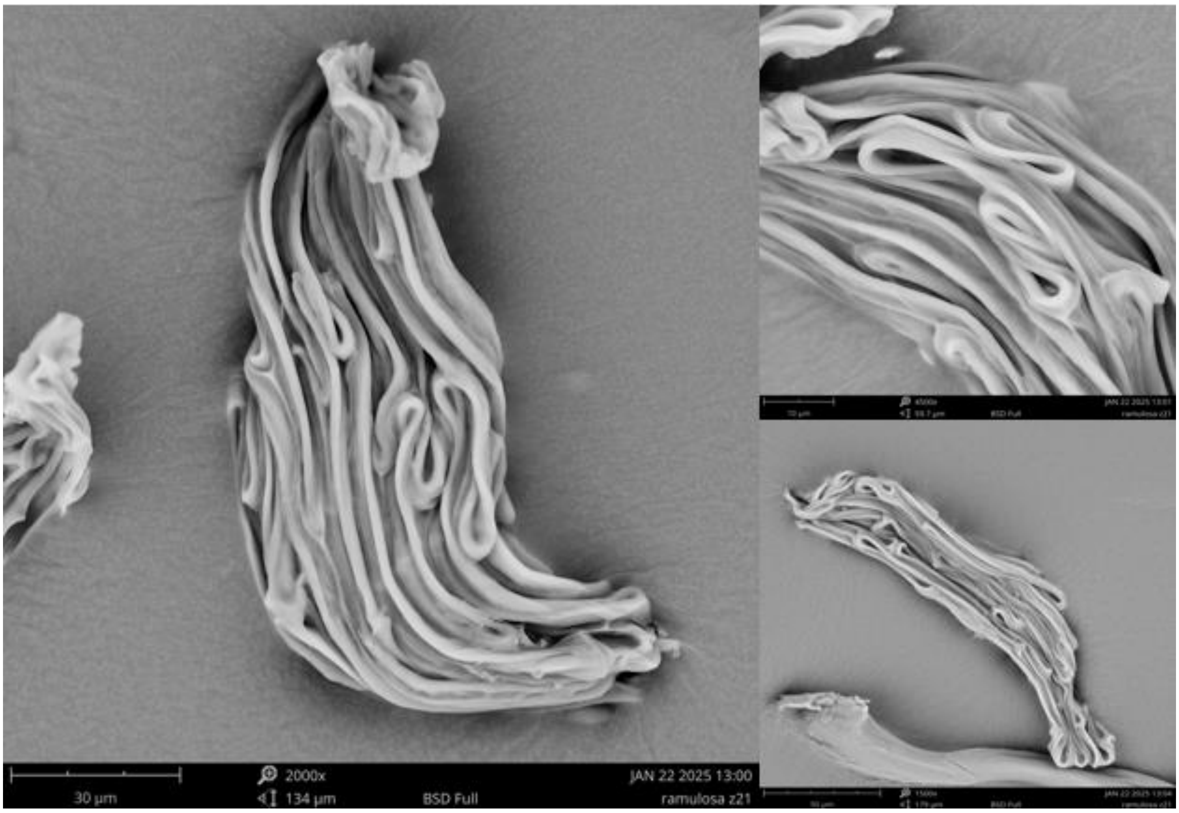

Fig.S150. *Polystachya ramulosa* Lindl. (P. ram)

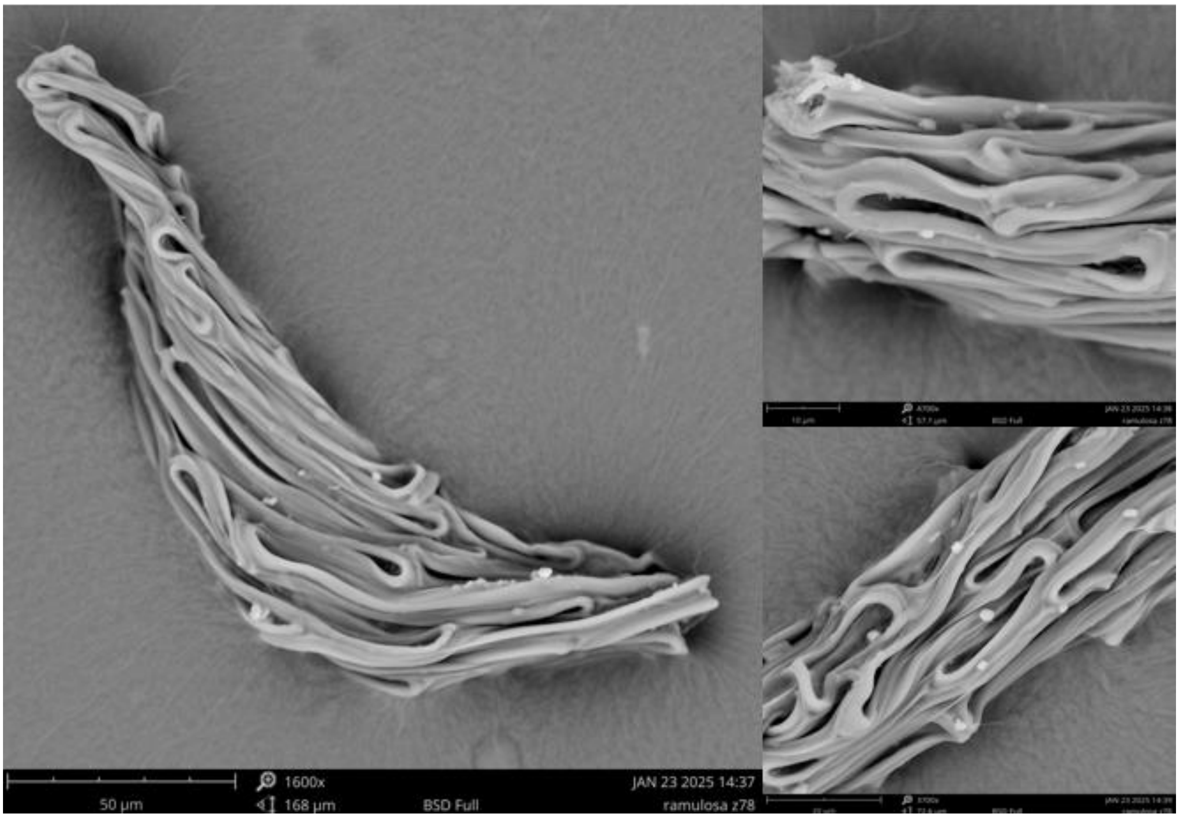

Fig.S151. *Polystachya ramulosa* Lindl. (P. ram3)

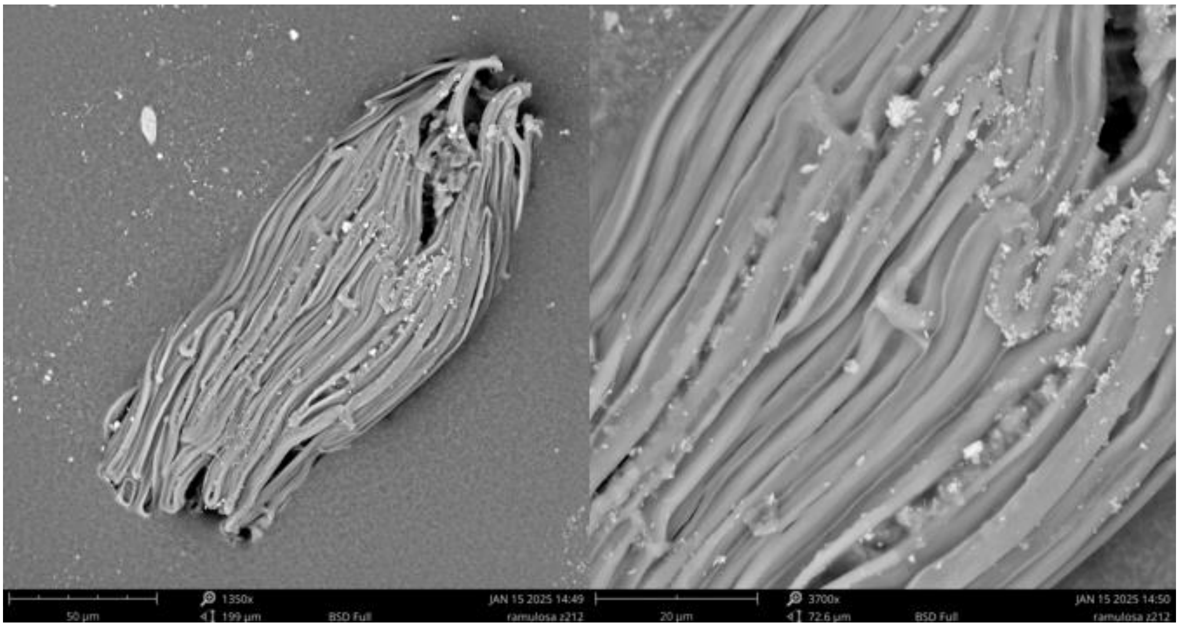

Fig.S152. *Polystachya ramulosa* Lindl. (P. ram4)

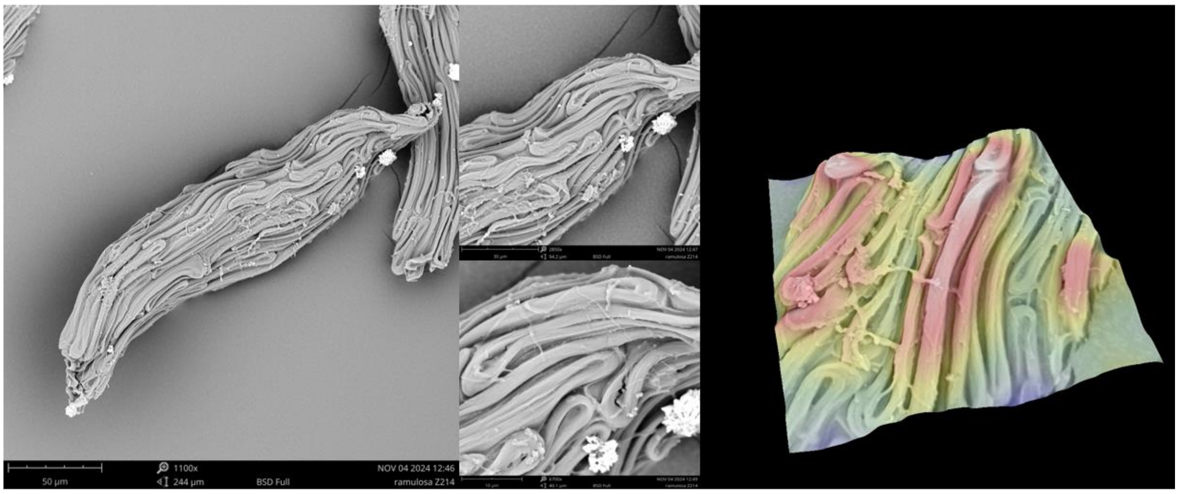

Fig.S153. *Polystachya ramulosa* Lindl. (P. ram5)

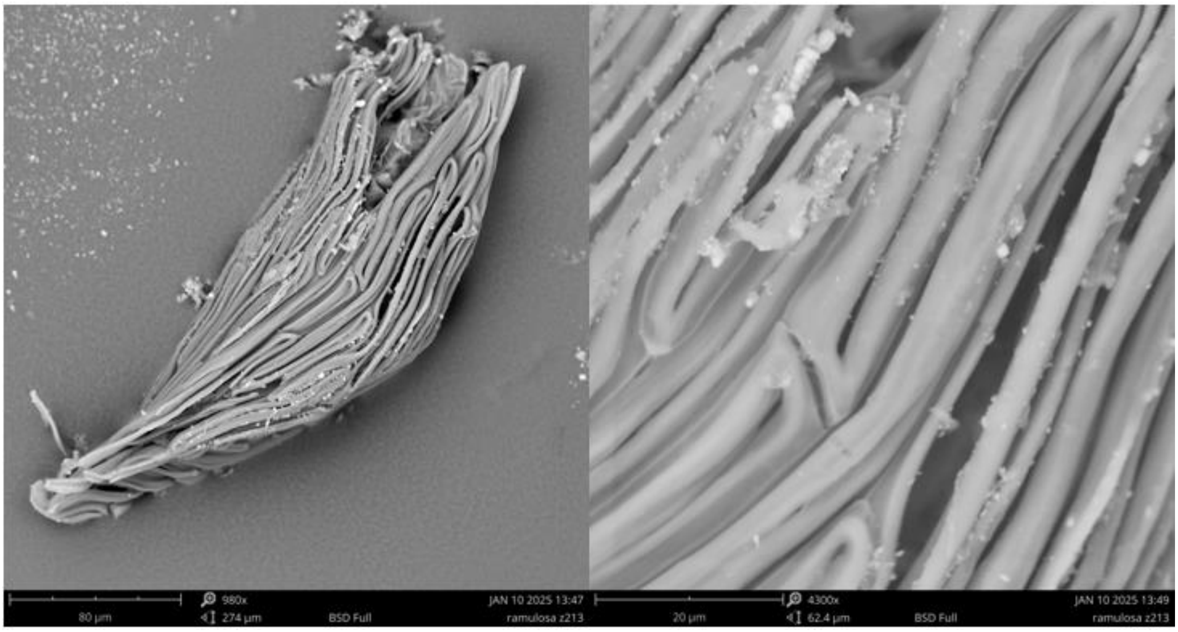

Fig.S154. *Polystachya ramulosa* Lindl. (P. ram6)

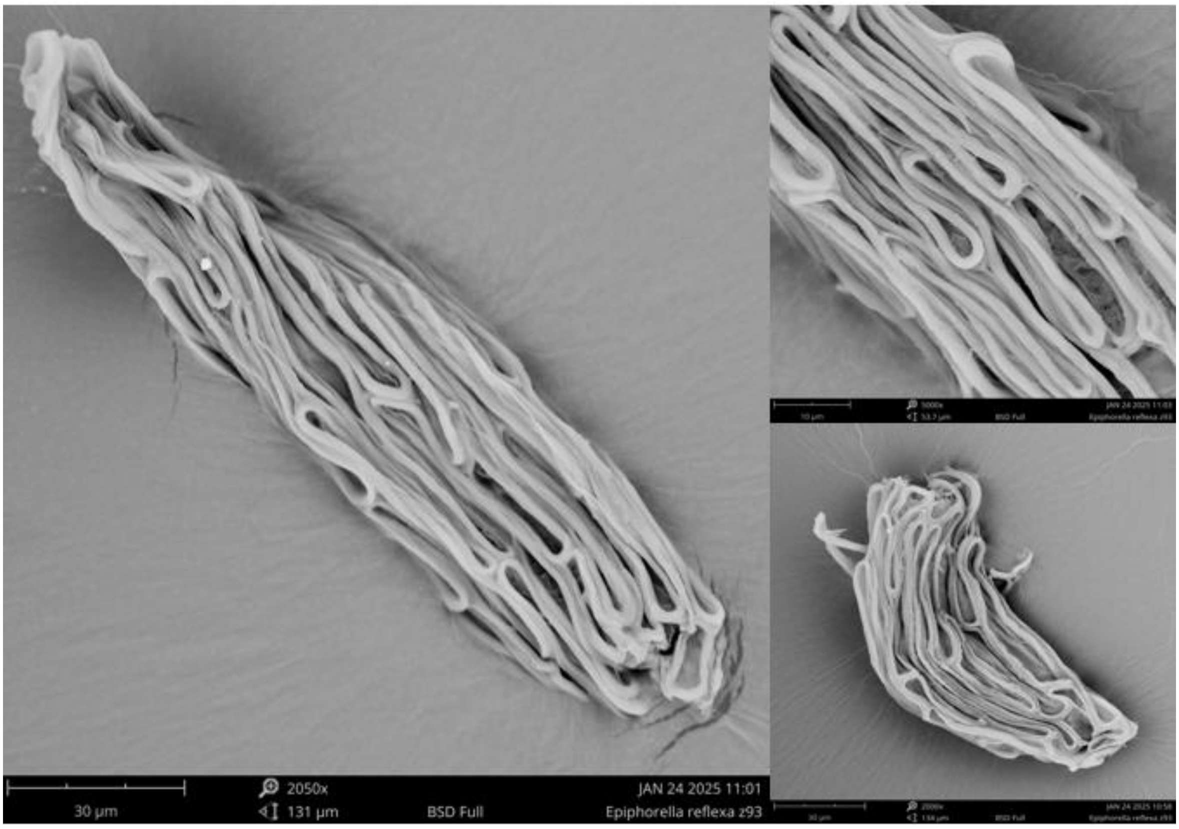

Fig.S155. *Polystachya reflexa* Lindl. (P. ref2)

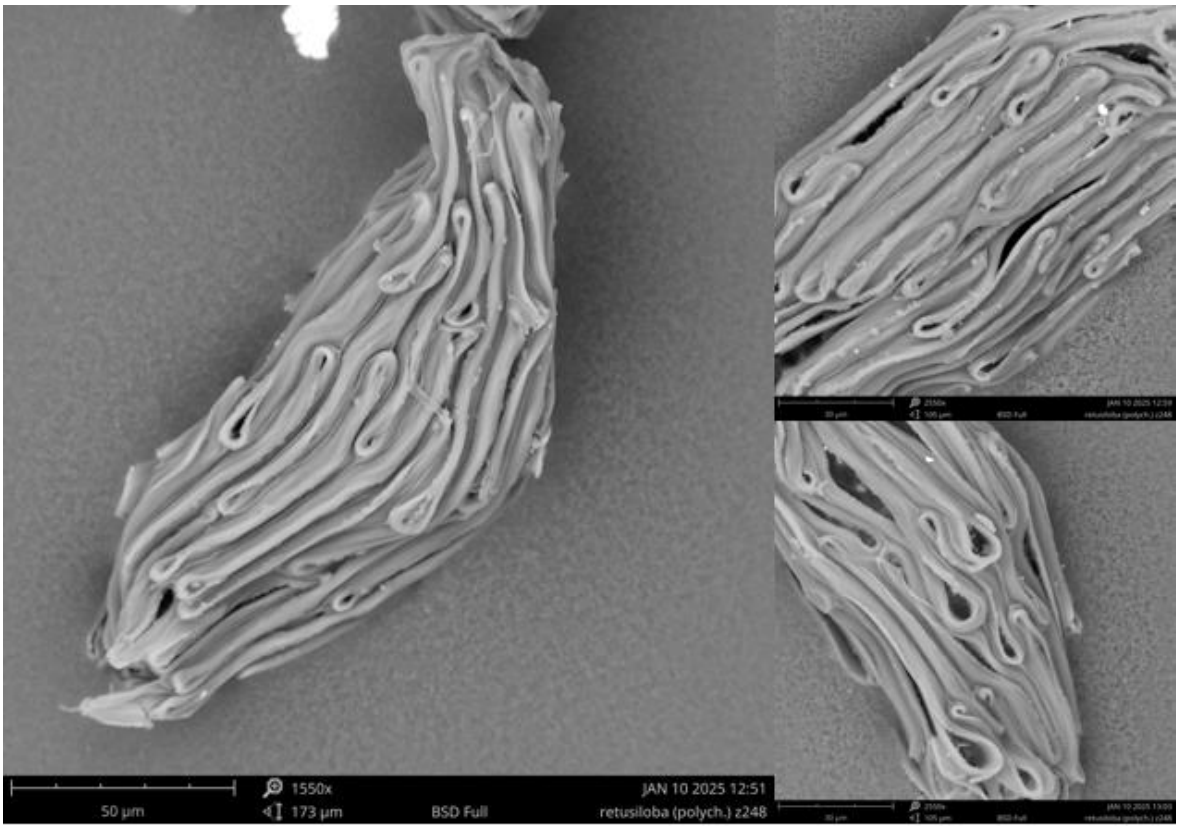

Fig.S156. *Polystachya retusiloba* Summerh. (P. ret)

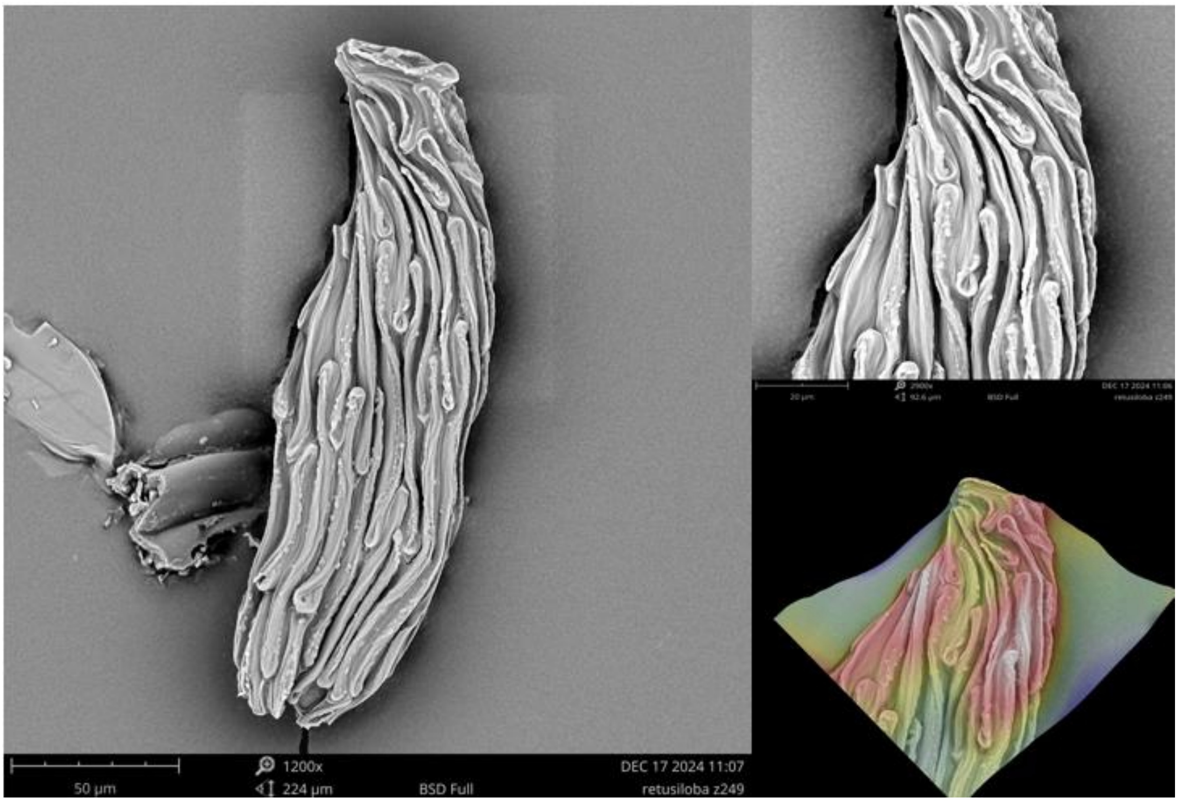

Fig.S157. *Polystachya retusiloba* Summerh. (P. ret2)

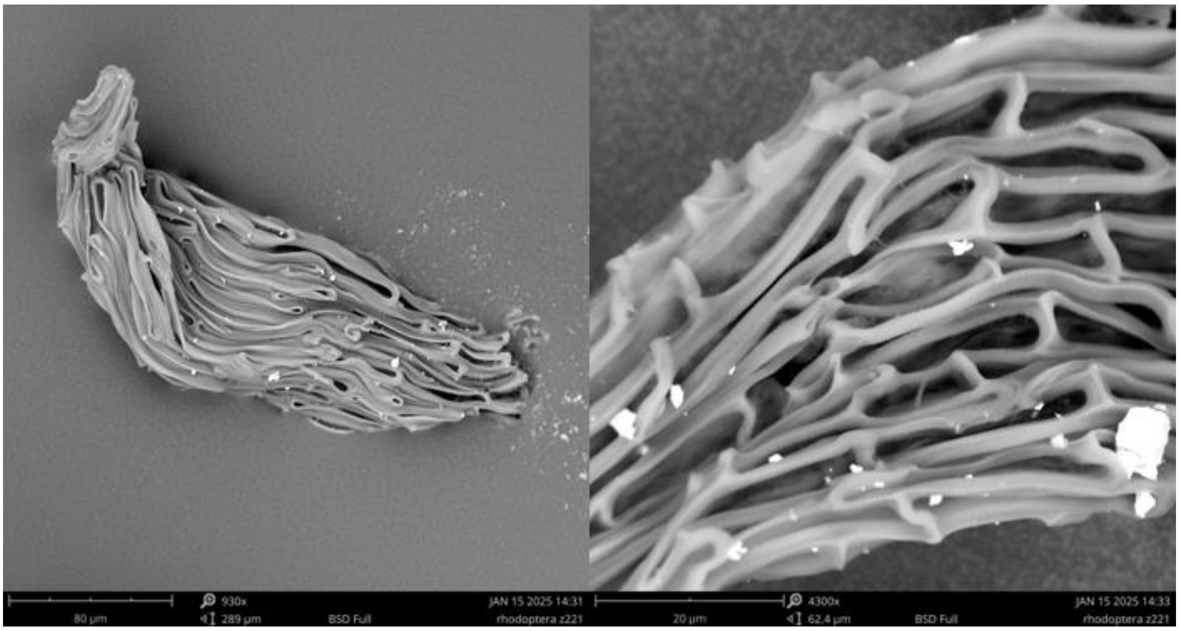

Fig.S158. *Polystachya rhodoptera* Rchb.f. (P. rho)

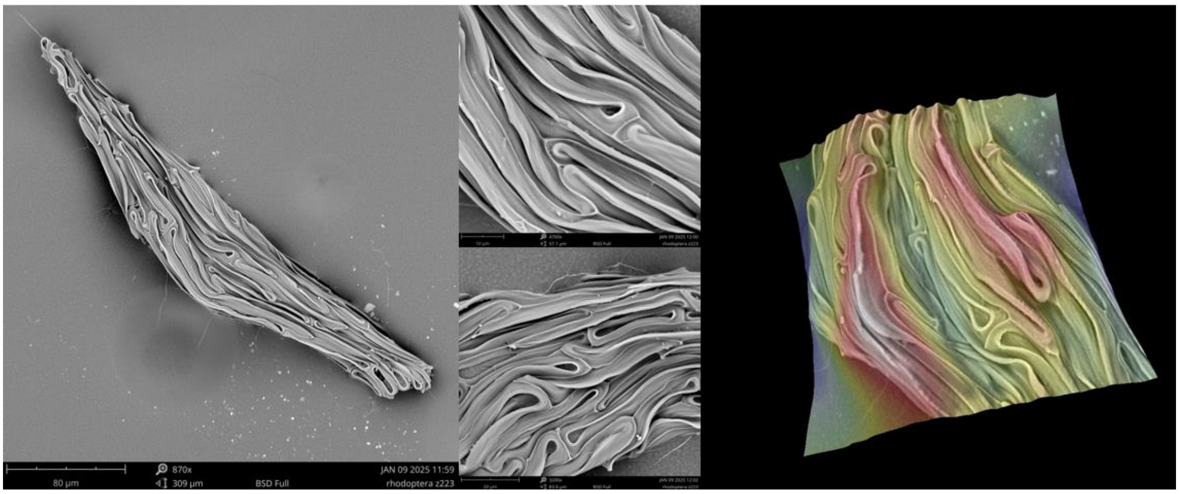

Fig.S159. *Polystachya rhodoptera* Rchb.f. (P. rho3)

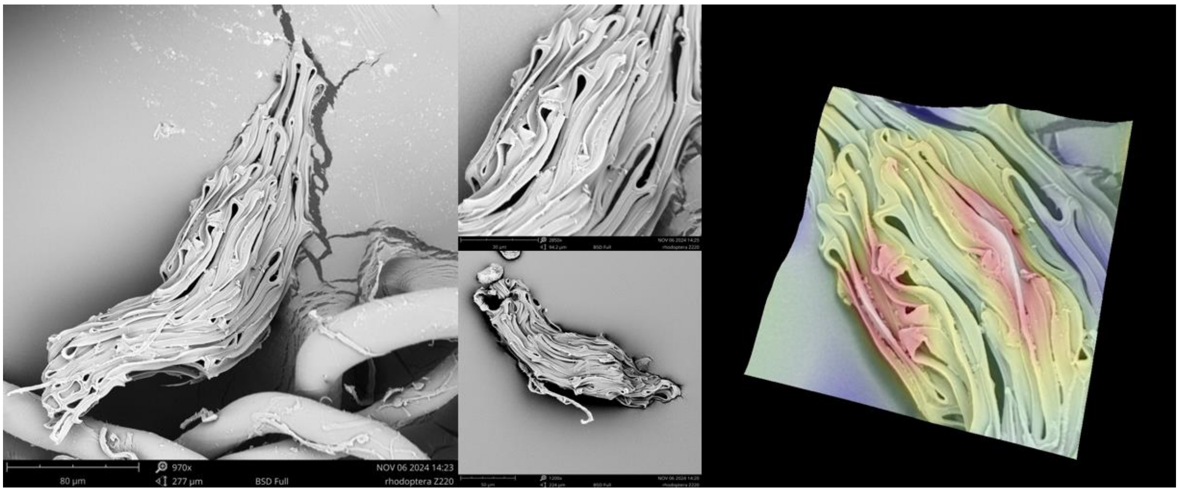

Fig.S160. *Polystachya rhodoptera* Rchb.f. (P. rho4)

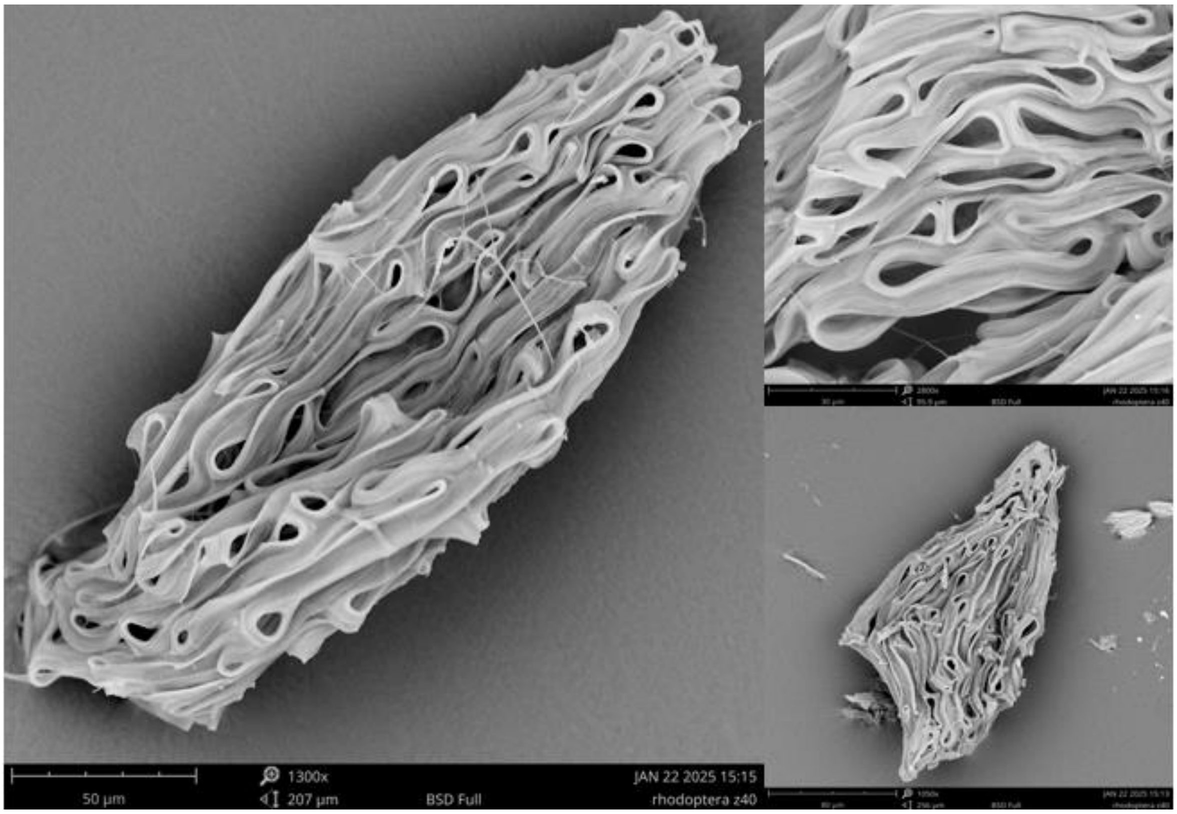

Fig.S161. *Polystachya rhodoptera* Rchb.f. (P. rho5)

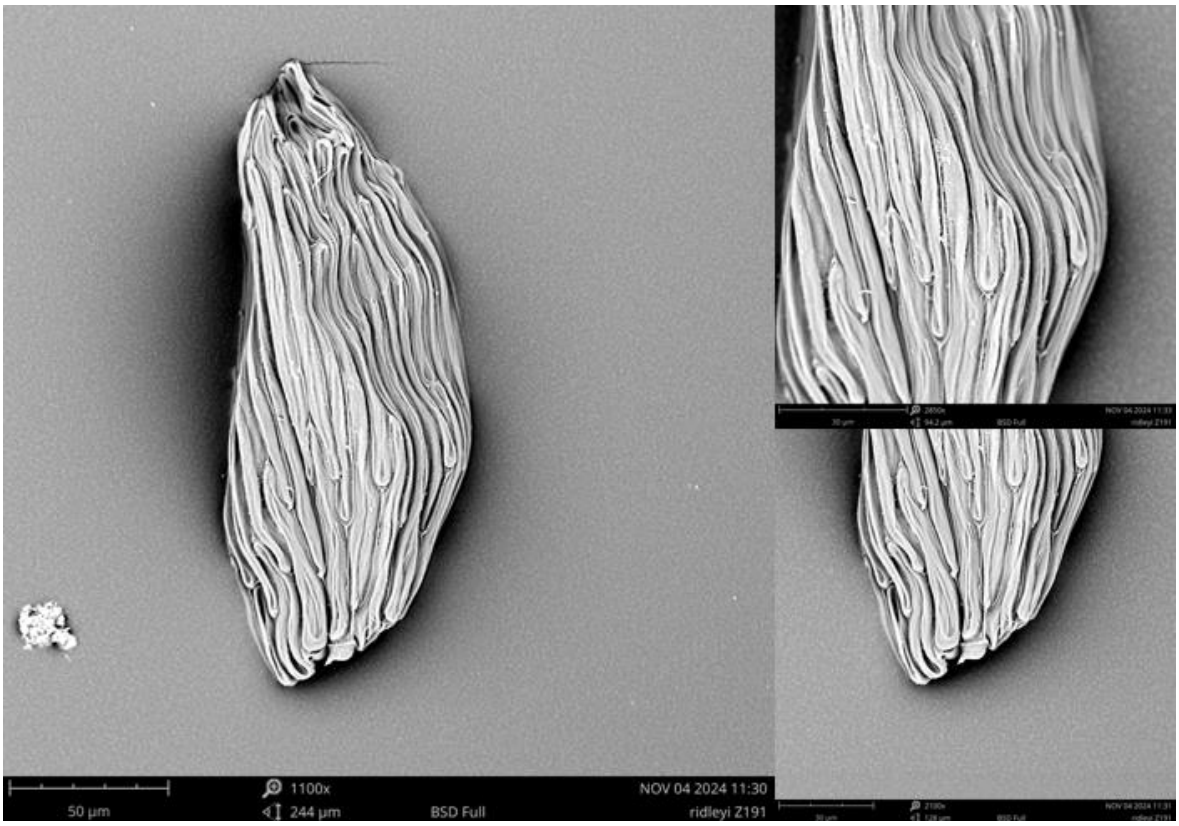

Fig.S162. *Polystachya ridleyi* Rolfe (P. rid)

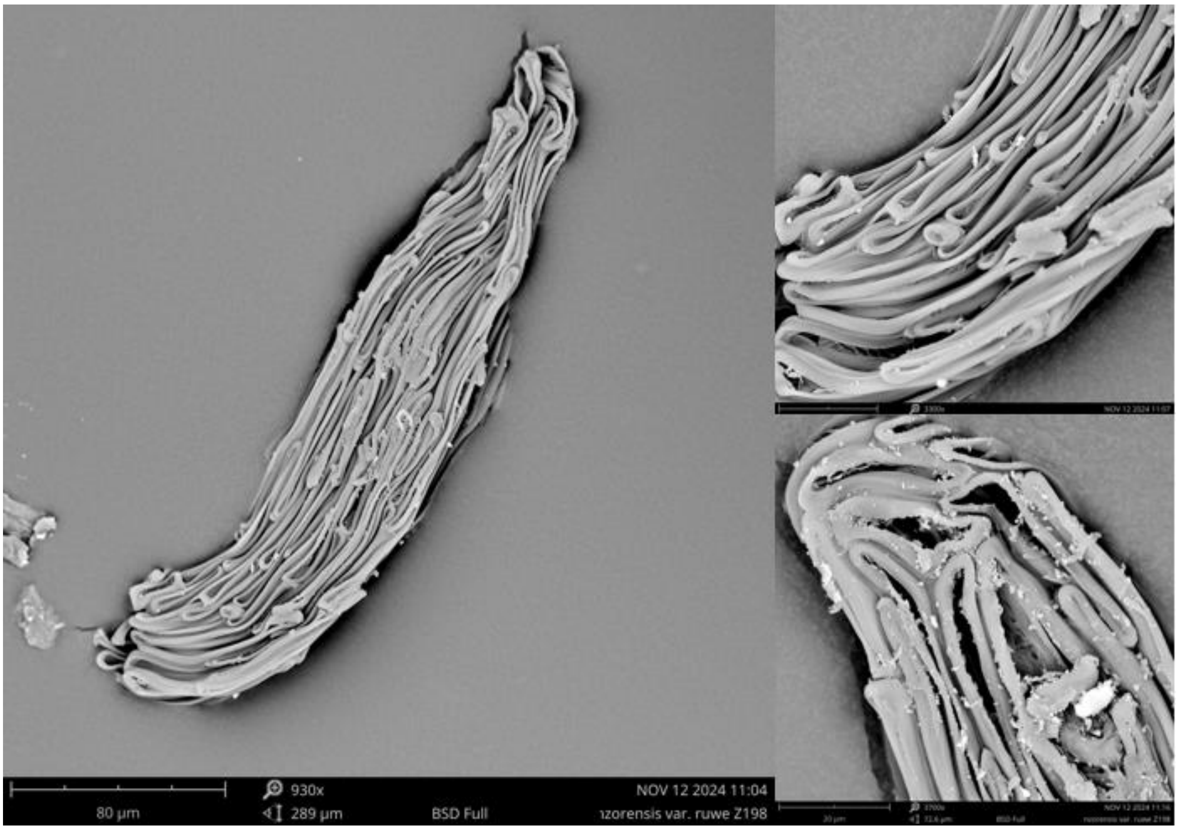

Fig.S163. *Polystachya ruwenzoriensis* Rendle (P. ruw)

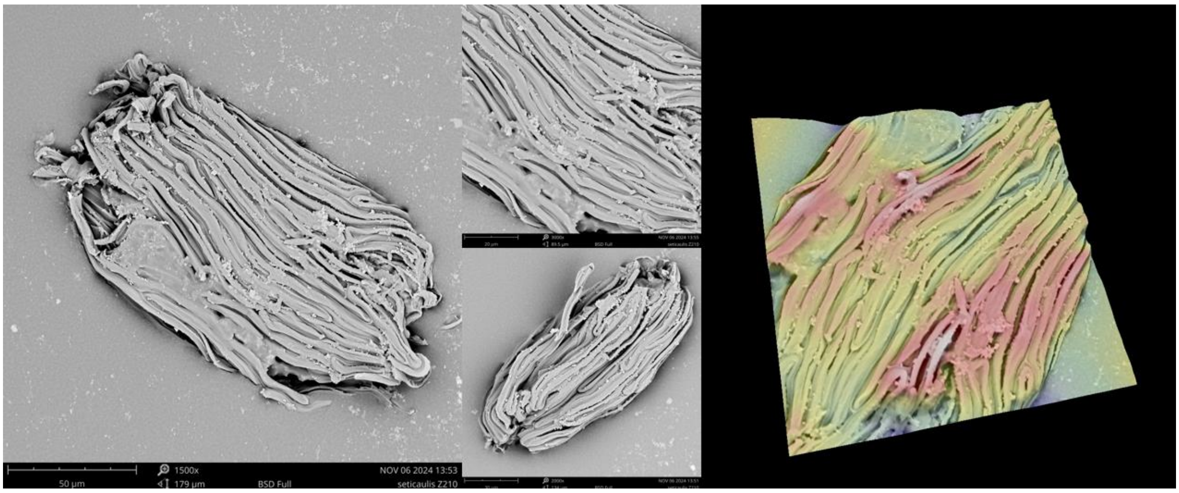

Fig.S164. *Polystachya seticaulis* Rendle (P. set2)

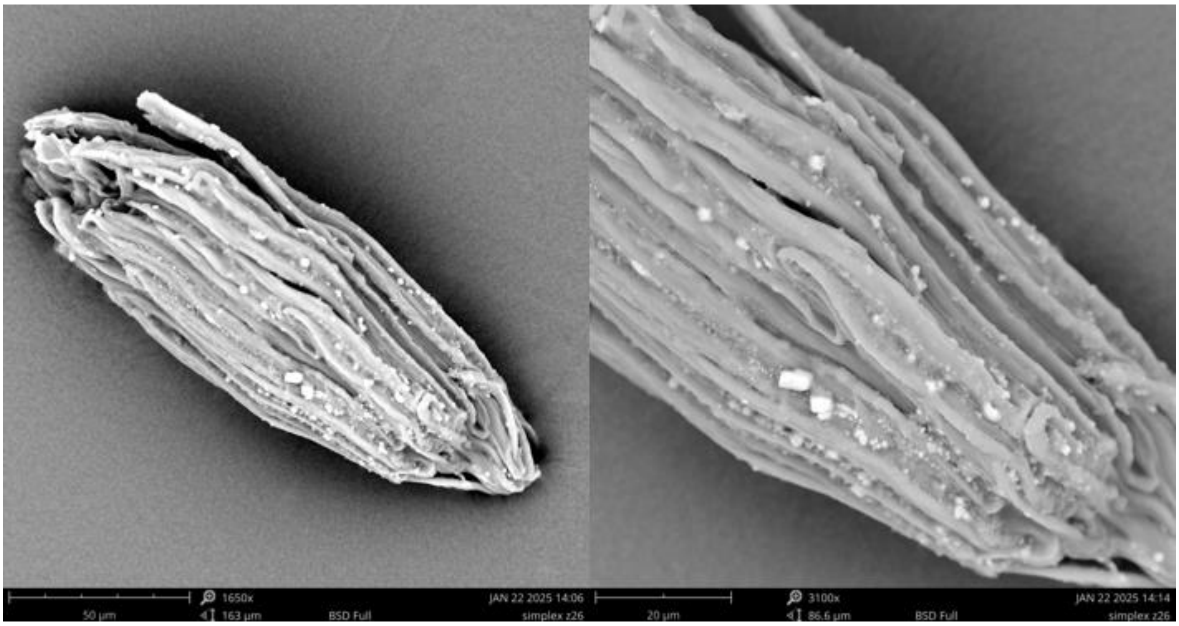

Fig.S165. *Polystachya simplex* Rendle (P. sim)

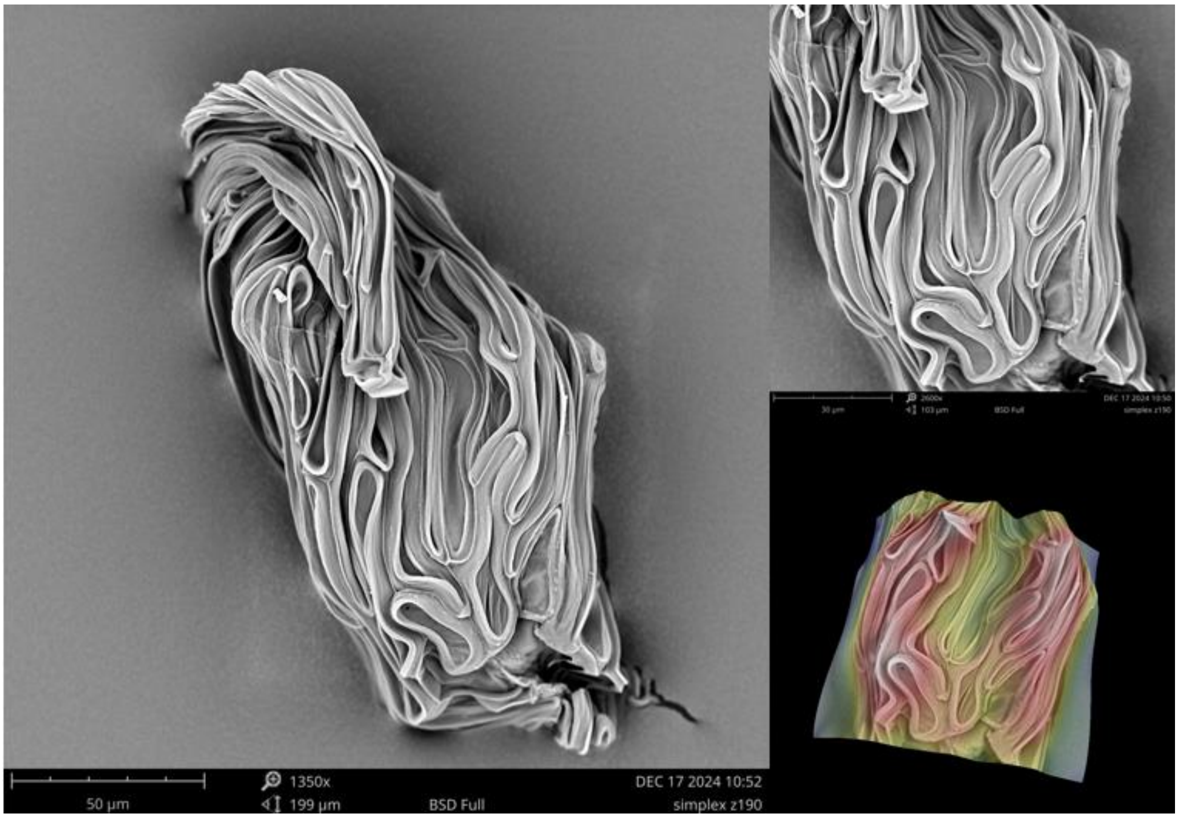

Fig.S166. *Polystachya simplex* Rendle (P. sim2)

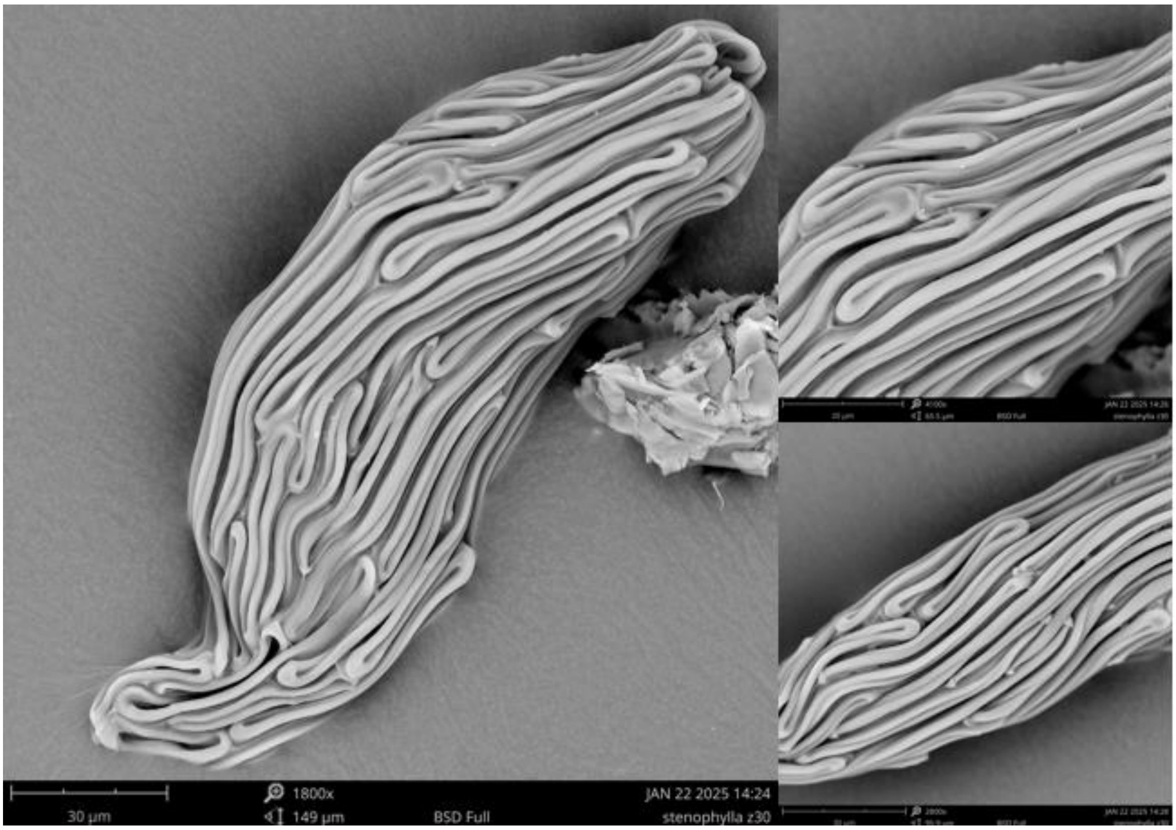

Fig.S167. *Polystachya stenophylla* Schltr. (P. ste)

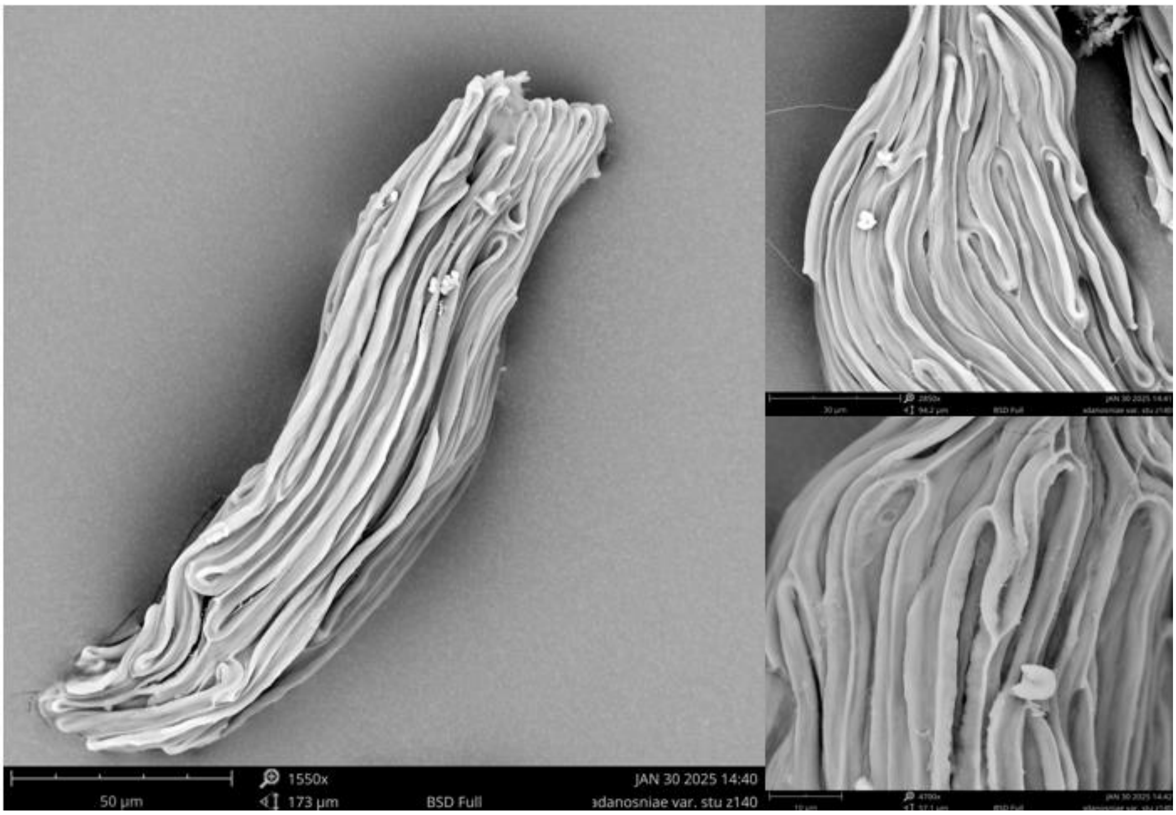

Fig.S168. *Polystachya stuhlmannii* Kraenzl. (P. stu)

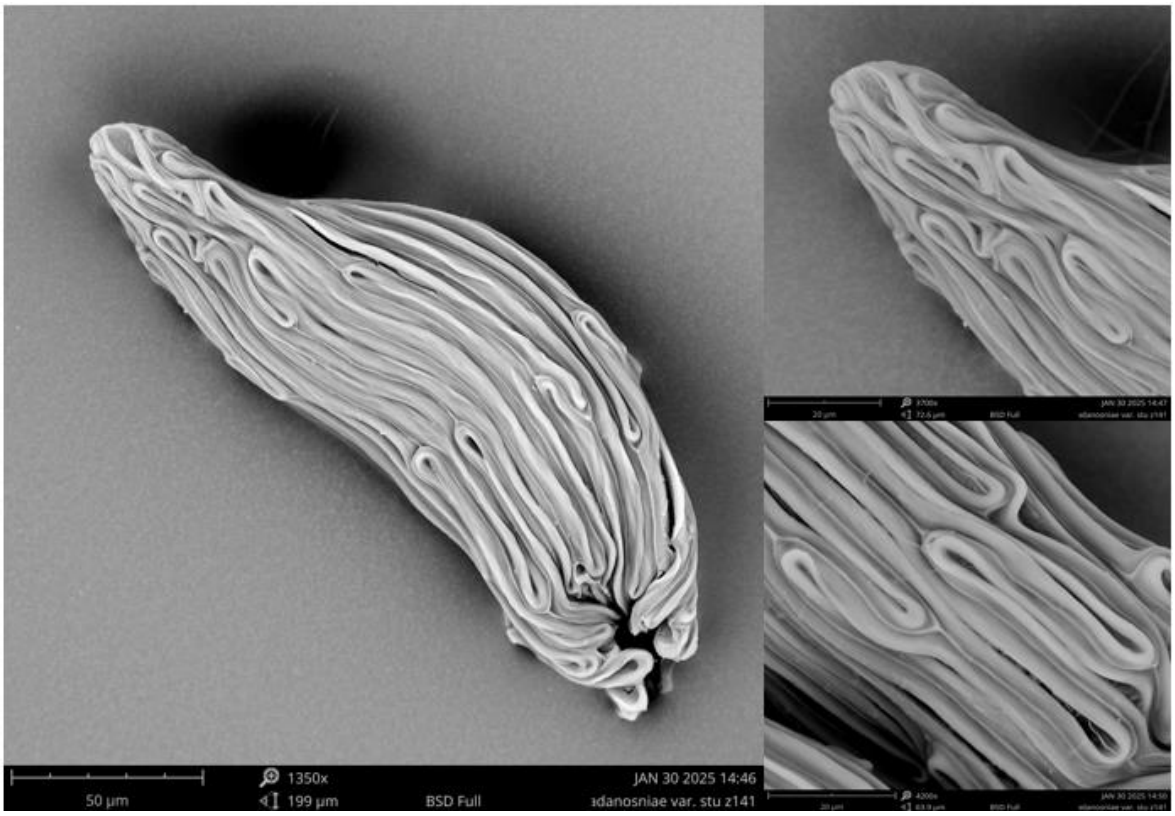

Fig.S169. *Polystachya stuhlmannii* Kraenzl. (P. stu2)

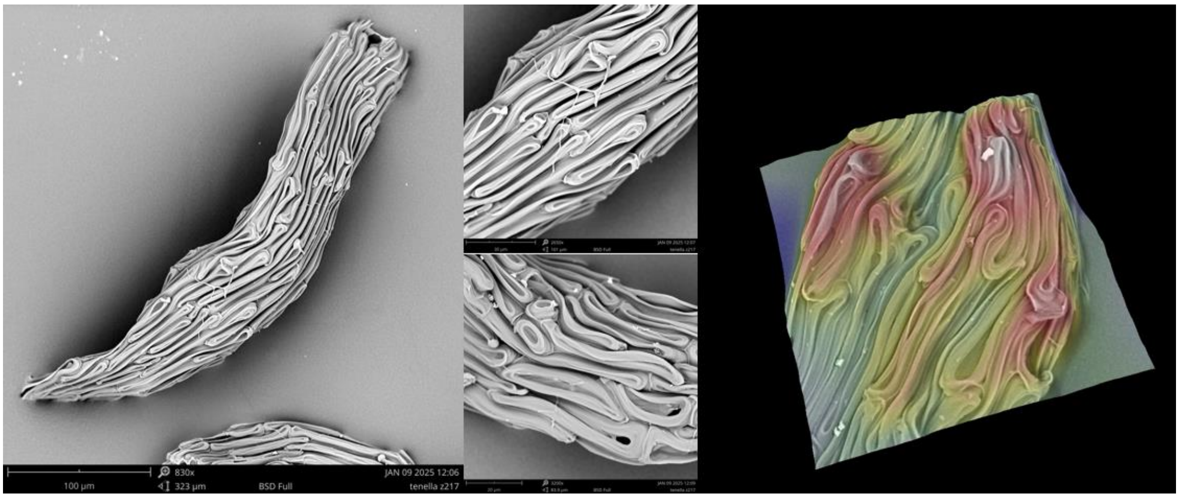

Fig.S170. *Polystachya tenella* Summerh. (P. ten)

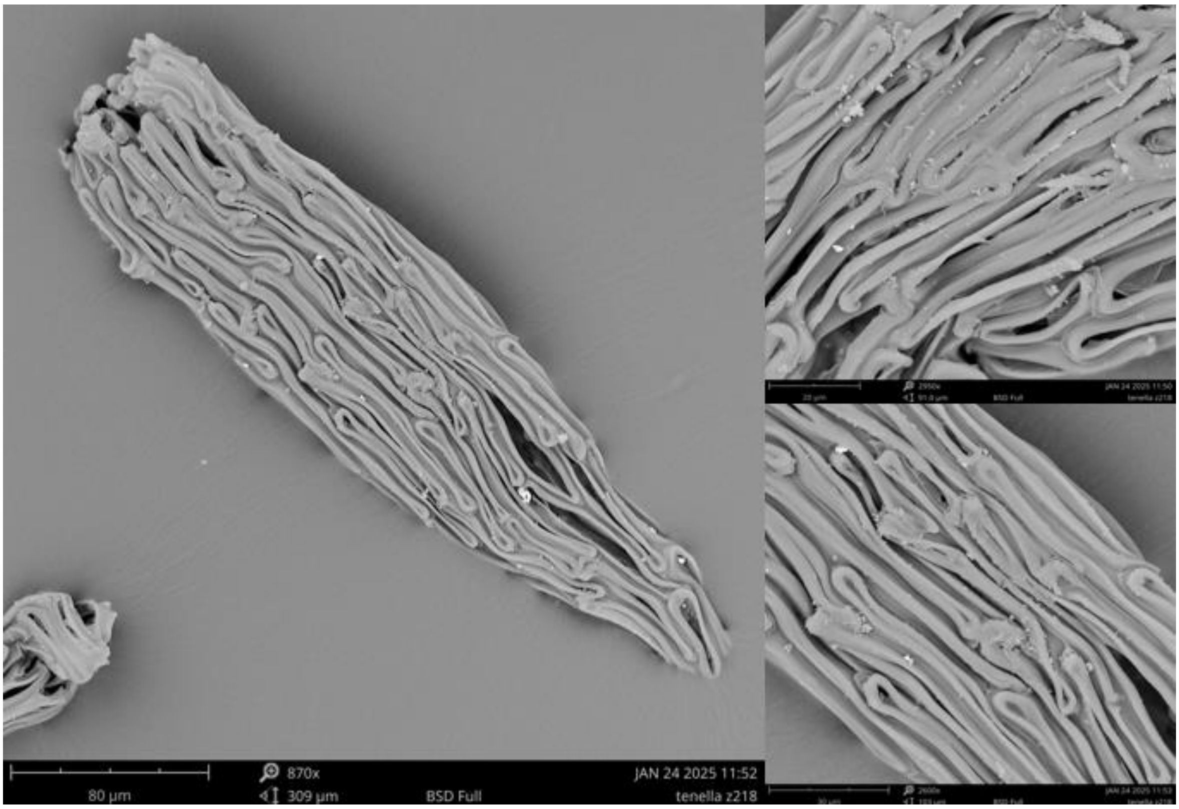

Fig.S171. *Polystachya tenella* Summerh. (P. ten2)

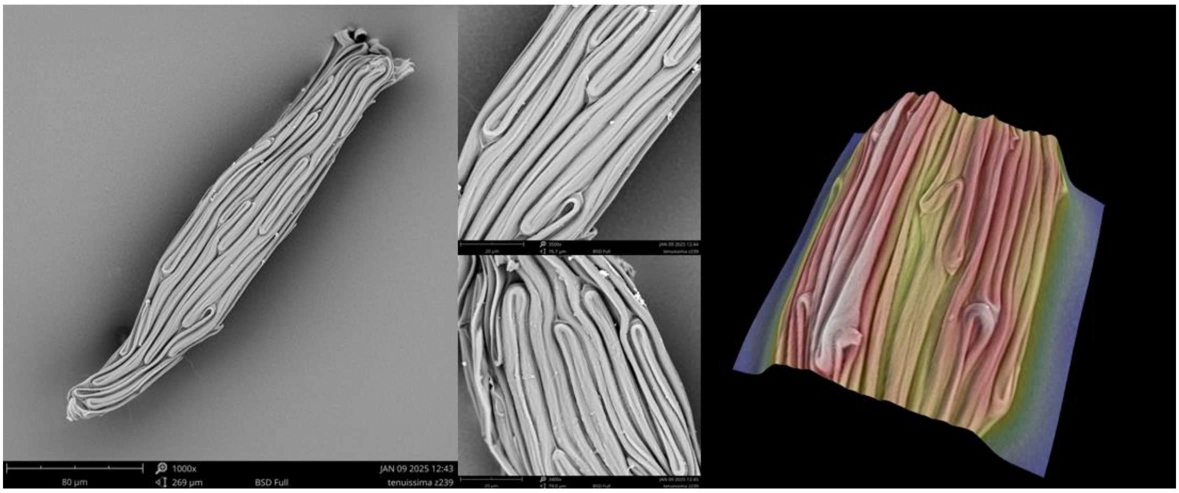

Fig.S172. *Polystachya tenuissima* Kraenzl. (P. tenu)

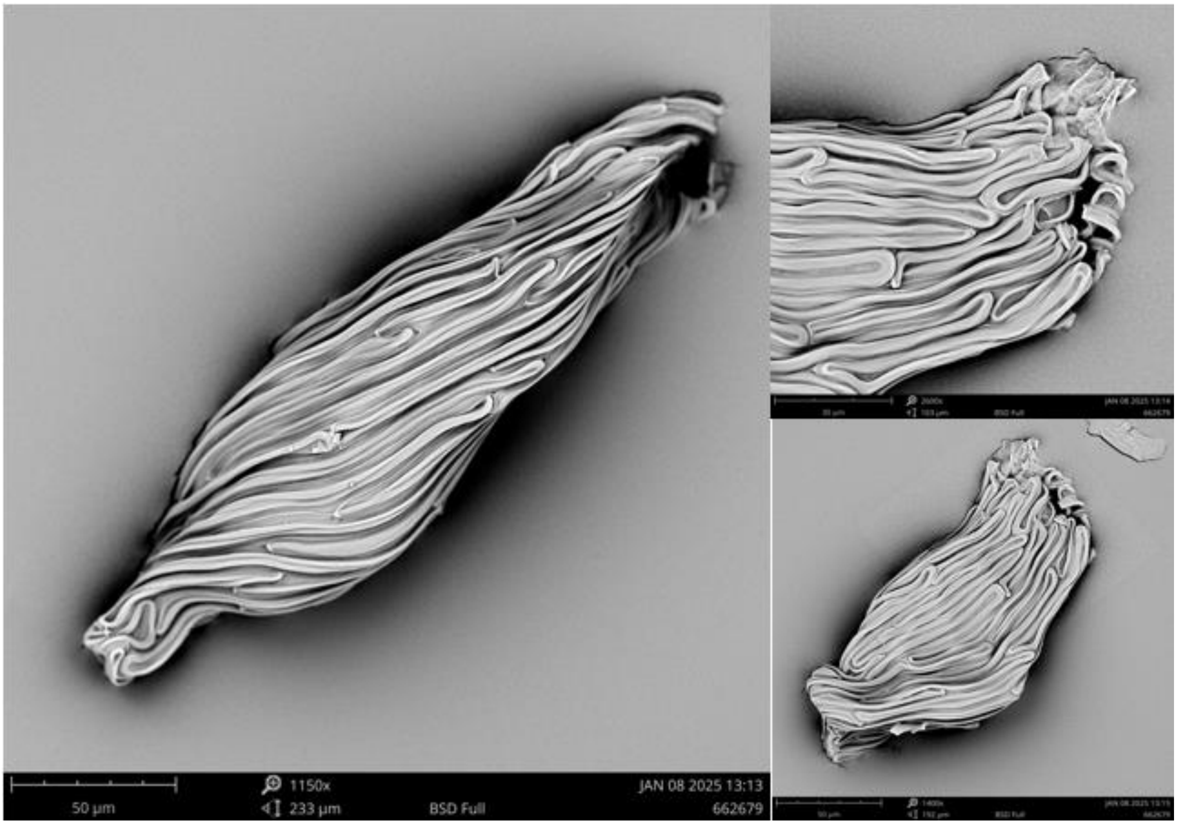

Fig.S173. *Polystachya transvaalensis* Schltr. (P. tra)

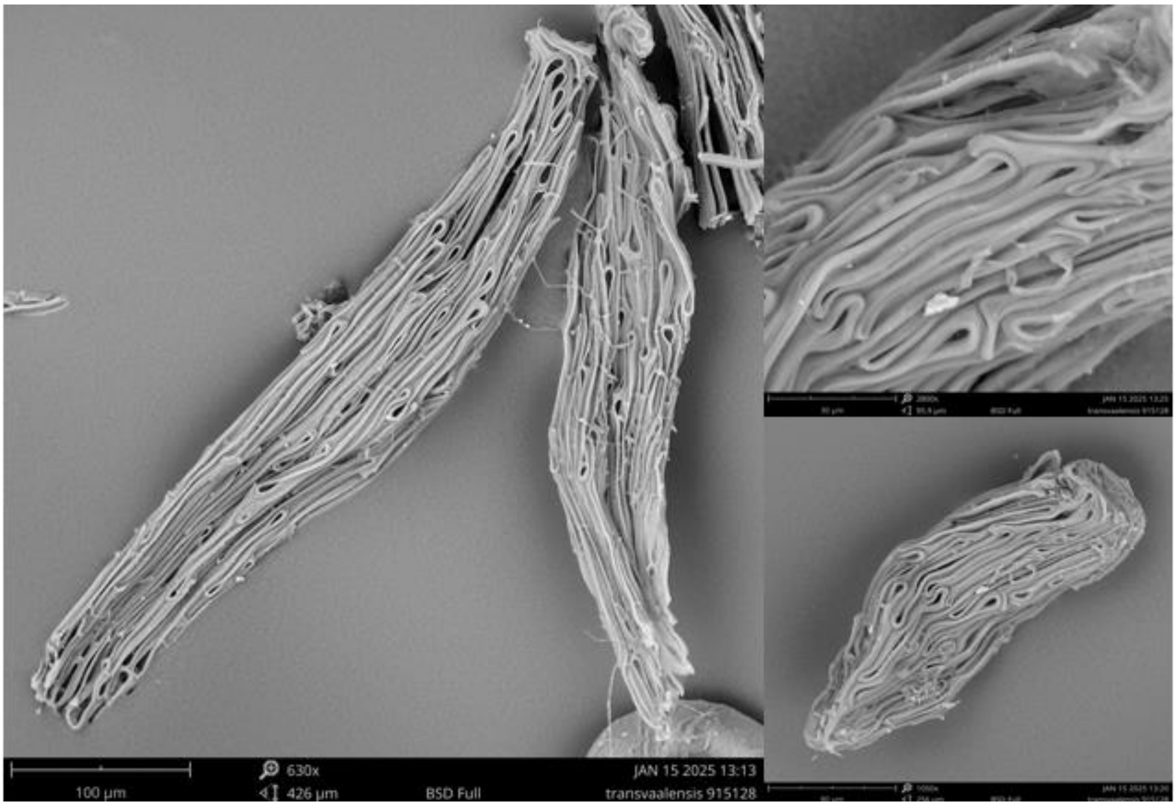

Fig.S174. *Polystachya transvaalensis* Schltr. (P. tra2)

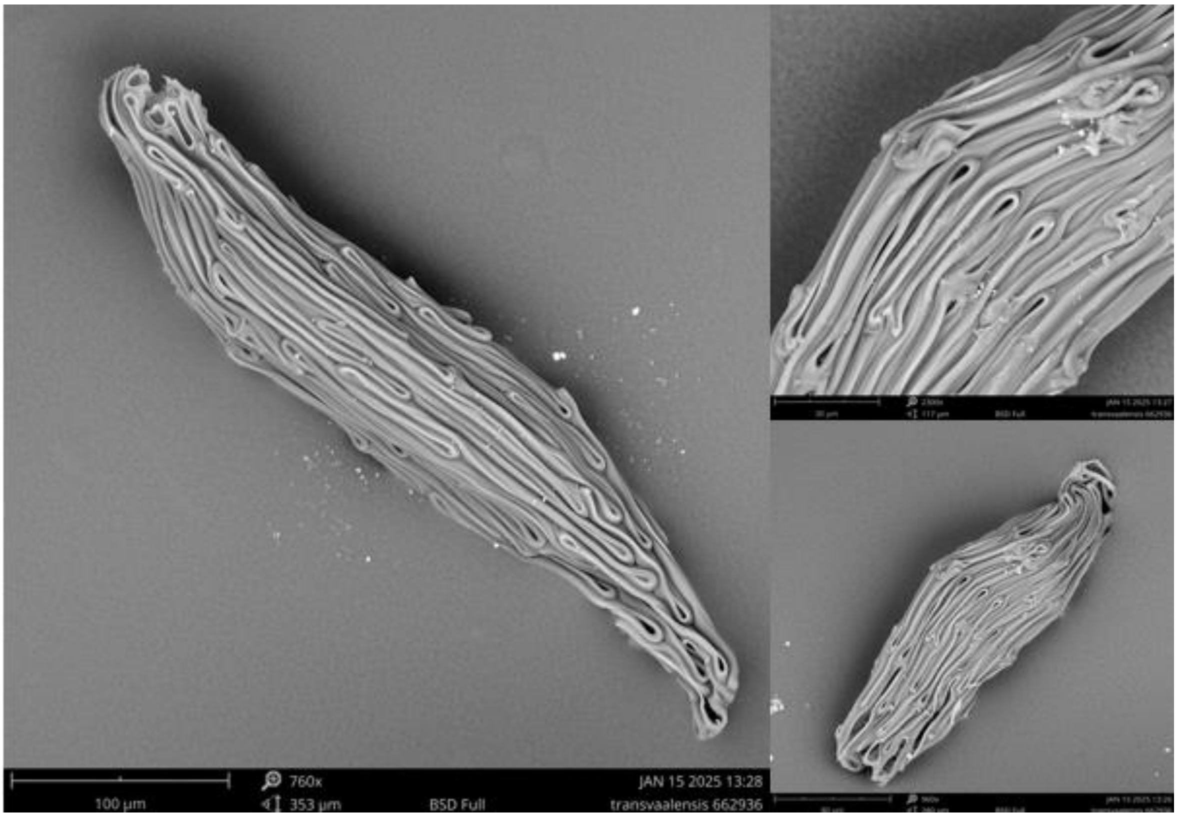

Fig.S175. *Polystachya transvaalensis* Schltr. (P. tra3)

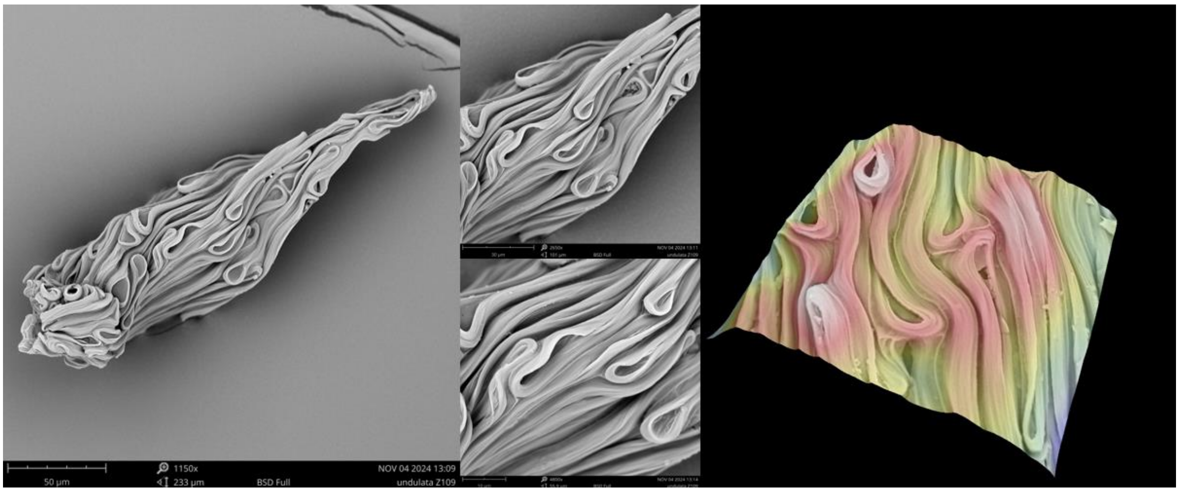

Fig.S176. *Polystachya undulata* P.J.Cribb & Podz. (P. und)

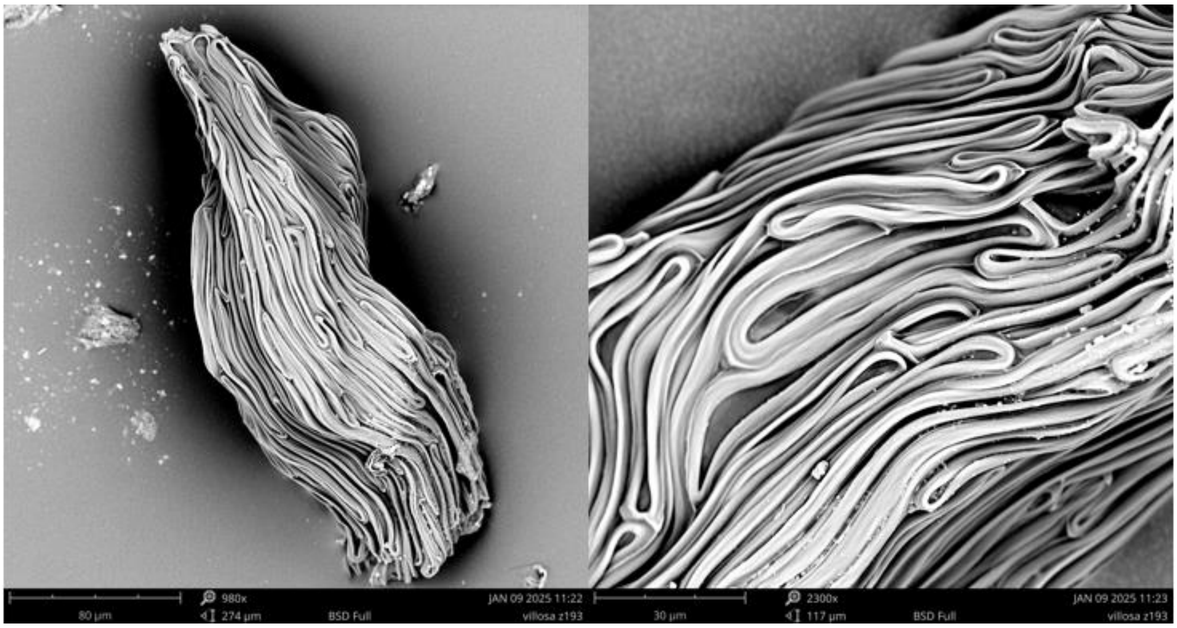

Fig.S177. *Polystachya villosa* Rolfe (P. vil)

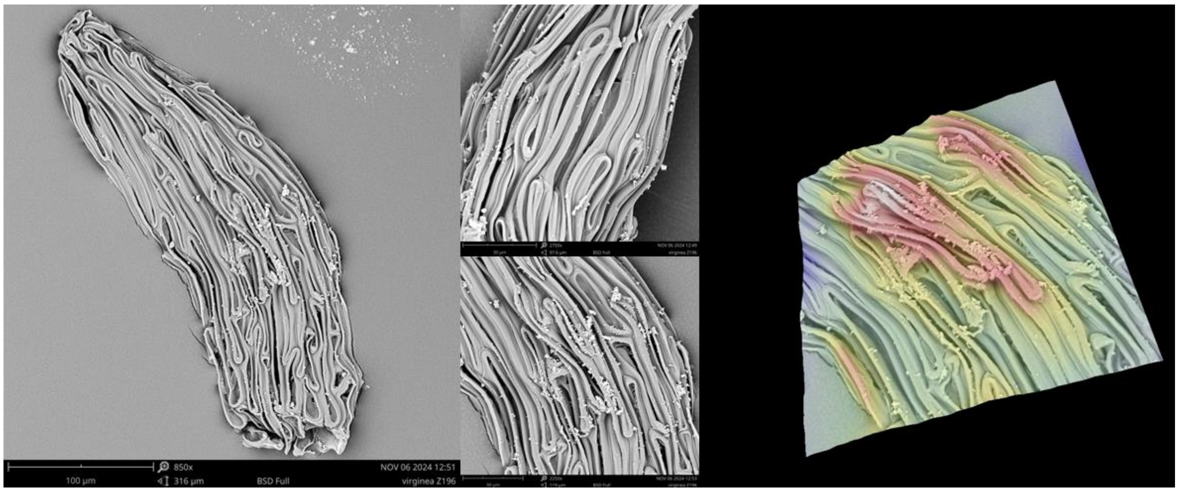

Fig.S178. *Polystachya viriginea* var. *viriginea* (P. vir. var. vir)

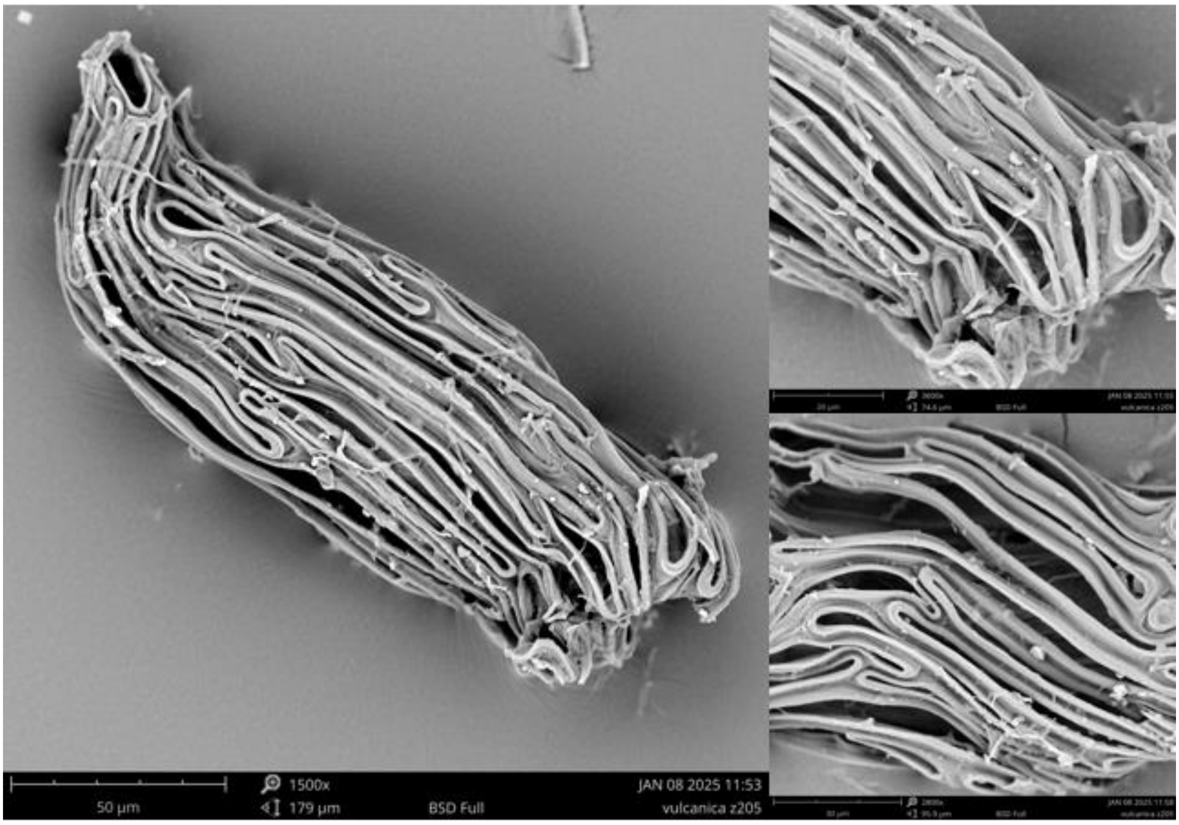

Fig.S179. *Polystachya vulcanica* Kraenzl. (P. vul)

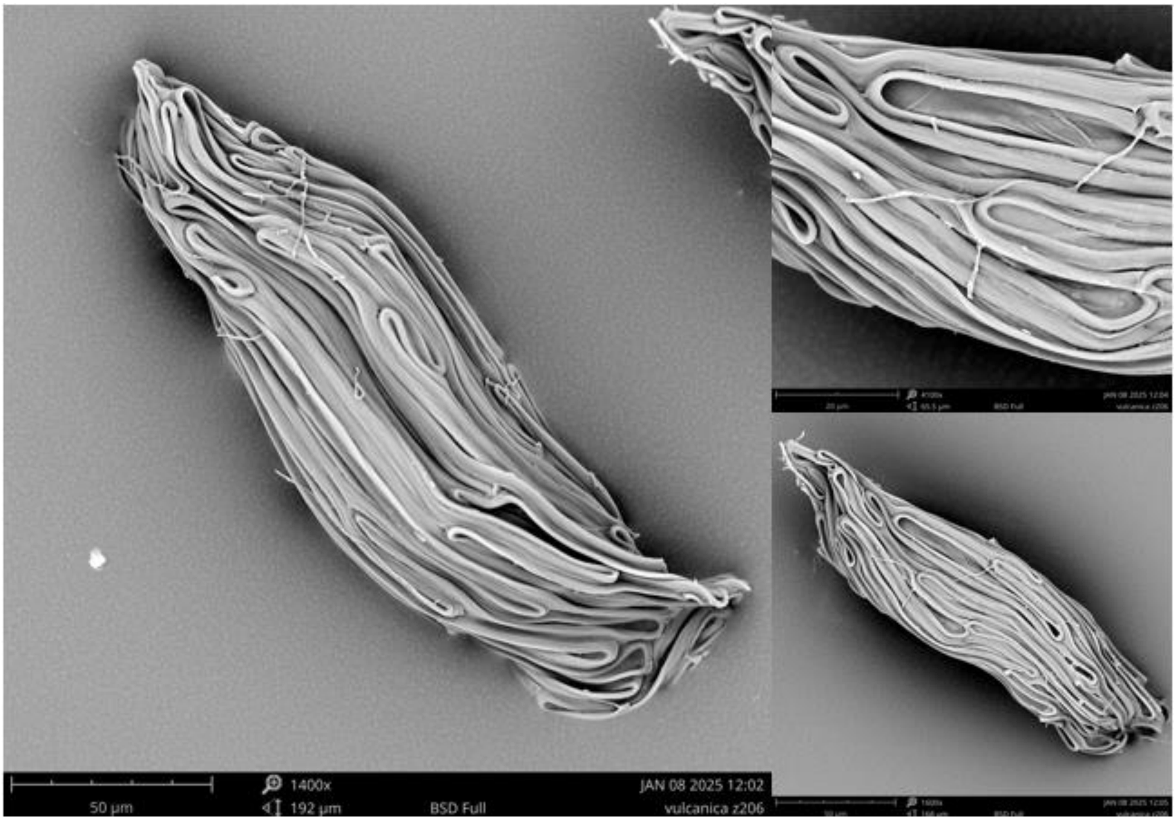

Fig.S180. *Polystachya vulcanica* Kraenzl. (P. vul2)

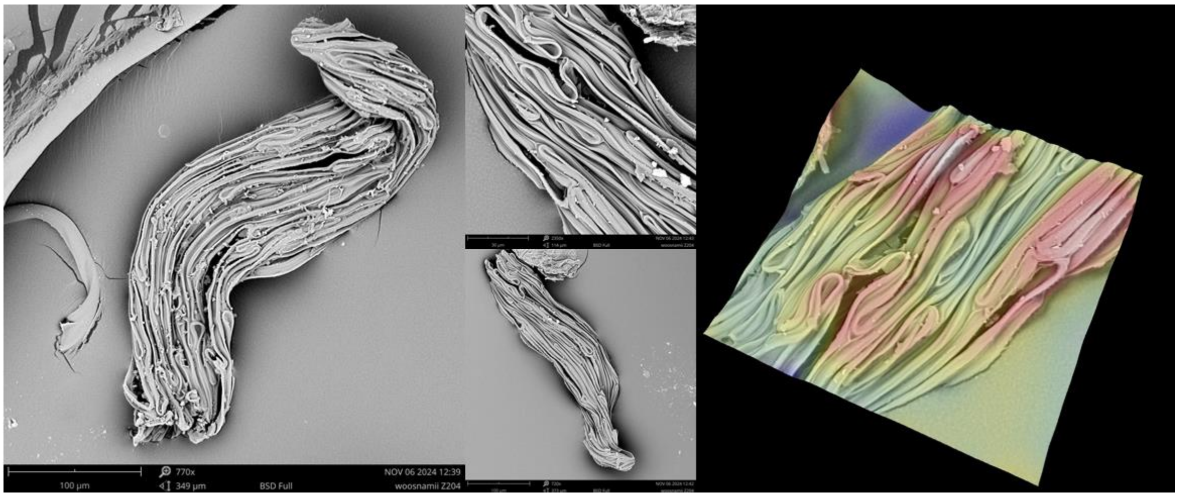

Fig.S181. *Polystachya woosnamii* Rendle (P. woo2)

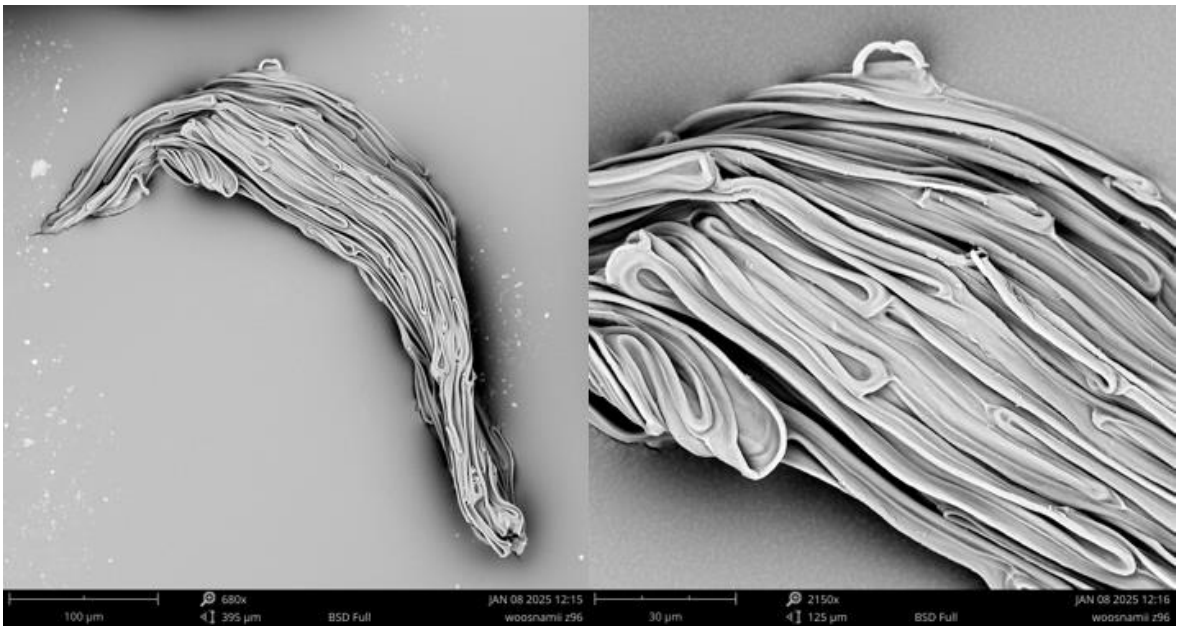

Fig.S182. *Polystachya woosnamii* Rendle (P. woo3)

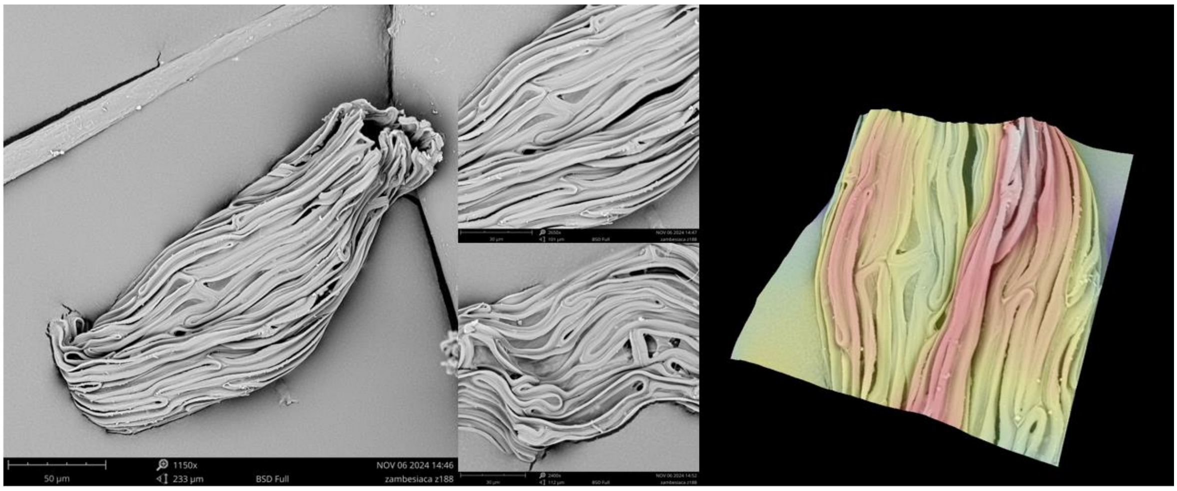

Fig.S183. *Polystachya zambesiaca* Rolfe (P. zam2)
