# Supplementary material for: Application of seed micromorphology in taxonomy of the genus Polystachya Hook. (Vandeae, Orchidaceae)
Source: Front Plant Sci. 2026 May 18;17:1761768. doi: 10.3389/fpls.2026.1761768 (PMC13223086; doi:10.3389/fpls.2026.1761768)
Supplement: Supplementary file 2 [file DataSheet2.pdf]

**Supplementary Material 1.**

PCA 3D and PC1 vs PC2 with convex hull of Polystachya concreta

https://figshare.com/s/70ab2a60e37972078401
